# Nonplanar Tub-Shaped Benzocyclooctatetraenes via Halogen-Radical Ring Opening of Dihydrobiphenylenes

**DOI:** 10.1021/acs.orglett.1c01881

**Published:** 2021-07-06

**Authors:** Jesús Bello-García, Damián Padín, Jesús A. Varela, Carlos Saá

**Affiliations:** Centro Singular de Investigación en Química Biolóxica e Materiais Moleculares (CiQUS), Departamento de Química Orgánica, Universidade de Santiago de Compostela, 15782 Santiago de Compostela, Spain

## Abstract

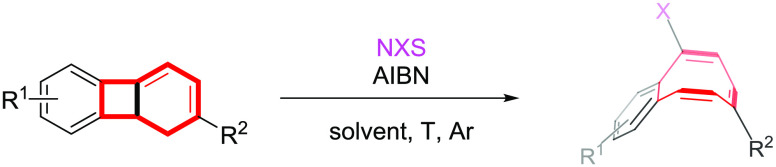

A novel tandem Ru-catalyzed [2+2+2] cycloaddition of arylenynes to dihydrobiphenylenes followed by halogen-radical ring opening has been developed for the construction of tub-shaped halogenated benzocyclooctatetraenes (bCOT’s). Cross-couplings and Diels–Alder reactions of the brominated bCOT’s allow the formation of the corresponding eight-membered ring-fused PAH’s. The halogen-radical ring opening probably occurs via a selective formation of a bis-allyl radical at the 1,3-cyclohexadiene moiety, halogenation at the bridgehead carbon, and finally electrocyclic ring opening.

Cyclooctatetraenes (COT) are nonplanar tub-shaped hydrocarbon compounds having a *D*_2*d*_ conformation (more stable in its dynamic equilibrium than the planar *D*_4*h*_ and delocalized *D*_8*h*_ conformations) that have attracted a great deal of interest due to their electronic properties that result from having cyclic conjugated eight-π-electron systems.^1^ They are also very useful sterically demanding ligands for metals.^2^ These important features triggered an enormous effort throughout the years that aimed to develop and efficient synthesis of these archetypical medium-sized carbocycles^3^ with the aim of understanding their aromatic and antiaromatic properties according to Hückel’s rules.^4^ More recently, nanographenes containing nonhexagonal rings are being considered as ideal models of defective graphene for building new semiconductor materials.^5^ In particular, distortion from planarity caused by the presence of eight-membered rings or the introduction of [8]circulene moieties that induce a deep curvature in the aromatic lattice and deeply influence the electronic and optical properties has attracted considerable attention.^6^ Consequently, the development of efficient synthetic methods for COT-embedded arenes is greatly significant and in high demand. In this context, synthetic approaches to dbCOT’s,^7^ dbCOTP’s,^8^ tribCOT’s,^9^ and tetraphenylenes^10^ are relatively well studied while the simple benzocyclooctatetraenes (bCOT’s) have received significantly less synthetic attention (Scheme 1).^11^ The parent benzocyclooctatetraene unit had also been observed in pioneer Günther’s^12^ studies of Birch reduction of biphenylene in which the double protonation of the dianion occurred at the bridgehead position giving 4a,8b-dihydrobiphenylene.^13^ This reactive species very rapidly evolved to the more stable benzocyclooctatetraene via thermal electrocyclic ring opening (Scheme 1).

**Scheme 1:**
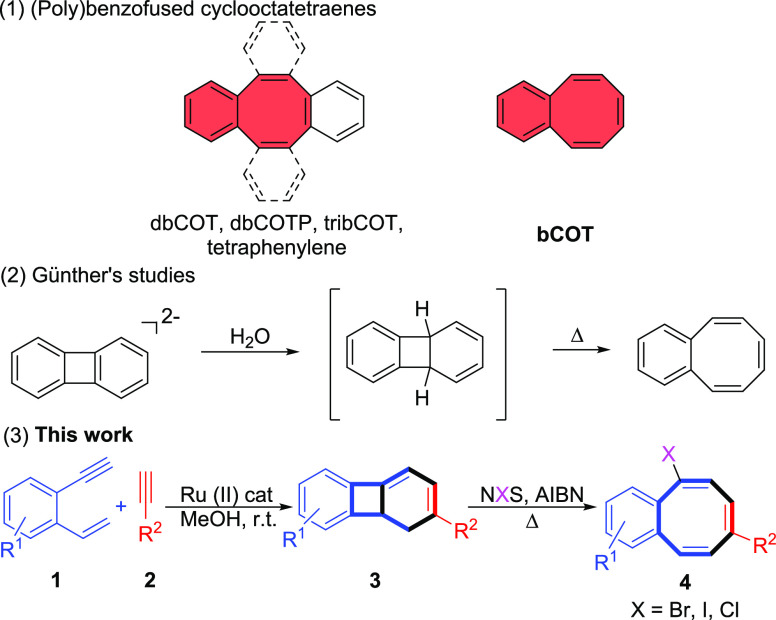
COT-Embedded Polycyclic Arenes, Birch Reduction of Biphenylene, and Formation of Cyclooctatetraenes by Halogen-Radical Ring Opening of 1,8b-Dihydrobiphenylenes

**Figure 1:**
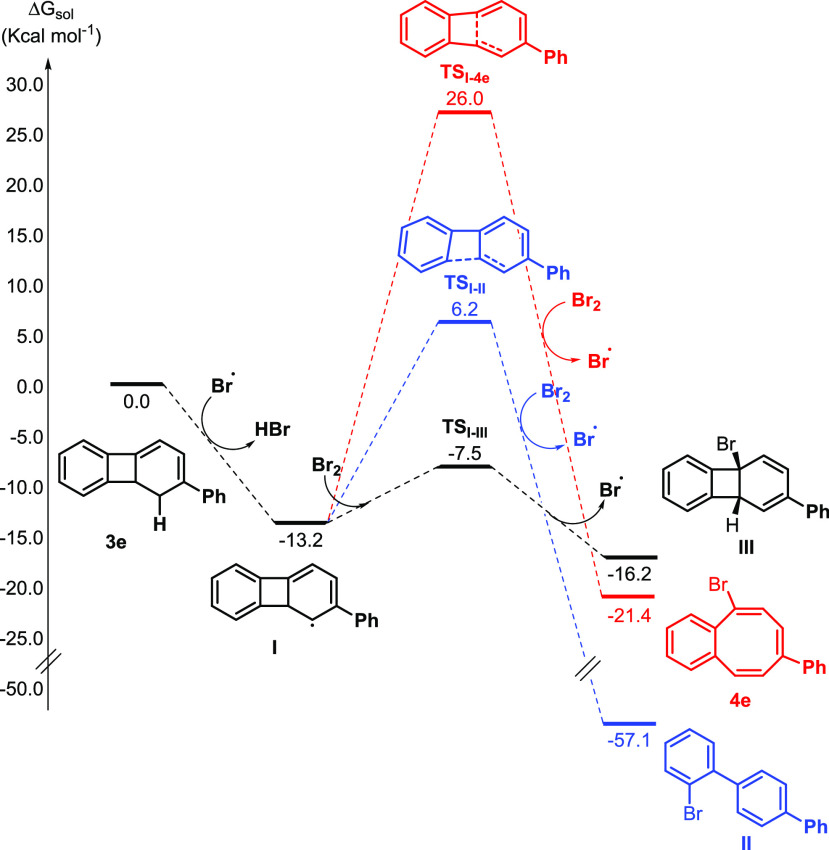
Free energy profiles for the radical bromination of **3e**. Energies are relative to **3e** and are mass balanced.

On the contrary, a mild and powerful method for assembling 1,3-cyclohexadiene units (dihydrobiphenylene isomers) had been recently developed in our group via Ru(II)-catalyzed [2+2+2] cycloaddition of arylenynes and alkynes.^14^ This type of cyclohexadiene has been utilized in efficient synthetic manipulations such as oxidations and Diels–Alder reactions.^15^ Moreover, a tandem Ru-catalyzed [2+2+2] cyclization/iodine-mediated ring expansion of enediynes led to a straightforward assembly of benzo-fused bridged ketones.^16^ However, to the best our knowledge, the radical opening of benzo-fused cyclohexadienes has not been investigated even though such combined processes have synthetic potential for accessing interesting functionalized scaffolds. Herein, we report an efficient tandem process based on a Ru-catalyzed [2+2+2] cycloaddition of arylenynes **1** with alkynes **2** to 1,8b-dihydrobiphenylenes **3**(14) followed by halogen-radical ring opening to benzocyclooctatetraenes **4** (Scheme 1). The halogenated (mainly, bromo derivatives) bCOT’s have proved to be privileged functionalized structural units for accessing PAH’s that combine aromatic and antiaromatic properties.^17^

Inspired by Günther’s observations, we began our investigation by examining the well-known Wohl–Ziegler bromination^18^ of dihydrobiphenylene **3a**. Thus, as a proof of concept, the use of NBS and AIBN as radical initiators in CCl_4_ at rt promoted the formation of the desired bromobenzocyclooctatetraene **4a**, although in low yield (Table 1, entry 1). Gratifyingly, when the reaction temperature is increased at reflux, the yield of **4a** increases to 89% (Table 1, entry 2). Other solvents were then tested. The use of chlorinated solvents like CHCl_3_ or CH_2_Cl_2_ or nonpolar heptane or polar CH_3_CN afforded **4a** but in lower yields (Table 1, entries 3–6). By contrast, polar ethereal or aprotic solvents such as 1,4-dioxane or DCE and a nonpolar solvent like benzene gave **4a** in fairly good yields (Table 1, entries 7–9). Experimental reaction conditions using CCl_4_ as a solvent were then examined. Thus, performing the reaction in the absence of light led to a lower yield of **4a** (Table 1, entry 10) as did not using AIBN as a radical initiator (Table 1, entry 11). In addition, the presence of NBS is mandatory for the consumption of starting product **3a**, while the rest gave rise to a complex mixture (Table 1, entry 12).^19^ The use of other halogen sources (NIS and NCS) is also feasible, affording the corresponding iodinated (**4a′**) and chlorinated (**4a″**) benzocyclooctatetraenes albeit in lower yields (Table 1, entries 13 and 14).

**Table 1:**
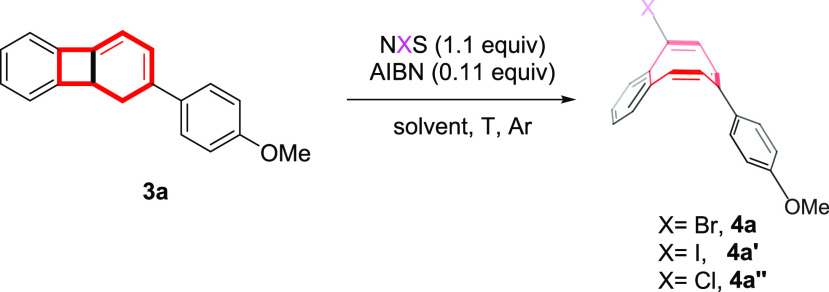
Optimization of Halogen-Radical Ring Opening of 1,8b-Dihydrobiphenylene **3a** to Halobenzocyclooctatetraenes **4a** (X = Br), **4a′** (X = I), and **4a″** (X = Cl)^a^

entry	solvent	*T* (°C)	yield of **4a**^b^
1	CCl_4_	rt	30
2	CCl_4_	reflux	85
3	DCM	reflux	32
4	CHCl_3_	reflux	38
5	heptane	reflux	44
6	CH_3_CN	reflux	40
7	1,4-dioxane	reflux	61
8	DCE	reflux	66
9	benzene	reflux	65
10	CCl_4_ (darkness)	reflux	53
11	CCl_4_ (no AIBN)	reflux	30
12	CCl_4_ (no NBS)	reflux	SM (50)
13^c^	CCl_4_	reflux	77, **4a′**
14^d^	CCl_4_	reflux	36, **4a″**

a Reaction conditions: **3a** (0.2–0.3 mmol) in solvent (0.036 M), NBS (1.1 equiv), AIBN (0.11 equiv), 1–1.5 h.

b Isolated yield.

c NIS.

d NCS.

With the optimized conditions in hand, we next investigated the scope of the reaction (Scheme 2). For dihydrobiphenylenes **3** arising from electron-rich arylalkynes **2** and arylenyne **1a** (R^1^ = H), either the trialkoxyphenyl **3b**, the 6-methoxynaphthyl **3c**, or the heteroaryl 3-thiophene **3d** behaves similarly giving fairly good yields of the corresponding bCOT’s **4b–d**. Not unexpectedly, the parent phenyl dihydrobiphenylene **3e** affords the benzocyclooctatetraene **4e** in a moderate yield (48%), probably due to the lower electron richness of the influential aryl ring involved in the electrocyclic opening.^20^ Curiously, with an extended conjugated π-system, such as in dihydrobiphenylene **3f**, the ring opening was favorably affected giving rise to the biphenyl benzocyclooctatetraene **4f** in a fairly good yield. On the contrary, dihydrobiphenylenes **3** arising from the electron-rich dialkoxy arylenyne **1b** (R^1^/R^1^ = OCH_2_O) and electron-rich alkynes **2** gave rise to the corresponding benzocyclooctatetraenes **4g–i** in moderate to good yields, showing the versatility of combining one or two electron-rich partners. Interestingly, the vinyl substituent on dihydrobiphenylene **3j**, derived from Ru-catalyzed dimerization of 1-ethynyl-4-methoxy-2-vinylbenzene **1c**,^14^ or the ethynyl substituent on **3k** [from Ru-catalyzed cycloaddition of **1d** (R^1^ = alkynyl) and **2a**] remained intact under the radical conditions giving the corresponding styrenic bCOT **4j** and acetylenic bCOT **4k** in fairly good yields that might be capable of future manipulations.^21^

**Scheme 2:**
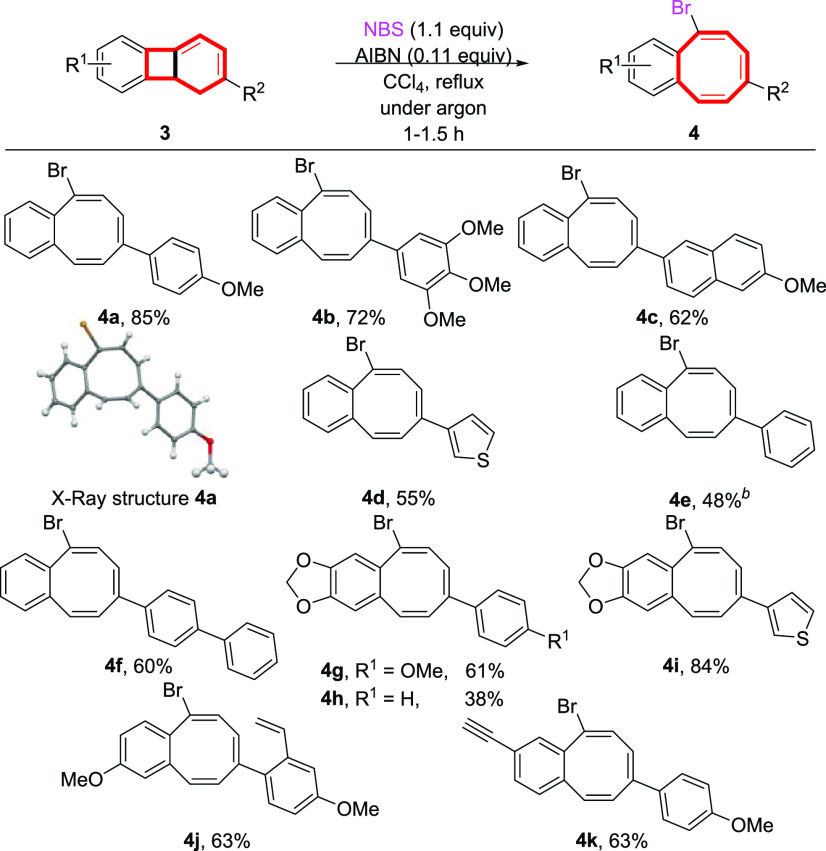
Radical Ring Opening of Dihydrobiphenylenes **3** to bCOT’s **4** Reaction conditions: 3 (0.2−0.3 mmol) in CCl_4_ (0.036 M), NBS (1.1 equiv), AIBN (0.11 equiv), 1−1.5 h. Isolated yield. The ORTEP drawing of **4a** shows ellipsoids at the 50% contour probability level. The reaction time was 4 h.

Interestingly, the heteroannulated benzocyclooctatetraenes **6a** and **6b** could be assembled in moderate yields via a one-pot, two-step process from arylenynes **5a** and **5b** bearing an O and a NTs group as linkers (Scheme 3, eq 1).^16^ Double tandem processes were also accessible. Thus, a simple and straightforward entry to the linear benzodiCOT **9** (benzo[1,2:4,5]di[8]annulene),^22^ an appealing nonbenzenoid PAH structure with intriguing electronic and aromatic properties,^23^ was achieved from 1,4-diethynyl-2,5-divinylbenzene **7**. The double Ru-catalyzed [2+2+2] cycloaddition of **7** with alkyne **2a** led to the linear tetrahydro[3]phenylene **8** in an excellent 80% yield. The halogen-radical double ring opening of **8** with NBS in DCE occurred uneventfully to give the benzodiCOT **9** in a satisfactory 62% yield as a mixture of U- and S-shaped conformers in solution, the S-shaped form being 1.2 kcal mol^–1^ more stable than the U-shaped form as shown by DFT calculations (Scheme 3, eq 2).^24^^1^H NMR spectra of **9** reveal the presence of the two conformers at rt in a 1:2.5 ratio, U- and S-shaped, which could be thermally equilibrated to 1:1.5 ratio at 100 °C. Single crystals of **9** suitable for X-ray diffraction analysis were grown from a solution in a hot CHCl_3_/hexane mixture by slow evaporation of the solvents. **9** shows an S-shaped geometry with the bromine atoms on opposite faces with respect to the central benzene plane. In addition, the two eight-membered rings are considerably bent up and down from the plane of the central benzene unit with a large dihedral angle of ∼138°. Similar to COT, the two eight-membered rings adopt a tub-shaped conformation, with large bond length alternation. The bonds of the central six-membered rings are within the typical range of 1.39–1.40 Å, revealing an aromatic benzenoid character. Scaling up was also feasible as shown by performing a tandem process from initial enyne **1a** and arylalkyne **2a** without the isolation of dihydrobiphenylene **3a**. Thus, reaction of **1a** (8.1 mmol) and **2a** (9.7 mmol) in MeOH under catalytic conditions (as little as 3% Ru) followed by a rapid replacement of the solvent with the apolar DCE to perform the radical reaction allowed us to obtain bCOT **4a** (2.1 g) in a 76% overall yield (Scheme 3, eq 3).

**Scheme 3:**
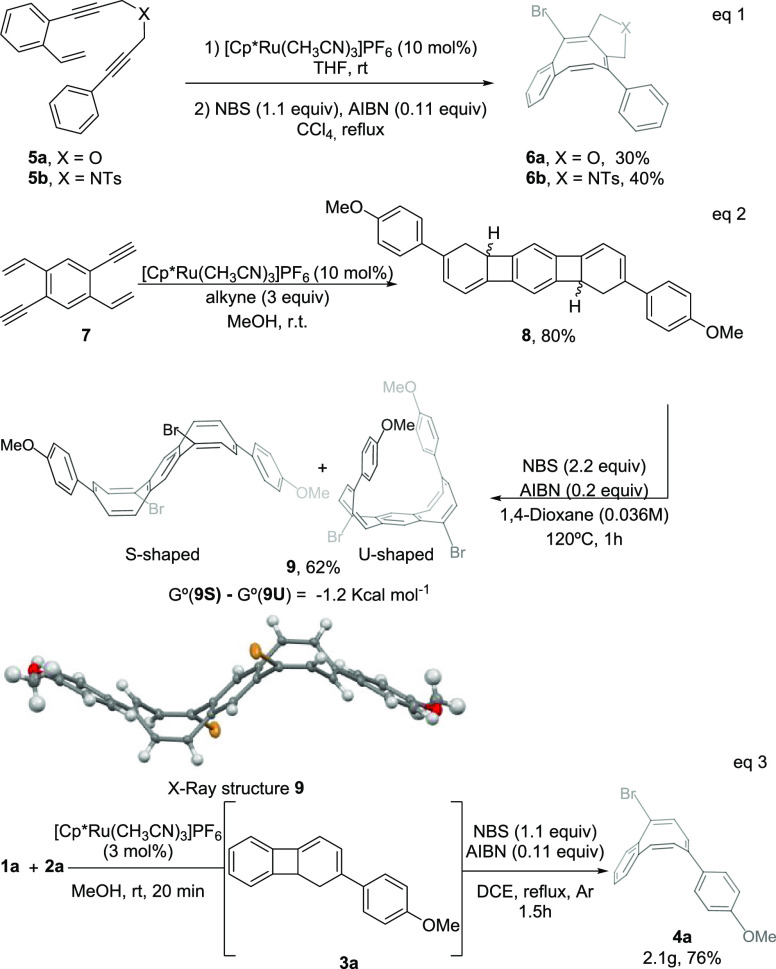
Heteroannulated bCOT’s **6a** and **6b**, Linear BenzodiCOT **9**, and Scale-Up Synthesis of bCOT **4a** The ORTEP drawing of **9** shows ellipsoids at the 50% contour probability level.

In an effort to gain further insights into the reaction mechanism, DFT calculations were performed to analyze all possible radical pathways.^24^ We began the mechanistic studies by elucidating the selectivity of the initial radical formation because two different radicals can be formed depending on the abstractions of the tertiary hydrogen H^1^ of the cyclobutene moiety or one of the two secondary hydrogens H^2^ on the 1,3-cyclohexadiene core. Even though tertiary C–H bonds are weaker than secondary ones, the presence of the cyclobutene moiety dramatically changes the reactivity of the 1,3-cyclohexadiene core, making the formation of the secondary bis-allylic radical **I** 4.3 kcal mol^–1^ more favorable than that of the allylic benzylic tertiary radical (Scheme 4). Atomic spin densities were then computed for the more stable allylic/secondary radical **I**, showing that, as expected, it is mainly divided among the three carbons of the central six-membered ring.^24^

**Scheme 4:**
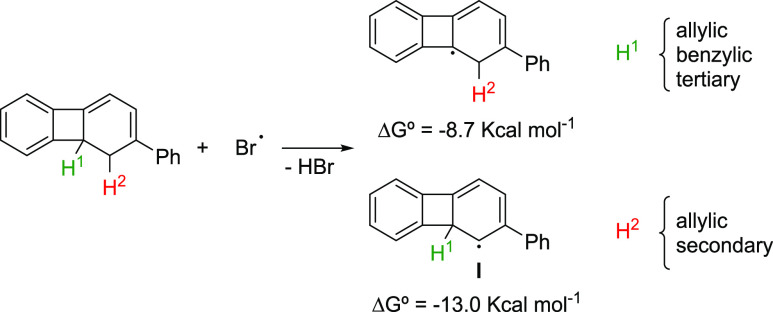
Bond Dissociation Energies (BDE’s) of H^1^ and H^2^

We then evaluate the three possible evolution pathways for the most stable radical **I** (Figure 1): (a) six-π-electron electrocyclic ring opening followed by trapping of the resulting radical with Br_2_ to afford the observed cyclooctatetraene **4e** (Δ*G*^⧧^ = 39.2 kcal mol^–1^, red pathway), (b) radical opening of the cyclobutane ring followed trapping with Br_2_ to afford terphenyl **II** (Δ*G*^⧧^ = 19.4 kcal mol^–1^, blue pathway), and (c) the most favorable one (Δ*G*^⧧^ = 5.7 kcal mol^–1^, black pathway) that involves direct bromination of the resonance structure of **I** with the radical into the tertiary, allylic, and benzylic position to give rise to the brominated dihydrobiphenylene **III**. Once **III** had been established as the most favorable product of radical bromination of **3e**, the observed product **4e** would be formed through a six-π-electrocyclic ring opening.^24^

The utility of the brominated bCOT’s **4** was tested in the preparation of valuable COT-embedded PAH’s (Scheme 5). Suzuki cross-coupling between **4a** and phenylboronic acid affords the expected phenyl-substituted bCOT **10** in 70% yield (Scheme 5, eq 1). Sonogashira couplings were also satisfactorily carried out under typical reaction conditions. Alkynyl-substituted COT’s **11a** and **11b** were obtained in good to excellent yields using trimethylsilylacetylene **2l** and alkynylaniline **2m**, respectively (Scheme 5, eq 2). To our delight, an efficient Sonogashira coupling between **4a** and alkynylCOT **11a′** (from desilylation of **11a**) renders uneventfully the interesting bis-COT derivative **11c**, as confirmed by X-ray analysis (Scheme 5, eq 3).^25^ Finally, treatment of **4a** with KO^t^Bu^10^ generates a strained cyclic alkyne that could be subsequently trapped as a dienophile with tetraphenylcyclopentadienone in a Diels–Alder reaction affording the π-extended dibenzoCOT **12** in very good yield (Scheme 5, eq 4). Note the higher reactivity of the triple bond in planarized systems containing one benzo-fused eight-membered ring (rt, 25 °C) as compared to the typical dibenzo-fused derivative (Ph_2_O reflux, >250 °C).^26^

**Scheme 5:**
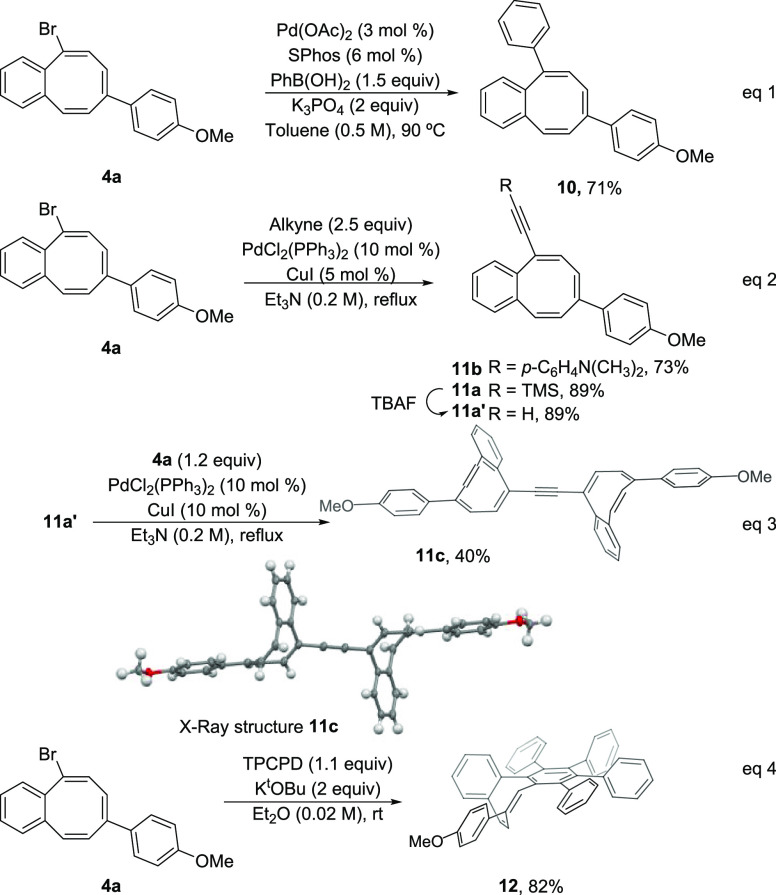
Synthetic Applications of Brominated bCOT’s The ORTEP drawing of **11c** shows ellipsoids at the 50% contour probability level.

In conclusion, we have developed a general synthetic method for constructing a new class of polycyclic arenes embedded with a brominated (halogenated) COT ring via a tandem Ru-catalyzed [2+2+2] cycloaddition of arylenynes to dihydrobiphenylenes followed by halogen-radical ring opening. The process involves the initial formation of a bis-allylic radical at the 1,3-cyclohexadiene core of the dihydrobiphenylene. Then, halogenation at the bridgehead position of the benzocyclobutene ring followed by a subsequent electrocyclic ring opening renders the observed cyclooctatetraene. This protocol provides a new synthetic approach to polycyclic arenes fused with an eight-membered ring (bCOT), which is expected to be applicable for the synthesis of diverse curved nanocarbons.

## Supporting Information Available

General experimental procedures, X-ray crystallographic data, NMR spectra, and DFT calculations (PDF)
Computational details, free energy profile for the isomerization of benzodiCOT **9** from the U- to S-shaped conformers, complete free energy profile for the radical bromination of dihydrobiphenylene **3e** and six-π-electron electrocyclic ring opening of brominated dihydrobiphenylene **III**, natural bond orbital analysis, references, and Cartesian coordinates, energy values, and imaginary frequencies for all of the stationary points involved throughout the DFT study (PDF)

## Accession Codes

CCDC 2085992–2085994 contain the supplementary crystallographic data for this paper. These data can be obtained free of charge via www.ccdc.cam.ac.uk/data_request/cif, or by emailing data_request@ccdc.cam.ac.uk, or by contacting The Cambridge Crystallographic Data Centre, 12 Union Road, Cambridge CB2 1EZ, UK; fax: +44 1223 336033.
