# Supplementary material for: Nonplanar Tub-Shaped Benzocyclooctatetraenes via Halogen-Radical Ring Opening of Dihydrobiphenylenes
Source: Org Lett. 2021 Jul 6;23(14):5539–44. doi: 10.1021/acs.orglett.1c01881 (PMC8499027; doi:10.1021/acs.orglett.1c01881)

Nonplanar Tub-shaped Benzocyclooctatetraenes via Halogen-Radical Ring Opening of Dihydrobiphenylenes

Jesús Bello-García, Damián Padín, Jesús A. Varela and Carlos Saá*

Centro Singular de Investigación en Química Biolóxica e Materiais Moleculares (CiQUS), Departamento de Química Orgánica, Universidade de Santiago de Compostela, 15782 Santiago de Compostela, Spain

Supporting Information

Table of contents

1 General experiments procedure 2

2 Synthesis of starting materials 3

2.1 Synthesis of 2,4-diethynyl-1-vinylbenzene **1d** 3

2.2 Synthesis of 1,4-diethynyl-2,5-divinylbenzene **7** 6

3 Ru-catalyzed [2+2+2] cycloaddition of enynes **1** with alkynes **2** 8

3.1 General procedure 8

3.2 Synthesis of dihydrobiphenylenes **3a-b**, **3f-i**, **3k** 8

4 Ru-catalyzed [2+2+2] cycloaddition of enyne **7** and alkyne **2a** 13

5 Radical ring opening of dihydrobiphenylenes **3** to halogenated benzofused cyclooctatetraenes (bCOT) **4** 13

5.1 General procedure 13

5.2 Synthesis of bCOT's **4a-k**, **4a'** and **4a''** 14

6 Sequential preparation of halogenated benzofused cyclooctatetraenes **6a-b** 21

7 Radical ring opening of tetrahydrobiphenylene **8** to linear-benzodiCOT **9** 23

7.1 Synthesis of linear-benzodiCOT **9** 23

7.2 Variable-temperature NMR and conformational studies of linear-benzodiCOT **9** 24

8 Sequential preparation and gram scale synthesis of bCOT **4a** 25

9 Derivatization of bCOT **4a** 25

10 X-Ray crystallographic Data 31

10.1 Crystallographic data for compound **4a** 31

10.2 Crystallographic data for compound **9** 32

10.3 Crystallographic data for compound **11c** 33

11 NMR Spectra 35

1 General experiments procedure

All reactions were performed under an inert atmosphere of argon and with anhydrous solvents in glassware oven or flame dried at 80 ºC unless otherwise stated. Commercially available chemicals were purchased from Acros Organics Ltd., Aldrich Chemical Co. Ltd., Alfa Aesar, Fluorochem Ltd., Strem Chemicals Inc. or TCI Europe N.V. chemical companies and used without further purification, unless otherwise stated.

Analytical thin layer chromatography was carried out on silica-coated aluminium plates (silica gel 60 F254 Merck) or on aluminium sheets (aluminium oxide 60 F254 neutral Merck) using UV light as visualizing agent (254 nm) and $KMnO_4$ (solution of 1.5 g of potassium permanganate, 10 g of potassium bicarbonate and 1.25 mL of 10% sodium hydroxide in 200 mL of water) with heat as developing agents. Flash column chromatography was performed on silica gel 60 (Merck, 230-400 mesh) with the indicated eluent.[1]

Complex [Ru (η^5-(C_5Me_5) (CH_3CN)$_3$]PF_6 was synthesized by reduction of [Ru (η^5-(C_5Me_5) Cl_2]$_n$[1] with $LiEt_3BH$ according to published procedures.[2]

^{1}H and ^{13}C nuclear magnetic resonance experiments were carried out using a Varian Inova 500MHz, a Varian Inova 400 MHz, a Varian Mercury 300MHz or a Bruker DPX 250 or a Bruker Avance 300 NMR spectrometers. Chemical shifts are referenced to residual solvent peaks (1H, 13C{1H}). Coupling constants *J* are given in Hertz (Hz). Multiplicities are reported as follows: s = singlet, d = doublet, t = triplet, q = quartet, m = multiplet or as a combination of them. Multiplicities of 13C NMR signals were determined by DEPT experiments.

Mass spectrometry was carried out on a Bruker micro TOF spectrometer.

Yields refer to isolated compounds estimated to be > 95% pure as determined by ^{1}H-NMR and capillary GC analysis.

X-ray crystallographic analysis was performed at the CACTUS facility of the University of Santiago de Compostela.

---

[1]W. C. Still, M.; Kahn, A.; Mitra, *J. Org. Chem.* **1978**, *43*, 2923-2925.
[2]Mbaye, M. D.; Demerseman, B.; Renaud, J-L.; Toupet, L.; B and Bruneau, C. *Adv. Synth. Catal.* **2004**, *346*, 835-841

2 Synthesis of starting materials

Enynes **1a-c**,[3] dihydrobiphenylenes **3a**, **3c-e, 3j**[3] and enedyines **5a-b**[4] were prepared according to the previously reported procedures.

2.1 Synthesis of 2,4-diethynyl-1-vinylbenzene **1d**

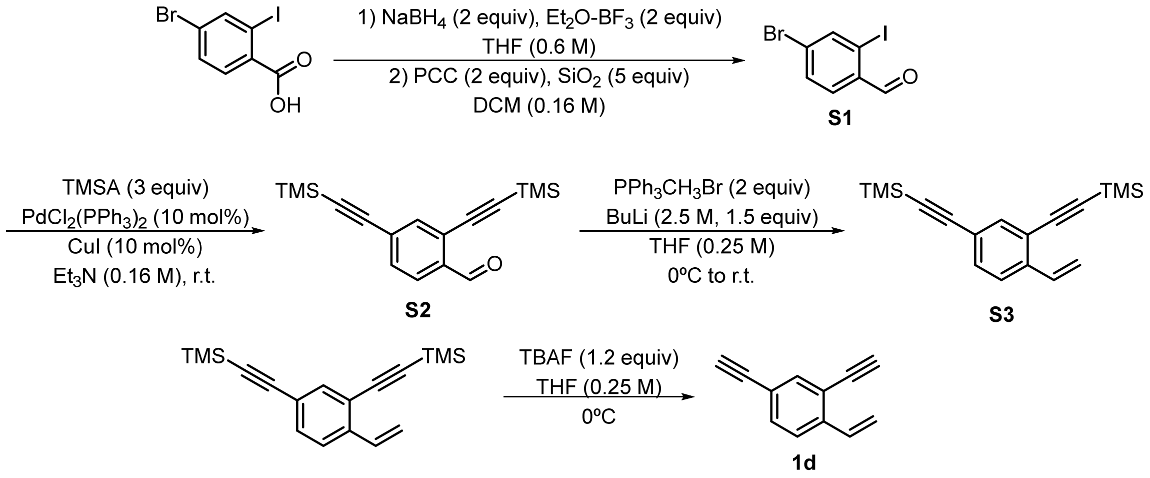

To a solution of 4-bromo-2-iodobenzoic acid (2.88 g, 8.81 mmol) and $NaBH_4$ (0.68 g, 17.6 mmol, 2 equiv) in THF (14 mL) at 0ºC was slowly added $Et_2O\cdot BF_3$ (2.2 mL, 17.6 mmol, 2 equiv) over a period of 30 minutes and then the mixture was vigorously stirred at room temperature. When the reaction was completed as determined by TLC analysis (2h), the reaction mixture was cooled to 0ºC, H_2O was slowly added and then extracted with ethyl acetate (3 × 20 mL). The combined organic layers were dried over anhydrous $MgSO_4$, and the solvent was evaporated in vacuum obtaining the (2-iodophenyl)methanol derivative as a white solid in quantitative yield (2.7 g), that was used without further purification.

PCC (2.01 g, 9.3 mmol, 2 equiv) was slowly added to the mixture of (4-bromo-2-iodophenyl)methanol (1.46 g, 4.6 mmol) and SiO_2 (1.4 g, 24.5 mmol, 2 equiv) in CH_2Cl_2 (0.16 M, 29 mL) at 0ºC. The resulting mixture was allowed to stir at room temperature for additional 2 hours. Then the reaction mixture was filtrated and concentrated under vacuo. The crude residue was purified by flash column chromatography through silica gel using a mixture of Hex/EtOAc (9:1) as eluent to afford 4-bromo-2-iodobenzaldehyde[5] **S1** (1.25 g, 86% yield) as a white solid.

---

[3]García-Rubín, S.; González-Rodríguez, C.; García-Yebra, C.; Varela, J. A.; Esteruelas, M. A.; Saá, C. *Angew. Chem. Int. Ed.* **2014**, *53,* 1841-1844
[4]Yamamoto, Y.; Nishimura, K.; Mori, S.; Shibuya, M. *Angew. Chem. Int. Ed.* **2017**, *56,* 5494–5497
[5] A. C. Jr, J. D. Tovar, *J.Org. Chem.* **2011**, *76*, 2227-223.

Melting point: 82.3-83.3 ºC

^{1}H NMR (300 MHz, $CDCl_3$), δ (ppm): 10.00 (d, J = 0.8 Hz, 1H), 8.14 (d, J = 1.8 Hz, 1H), 7.73 (d, J = 8.3 Hz, 1H), 7.62 (dd, J = 8.3, 2.5 Hz, 1H).

^{13}C NMR, DEPT (75 MHz, $CDCl_3$), δ (ppm): 194.7 (CH), 142.8 (CH), 134.1 (C), 132.3 (CH), 131.2 (CH), 130.2 (C), 100.9 (C).

A round bottomed flask containing 4-bromo-2-iodobenzaldehyde (1.2 g, 3.9 mmol), $Pd(PPh_3)Cl_2$ (0.27 g, 0.39 mmol, 0.1 equiv) and CuI (0.074 mg, 0.39 mmol, 0.1 equiv) was evacuated and refilled with Ar three times. Et_3N (0.16 M, 24 mL) was added and the mixture was stirred during 5 minutes before the addition of TMSA (2.2 mL, 15.4 mmol, 4 equiv). The resulting mixture was stirred at room temperature until disappearance of the starting material (TLC monitoring, 12h). The mixture was filtered through silica gel and the filtrate was evaporated to dryness. The residue was dissolved in EtOAc (20 mL) and washed with brine (20 mL) and a solution of NH_4Cl_{sat} (20 mL). The combined organic layers were dried over anhydrous Na_2SO_4, filtered and evaporated under vacuum. The residue was purified by flash column chromatography through silica gel using a mixture of EtOAc/Hex (5:95) as eluent to afford of the corresponding alkynyl benzaldehyde **S2** (1.07 g, 93% yield) as a white solid.

Melting point: 131.5-132.0 ºC

^{1}H NMR (500 MHz, $CDCl_3$), δ (ppm): 10.50 (d, J = 0.9 Hz, 1H), 7.83 (dd, J = 8.1, 0.6 Hz, 1H), 7.66 (dd, J = 1.6, 0.6 Hz, 1H), 7.47 (ddd, J = 8.1, 1.6, 0.9 Hz, 1H), 0.27 (s, 9H), 0.25 (s, 9H).

^{13}C NMR, DEPT (126 MHz, $CDCl_3$), δ (ppm): 191.2 (CH), 136.9 (CH), 135.3 (C), 132.1 (CH), 129.0 (C), 126.9 (CH), 103.2 (C), 103.2 (C), 99.7 (C), 99.4 (C), -0.1 ($3xCH_3$), -0.12 ($3xCH_3$).

HRMS (APCI) calculated for $C_{17}H_{23}OSi_2$ $[M+H]^+$: 299.1282, found 299.1285

To a solution of methyl(triphenyl)phosphonium bromide (2.39 g, 6.7 mmol, 2 equiv) in THF (10 mL, 0.25 M) cooled at 0ºC, ^{n}BuLi (1.9 mL, 1.4 equiv, 2.5 M) was added dropwise. The resulting mixture was stirred during 45 min and, then, a solution of 2,4-bis((trimethylsilyl)ethynyl)benzaldehyde (1.0 g) in THF (4 mL) was added. After reaching room temperature the mixture was stirred until disappearance of the starting material (TLC monitoring, 1h). A saturated NH_4Cl solution was added to the mixture and

then extracted with EtOAc (4 x 20 mL). The combined organic layers were dried over anhydrous $MgSO_4$, filtered and evaporated to dryness. The residue was purified by flash column chromatography through silica gel using a mixture of Hex/EtOAc (98:2) as eluent to afford ((4-vinyl-1,3-phenylene)bis(ethyne-2,1-diyl))bis(trimethylsilane) **S3** (0.78 g, 79% yield) as a colorless oil.

^{1}H NMR (300 MHz, $CDCl_3$), δ (ppm): 7.72 – 7.66 (m, 1H), 7.58 (d, *J* = 8.6 Hz, 1H), 7.52 – 7.40 (m, 1H), 7.27 (dd, *J* = 17.6, 11.0 Hz, 1H), 5.93 (d, *J* = 17.6 Hz, 1H), 5.49 (d, *J* = 11.0 Hz, 1H), 0.36 (s, 9H), 0.35 (s, 9H).

^{13}C NMR, DEPT (75 MHz, $CDCl_3$), δ (ppm): 139.4 (C), 136.8 (CH), 134.6 (CH), 132.2 (CH), 124.8 (CH), 122.7 (C), 122.4 (C), 117.9 (CH_2), 104.5 (C), 102.7 (C), 100.7 (C), 96.6 (C), 0.34 (6xCH_3).

HRMS (APCI) calculated for $C_{18}H_{25}Si_2$ $[M+H]^+$: 297.1489, found 297.1485

To a solution of **S3** (0.75 g, 2.53 mmol) in THF (10 mL, 0.25 M) cooled at 0ºC TBAF (3 mL, 1.2 equiv, 1M in THF) was added. Then, the mixture was stirred until disappearance of the starting material (TLC monitoring, 1h). Upon completion, a solution of $NH_4Cl_{(sat)}$ was added and the mixture was extracted with Et_2O (3 x 10 mL). The combined organic layers were dried over anhydrous $MgSO_4$, filtered and evaporated under vacuum. The resulting crude was purified by flash column chromatography through silica gel using a mixture of Hex/EtOAc (95/5) as eluent to afford 2,4-diethynyl-1-vinylbenzene **1d** (0.31g, 82%) as a colorless oil.

^{1}H NMR (300 MHz, $CDCl_3$), δ (ppm): 7.62 (d, *J* = 1.8 Hz, 1H), 7.54 (d, *J* = 8.2 Hz, 1H), 7.42 (dd, J = 8.2, 1.8 Hz, 1H), 7.20 (dd, *J* = 17.6, 11.0 Hz, 1H), 5.84 (dd, *J* = 17.6, 0.9 Hz, 1H), 5.42 (dd, *J* = 11.0, 0.9 Hz, 1H), 3.32 (s, 1H), 3.11 (s, 1H).

^{13}C NMR, DEPT (75 MHz, $CDCl_3$), δ (ppm): 140.0 (C), 136.8 (CH), 134.1 (CH), 131.9 (CH), 124.1 (CH), 121.5 (C), 120.2 (C), 117.1 (CH_2), 82.7 (C), 82.5 (CH), 81.0 (C), 78.5 (C).

HRMS (APCI) calculated for $C_{12}H_9$ $[M+H]^+$:153.0699, found 153.0695

2.2 Synthesis of 1,4-diethynyl-2,5-divinylbenzene **7**

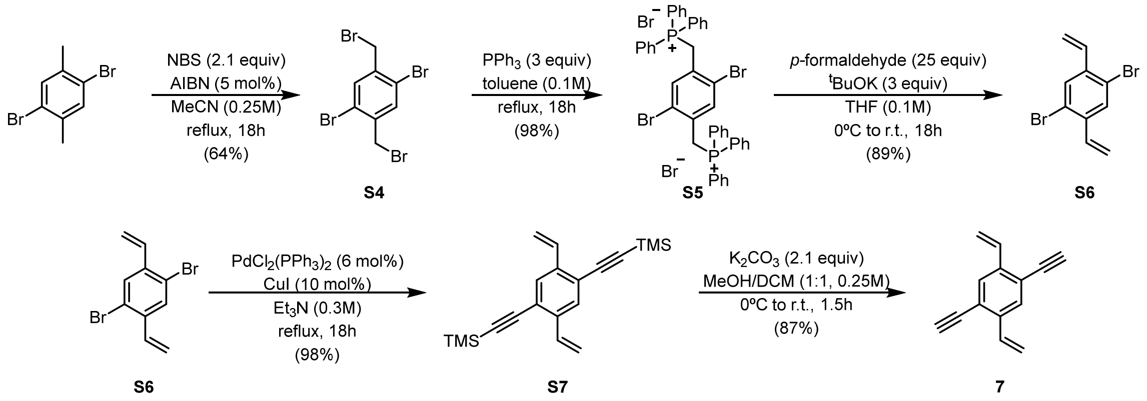

1,4-Dibromo-2,5-dimethylbenzene (5 g, 19 mmol), NBS (7.1 g, 40 mmol, 2.1 equiv) and AIBN (0.164 g, 1 mmol, 0.05 equiv) were dissolved in acetonitrile (75 mL) in a round bottomed flask equipped with a stirring bar. The reaction mixture was heated at 100ºC in an aluminium heating block during 24h. The reaction was then cooled to room temperature and the solvent evaporated under reduced pressure. The crude was washed with hot methanol to yield the tetrabrominated product **S4** (5.1 g, 64% yield) as a white solid. NMR data is in accordance with the previously reported.[6]

^{1}H NMR (300 MHz, $CDCl_3$) δ (ppm): 7.65 (d, *J* = 1.1 Hz, 2H), 4.50 (d, *J* = 1.0 Hz, 4H).

In a round bottomed flask equipped with a stirring bar, **S4** (5 g, 11.8 mmol) and PPh_3 (9.3 g, 35.6 mmol, 3.0 equiv) were dissolved in toluene (119 mL). The solution was then heated at 120 ºC in an aluminium heating block for 19h. Upon completion the reaction was cooled to room temperature, filtered and the resulting solid was washed with hexane. The obtained white solid was dried several hours at high vacuum. 2,5-Dibromo-*p*-xylenebis(triphenylphosphonium) dibromide, **S5**, was obtained as a white powder (10.9 g, 98% yield). NMR data is in accordance with the previously reported.[7]

^{1}H NMR (300 MHz, $CDCl_3$) δ (ppm): 7.86 – 7.78 (m, 6H), 7.76 – 7.62 (m, 24H), 7.39 (d, J = 2.0 Hz, 2H), 5.75 (d, J = 10.3, 4H).

S5 (10.8 g, 11.5 mmol) and *p*-formaldehyde (8.6 g) were dissolved in anhydrous THF (114 mL) in a round bottomed flask equipped with a stiring bar under nitrogen. The solution was cooled at 0ºC in an ice/water bath and, then, anhydrous KO^{t}Bu (4 g, 34.5 mmol, 3 equiv) was added in one portion. The reaction mixture was allowed to warm to

[6] Auffray, M.; Charra, F.; Sosa Vargas, L.; Mathevet, F.; Attias, A.-J.; Kreher, D. *New J. Chem.,* **2020**, *44*, 7665-7674

[7] Jones, D. R.; Point, B.; Levine, M. *J. Phys. Chem. B*, **2019**, *123*, 4604–4610

rt and stirred at the same temperature during 19 h. The solvent was removed at reduced pressure and the crude was purified by flash column chromatography using hexane as solvent to afford the styrene **S6** (2.9 g, 89% yield) as a white solid. NMR data is in accordance with the previously reported.[8]

^{1}H NMR (300 MHz, $CDCl_3$) δ (ppm): 7.71 (s, 2H), 6.95 (dd, *J* = 17.2, 10.4 Hz, 2H), 5.71 (d, *J* = 17.2 Hz, 2H), 5.40 (d, *J* = 10.4 Hz, 2H).

Et_3N (35 mL) was added over a mixture of **S6** (2.9 g, 10.1 mmol), $PdCl_2(PPh_3)_2$ (0.428 g, 0.61 mmol, 0.06 equiv) and CuI (0.194 g, 1 mmol, 0.1 equiv) in a round bottomed flask under argon. The mixture was stirred during 1 min and then TMSA (5.8 mL, 41 mmol, 4 equiv) was added. The reaction mixture was stirred overnight at reflux in an aluminium heating block until disappearance of the starting material (TLC and GC-MS monitoring). The mixture was filtered through silica gel using hexane as eluent and the filtrate was evaporated to dryness. The residue was dissolved in EtOAc and washed with a saturated solution of NH_4Cl (10 mL) and brine (10 mL). The combined organic layers were dried over anhydrous $MgSO_4$, filtered and evaporated under vacuum. The residue was purified by flash column chromatography through silica gel using hexane as eluent to afford the silylated arenyne **S7** (3.2g, 98% yield) as a white solid.

Melting point: 111.0-111.9 ºC

^{1}H NMR (500 MHz, $CDCl_3$) δ (ppm): 7.65 (s, 2H), 7.10 (dd, *J* = 17.6, 11.0 Hz, 2H), 5.85 (dd, *J* = 17.6, 0.9 Hz, 2H), 5.36 (dd, *J* = 11.0, 0.9 Hz, 2H), 0.27 (s, 18H).

^{13}C NMR (126 MHz, $CDCl_3$) δ (ppm): 137.1 (2 x C), 132.7 (2 x CH), 127.9 (2 x CH), 120.9 (2 x C), 115.2 (2 x CH_2), 101.8 (2 x C), 99.6 (2 x C), -1.1 (6 x CH_3).

HRMS (APCI) calculated for $C_{20}H_{27}Si_2$ $[M+H]^+$: 323.1646, found 323.1643

To a solution of **S7** (3.1 g, 9.6 mmol) in MeOH/DCM (1:1, 36 mL) was added solid K_2CO_3 (2.8 g, 20.2 mmol, 2.1 equiv). The reaction mixture was stirred at room temperature until disappearance of the starting material (TLC monitoring, 2h). The solvent was removed under reduced pressure and the residue was dissolved in EtOAc and washed twice with an aqueous solution of HCl 5% and once with brine. The combined organic layers were dried over anhydrous $MgSO_4$, filtered and evaporated to dryness. The

[8] Gagnon, C.; Godin, É.; Minozzi, C.; Sosoe, J.; Pochet, C.; Collins, S.K. *Science*, **2020**, *367*, 917-921

residue was purified by flash column chromatography through silica gel using hexane as solvent to give 1,4-diethynyl-2,5-divinylbenzene **8** (1.54 g, 89% yield) as a white solid.

Melting point: 99.3–100.0 ºC

^{1}H NMR (500 MHz, CDCl$_3$) δ (ppm): 7.70 (s, 2H), 7.13 (dd, J = 17.6, 11.0 Hz, 2H), 5.83 (dd, J = 17.6, 0.8 Hz, 2H), 5.39 (dd, J = 11.0, 0.8 Hz, 2H), 3.38 (s, 2H).

^{13}C NMR (126 MHz, CDCl$_3$) δ (ppm): 137.5 (2x C), 132.5 (2 x CH), 128.3 (2 x CH), 120.4 (2 x C), 115.6 (2 x CH_2), 82.0 (2 x CH), 80.0 (2 x C).

HRMS (APCI) calculated for $C_{14}H_{11}$ [M+H]$^+$: 179.0855, found 179.0854

3 Ru-catalyzed [2+2+2] cycloaddition of enynes **1** with alkynes **2**

3.1 General procedure

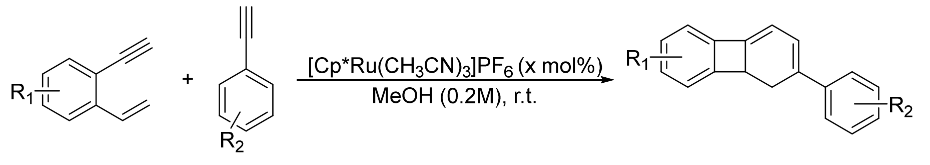

In a 10 mL round-bottomed flask, dry and under argon, were introduced the enyne **1** and the alkyne **2** (4 equiv) in MeOH (0.2 M). Then, [Cp*Ru(CH_3CN)$_3$]PF_6 (0.1 equiv) was added and the resulting solution was stirred at room temperature until disappearance of the starting material (TLC and GC-MS monitoring). The mixture was concentrated under vacuum and the residue was purified by flash column chromatography through silica gel using a mixture of Hex/EtOAc as eluent to afford the corresponding dihydrobiphenylene **3**.

3.2 Synthesis of dihydrobiphenylenes **3a-b**, **3f-i**, **3k**

2-(4-methoxyphenyl)-1,8b-dihydrobiphenylene **3a**[9]

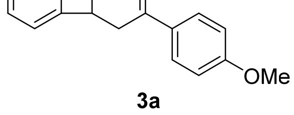

3a

Amounts used: 1-ethynyl-2-vinylbenzene **1a**, (0.052 g, 0.4 mmol), 4-ethynylanisole **2a** (0.065 mL, 0.5 mmol), [Cp*Ru(CH_3CN)$_3$]PF_6 (0.006 mg, 0.012 mmol), MeOH (2 mL).

[9] García-Rubín, S.; González-Rodríguez, C.; García-Yebra, C.; Varela, J. A.; Esteruelas, M. A.; Saá, C. *Angew. Chem. Int. Ed.* **2014**, *53,* 1841-1844

Solvents used: Hex/EtOAc 95/5. Product obtained: dihydrobiphenylene **3a** (92 mg, 87% yield), pale yellow solid.

^{1}H NMR (300 MHz, $CDCl_3$), δ (ppm): 7.49 – 7.42 (m, 2H), 7.34 – 7.24 (m, 4H), 6.97 – 6.89 (m, 2H), 6.48 (dd, J = 5.1, 2.9 Hz, 1H), 6.25 (ddd, J = 5.1, 1.6, 0.9 Hz, 1H), 4.01 (ddd, J = 15.2, 7.0, 1.6 Hz, 1H), 3.86 (s, 3H), 3.16 (dd, J = 15.2, 7.0 Hz, 1H), 2.64 (tdd, J = 15.2, 2.9, 0.9 Hz, 1H).

^{13}C NMR, DEPT (75 MHz, $CDCl_3$), δ (ppm):158.8 (C), 147.0 (C), 142.9 (C), 139.0 (C), 137.2 (C), 134.5 (C), 128.6 (CH), 128.1 (CH), 126.6 (2 x CH), 122.7 (CH), 121.8 (CH), 119.6 (CH), 113.9 (2 x CH), 113.3 (CH), 55.3 (CH_3), 43.3 (CH), 31.4 (CH_2).

Scale-up: **1a** (0.370 g, 2.9 mmol), **2a** (0.450 mL, 3.4 mmol). Dihydrobiphenylene **3a** obtained: 0.52 g, 68% yield.

2-(3,4,5-trimethoxyphenyl)-1,8b-dihydrobiphenylene **3b**

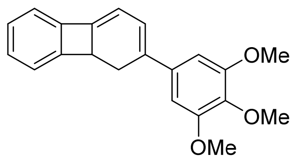

3b

Amounts used: 1-ethynyl-2-vinylbenzene **1a** (0.039 g, 0.3 mmol), 5-ethynyl-1,2,3-trimethoxybenzene **2b** (0.292 mg, 1.5 mmol), [Cp*Ru(CH_3CN)$_3$]PF_6 (0.015 mg, 0.03 mmol), MeOH (1.5 mL). Solvents used: gradient Hex/EtOAc 95/5 to 90/10. Product obtained: dihydrobiphenylene **3b** (55 mg, 56% yield), pale green oil.

^{1}H NMR (300 MHz, $CDCl_3$), δ (ppm): 7.35 – 7.21 (m, 4H), 6.69 (s, 2H), 6.48 (dd, J = 5.1, 2.9 Hz, 1H), 6.21 (ddd, J = 5.1, 1.7, 0.9 Hz, 1H), 3.99 (ddd, J = 15.2, 7.0, 1.7 Hz, 1H), 3.91 (s, 6H), 3.87 (s, 3H), 3.12 (dd, J = 15.2, 6.9 Hz, 1H), 2.64 (tdd, J = 15.2, 3.0, 0.9 Hz, 1H).

^{13}C NMR, DEPT (75 MHz, $CDCl_3$), δ (ppm): 152.7 (C), 146.9 (C), 142.9 (C), 139.8 (C), 138.1 (C), 137.9 (C), 137.6 (C), 129.0 (CH), 127.7 (CH), 123.3 (CH), 122.8 (CH), 119.8 (CH), 113.0 (CH), 109.4 (CH), 102.8 (2xCH), 61.1 (CH_3), 56.2 (2xCH_3), 43.4 (CH), 31.8 (CH_2).

HRMS (APCI) calculated for $C_{21}H_{21}O_3$ $[M+H]^+$: 321.1485, found 321.1487

2-([1,1'-biphenyl]-4-yl)-1,8b-dihydrobiphenylene **3f**

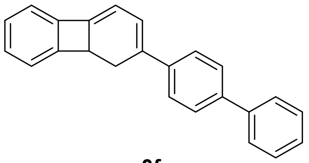

3f

Amounts used: 1-ethynyl-2-vinylbenzene **1a** (0.025 g, 0.19 mmol), 4-ethynyl-1,1'-biphenyl **2f** (0.094 g, 0.5 mmol), [Cp*Ru(CH_3CN)$_3$]PF_6 (0.01 g, 0.02 mmol), MeOH (1 mL). Solvents used: gradient Hex/EtOAc 95/5. Product obtained: dihydrobiphenylene **3f** (26 mg, 42% yield), yellow solid.

^{1}H NMR (300 MHz, $CDCl_3$), δ (ppm): 7.75 – 7.53 (m, 6H), 7.53 – 7.43 (m, 2H), 7.43 – 7.35 (m, 1H), 7.35 – 7.26 (m, 4H), 6.74 – 6.52 (m, 1H), 6.27 (d, *J* = 5.0 Hz, 1H), 4.04 (dd, *J* = 15.2, 7.0 Hz, 1H), 3.23 (ddd, *J* = 15.2, 7.0, 2.2 Hz, 1H), 2.70 (dd, *J* = 17.1, 14.0 Hz, 1H).

^{13}C NMR, DEPT (75 MHz, $CDCl_3$), δ (ppm): 146.0 (C), 141.8 (C), 139.7 (C), 139.6 (C), 138.7 (C), 137.8 (C), 137.1 (C), 127.8 (CH), 127.8 (2xCH), 127.1 (CH), 126.2 (CH), 126.1 (2xCH), 125.9 (2xCH), 124.7 (2xCH), 122.4 (CH), 121.7 (CH), 118.7 (CH), 112.1 (CH), 42.3 (CH), 30.2 (CH_2).

HRMS (APCI) calculated for $C_{24}H_{19}$ $[M+H]^+$: 307.1481, found 307.1485

6-(4-methoxyphenyl)-4b,5-dihydrobiphenyleno[2,3-d][1,3]dioxole **3g**

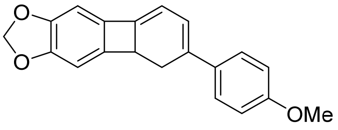

3g

Amounts used: 5-ethynyl-6-vinylbenzo[*d*][1,3]dioxole **1b** (0.052 g, 0.3 mmol), 1-ethynyl-4-methoxybenzene **2a** (0.150 mL, 1.2 mmol), [Cp*Ru(CH_3CN)$_3$]PF_6 (0.015 g, 0.03 mmol), MeOH (1.5 mL). Solvents used: gradient Hex/EtOAc 90/10. Product obtained: dihydrobiphenylene **3f** (0.074 g, 80% yield), yellow solid.

^{1}H NMR (500 MHz, $CDCl_3$), δ (ppm): 7.43 – 7.40 (m, 2H), 6.91 (d, *J* = 8.8 Hz, 2H), 6.81 (t, *J* = 0.8 Hz, 1H), 6.77 (t, *J* = 0.8 Hz, 1H), 6.44 (dd, *J* = 5.0, 2.9 Hz, 1H), 6.05 (ddd, *J* = 5.0, 1.5, 0.8 Hz, 1H), 5.97 (d, *J* = 1.5 Hz, 1H), 5.95 (d, *J* = 1.5 Hz, 1H), 3.85 (s, 3H), 3.83 – 3.78 (m, 1H), 3.10 (dd, *J* = 15.0, 6.6 Hz, 1H), 2.51 (tdd, *J* = 15.0, 2.9, 1.0 Hz, 1H).

^{13}C NMR, DEPT (126 MHz, $CDCl_3$), δ (ppm): 158.6 (C), 148.5 (C), 148.1 (C), 141.0 (C), 138.1 (C), 135.9 (C), 135.8 (C), 134.6 (C), 126.5 (2 x CH), 121.9 (CH), 113.8 (2 x CH), 110.3 (CH), 104.9 (CH), 101.5 (CH), 100.6 (CH_2), 55.3 (CH_3), 41.6 (CH), 31.7 (CH_2).

HRMS (APCI) calculated for $C_{20}H_{17}O_3$ $[M+H]^+$: 305.1172, found 305.1169

6-phenyl-4b,5-dihydrobiphenyleno[2,3-d][1,3]dioxole **3h**

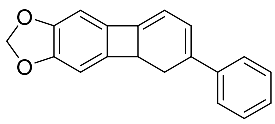

3h

Amounts used: 5-ethynyl-6-vinylbenzo[*d*][1,3]dioxole **1b** (0.050 g, 0.29 mmol), phenylacetylene **2e** (0.130 mL, 1.1 mmol), [Cp*Ru(CH_3CN)$_3$]PF_6(0.015 g, 0.029 mmol), MeOH (1.5 mL). Solvents used: gradient Hex/EtOAc 90/10. Product obtained: dihydrobiphenylene **3h** (0.024 g, 30% yield), pale yellow solid.

^{1}H NMR (300 MHz, $CDCl_3$), δ (ppm): 7.47 (d, J = 7.3 Hz, 2H), 7.36 (t, J = 7.5 Hz, 2H), 7.26 (d, J = 7.9 Hz, 1H), 6.79 (d, J = 10.8 Hz, 2H), 6.53 (dd, J = 5.1, 23.0 Hz, 1H), 6.06 (d, J = 5.1 Hz, 1H), 6.04 – 5.89 (m, 2H), 3.83 (dd, J = 14.9, 6.6 Hz, 1H), 3.13 (dd, J = 14.9, 6.6 Hz, 1H), 2.55 (td, J = 14.9, 3.0 Hz, 1H).

^{13}C NMR, DEPT (75 MHz, $CDCl_3$), δ (ppm): 148.7 (C), 148.2 (C), 141.8 (C), 141.1 (C), 138.5 (C), 136.6 (C), 135.8 (C), 128.4 (2 x CH), 126.8 (CH), 125.3 (2 x CH), 123.6 (CH), 110.1 (CH), 105.0 (CH), 101.5 (CH), 100.7 (CH_2), 41.6 (CH), 31.7 (CH_2).

HRMS (APCI) calculated for $C_{19}H_{15}O_2$ $[M+H]^+$: 275.1067, found 275.1068

6-(thiophen-3-yl)-4b,5-dihydrobiphenyleno[2,3-d][1,3]dioxole **3i**

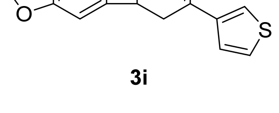

3i

Amounts used: 5-ethynyl-6-vinylbenzo[*d*][1,3]dioxole **1b** (0.049 g, 0.28 mmol), 3-ethynylthiophene **2d** (0.110 mL, 1.1 mmol), [Cp*Ru(CH_3CN)$_3$]PF_6 (0.014 g, 0.028 mmol), MeOH (1.4 mL). Solvents used: gradient Hex/EtOAc 90/10. Product obtained: dihydrobiphenylene **3i** (0.030 g, 38% yield), yellow solid.

^{1}H NMR (300 MHz, $CDCl_3$), δ (ppm): 7.31 (t, *J* = 1.7 Hz, 2H), 7.23 (s, 2H), 6.78 (d, *J* = 14.6 Hz, 1H), 6.56 (d, *J* = 5.5 Hz, 1H), 6.03 (d, *J* = 5.5 Hz, 1H), 5.96 (dd, *J* = 6.9, 1.7 Hz, 2H), 3.86 – 3.76 (m, 1H), 3.12 (dd, *J* = 15.0, 6.5 Hz, 1H), 2.47 (t, *J* = 15.0 Hz, 1H).

^{13}C NMR, DEPT (75 MHz, $CDCl_3$), δ (ppm): 147.6 (C), 147.1 (C), 142.3 (C), 139.9 (C), 135.4 (C), 134.9 (C), 132.4 (C), 124.7 (CH), 124.0 (CH), 121.2 (CH), 118.2 (CH), 108.9 (CH), 103.9 (CH), 100.4 (CH), 99.6 (CH_2), 40.6 (CH), 30.4 (CH_2).

HRMS (APCI) calculated for $C_{17}H_{13}O_2S$ $[M+H]^+$: 281.0631, found 281.0635

6-ethynyl-2-(4-methoxyphenyl)-1,8b-dihydrobiphenylene **3k**

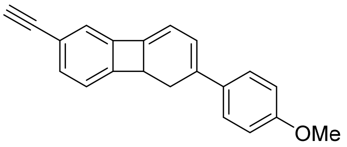

3k

Amounts used: 2,4-diethynyl-1-vinylbenzene **1d** (0.050 g, 0.32 mmol), 4-ethynylanisole **2a** (0.21 mL, 1.64 mmol), $[Cp^*Ru(CH_3CN)_3]PF_6$(0.016 g, 0.033 mmol), MeOH (1.6 mL). Solvents used: gradient Hex/EtOAc 95/05. Product obtained: dihydrobiphenylene **3k** (0.044 g, 47% yield), yellow solid.

^{1}H NMR (300 MHz, $CDCl_3$), δ (ppm): 7.41 (dd, *J* = 8.9, 3.0 Hz, 3H), 7.36 (s, 1H), 7.21 (d, *J* = 7.5 Hz, 1H), 6.95 – 6.84 (m, 2H), 6.44 (dd, *J* = 5.1, 2.9 Hz, 1H), 6.24 (d, *J* = 4.8 Hz, 1H), 3.96 (dd, *J* = 15.1, 6.9 Hz, 1H), 3.83 (s, 3H), 3.18 – 3.07 (m, 1H), 3.08 (s, 1H), 2.59 (td, *J* = 15.1, 2.9 Hz, 1H).

^{13}C NMR, DEPT (75 MHz, $CDCl_3$), δ (ppm): 158.9 (C), 147.7 (C), 142.8 (C), 139.3 (C), 135.9 (C), 134.2 (C), 132.7 (CH), 127.2 (2xCH), 122.9 (CH), 122.7 (CH), 121.8 (CH), 121.6 (CH), 114.3 (CH), 113.9 (2 x CH), 84.1 (C), 76.8 (CH), 55.3 (CH), 43.3 (CH), 30.6 (CH_2).

HRMS (APCI) calculated for $C_{21}H_{17}O$ $[M+H]^+$: 285.1274, found 285.1270

4 Ru-catalyzed [2+2+2] cycloaddition of enyne **7** and alkyne **2a**

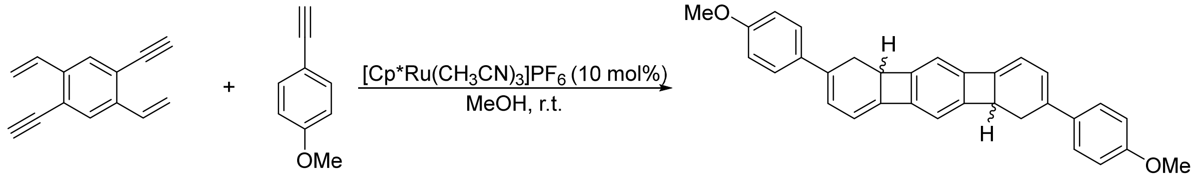

7 **2a** **8**

In a 10 mL round bottomed flask, dry and under Ar, were introduced 1,4-diethynyl-2,5-divinylbenzene **7** (55 mg, 0.31 mmol) and 4-ethynylanisole **2a** (0.12 mL, 0.93 mmol, 3 equiv) in anhydrous MeOH (1.5 mL, 0.2M). After stirring during 5 min, [Cp*Ru(CH_3CN)$_3$]PF_6 (16 mg, 0.031 mmol, 0.1 equiv) was added and the resulting mixture was stirred at room temperature until disappearance of the starting material (TLC monitoring, 24h). Upon completion, the mixture was filtered using Et_2O as eluent to afford the tetrahydrobiphenylene **8** (0.110 mg, 80% yield) as a green-yellow solid.

Scale up: enyne **7** (0. 178g, 1 mmol), alkyne **2a** (3 mmol). Product obtained: tetrahydrobiphenylene **8** (353 mg, 80 % yield).

Melting point: >400ºC

^{1}H NMR (500 MHz, 1,1,2,2-tetrachloroethane-*d*$_2$, 50 ºC) δ (ppm): 6.57 (d, *J* = 8.4 Hz, 2H), 6.29 (s, 1H), 6.03 (d, *J* = 8.4 Hz, 2H), 5.61 (dd, *J* = 5.1, 2.7 Hz, 1H), 5.35 (d, *J* = 5.1 Hz, 1H), 3.04 (dd, *J* = 15.2, 6.8 Hz, 1H), 2.96 (s, 3H), 2.28 (dd, *J* = 15.2, 6.8 Hz, 1H), 1.71 (m, 1H). *Due to the symmetry of the molecule, only half of the peaks are observed.*

^{13}C NMR (126 MHz, 1,1,2,2-tetrachloroethane-*d*$_2$, 50 ºC) δ (ppm): 158.3 (C), 146.4 (C), 142.5 (C), 138.3 (C), 136.6 (C), 133.8 (C), 126.0 (2 x CH), 121.4 (CH), 113.8 (CH), 113.5 (2 x CH), 112.4 (CH), 54.9 (CH_3), 42.1 (CH), 30.8 (CH_2).

HRMS (APCI) calculated for $C_{32}H_{27}O_2$ $[M+H]^+$: 443.2006, found 443.2016

5 Radical ring opening of dihydrobiphenylenes **3** to halogenated benzofused cyclooctatetraenes (bCOT) **4**

5.1 General procedure

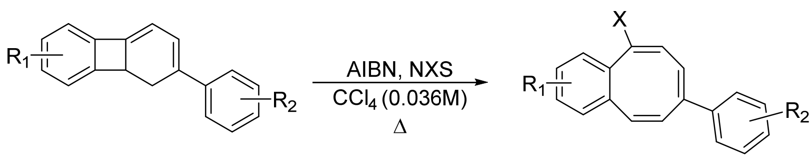

3 **4**

In a round bottomed flask, dry and under Ar, was prepared a solution of dihydrobiphenylene **3** in CCl_4 (0.036 M)[10] and degassed with an Ar balloon for 15 min. Then, AIBN (10 mol%) and NXS (1.1 equiv) were added. The resulting mixture was refluxed in an aluminium heating block until disappearance of the starting material (TLC monitoring, 1h). The crude was concentrated to dryness and the residue was purified by flash column chromatography through silica gel using a mixture of Hex/EtOAc as eluent to afford the corresponding benzofused cyclooctatetraene **4**.

5.2 Synthesis of bCOT's **4a-k**, **4a'** and **4a''**

5-bromo-8-(4-methoxyphenyl)benzo[8]annulene **4a**

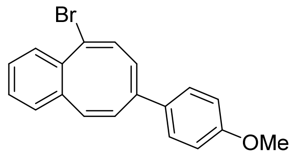

4a

Amounts used: dihydrobiphenylene **3a** (0.072 g, 0.28 mmol), AIBN (0.005 g, 0.03 mmol), NBS (0.056 g, 0.3 mmol), CCl_4 (7.8 mL). Solvents used: gradient Hex/EtOAc 95/05. Product obtained: bCOT **4a** (80 mg, 85% yield), yellow solid.

Melting point: 111.8-112.9 ºC

^{1}H NMR (500 MHz, $CDCl_3$), δ (ppm): δ 7.47 – 7.42 (m, 1H), 7.33 – 7.26 (m, 4H), 7.06 (ddd, J = 6.6, 2.3, 0.8 Hz, 1H), 6.93 (d, J = 11.6 Hz, 1H), 6.85 (d, J = 8.9 Hz, 2H), 6.76 (dd, J = 4.1, 1.3 Hz, 1H), 6.37 (dd, J = 11.6, 1.3 Hz, 1H), 6.06 (d, J = 4.1 Hz, 1H), 3.82 (s, 3H).

^{13}C NMR, DEPT (126 MHz, $CDCl_3$), δ (ppm): 158.5 (C), 140.0 (C), 138.2 (C), 135.7 (C), 132.9 (CH), 132.0 (CH), 131.5 (CH), 130.6 (C), 129.2 (CH), 127.6 (CH), 127.3 (CH), 126.5 (2 x CH), 126.2 (CH), 122.9 (CH), 121.3 (C), 112.7 (2 x CH), 54.3 (CH_3).

HRMS (APCI) calculated for $C_{19}H_{16}BrO$ $[M+H]^+$: 339.0379, found 339.0380

5-bromo-8-(3,4,5-trimethoxyphenyl)benzo[8]annulene **4b**

[10] For the use of others solvents, the procedure and concentration were the same

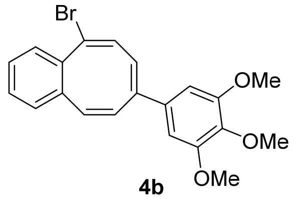

4b

Amounts used: dihydrobiphenylene **3b** (0.050 g, 0.156 mmol), AIBN (0.003 g, 0.016 mmol), NBS (0.030 g, 0.17 mmol), CCl_4 (4.5 mL). Solvents used: gradient Hex/EtOAc 95/05. Product obtained: bCOT **4b** (45 mg, 72% yield), yellow solid.

Melting point: 112.9-114.4 ºC

^{1}H NMR (300 MHz, $CDCl_3$), δ (ppm): 7.49 – 7.30 (m, 1H), 7.29 – 7.18 (m, 2H), 7.07 – 6.98 (m, 1H), 6.90 (d, J = 11.6 Hz, 1H), 6.71 (dd, J = 4.0, 1.3 Hz, 1H), 6.52 (s, 2H), 6.37 – 6.27 (m, 1H), 6.05 (d, J = 4.0 Hz, 1H), 3.83 (s, 6H), 3.80 (s, 3H).

^{13}C NMR, DEPT (75 MHz, $CDCl_3$), δ (ppm): 153.1 (2 x C), 141.5 (C), 139.1 (C), 138.3 (C), 136.5 (C), 134.8 (C), 133.6 (CH), 133.3 (CH), 132.2 (CH), 130.3 (CH), 128.5 (CH), 128.4 (CH), 127.3 (CH), 125.3 (CH), 122.7 (C), 103.8 (2 x CH), 60.9 (CH_3), 56.2 (2 x CH_3).

HRMS (APCI) calculated for $C_{21}H_{20}BrO_3$ $[M+H]^+$: 399.0590, found 399.0576

5-bromo-8-(6-methoxynaphthalen-2-yl)benzo[8]annulene **4c**

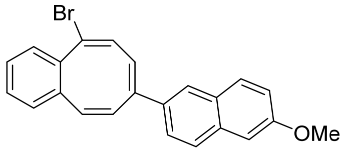

4c

Amounts used: dihydrobiphenylene **3c** (0.044 g, 0.142 mmol), AIBN (0.003 g, 0.016 mmol), NBS (0.028 g, 0.16 mmol), CCl_4 (4 mL). Solvents used: gradient Hex/EtOAc 95/05. Product obtained: bCOT **4c** (34 mg, 62% yield), yellow solid.

Melting point: 140.8-141.9ºC

^{1}H NMR (500 MHz, $CDCl_3$), δ (ppm): 7.73 (d, J = 8.9 Hz, 1H), 7.69 (d, J = 1.9 Hz, 1H), 7.67 (d, J = 8.7 Hz, 1H), 7.52 – 7.44 (m, 2H), 7.34 – 7.25 (m, 2H), 7.18 – 7.13 (m, 1H), 7.13 – 7.09 (m, 2H), 7.00 (d, J = 11.6 Hz, 1H), 6.86 – 6.78 (m, 1H), 6.49 (dd, J = 11.6, 1.4 Hz, 1H), 6.26 (d, J = 4.1 Hz, 1H), 3.93 (s, 3H).

^{13}C NMR, DEPT (126 MHz, $CDCl_3$), δ (ppm): 158.0 (C), 141.5 (C), 139.2 (C), 136.8 (C), 134.2 (C), 134.2 (C), 134.0 (CH), 133.3 (CH), 132.5 (CH), 130.3 (CH), 129.8 (CH), 128.7 (C), 128.7 (CH), 128.4 (CH), 127.3 (CH), 126.9 (CH), 125.5 (CH), 125.2 (CH), 124.5 (CH), 122.6 (C), 119.1 (CH), 105.6 (CH), 55.3 (CH_3).

HRMS (APCI) calculated for $C_{23}H_{18}BrO$ $[M+H]^+$: 389.0536, found 389.0532

3-(10-bromobenzo[8]annulen-7-yl)thiophene **4d**

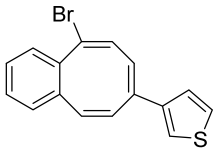

4d

Amounts used: dihydrobiphenylene **3d** (0.072 g, 0.3 mmol), AIBN (0.006 g, 0.034 mmol), NBS (0.060 g, 0.33 mmol), CCl_4 (8.5 mL). Solvents used: gradient Hex/EtOAc 95/05. Product obtained: bCOT **4d** (53 mg, 55% yield), yellow solid.

Melting point: 86.0-86.9 ºC

^{1}H NMR (500 MHz, $CDCl_3$), δ (ppm): 7.51 – 7.36 (m, 1H), 7.32 – 7.24 (m, 3H), 7.19 – 7.16 (m, 2H), 7.09 – 6.99 (m, 1H), 6.91 (d, J = 11.6 Hz, 1H), 6.77 (dd, J = 4.2, 1.3 Hz, 1H), 6.44 (dd, J = 11.6, 1.3 Hz, 1H), 6.15 (d, J = 4.2 Hz, 1H).

^{13}C NMR, DEPT (126 MHz, $CDCl_3$), δ (ppm): 141.0 (C), 139.0 (C), 136.6 (C), 136.5 (C), 133.5 (CH), 133.1 (CH), 131.7 (CH), 130.3 (CH), 128.7 (CH), 128.4 (CH), 127.3 (CH), 125.9 (CH), 125.0 (CH), 124.5 (CH), 122.7 (C), 121.9 (CH).

HRMS (APCI) calculated for $C_{16}H_{12}BrS$ $[M+H]^+$: 314.9838, found 314.9835

5-bromo-8-phenylbenzo[8]annulene **4e**

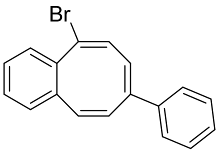

4e

Amounts used: dihydrobiphenylene **3e** (0.068 g, 0.29 mmol), AIBN (0.006 g, 0.034 mmol), NBS (0.058 g, 0.32 mmol), CCl_4 (8.3 mL). Solvents used: gradient Hex/EtOAc 98/02. Product obtained: bCOT **4e** (44 mg, 48% yield), yellow solid.

^{1}H NMR (500 MHz, $CDCl_3$), δ (ppm): 7.50 – 7.35 (m, 1H), 7.33 (dd, J = 7.6, 1.9 Hz, 1H), 7.26 – 7.22 (m, 2H), 7.22 – 7.19 (m, 2H), 7.19 – 7.15 (m, 2H), 6.95 (dd, J = 7.0, 1.9 Hz, 1H), 6.83 (d, J = 11.6 Hz, 1H), 6.65 (dd, J = 4.1, 1.3 Hz, 1H), 6.26 (dd, J = 11.6, 1.3 Hz, 1H), 6.03 (d, J = 4.0 Hz, 1H).

^{13}C NMR, DEPT (126 MHz, $CDCl_3$), δ (ppm): 141.8 (C), 139.3 (C), 139.2 (C), 136.8 (C), 133.9 (CH), 133.4 (CH), 132.5 (CH), 130.4 (CH), 128.8 (CH), 128.5 (3 x CH), 128.1 (CH), 127.4 (CH), 126.5 (2 x CH), 125.7 (CH), 122.8 (C).

HRMS (APCI) calculated for $C_{18}H_{14}Br$ $[M+H]^+$: 309.0273; found: 309.0271

8-([1,1'-biphenyl]-4-yl)-5-bromobenzo[8]annulene **4f**

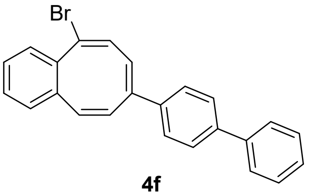

4f

Amounts used: dihydrobiphenylene **3f** (0.031 g, 0.1 mmol), AIBN (0.002 g, 0.011 mmol), NBS (0.020 g, 0.11 mmol), CCl_4 (2.8 mL). Solvents used: gradient Hex/EtOAc 97/03. Product obtained: bCOT **4f** (23 mg, 60% yield), yellow solid.

Melting point: 148.0-149.1ºC

^{1}H NMR (300 MHz, $CDCl_3$), δ (ppm): 7.57 (ddd, J = 11.5, 7.7, 1.8 Hz, 4H), 7.49 – 7.40 (m, 5H), 7.38 (dd, J = 6.7, 1.8 Hz, 1H), 7.32 – 7.26 (m, 2H), 7.13 – 7.05 (m, 1H), 6.97 (d, J = 11.5 Hz, 1H), 6.79 (dd, J = 4.0, 1.3 Hz, 1H), 6.42 (dd, J = 11.6, 1.3 Hz, 1H), 6.20 (d, J = 4.0 Hz, 1H).

^{13}C NMR, DEPT (75 MHz, $CDCl_3$), δ (ppm): 141.2 (C), 140.8 (C), 140.6 (C), 139.2 (C), 138.0 (C), 136.6 (C), 133.8 (CH), 133.4 (CH), 132.3 (CH), 130.3 (CH), 128.8 (2 x CH), 128.6 (CH), 128.4 (CH), 127.4 (CH), 127.3 (CH), 127.1 (2 x CH), 127.0 (2 x CH), 126.7 (2 x CH), 125.6 (CH), 122.7 (C).

HRMS (APCI) calculated for $C_{24}H_{18}Br$ $[M+H]^+$: 385.0586, found 385.0583

5-bromo-8-(4-methoxyphenyl)cycloocta[4,5]venzo[1,2-d][1,3]dioxole **4g**

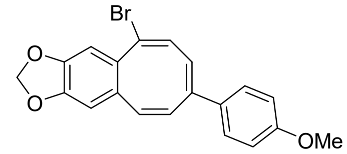

4g

Amounts used: dihydrobiphenylene **3g** (0.046 g, 0.15 mmol), AIBN (0.003 g, 0.017 mmol), NBS (0.029 g, 0.16 mmol), CCl_4 (4.2 mL). Solvents used: gradient Hex/EtOAc 95/05. Product obtained: bCOT **4g** (35 mg, 61% yield), orange oil.

^{1}H NMR (300 MHz, $CDCl_3$), δ (ppm): 7.34 – 7.19 (m, 2H), 6.86 – 6.79 (m, 3H), 6.76 (d, *J* = 11.3 Hz, 1H), 6.66 (dd, *J* = 3.9, 1.0 Hz, 1H), 6.47 (s, 1H), 6.28 (d, *J* = 10.7 Hz, 1H), 6.04 (d, *J* = 3.9 Hz, 1H), 5.93 (dd, *J* = 11.3, 1.3 Hz, 2H), 3.79 (s, 3H).

^{13}C NMR, DEPT (75 MHz, $CDCl_3$), δ (ppm): 159.6 (C), 148.2 (C), 147.1 (C), 141.2 (C), 133.8 (CH), 132.8 (CH), 132.6 (C), 132.4 (CH), 131.5 (C), 130.9 (C), 127.5 (2 x CH), 124.1 (CH), 122.0 (C), 113.8 (2 x CH), 109.7 (CH), 107.8 (CH), 101.5 (CH_2), 55.3 (CH_3).

HRMS (APCI) calculated for $C_{20}H_{16}BrO_3$ $[M+H]^+$: 383.0277, found 383.0275

5-bromo-8-phenylcycloocta[4,5]benzo[1,2-d][1,3]dioxole **4h**

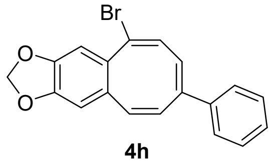

4h

Amounts used: dihydrobiphenylene **3h** (0.058 g, 0.21 mmol), AIBN (0.004 g, 0.023 mmol), NBS (0.041 g, 0.23 mmol), CCl_4 (5.9 mL). Solvents used: gradient Hex/EtOAc 95/05. Product obtained: bCOT **4h** (29 mg, 38% yield), yellow solid.

Melting point: 102.3-103.4ºC

^{1}H NMR (300 MHz, $CDCl_3$), δ (ppm): 7.36-7.27 (m, 5H), 6.87 (s, 1H), 6.80 (d, *J* = 11.5 Hz, 1H), 6.70 (dd, *J* = 3.9, 1.3 Hz, 1H), 6.50 (s, 1H), 6.33 (dd, *J* = 11.5, 1.3 Hz, 1H), 6.15 (d, *J* = 3.9 Hz, 1H), 5.96 (dd, *J* = 11.0, 1.3 Hz, 2H).

^{13}C NMR, DEPT (126 MHz, $CDCl_3$), δ (ppm): 148.2 (C), 147.2 (C), 141.8 (C), 139.0 (C), 133.6 (CH), 133.0 (CH), 132.5 (C), 132.3 (CH), 130.8 (C), 128.4 (2 x CH), 128.0 (CH), 126.3 (2 x CH), 125.7 (CH), 122.4 (C), 109.7 (CH), 107.8 (CH), 101.5 (CH_2).

HRMS (APCI) calculated for $C_{19}H_{14}BrO_2$ $[M+H]^+$: 353.0172, found 353.0174

5-bromo-8-(thiophen-3-yl)cycloocta[4,5]benzo[1,2-d][1,3]dioxole **4i**

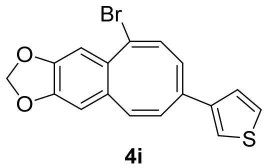

4i

Amounts used: dihydrobiphenylene **3i** (0.028 g, 0.1 mmol), AIBN (0.002 g, 0.011 mmol), NBS (0.020 g, 0.1 mmol), CCl_4 (2.8 mL). Solvents used: gradient Hex/EtOAc 98/02. Product obtained: bCOT **4i** (30 mg, 84% yield), yellow solid.

^{1}H NMR (500 MHz, $CDCl_3$), δ (ppm): 7.24 (dd, *J* = 5.1, 3.0 Hz, 1H), 7.15 (dd, *J* = 5.1, 1.3 Hz, 1H), 7.13 (dd, *J* = 2.9, 1.3 Hz, 1H), 6.84 (s, 1H), 6.74 (d, *J* = 11.3 Hz, 1H), 6.67 (dd, *J* = 4.0, 1.1 Hz, 1H), 6.44 (s, 1H), 6.37 (dd, *J* = 11.3, 1.1 Hz, 1H), 6.13 (d, *J* = 4.0 Hz, 1H), 5.95 (d, *J* = 1.4 Hz, 1H), 5.91 (d, *J* = 1.4 Hz, 1H).

^{13}C NMR, DEPT (126 MHz, $CDCl_3$), δ (ppm):148.2 (C), 147.2 (C), 140.9 (C), 136.7 (C), 133.3 (CH), 132.8 (CH), 132.4 (C), 131.6 (CH), 130.7 (C), 126.0 (CH), 124.9 (CH), 124.7 (CH), 122.3 (C), 121.9 (CH), 109.7 (CH), 107.8 (C), 101.5 (CH_2).

HRMS (APCI) calculated for $C_{17}H_{12}BrO_2S$ $[M+H]^+$: 358.9736, found 358.9734

5-bromo-2-methoxy-8-(4-methoxy-2-vinylphenyl)benzo[8]annulene **4j**

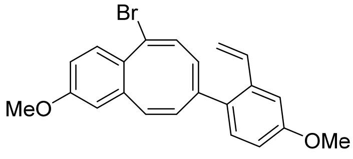

4j

Amounts used: dihydrobiphenylene **3j** (0.038 g, 0.12 mmol), AIBN (0.003 g, 0.013 mmol), NBS (0.024 g, 0.13 mmol), CCl_4 (3.3 mL). Solvents used: gradient Hex/EtOAc 90/10. Product obtained: bCOT **4j** (31 mg, 63% yield), amorphous orange solid.

^{1}H NMR (500 MHz, $CDCl_3$), δ (ppm): 7.37 (d, *J* = 8.8 Hz, 1H), 7.01 – 6.96 (m, 2H), 6.88 (ddd, *J* = 8.7, 2.7, 0.5 Hz, 1H), 6.75 (dd, *J* = 8.5, 2.7 Hz, 1H), 6.68 – 6.63 (m, 2H), 6.56 – 6.49 (m, 2H), 6.16 – 6.10 (m, 1H), 5.65 (d, *J* = 3.9 Hz, 1H), 5.56 (dd, *J* = 17.4, 1.3 Hz, 1H), 5.09 (dd, *J* = 10.9, 1.2 Hz, 1H), 3.82 (s, 3H), 3.80 (s, 3H).

^{13}C NMR, DEPT (126 MHz, $CDCl_3$), δ (ppm):159.6 (C), 159.2 (C), 142.2 (C), 138.5 (C), 137.3 (C), 135.7 (C), 134.3 (C), 133.0 (CH), 132.4 (C), 131.9 (CH), 131.8 (C),

131.6 (CH), 130.2 (CH), 129.7 (CH), 122.9 (C), 114.4 (CH_2), 114.0 (CH), 113.4 (CH), 112.8 (CH), 110.5 (CH), 55.4 (CH_3), 55.3 (CH_3).

HRMS (APCI) calculated for $C_{22}H_{20}BrO_2$ $[M+H]^+$: 395.0641, found 395.0642

10-bromo-2-ethynyl-7-(4-methoxyphenyl)benzo[8]annulene **4k**

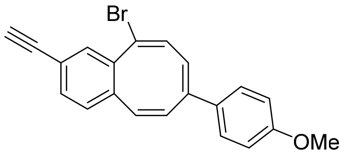

4k

Amounts used: dihydrobiphenylene **3k** (0.080 g, 0.28 mmol), AIBN (0.005 g, 0.028 mmol), NBS (0.055 g, 0.31 mmol), CCl_4 (7.5 mL). Solvents used: gradient Hex/EtOAc 95/05. Product obtained: bCOT **4k** (65 mg, 63% yield), yellow solid.

Melting point: 104.0-105.0 ºC

^{1}H NMR (300 MHz, $CDCl_3$), δ (ppm): 7.54 (s, 1H), 7.34 (d, J = 8.3 Hz, 1H), 7.24 (d, J = 8.6 Hz, 2H), 6.98 (d, J = 6.8 Hz, 1H), 6.89 – 6.77 (m, 3H), 6.73 (d, J = 4.0 Hz, 1H), 6.34 (d, J = 11.8 Hz, 1H), 6.05 – 5.99 (m, 1H), 3.77 (s, 3H), 3.08 (s, 1H).

^{13}C NMR, DEPT (75 MHz, $CDCl_3$), δ (ppm): 159.7 (C), 141.1 (C), 139.6 (C), 137.6 (C), 134.7 (CH), 134.1 (CH), 133.3 (CH), 132.4 (CH), 131.8 (CH), 131.5 (C), 128.8 (CH), 127.6 (2 x CH), 123.5 (CH), 121.3 (C), 121.0 (C), 113.9 (2 x CH), 83.00 (C), 78.1 (CH), 55.4 (CH_3).

HRMS (APCI) calculated for $C_{21}H_{16}BrO$ $[M+H]^+$: 363.0379 , found 363.0380

5-iodo-8-(4-methoxyphenyl)benzo[8]annulene **4a'**

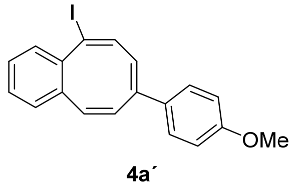

4a´

Amounts used: dihydrobiphenylene **3a** (0.075 g, 0.29 mmol), AIBN (0.003 g, 0.020 mmol), NIS (0.073 g, 0.32 mmol), CCl_4 (8.5 mL). Solvents used: gradient Hex/EtOAc 95/05. Product obtained: bCOT **4a'** (85 mg, 77% yield), yellow solid.

Melting point: 114.8-115.6ºC

^{1}H NMR (500 MHz, $CDCl_3$), δ (ppm): 7.43 (dd, *J* = 7.6, 1.4 Hz, 1H), 7.32 – 7.27 (m, 3H), 7.22 (td, *J* = 7.6, 1.4 Hz, 1H), 7.04 – 6.99 (m, 2H), 6.92 (d, *J* = 11.6 Hz, 1H), 6.85 (d, *J* = 8.9 Hz, 2H), 6.34 (dd, *J* = 11.6, 1.4 Hz, 1H), 6.04 (d, *J* = 3.9 Hz, 1H), 3.81 (s, 3H).

^{13}C NMR, DEPT (126 MHz, $CDCl_3$), δ (ppm): 159.6 (C), 142.6 (CH), 142.0 (C), 141.1 (C), 135.7 (C), 133.1 (CH), 132.6 (CH), 131.6 (C), 131.0 (CH), 128.5 (CH), 128.0 (CH), 127.5 (2 x CH), 127.1 (CH), 125.6 (CH), 113.8 (2 x CH), 98.0 (C), 55.3 (CH_3).

HRMS (APCI) calculated for $C_{19}H_{16}IO$ $[M+H]^+$: 387.0240, found 387.0241

5-chloro-8-(4-methoxyphenyl)benzo[8]annulene **4a''**

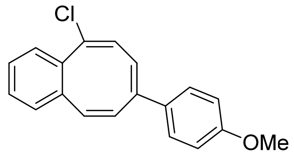

4a´´

Amounts used: dihydrobiphenylene **3a** (0.051 g, 0.20 mmol), AIBN (0.003 g, 0.020 mmol), NCS (0.028 g, 0.16 mmol), CCl_4 (5.7 mL). Solvents used: gradient Hex/EtOAc 95/05. Product obtained: bCOT **4a''** (21 mg, 36% yield), yellow solid.

Melting point: 107.0-108.5ºC

^{1}H NMR (500 MHz, $CDCl_3$), δ (ppm): 7.42 (dd, *J* = 5.5, 3.7 Hz, 1H), 7.32 – 7.26 (m, 4H), 7.09 – 7.06 (m, 1H), 6.91 (d, *J* = 11.6 Hz, 1H), 6.86 – 6.82 (m, 2H), 6.53 (dd, *J* = 4.2, 1.4 Hz, 1H), 6.36 (dd, *J* = 11.6, 1.4 Hz, 1H), 6.10 (d, *J* = 4.2 Hz, 1H), 3.81 (s, 3H).

^{13}C NMR, DEPT (126 MHz, $CDCl_3$), δ (ppm): 159.5 (C), 141.1 (C), 138.0 (C), 137.2 (C), 133.1 (CH), 132.5 (CH), 132.4 (C), 131.7 (C), 129.7 (CH), 129.5 (CH), 128.7 (CH), 128.4 (CH), 127.5 (CH), 127.5 (2 x CH), 122.9 (CH), 113.7 (2 x CH), 55.3 (CH_3).

HRMS (APCI) calculated for $C_{19}H_{16}ClO$ $[M+H]^+$: 295.0884, found 295.0884

6 Sequential preparation of halogenated benzofused cyclooctatetraenes **6a-b**

11-bromo-4-phenyl-1,3-dihydrobenzo[4,5]cycloocta[1,2-c]furan **6a**

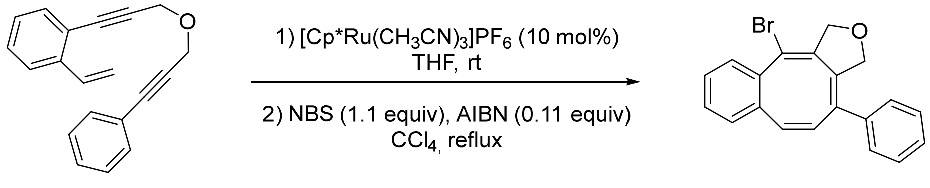

5a **6a**

To a solution of **5a** (0.040 g, 0.15 mmol) in THF (0.73 mL), degassed at -196ºC using liquid N_2, was added [Cp*Ru(CH_3CN)$_3$]PF_6 (0.007 mg, 0.015 mmol). The reaction was stirred at room temperature until disappearance of the starting material (TLC, 25 min). The resulting mixture was filtered through a path of silica using DCM as eluent and evaporated to dryness. The final crude was used in the next step without further purification.

The general procedure was followed using the previous crude, AIBN (0.0027 g, 0.016 mmol), NBS (0.029 g, 0.016 mmol) in CCl_4 (4.1 mL). Upon completion (1h) and work-up, the residue was purified by flash column chromatography through silica gel using a mixture of Hex/EtOAc (97/3) as eluent to give **6a** (15 mg, 30% after two steps) as a yellow oil.

^{1}H NMR (500 MHz, $CDCl_3$), δ (ppm): 7.46 (dd, J = 8.0, 1.3 Hz, 1H), 7.32 – 7.27 (m, 4H), 7.22 (td, J = 7.5, 1.3 Hz, 1H), 7.16 (dd, J = 8.0, 1.6 Hz, 1H), 6.88 (d, J = 8.4 Hz, 1H), 6.68 (d, J = 12.0 Hz, 1H), 6.58 (d, J = 12.0 Hz, 1H), 4.58 (d, J = 13.2 Hz, 1H), 4.42 (d, J = 13.2 Hz, 1H), 4.36 (dd, J = 11.2, 1.2 Hz, 1H), 4.13 (d, J = 11.2 Hz, 1H).

^{13}C NMR, DEPT (126 MHz, $CDCl_3$), δ (ppm): 142.5 (C), 140.2 (C), 139.5 (C), 138.7 (C), 137.7 (C), 136.4 (C), 134.8 (CH), 131.3 (CH), 131.2 (CH), 129.2 (CH), 128.4 (CH), 128.4 (2 x CH), 128.2 (CH), 127.4 (2 x CH), 127.3 (CH), 118.2 (C), 73.3 (CH_2), 72.1 (CH_2).

HRMS (APCI) calculated for $C_{20}H_{16}BrO$ $[M+H]^+$: 351.0379, found 351.0379

11-bromo-4-phenyl-2-tosyl-2,3-dihydro-1H-benzo[4,5]cycloocta[1,2-c]pyrrole **6b**

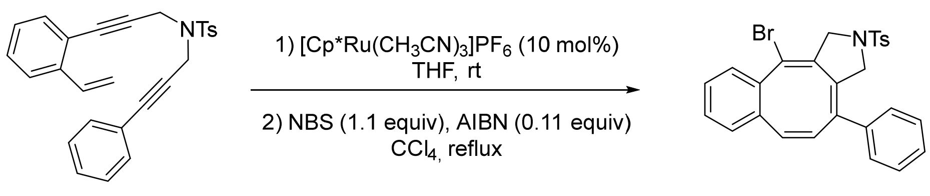

5b **6b**

To a solution of **5b** (0.064 g, 0.15 mmol) in THF (0.75 mL), degassed at -196ºC using liquid N_2, was added [Cp*Ru($CH_3CN)_3$]PF_6 (0.008 mg, 0.015 mmol). The reaction was stirred at room temperature until disappearance of the starting material (TLC, 25 min). The resulting mixture was filtered through a path of silica using DCM as eluent and evaporated to dryness. The final crude was used in the next step without further purification.

The general procedure was followed using the previous crude, AIBN (0.0029 g, 0.018 mmol), NBS (0.031 g, 0.017 mmol) in CCl_4 (4.4 mL). Upon completion (1h) and work-up, the residue was purified by flash column chromatography through silica gel using a mixture of Hex/EtOAc (97/3) as eluent to give an inseparable mixture of **6b** and 4-phenyl-2-tosyl-2,3-dihydro-1H-benzo[3,4]cyclobuta[1,2-*e*]isoindole **6b*** in a 3:1 ratio (33 mg, 40% after two steps) as a yellow oil.

^{1}H NMR (300 MHz, $CDCl_3$), δ (ppm): 7.63 (d, *J* = 8.3 Hz, 2H), 7.33 (dd, *J* = 6.8, 3.0 Hz, 5H), 7.30 – 7.24 (m, 3H), 7.04 (ddd, *J* = 4.7, 2.4, 1.4 Hz, 2H), 6.89 – 6.83 (m, 1H), 6.62 (d, *J* = 11.9 Hz, 1H), 6.35 (dd, *J* = 11.9, 1.2 Hz, 1H), 4.27 (d, *J* = 14.4 Hz, 1H), 4.03 (d, *J* = 14.4 Hz, 1H), 3.86 (d, *J* = 1.2 Hz, 2H), 2.50 (s, 3H). Minor product **6b***: 7.76 (d, *J* = 8.2 Hz, 2H), 7.45 – 7.37 (m, 3H), 7.21 (ddd, *J* = 7.7, 5.1, 3.7 Hz, 4H), 6.79 (dd, *J* = 4.9, 2.9 Hz, 2H), 6.69 – 6.65 (m, 1H), 6.63 (d, *J* = 0.9 Hz, 1H), 6.56 (s, 1H), 4.44 (s, 2H), 4.41 (s, 2H), 2.44 (s, 3H).

^{13}C NMR, DEPT (126 MHz, $CDCl_3$), δ (ppm): 143.8 (C), 139.9 (C), 139.7 (C), 139.4 (C), 139.0 (C), 136.2 (C), 134.5 (CH), 133.9 (C), 133.5 (C), 131.5 (CH), 131.1 (CH), 129.9 (2 x CH), 129.1 (CH), 128.7 (2 x CH), 128.6 (2 x CH), 128.5 (CH), 127.8 (CH), 127.7 (CH), 127.5 (CH), 127.4 (CH), 120.0 (C), 54.9 (CH_2), 52.9 (CH_2), 21.7 (CH_3). Minor product **6b***: 151.2 (C), 150.6 (C), 149.6 (C), 143.9 (C), 143.7 (C), 139.9 (C), 137.0 (C), 135.4 (C), 133.6 (C), 130.0 (2 x CH), 128.9 (C), 128.8 (2 x CH), 128.8 (CH), 127.8 (2 x CH), 126.6 (C), 117.9 (CH), 117.8 (CH), 117.6 (CH), 52.9 (CH_2), 51.2 (CH_2), 21.7 (CH_3).

HRMS (APCI) calculated for $C_{27}H_{23}BrNO_2S$ $[M+H]^+$: 504.0627, found 504.0635

7 Radical ring opening of tetrahydrobiphenylene **8** to linear-benzodiCOT **9**

7.1 Synthesis of linear-benzodiCOT **9**

1,8-dibromo-4,11-bis(4-methoxyphenyl)benzo[1,2:4,5]di[8]annulene **9**

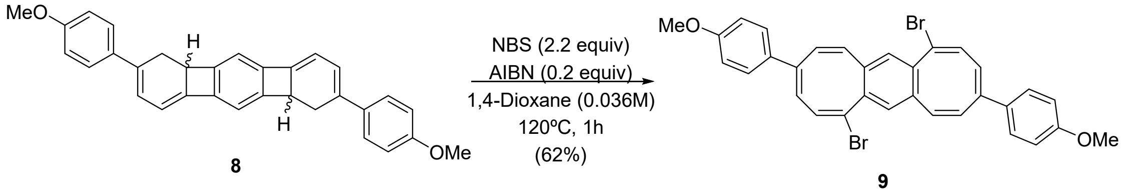

A solution of **8** (0.057 g, 0.13 mmol) in 1,4-dioxane (3.6 mL) was degassed with Ar for 15 min. Then, AIBN (0.004 g, 0.026 mmol) and NBS (0.052 g, 0.28 mmol) were added. The resulting mixture was stirred at reflux in an aluminium heating block until disappearance of the starting material (TLC monitoring, 1h). The crude was concentrated to dryness and the residue was purified by filtration using hot MeOH as eluent to afford bCOT **9** (48 mg, 62 % yield) as a brown-green solid.

Melting point: >240ºC

^{1}H NMR (500 MHz, 1,1,2,2-tetrachloroethane-d_2, 50ºC) δ (ppm): *major S conformer*, 6.56 (d, *J* = 8.8 Hz, 4H), 6.40 (s, 2H), 6.15 (d, *J* = 11.7 Hz, 2H), 6.10 (d, *J* = 8.8 Hz, 4H), 6.03 (dd, *J* = 4.2, 1.3 Hz, 2H), 5.62 (dd, *J* = 11.7, 1.3 Hz, 2H), 5.30 – 5.27 (m, 2H), 3.05 (s, 6H). For *minor U conformer*: 6.59 (d, *J* = 8.9 Hz, 4H), 6.39 (s, 2H), 6.15 – 6.12 (m, 2H), 6.10 (d, *J* = 8.8 Hz, 4H), 6.04 (d, *J* = 1.2 Hz, 2H), 5.62 (dd, *J* = 11.7, 1.2 Hz, 2H), 5.32 (d, *J* = 4.1 Hz, 2H), 3.09 (s, 6H). *Due to the overlapping of both conformers peaks, is not possible a clear assignment of the region from 6.0 to 6.2 ppm.*

^{13}C NMR (126 MHz, 1,1,2,2-tetrachloroethane-d_2, 50ºC) δ (ppm): *major S conformer*, 158.9 (2 x C), 140.0 (2 x C), 138.7 (2 x C), 135.3 (2 x C), 134.4 (2 x CH), 132.4 (2 x CH), 131.9 (2 x CH), 130.8 (2 x C), 130.2 (2 x CH), 127.1 (4 x CH), 123.2 (2 x CH), 120.6 (2 x C), 113.3 (4 x CH), 54.9 (2 x CH_3). *Minor U conformer*: 159.0 (2 x C), 140.5 (2 x C), 138.8 (2 x C), 135.3 (2 x C), 134.0 (2 x CH), 132.3 (2 x CH), 131.7 (2 x CH), 130.8 (2 x C), 130.1 (2 x CH), 127.1 (4 x CH), 123.2 (2 x CH), 120.7 (2 x C), 113.4 (4 x CH), 54.9 (2 x CH_3).

HRMS (APCI) calculated for $C_{32}H_{24}Br_2O_2$ $[M+]^+$: 598.0138, found 598.0136

7.2 Variable-temperature NMR and conformational studies of linear-benzodiCOT **9**

The ^{1}H NMR spectrum (1,1,2,2-tetrachloethane-d_2) of **9** at room temperature (25ºC) showed a 1:2.5 ratio, U-and S-shaped, as judged by the -OMe signals. This relation could be thermally equilibrated to 1:1.5 ratio at 100ºC.

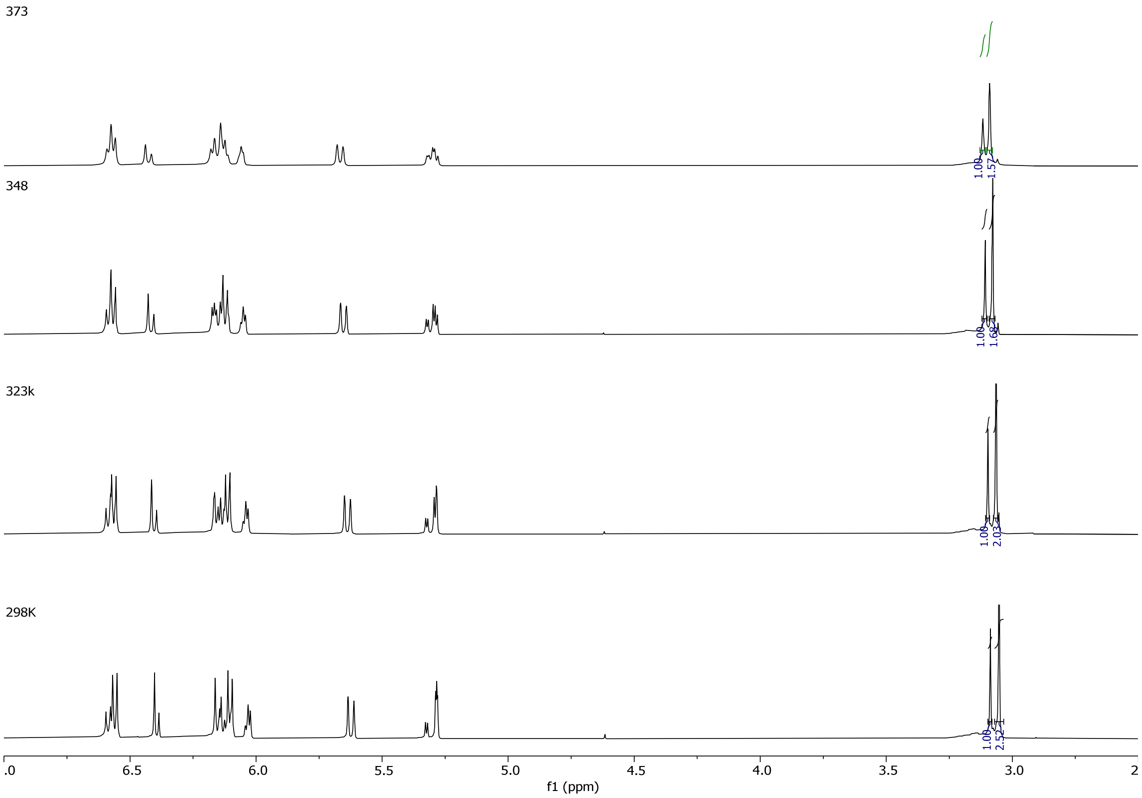

8 Sequential preparation and gram scale synthesis of bCOT **4a**

The general procedure for the Ru-catalyzed [2+2+2] cycloaddition was modified using $[Cp^*Ru(CH_3CN)_3]PF_6$ (0.122 mg, 0.24 mmol), 1-ethynyl-2-vinylbenzene **1a** (1036 mg, 8.1 mmol) and 4-ethynylanisole **2a** (1.3 mL, 9.7 mmol) in MeOH (40 mL). After completion (20 min) the reaction mixture was dissolved in DCM and evaporated to dryness. The final crude was used in the next step without further purification.

A solution of previous crude in DCE (220 mL) was degassed with Ar for 15 min. Then, AIBN (0.132 g, 0.81 mmol) and NBS (1.630 g, 8.8 mmol) were added. The resulting mixture was placed in an aluminium heating block and stirred at reflux for 1.5 h until disappearance of the starting material (TLC monitoring). The crude was concentrated to dryness and the residue was purified by flash column chromatography through silica gel using a mixture of Hex/EtOAc (95/5) as eluent to afford **4a** (2.10 g, 76 %) as a yellow solid.

9 Derivatization of bCOT **4a**

8-(4-methoxyphenyl)-5-phenylbenzo[8]annulene **10**

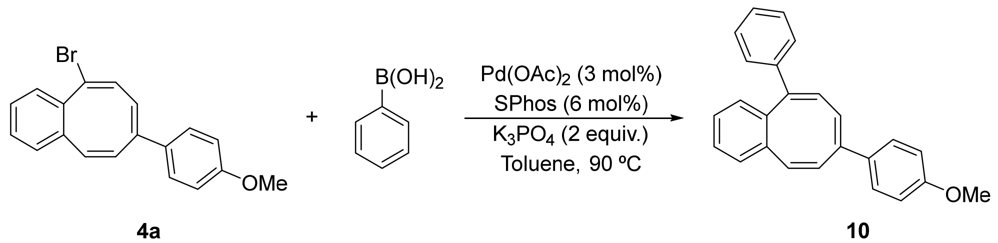

A solution of SPhos (0.008 g, 0.018 mmol), $Pd(OAc)_2$ (0.002 g, 0.009 mmol), K_3PO_4 (0.129 g, 0.59 mmol), phenyboronic acid (0.054 g, 0.442 mmol) and **4a** (0.100 g, 0.295 mmol) in toluene (0.860 mL, 0.5 M) was stirred at 90 ºC in an oil bath. Upon completion (24 h), the reaction mixture was diluted in EtOAc and filtered through silica gel. The crude was purified by flash column chromatography through silica gel using a mixture of Hex/EtOAc (98/2) as eluent to afford the phenylbenzo[8]annulene **10** (70 mg, 71% yield) as a pale yellow solid.

Melting point: 178.3-179.9 ºC

^{1}H NMR (300 MHz, $CDCl_3$), δ (ppm): 7.38 (d, J = 8.6 Hz, 2H), 7.34 – 7.25 (m, 6H), 7.20 (t, J = 7.3 Hz, 2H), 6.98 (t, J = 11.1 Hz, 2H), 6.88 (d, J = 8.6 Hz, 2H), 6.69 (d, J = 3.5 Hz, 1H), 6.40 (dd, J = 7.3, 3.5 Hz, 2H), 3.83 (s, 3H).

^{13}C NMR, DEPT (75 MHz, $CDCl_3$), δ (ppm): 159.3 (C), 144.6 (C), 142.9 (C), 140.6 (C), 140.6 (C), 138.9 (C), 133.3 (CH), 132.7 (CH), 132.4 (C), 130.3 (CH), 128.5 (2 x CH), 128.2 (2 x CH), 127.8 (2 x CH), 127.5 (2 x CH), 127.2 (CH), 127.0 (CH), 126.8(CH), 125.7 (CH), 113.7 (2 x CH), 55.3 (CH_3).

HRMS (APCI) calculated for $C_{25}H_{21}O$ $[M+H]^+$: 337.1587, found 337.1586

((8-(4-methoxyphenyl)benzo[8]annulen-5-yl)ethynyl)trimethylsilane **11a**

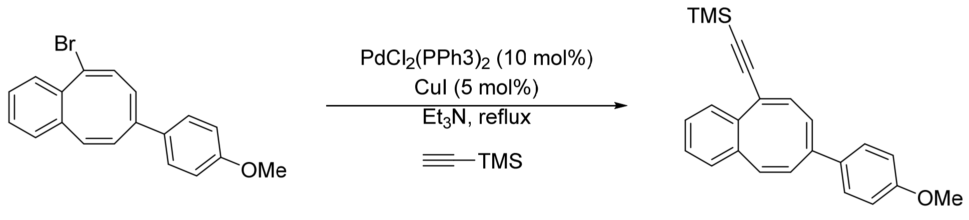

Degassed Et_3N (7 mL, 0.2M) and TMSA (0.485 mL, 3.5 mmol, 2.5 equiv) were added to a mixture of **4a** (0.475 g, 1.4 mmol), $PdCl_2(PPh_3)_2$ (0.098 g, 0.14 mmol) and CuI (0.013 g, 0.07 mmol) in a 25 mL vial flask under argon. The resulting solution was stirred at reflux in an aluminium heating block until disappearance of the starting material (TLC

monitoring, o/n). Upon completion, the mixture was filtered over a path of silica gel and evaporated to dryness. The crude was diluted in EtOAc, washed with an aqueous solution of $NH_4Cl_{(sat)}$ and with brine, dried over anhydrous $MgSO_4$, filtered and concentrated to dryness. The resulting crude was purified by flash column chromatography through silica gel using a mixture of Hex/EtOAc (93/7) as eluent to afford the alkynyl COT **11a**, (0.445 g, 89 % yield) as a yellow solid.

Melting point: 72.3-72.8 ºC

^{1}H NMR (500 MHz, $CDCl_3$), δ (ppm): 7.37 – 7.34 (m, 1H), 7.32 – 7.28 (m, 2H), 7.26 – 7.23 (m, 2H), 7.05 – 7.02 (m, 1H), 6.85 (d, J = 11.6 Hz, 1H), 6.84 – 6.81 (m, 2H), 6.71 (dd, J = 4.5, 1.2 Hz, 1H), 6.30 (dd, J = 11.5, 1.2 Hz, 1H), 6.18 (d, J = 4.5 Hz, 1H), 3.79 (s, 3H), 0.20 (s, 9H).

^{13}C NMR, DEPT (126 MHz, $CDCl_3$), δ (ppm): 159.5 (C), 141.5 (C), 139.3 (CH), 138.0 (C), 137.5 (C), 133.6 (CH), 132.2 (CH), 132.0 (C), 129.4 (CH), 128.8 (CH), 127.5 (CH), 127.5 (2 x CH), 127.1 (CH), 126.8 (C), 123.7 (CH), 113.7 (2 x CH), 107.3 (C), 92.6 (C), 55.3 (CH_3), 0.0 (3 x CH_3).

HRMS (APCI) calculated for $C_{24}H_{25}OSi$ $[M+H]^+$: 357.1669, found 357.1671

5-ethynyl-8-(4-methoxyphenyl)benzo[8]annulene **11a'**

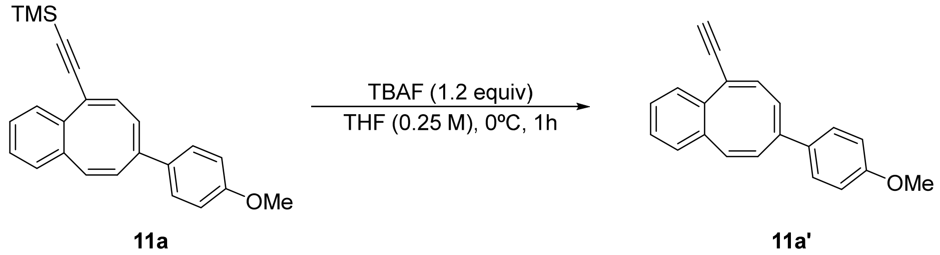

To a solution of **11a** (0.439 g, 1.231 mmol) in THF (4.9 mL) cooled at 0ºC was added TBAF (1M in THF, 1.2 equiv). The resulting mixture was stirred at the same temperature until disappearance of the starting material (TLC monitoring). After 1.5 h the reaction was quenched with water and extracted with Et_2O (x 3). The combined organic layers were dried over anhydrous $MgSO_4$, filtered and evaporated to dryness. The resulting crude was purified by flash column chromatography through silica gel using a mixture of Hex/EtOAc (95/5) as eluent to afford alkynyl COT **11a'** (0.314 g, 89% yield) as a yellow solid.

Melting point: 170.2-171.4 ºC

^{1}H NMR (500 MHz, $CDCl_3$), δ (ppm): 7.36 – 7.33 (m, 1H), 7.29 (d, *J* = 8.8 Hz, 2H), 7.25 – 7.23 (m, 2H), 7.06 – 7.02 (m, 1H), 6.86 (d, *J* = 11.6 Hz, 1H), 6.82 (d, *J* = 8.8 Hz, 2H), 6.74 (d, *J* = 4.4 Hz, 1H), 6.30 (dd, *J* = 11.6, 1.3 Hz, 1H), 6.18 (d, *J* = 4.4 Hz, 1H), 3.79 (s, 3H), 2.95 (s, 1H).

^{13}C NMR, DEPT (126 MHz, $CDCl_3$), δ (ppm): 159.7 (C), 141.8 (C), 139.9 (CH), 137.9 (C), 137.6 (C), 133.6 (CH), 132.3 (CH), 132.0 (C), 129.3 (CH), 129.0 (CH), 127.8 (CH), 127.6 (2 x CH), 127.3 (CH), 125.7 (C), 123.7 (CH), 113.9 (2 x CH), 86.0 (C), 75.9 (CH), 55.5 (CH_3).

HRMS (APCI) calculated for $C_{21}H_{17}O$ $[M+H]^+$: 285.1274; found: 285.1277

8-(4-methoxyphenyl)-5-(8-(4-methoxyphenyl)benzo[8]annulen-5-yl)ethynyl)benzo[8]annulene **11c**

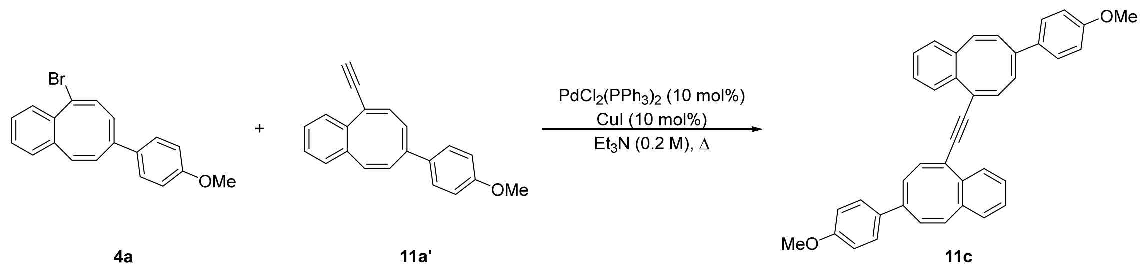

In a double-neck round bottomed flask were introduced $PdCl_2(PPh_3)_2$ (0.035 g, 0.049 mmol), CuI (0.010 g, 0.049 mmol), **4a** (0.200 g, 0.590 mmol) and **11a'** (0.140 g, 0.490 mmol). The flask was evacuated and refilled with Ar three times. Then, Et_3N (2.5 mL, 0.2 M) was added and the reaction stirred at reflux in an aluminium heating block during 4h (TLC monitoring). The reaction mixture was filtered through silica gel and evaporated to dryness. The crude was diluted in EtOAc, washed twice with a solution of NH_4Cl (sat) and brine. The organic layers were dried over anhydrous $MgSO_4$, filtered and evaporated to dryness and the resulting crude was purified by flash column chromatography through silica gel using a mixture of Hex/EtOAc (95/5 to 90/10) as eluent to afford the alkynyl COT **11c** (106 mg, 40% yield) as a yellow solid.

Melting point: 234.8-235.0 ºC

^{1}H NMR (500 MHz, $CDCl_3$), δ (ppm): 7.37 – 7.31 (m, 2H), 7.31 – 7.28 (m, 4H), 7.22 (ddd, *J* = 7.3, 3.5, 1.6 Hz, 4H), 7.06 – 6.99 (m, 2H), 6.85 (dd, *J* = 11.5, 2.5 Hz, 2H), 6.82 (d, *J* = 8.8 Hz, 4H), 6.64 (ddd, *J* = 5.6, 4.5, 1.2 Hz, 2H), 6.30 (ddd, *J* = 11.5, 3.8, 1.2 Hz, 2H), 6.19 (dd, *J* = 4.4, 1.4 Hz, 2H), 3.78 (s, 6H).

^{13}C NMR, DEPT (126 MHz, $CDCl_3$), δ (ppm): 159.5 (2 x C), 141.4 (2 x C), 138.3 (C), 138.3 (C), 138.2 (CH), 138.1 (CH), 137.4 (C), 133.5 (C), 133.5 (C), 132.2 (2 x CH), 132.0 (2 x C), 129.5 (C), 129.4 (C), 128.8 (2 x CH), 128.2 (C), 127.4 (4 x CH), 127.1 (CH), 127.0 (CH), 126.9 (2 x C), 124.0 (2 x C), 123.9 (2 x C), 113.7 (4 x CH), 90.7 (C), 90.6 (C), 55.3 (2 x CH_3).

HRMS (APCI) calculated for $C_{40}H_{31}O_2$ $[M+H]^+$: 543.2319, found: 543.2319

4-((8-(4-methoxyphenyl)benzo[8]annulen-5-yl)ethynyl)-N,N-dimethylaniline **11b**

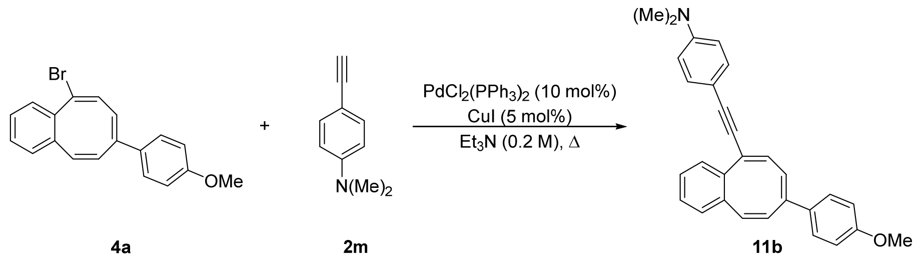

In a double-neck round bottomed flask were introduced $PdCl_2(PPh_3)_2$ (0.050 g, 0.072 mmol), CuI (0.007g, 0.036 mmol), **4a** (0.243 g, 0.716 mmol) and **2m** (0.260 g, 1.791 mmol, 2.5 equiv). Then, Et_3N (3.6 mL, 0.2 M) was added and the reaction was stirred at reflux in an aluminium heating block during 24h (TLC monitoring). The reaction mixture was filtered through silica gel and evaporated to dryness. The crude was diluted in EtOAc, washed twice with a solution of $NH_4Cl_{(sat)}$ and brine and the organic layers were dried over anhydrous $MgSO_4$, filtered and evaporated to dryness. The resulting crude was purified by flash column chromatography through silica gel using a mixture of Hex/EtOAc (95/5 to 8/2) as eluent to afford alkynyl COT **11b** (0.212 g, 73% yield) as a dark green solid.

Melting point: 193.4-194.0 ºC

^{1}H NMR (500 MHz, $CDCl_3$), δ (ppm): 7.46 (dd, J = 6.7, 2.4 Hz, 1H), 7.41 (d, J = 8.4 Hz, 1H), 7.37 – 7.31 (m, 3H), 7.27 (dd, J = 8.4, 4.4 Hz, 2H), 7.10 – 7.05 (m, 1H), 6.91 (d, J = 11.5 Hz, 1H), 6.85 (d, J = 8.7 Hz, 2H), 6.72 – 6.67 (m, 1H), 6.65 – 6.61 (m, 2H), 6.35 (dd, J = 11.5, 1.5 Hz, 1H), 6.26 (d, J = 4.4 Hz, 1H), 3.81 (s, 3H), 2.99 (s, 6H).

^{13}C NMR, DEPT (126 MHz, $CDCl_3$), δ (ppm): 159.4 (C), 150.0 (C), 141.0 (C), 138.8 (C), 137.4 (C), 136.5 (CH), 133.6 (CH), 133.5 (CH), 132.7 (2 x CH), 132.2 (CH), 132.2 (CH), 129.6 (CH), 128.7 (CH), 127.4 (2 x CH), 127.3 (CH), 127.1 (CH), 124.3 (CH), 113.7 (2 x CH), 111.8 (2 x CH), 110.2 (C), 90.1 (C), 89.4 (C), 55.3 (CH_3), 40.2 (2 x CH_3).

HRMS (APCI) calculated for $C_{29}H_{26}NO$ $[M+H]^+$: 404.2009, found: 404.2002

6-(4-methoxyphenyl)-1,2,3,4-tetraphenyldibenzo[a,c][8]annulene **12**

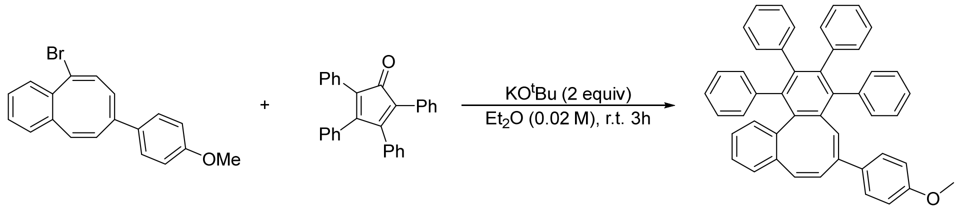

4a **12**

To a solution of tetraphenylcyclopentadienone (0.302 g, 0.77 mmol, 1.1 equiv) and KO^{t}Bu (0.157 g, 1.4 mmol, 2 equiv) in anhydrous Et_2O (35 mL) was added **4a** (0.237 g, 0.7 mmol). The mixture was stirred at rt until disappearance of the starting material (TLC monitoring, 3h). Upon completion, the reaction was quenched with an aqueous solution of NH_4Cl (sat) and washed twice with brine. The organic layers were dried over anhydrous $MgSO_4$, filtered and evaporated to dryness. The resulting crude was purified by flash column chromatography through silica gel using a mixture of Hex/EtOAc (95/5) as eluent to afford benzoCOT **12**, (0.353 g, 82% yield) as a white solid.

Melting point: 246.0-246.4 ºC

^{1}H NMR (500 MHz, $CDCl_3$), δ (ppm): 7.39 – 7.34 (m, 1H), 7.30 (t, J = 7.3 Hz, 1H), 7.23 – 7.17 (m, 2H), 7.13 (t, J = 6.7 Hz, 2H), 7.10 – 7.04 (m, 2H), 7.02 – 6.95 (m, 2H), 6.93 (d, J = 1.9 Hz, 2H), 6.88 – 6.80 (m, 5H), 6.81 – 6.75 (m, 8H), 6.70 - 6.68 (m, 4H), 6.54 (d, J = 5.4 Hz, 1H), 6.35 (d, J = 11.1 Hz, 1H), 3.73 (s, 3H).

^{13}C NMR, DEPT (126 MHz, $CDCl_3$), δ (ppm): 159.2 (C) , 141.5 (C), 140.5 (C), 140.5 (C), 140.4 (C), 140.3 (C), 140.1 (C), 140.0 (C), 139.6 (C), 138.8 (C), 138.5 (C), 137.7 (C), 134.0 (CH), 132.8 (C), 132.3 (CH), 131.9 (CH), 131.8 (CH), 131.7 (CH), 131.5 (CH), 131.2 (CH), 131.0 (CH), 130.9 (CH), 130.7 (CH), 130.0 (CH), 129.1 (CH), 128.1 (2 x CH), 127.8 (CH), 127.3 (CH), 126.8 (2x CH), 126.8 (2 x CH), 126.7 (CH), 126.4 (2 x CH), 126.3 (2 x CH), 126.0 (CH), 125.9 (CH), 125.7 (CH), 125.4 (CH), 125.2 (CH), 125.1 (CH), 113.5 (2 x CH), 55.3 (CH_3).

HRMS (APCI) calculated for $C_{47}H_{35}O$ $[M+H]^+$: 615.2682, found 615.2683

10 X-Ray crystallographic Data

All X-ray structure were collected in a Bruker D8 VENTURE PHOTON-III C14 Diffractometer, using a microfocus sealed tube Incoatec IμS 3.0 multilayer mirror monochromator as radiation source and a detector resolution of 7.3910 pixels mm^{-1}.

Data collection: Bruker APEX3 software; cell refinement: SAINT V8.40A (Bruker Nano, Inc., 2019); data reduction: SAINT V8.40A (Bruker Nano, Inc., 2019); program(s) used to solve structure: SHELXT 2018/2 (Sheldrick, 2015); program(s) used to refine structure: SHELXL2018/3 (Sheldrick, 2018); molecular graphics: ORTEP 2014.1 (Farrugia, 2012); software used to prepare material for publication: IUCr Journals printCIF.

10.1 Crystallographic data for compound **4a**

An X-ray crystal of compound **4a** (CCDC2085994) was grown by vapor diffusion using DCM and hexanes as mixture of solvents.

Figure S1. ORTEP drawing of compound **4a** (CCDC2085994) showing thermal ellipsoids at the 50% contour probability level

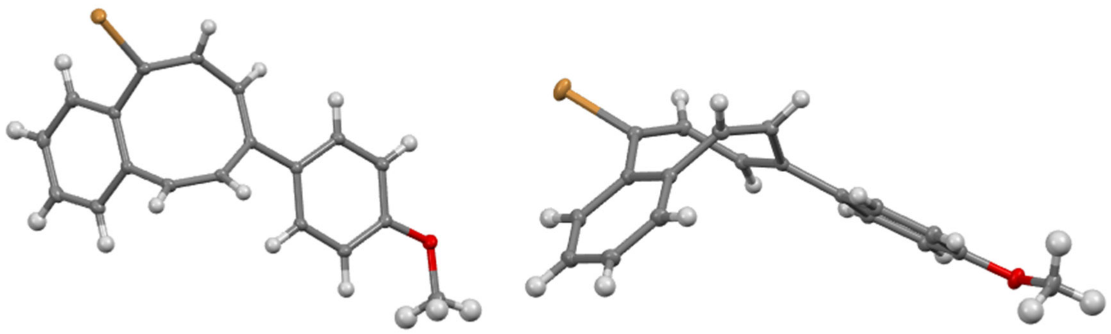

Table S1. Crystal data and structure refinement for **4a**

Deposition Number CCDC	2085994
Chemical formula	$C_{19}H_{15}BrO$
Molecular weight	339.22
Temperature	100 K
Wavelength	0.71073 Å
Crystal size	0.15 × 0.08 × 0.08 mm
Crystal habit	Colorless block
Crystal system	Monoclinic

Space group	$P2_1$
Unit cell dimensions	a = 9.7753 (3) Å
	b = 6.0276 (2) Å β = 97.881 (1)º
	c = 12.8424 (5) Å
Volume	749.55 (4) Å^3
Z	2
Density (calculated)	1.503 g/cm^3
Absortion coefficient	2.74 mm^{-1}
F(000)	344

10.2 Crystallographic data for compound **9**

An X-ray crystal of compound **9** (CCDC2085993) was grown by vapor diffusion using hot $CHCl_3$ and hexane as mixture of solvents.

Figure S2. ORTEP drawing of compound **9** (CCDC2085993) showing thermal ellipsoids at the 50% contour probability level

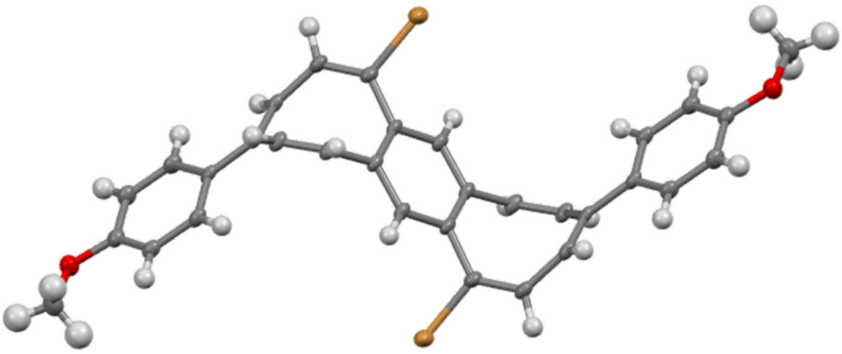

Table S2. Crystal data and structure refinement for **9**

Deposition Number CCDC	CCDC2085993
Chemical formula	$C_{32}H_{24}Br_2O_2$
Molecular weight	600.33
Temperature	100 K
Wavelength	0.71073 Å
Crystal size	0.25 × 0.12 × 0.11 mm
Crystal habit	Block, clear yellow

Crystal system	Monoclinic
Space group	$P2_{1/n}$
Unit cell dimensions	a = 10.1111 (3) Å
	b = 5.9449 (2) Å β = 99.697 (1)°
	c = 21.6066 (7) Å
Volume	1280.21 (7) Å3
Z	2
Density (calculated)	1.557 g/cm^3
Absorption coefficient	3.19 mm^{-1}
F(000)	604

10.3 Crystallographic data for compound **11c**

An X-ray crystal of compound **11c** (CCDC2085992) was grown after solving in hot toluene until reach rt (o/n)

Figure S3. ORTEP drawing of compound **11c** (CCDC2085992) showing thermal ellipsoids at the 50% contour probability level

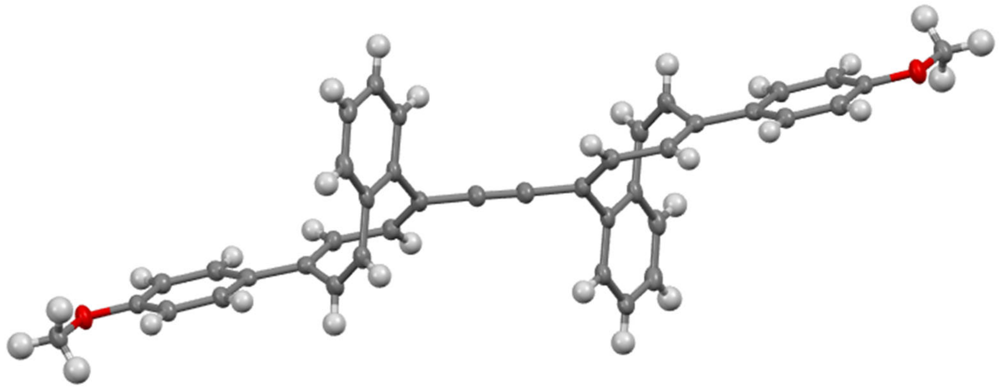

Table S3. Crystal data and structure refinement for **11c**

Deposition Number CCDC	2085992
Chemical formula	$C_{40}H_{30}O_2$
Molecular weight	542.64
Temperature	100 K
Wavelength	1.54178 Å
Crystal size	0.23 × 0.11 × 0.01 mm
Crystal habit	Plate, clear yellow
Crystal system	Monoclinic

Space group	$P2_{1/c}$	
Unit cell dimensions	a = 17.2433 (6)Å	
	b = 6.0650 (2)Å	β = 112.9148 (18)º
	c = 14.3696 (5)Å	
Volume	1384.19 (8)Å^3	
Z	2	
Density (calculated)	1.302 g/cm^3	
Absortion coefficient	0.61 mm^{-1}	
F(000)	572	

11 NMR Spectra

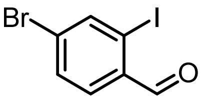

S1

^{1}H-NMR (300 Hz) and ^{13}C-NMR, DEPT (126 Hz) in $CDCl_3$

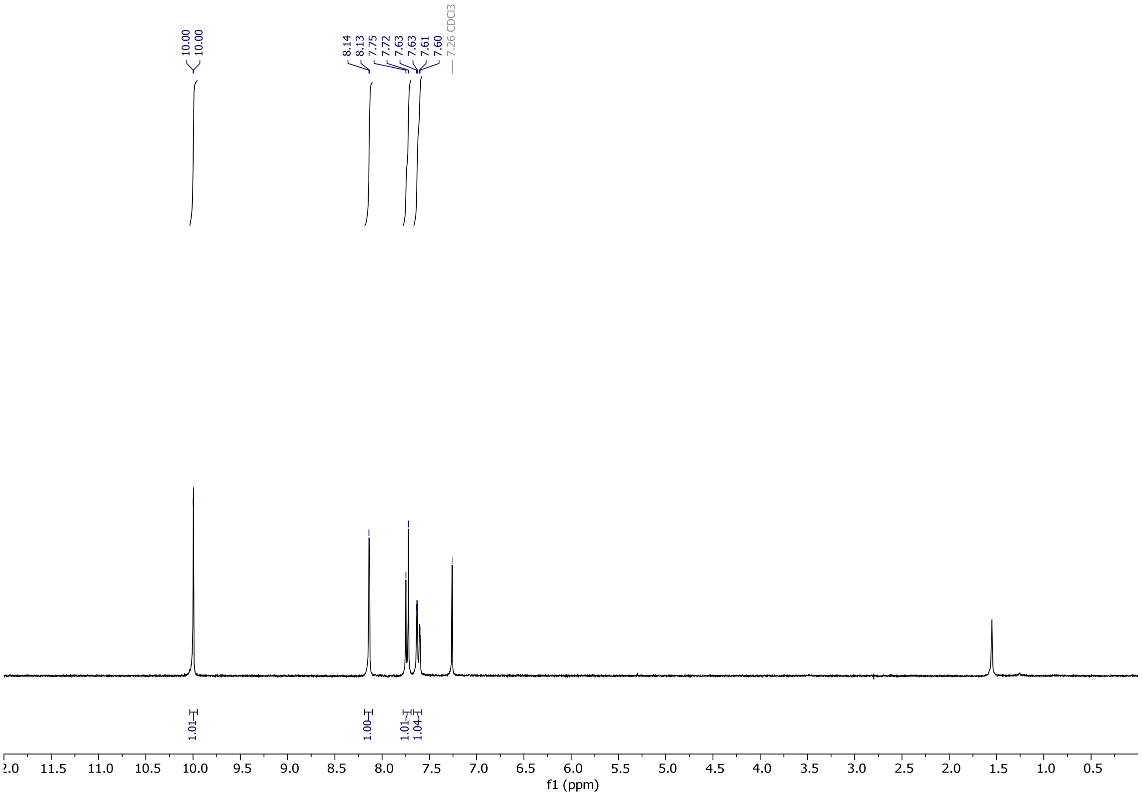

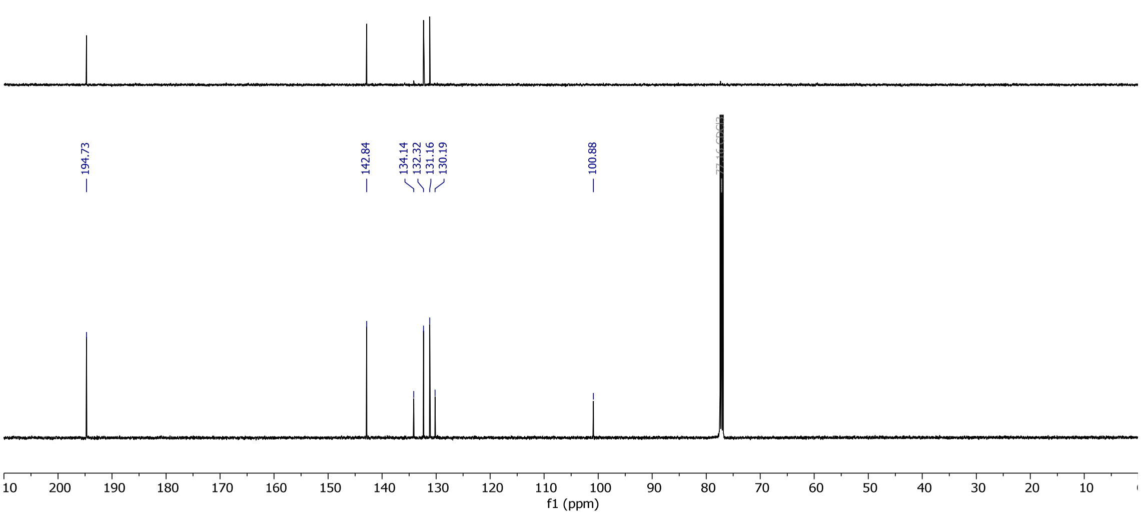

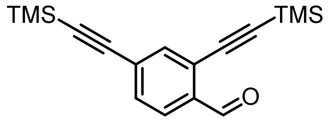

S2

^{1}H-NMR (500 Hz) and ^{13}C-NMR, DEPT (126 Hz) in $CDCl_3$

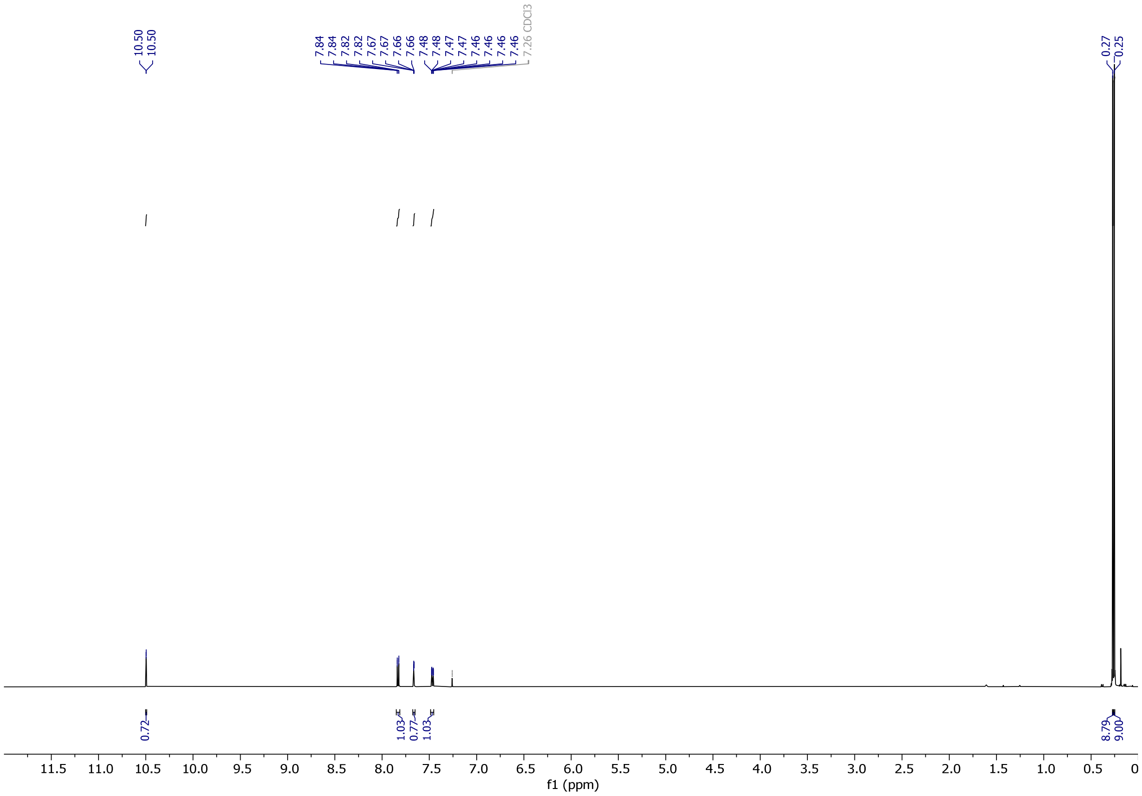

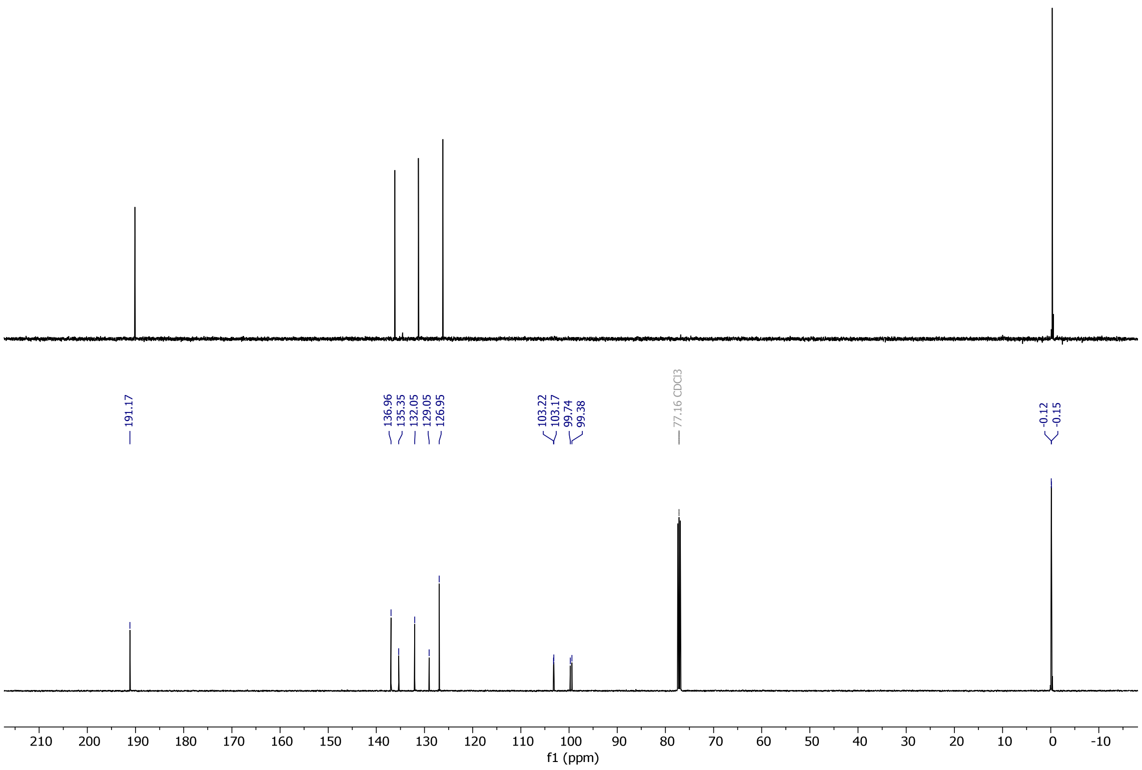

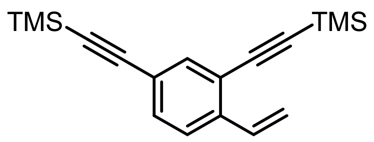

S3

^{1}H-NMR (300 Hz) and ^{13}C-NMR, DEPT (75 Hz) in $CDCl_3$

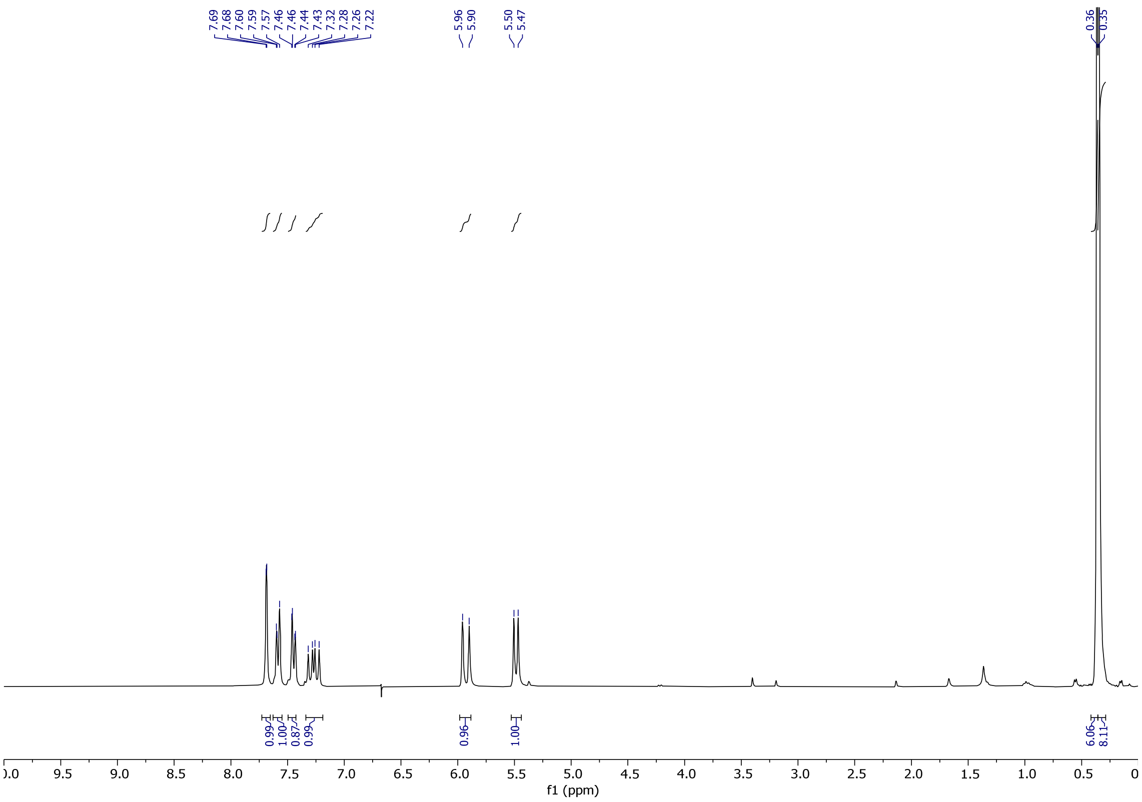

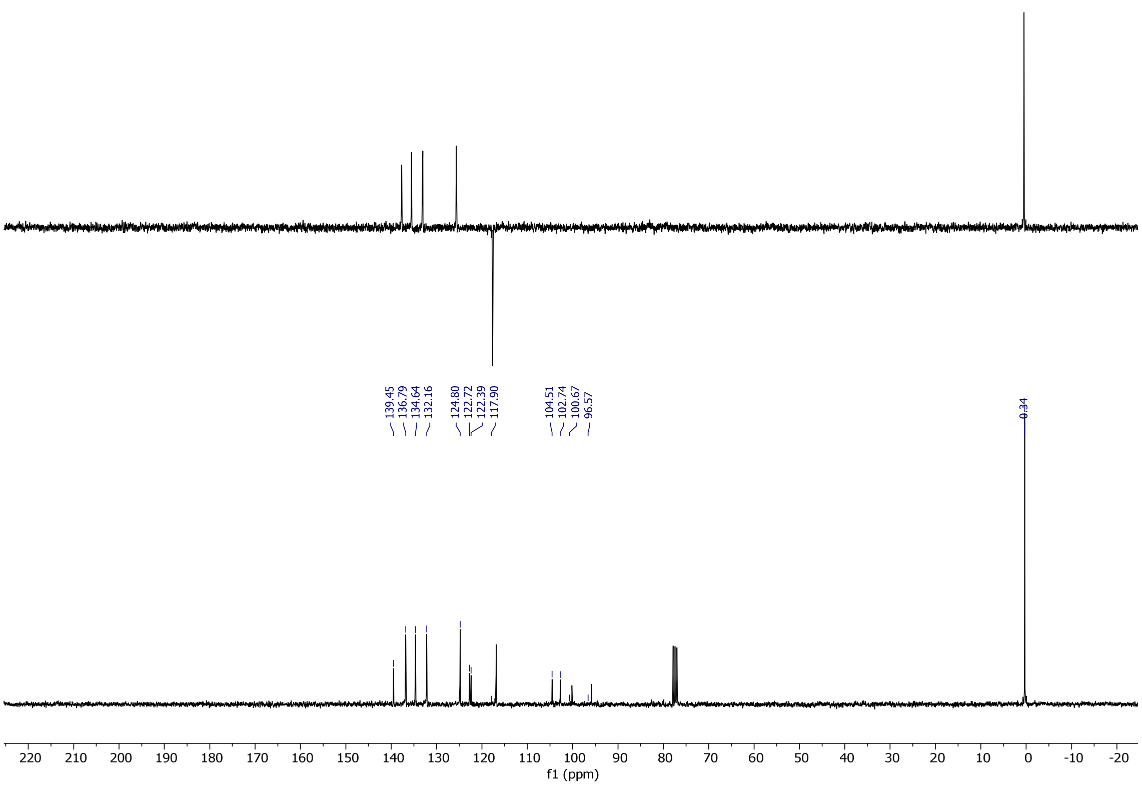

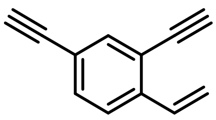

1d

^{1}H-NMR (300 Hz) and ^{13}C-NMR, DEPT (75 Hz) in $CDCl_3$

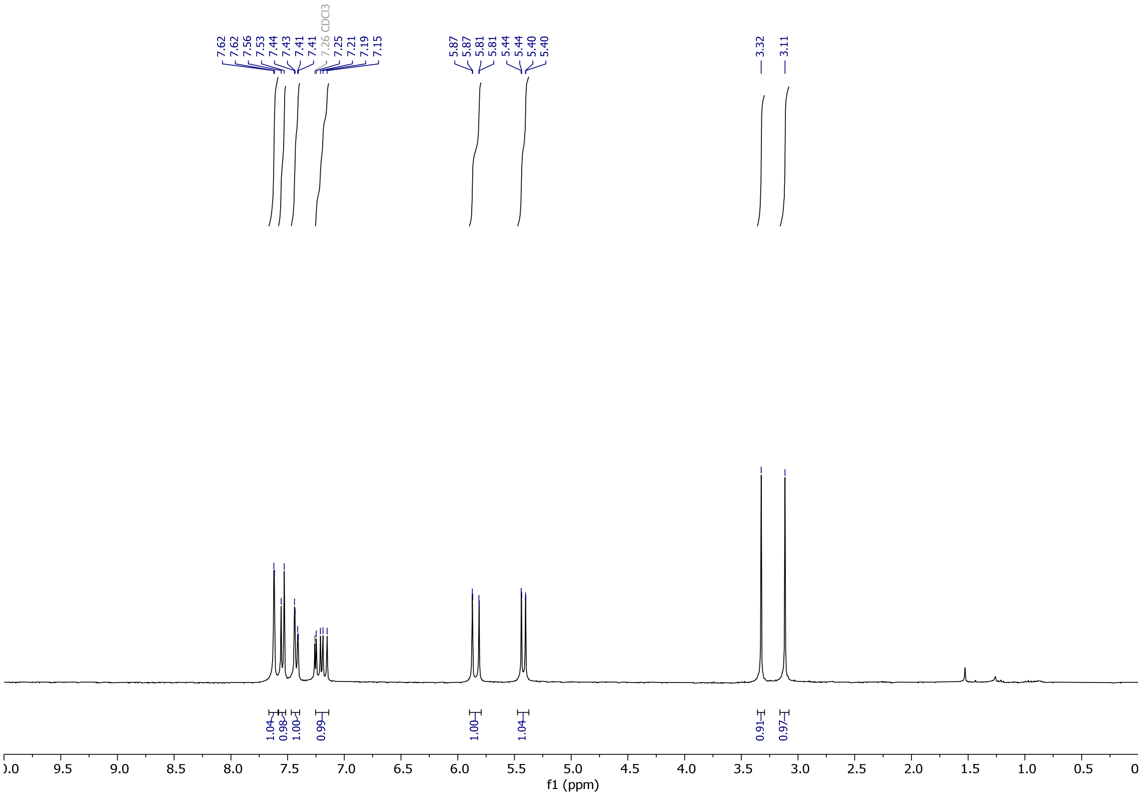

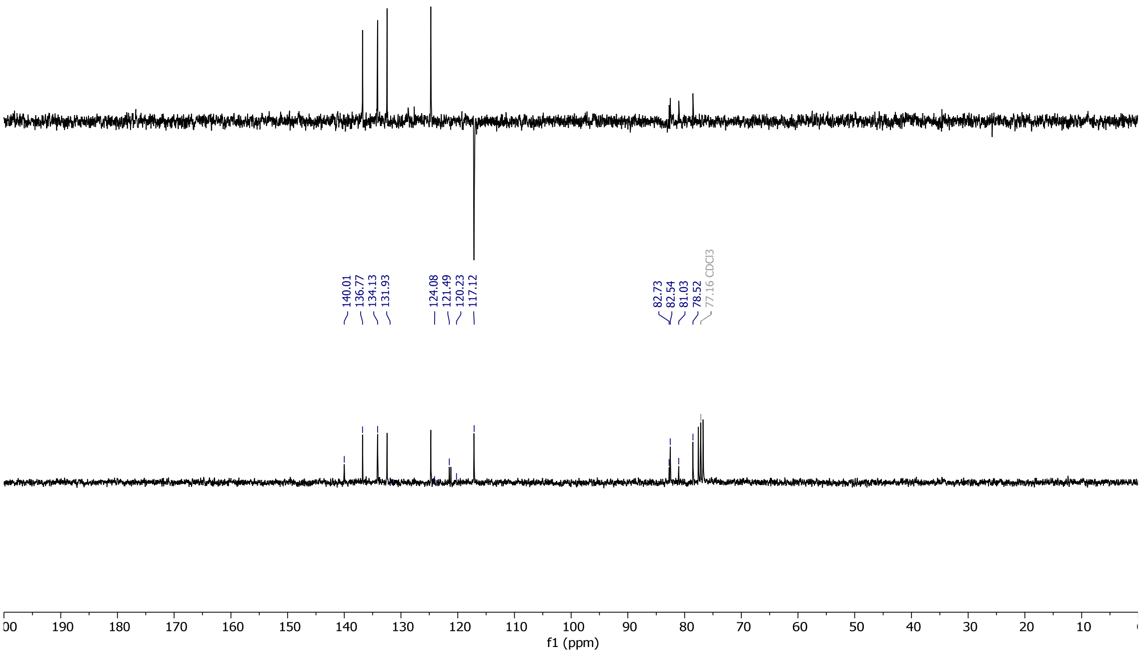

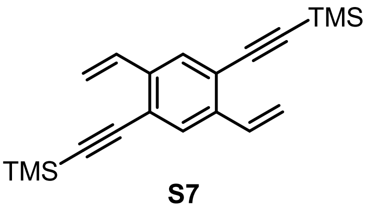

S7

^{1}H-NMR (500 Hz) and ^{13}C-NMR, DEPT (126 Hz) in $CDCl_3$

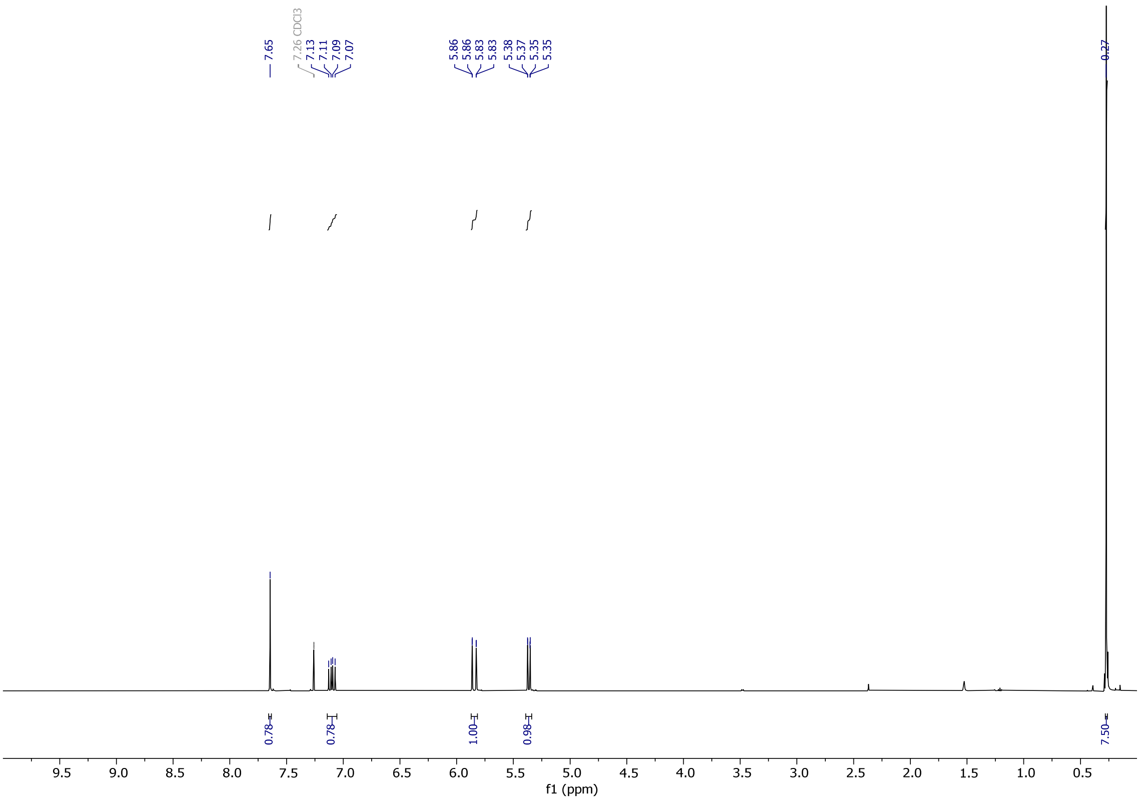

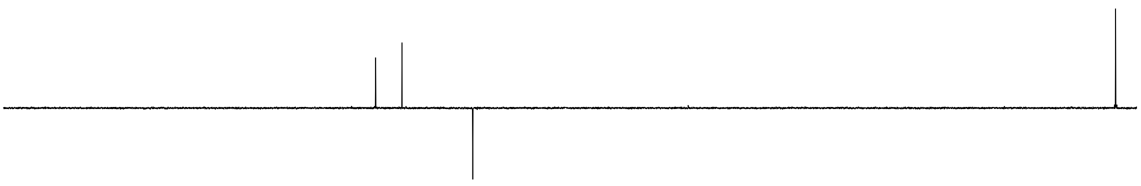

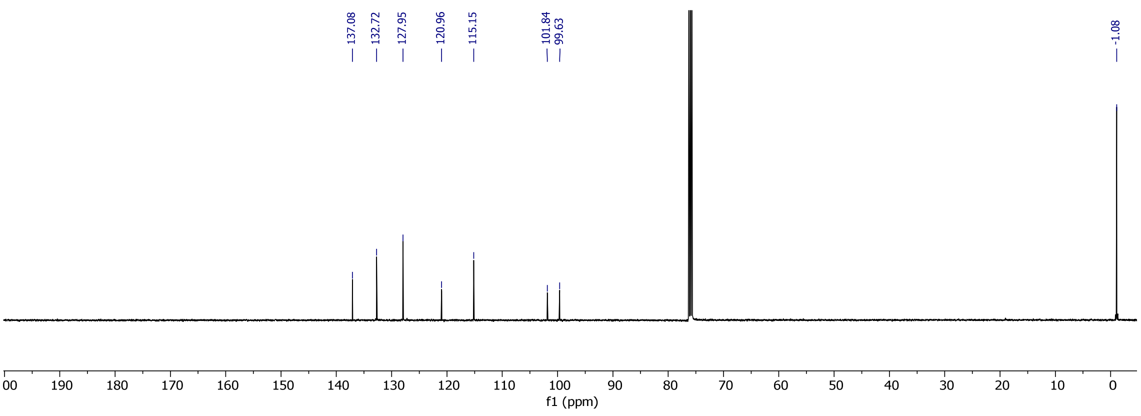

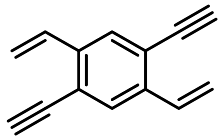

7

^{1}H-NMR (500 Hz) and ^{13}C-NMR, DEPT (126 Hz) in $CDCl_3$

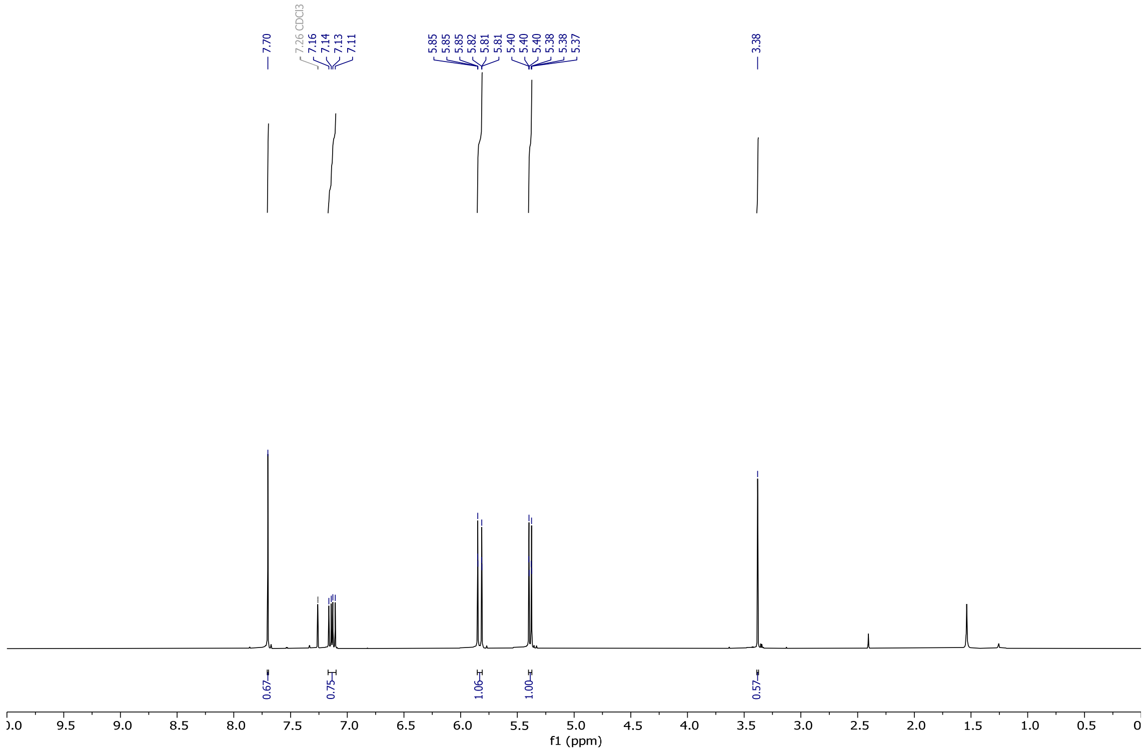

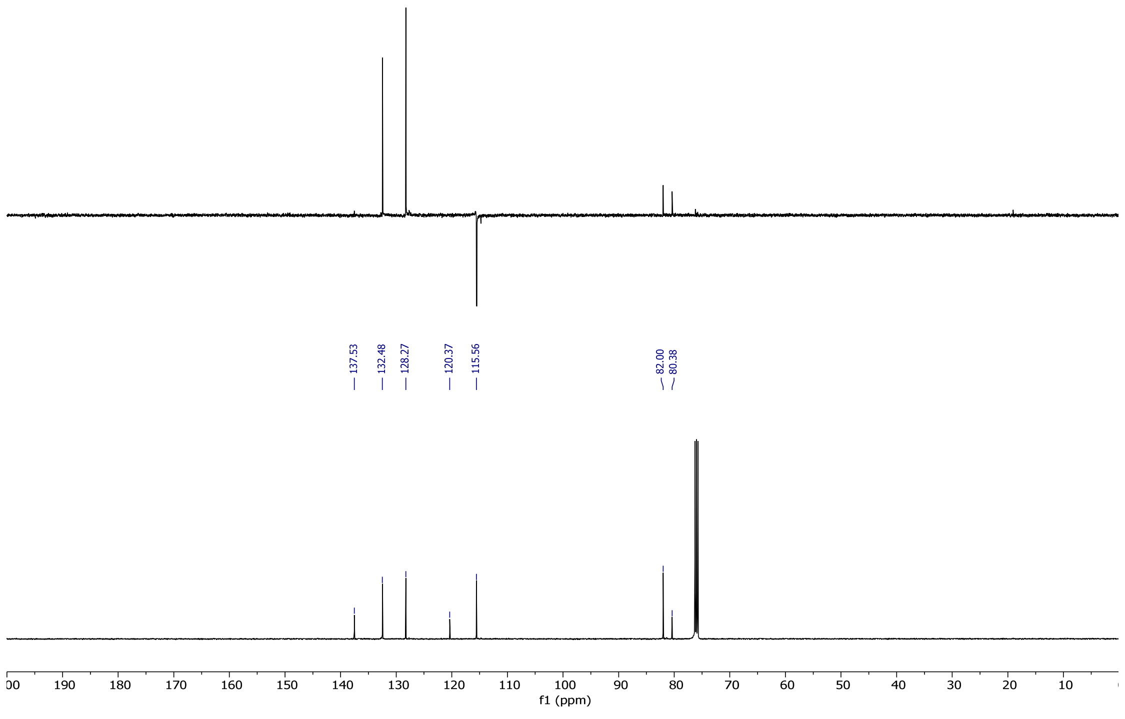

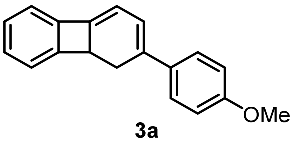

3a

^{1}H-NMR (300 Hz) and ^{13}C-NMR, DEPT (75 Hz) in $CDCl_3$

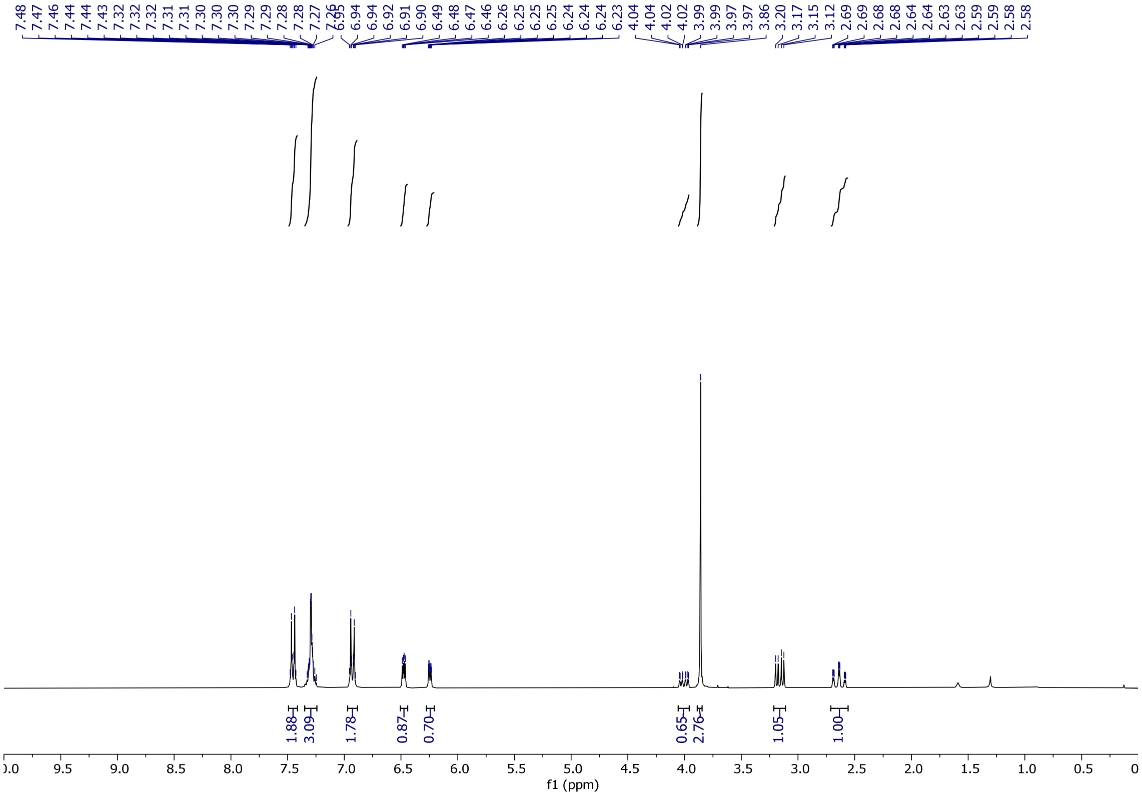

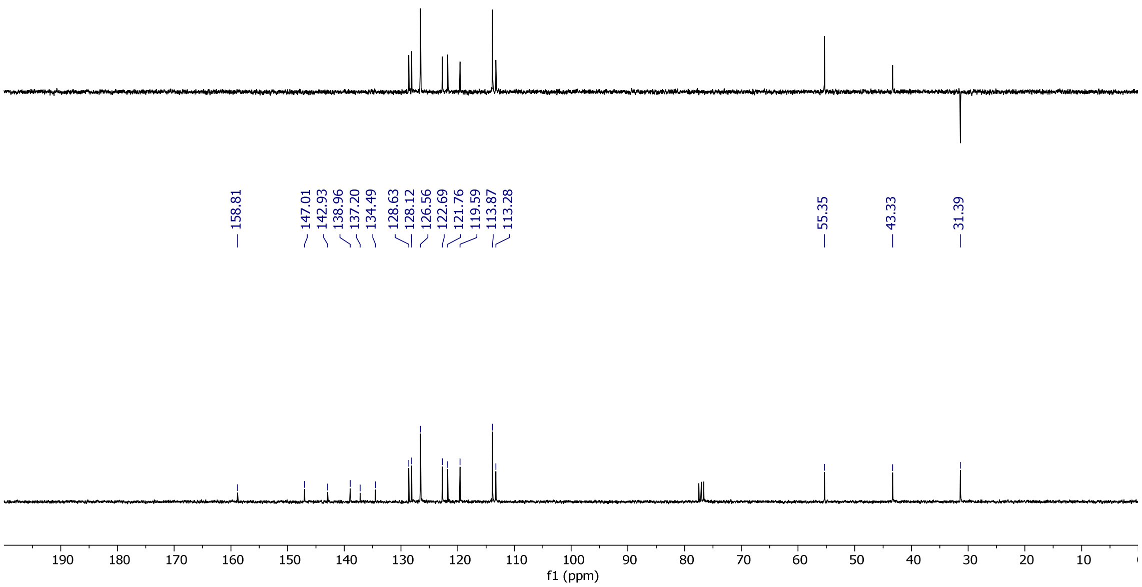

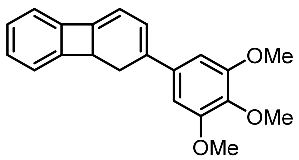

3b

^{1}H-NMR (300 Hz) and ^{13}C-NMR, DEPT (75 Hz) in $CDCl_3$

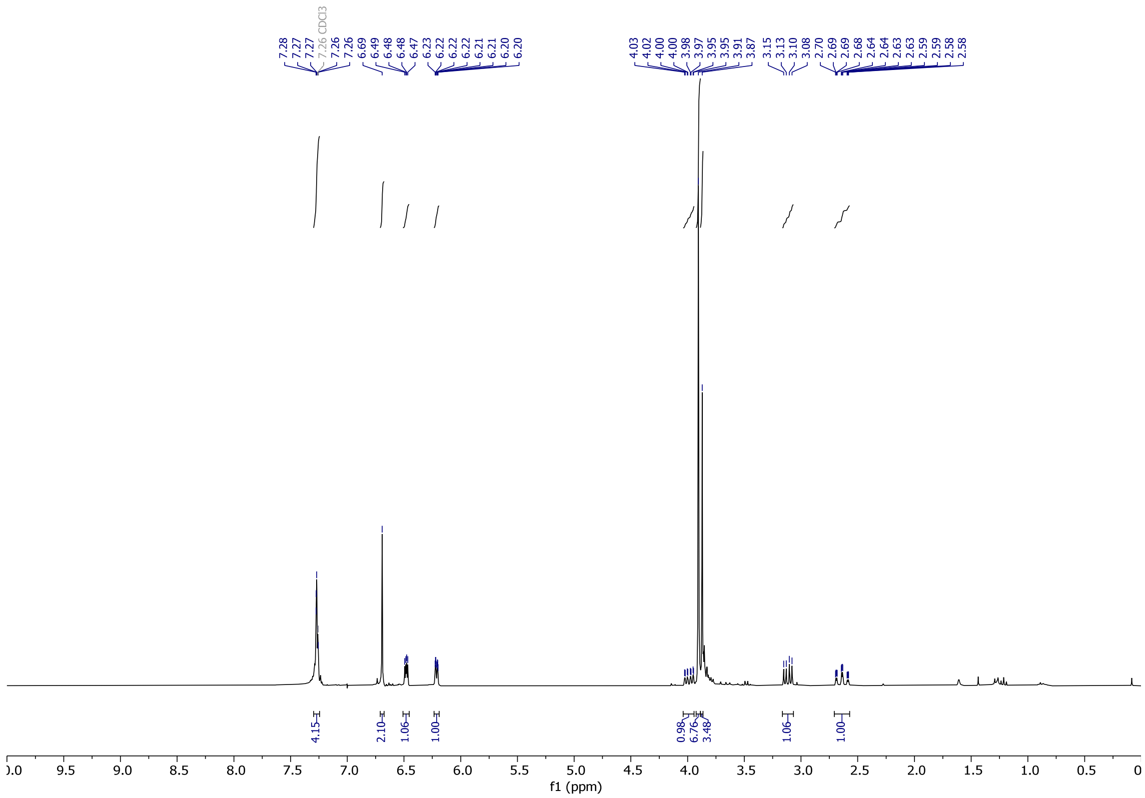

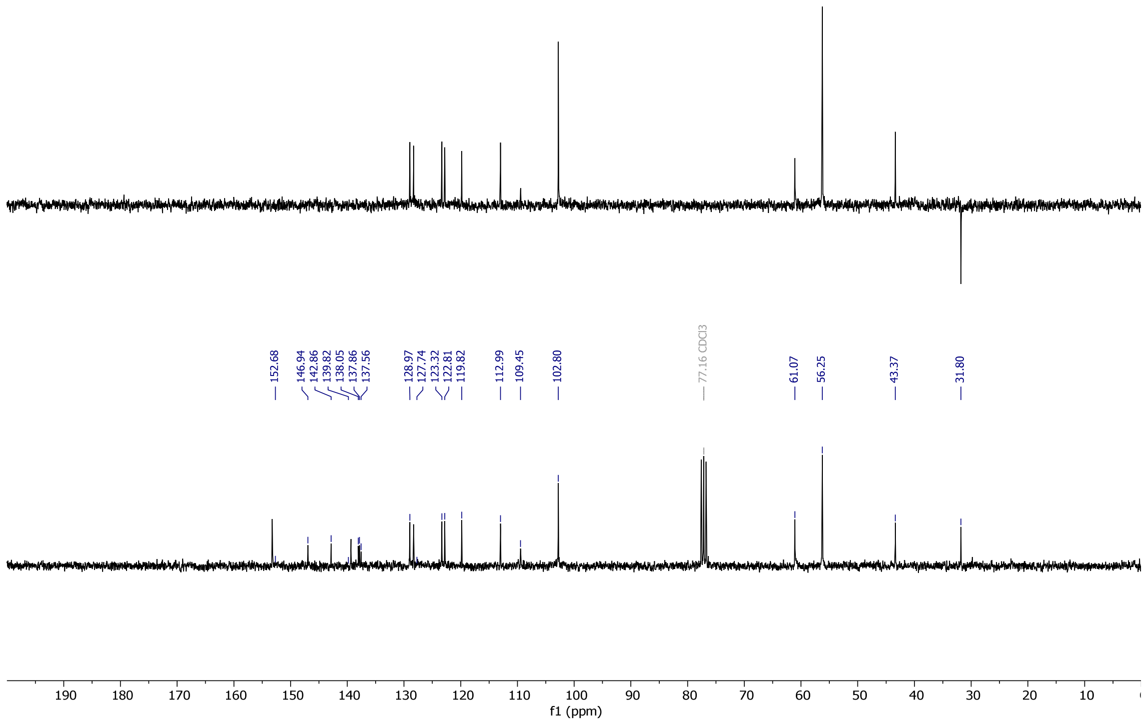

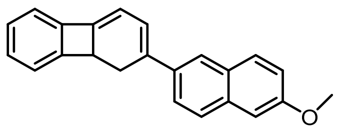

3c

^{1}H-NMR (300 Hz) and ^{13}C-NMR, DEPT (75 Hz) in $CDCl_3$

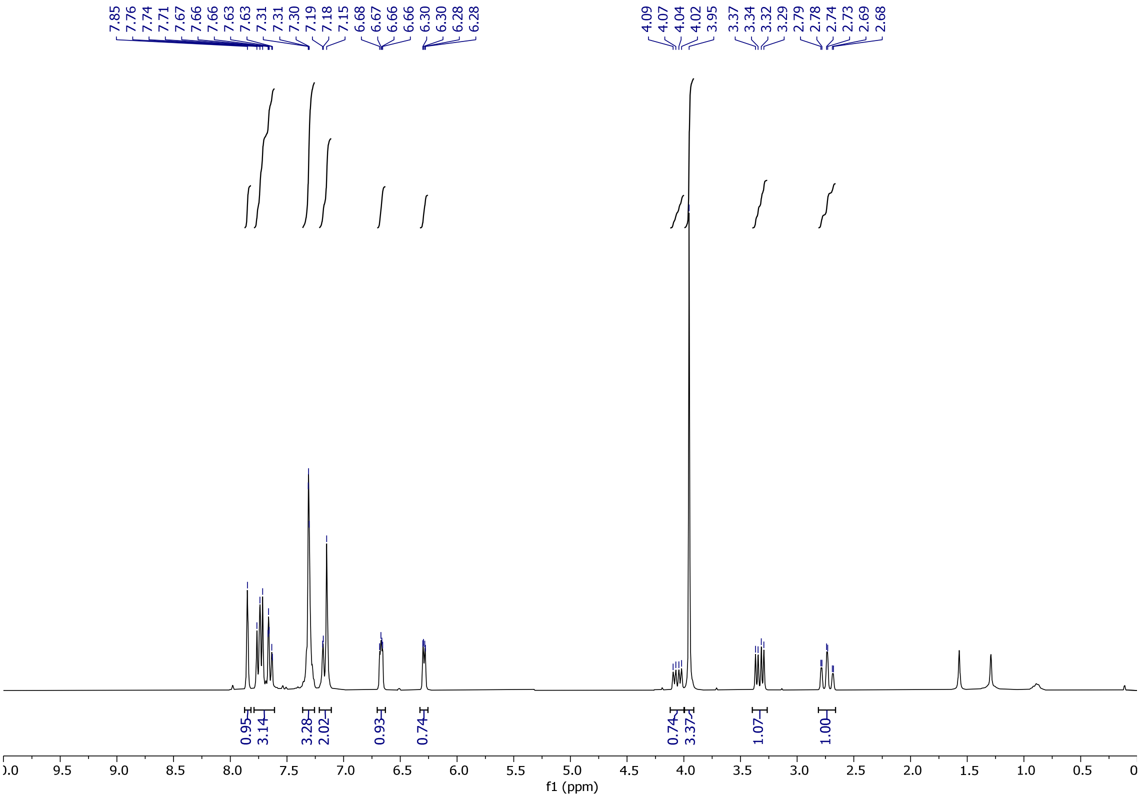

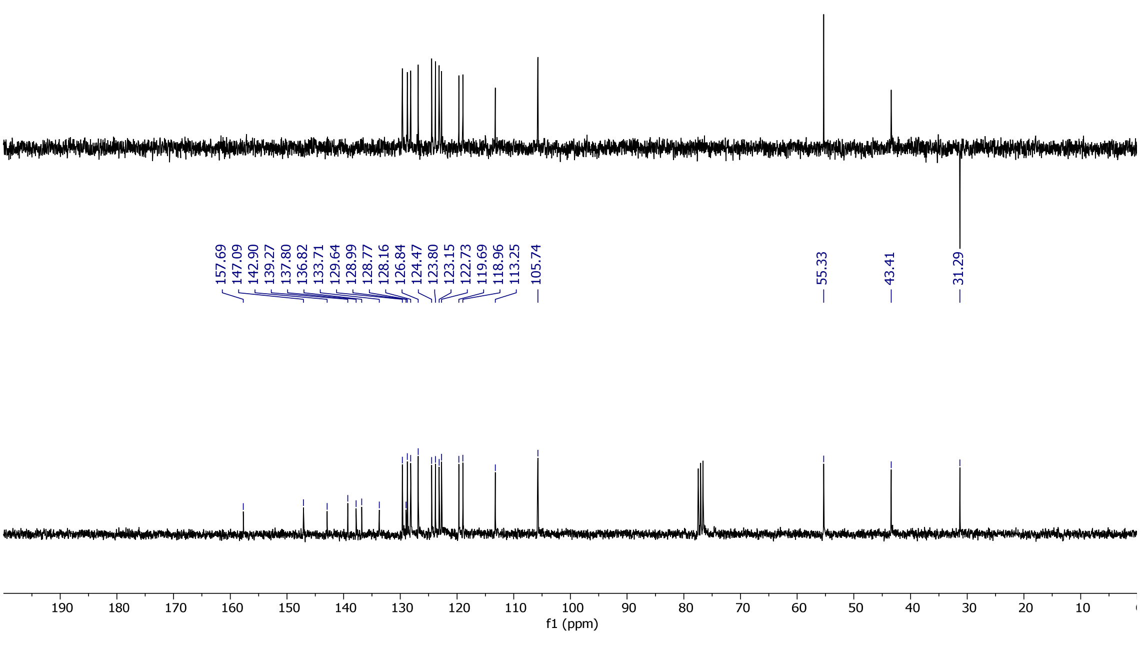

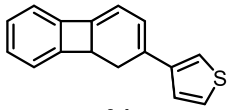

3d

^{1}H-NMR (300 Hz) and ^{13}C-NMR, DEPT (75 Hz) in $CDCl_3$

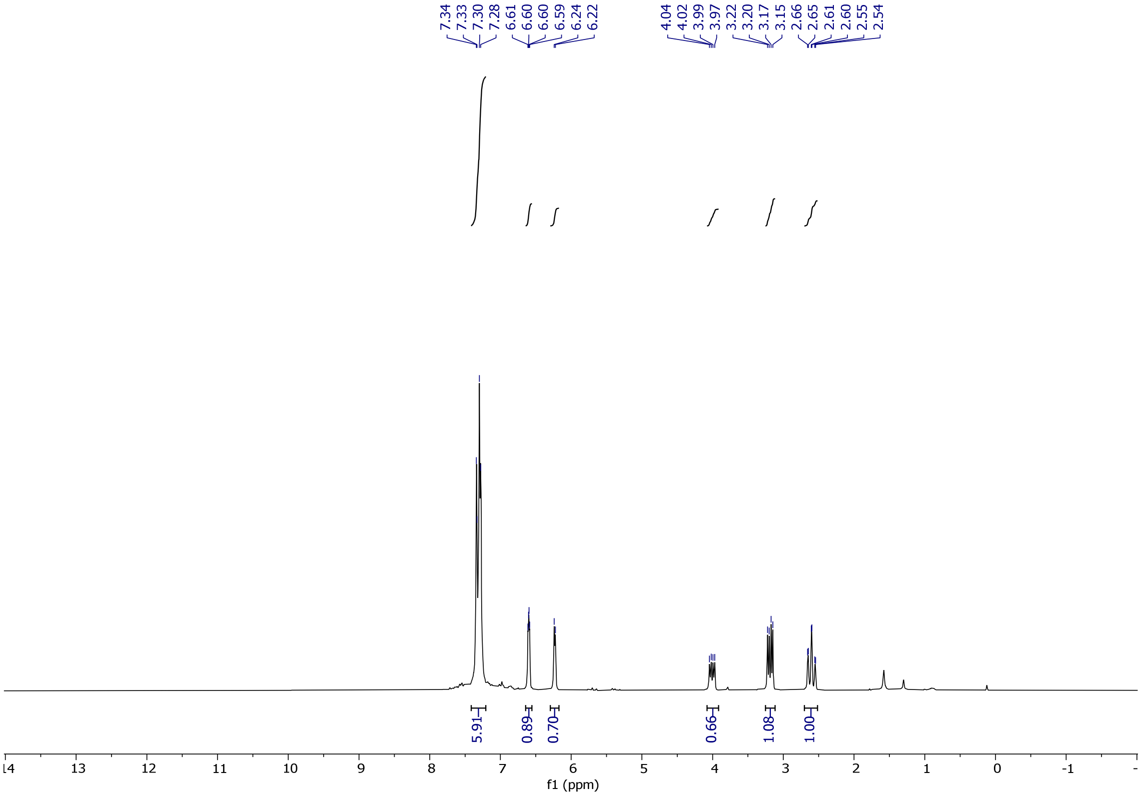

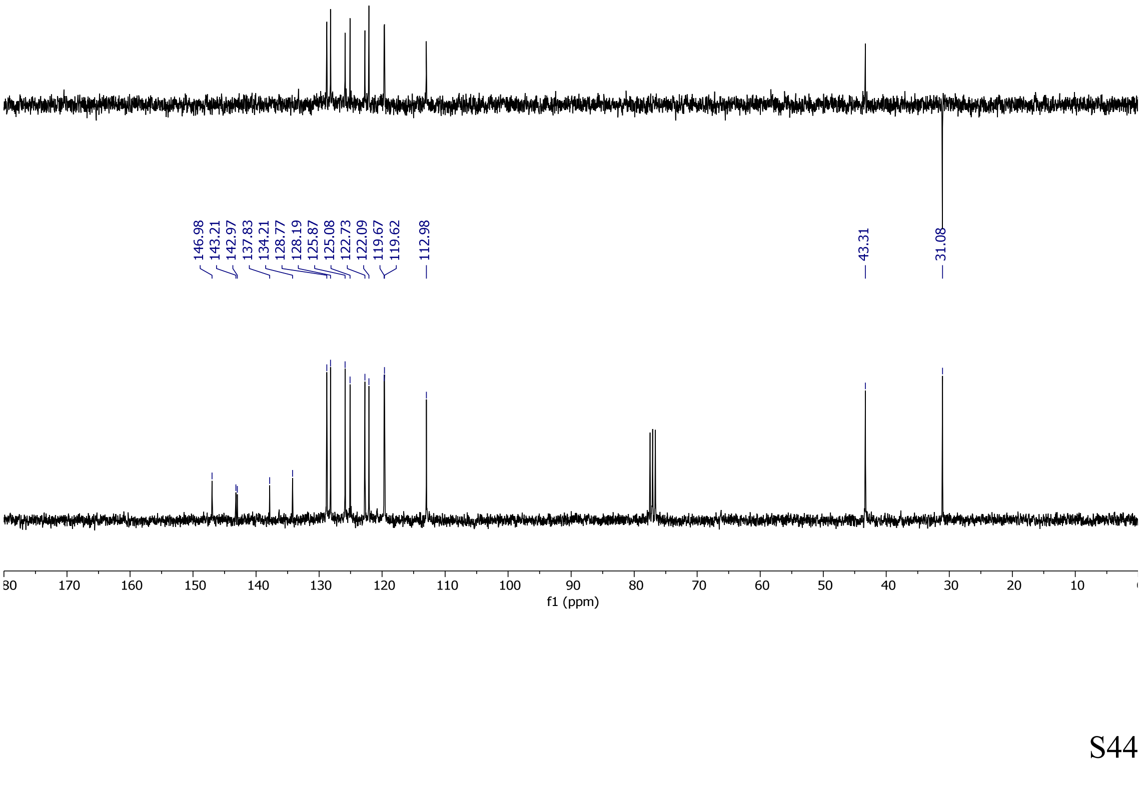

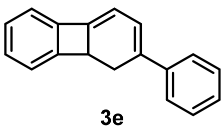

3e

^{1}H-NMR (300 Hz) and ^{13}C-NMR, DEPT (75 Hz) in $CDCl_3$

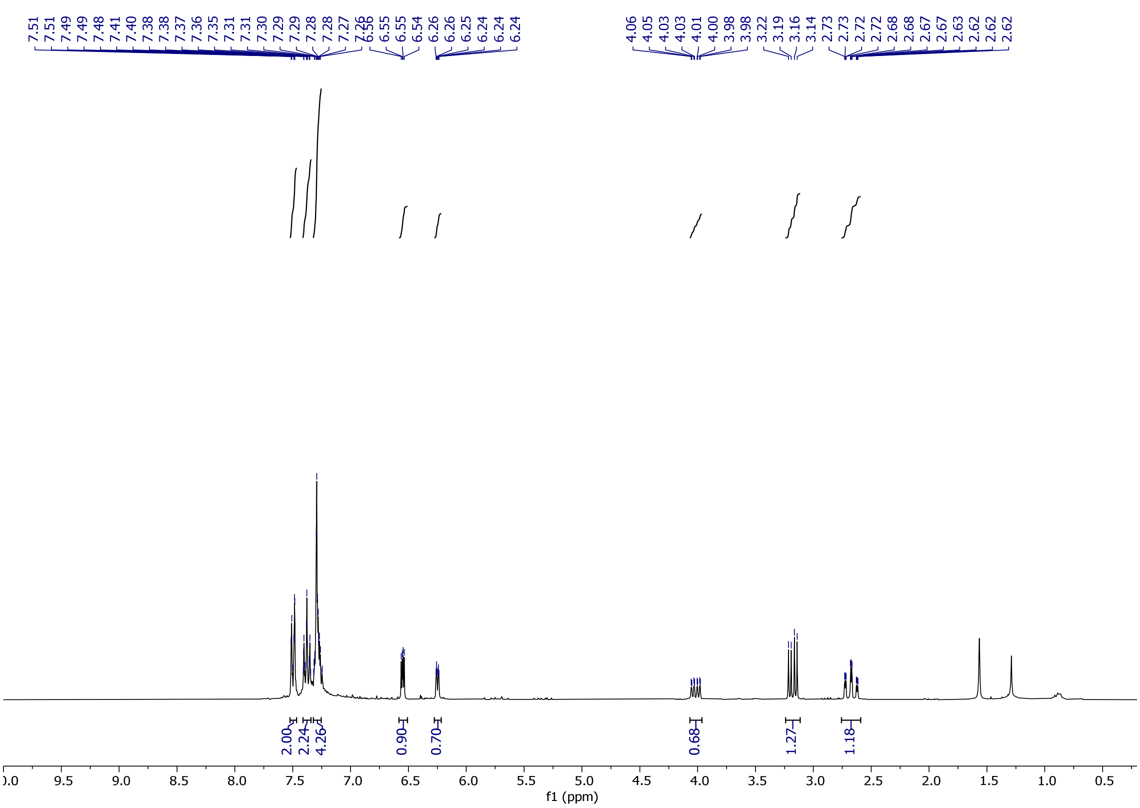

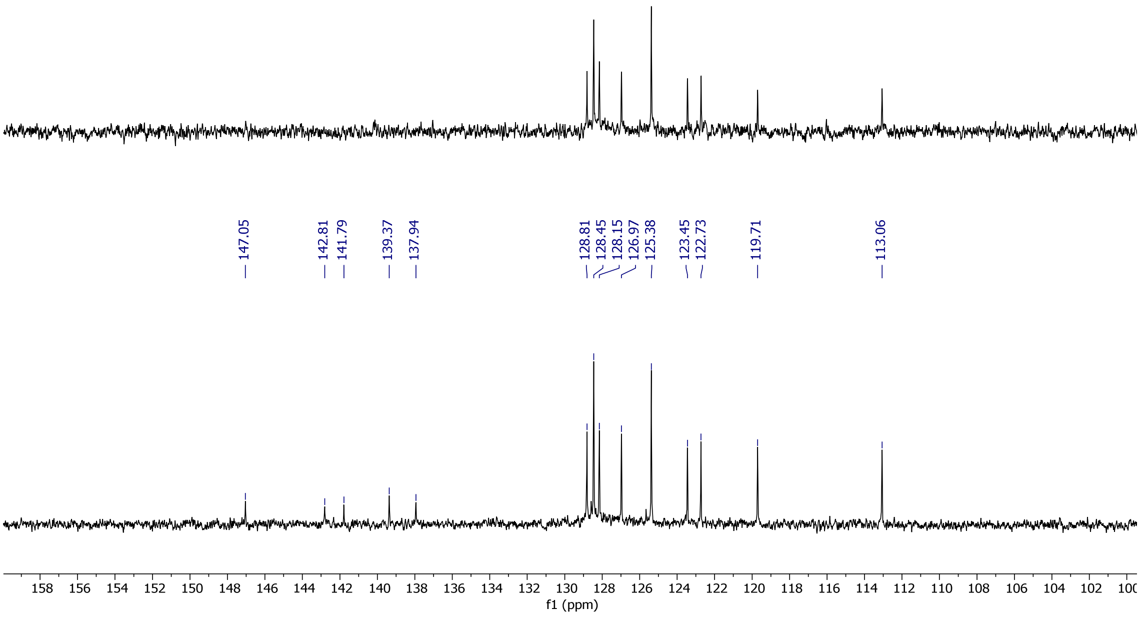

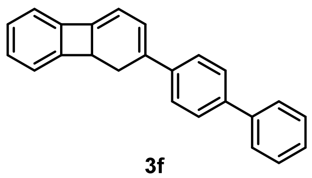

3f

^{1}H-NMR (300 Hz) and ^{13}C-NMR, DEPT (75 Hz) in $CDCl_3$

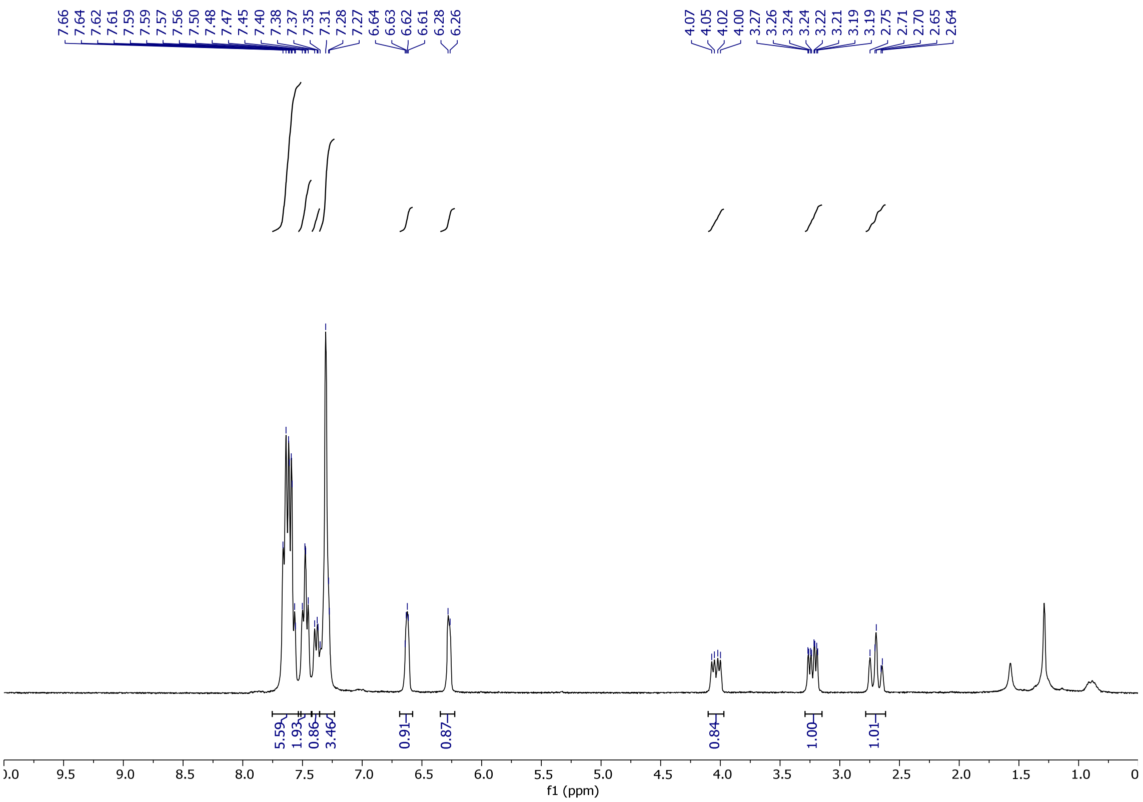

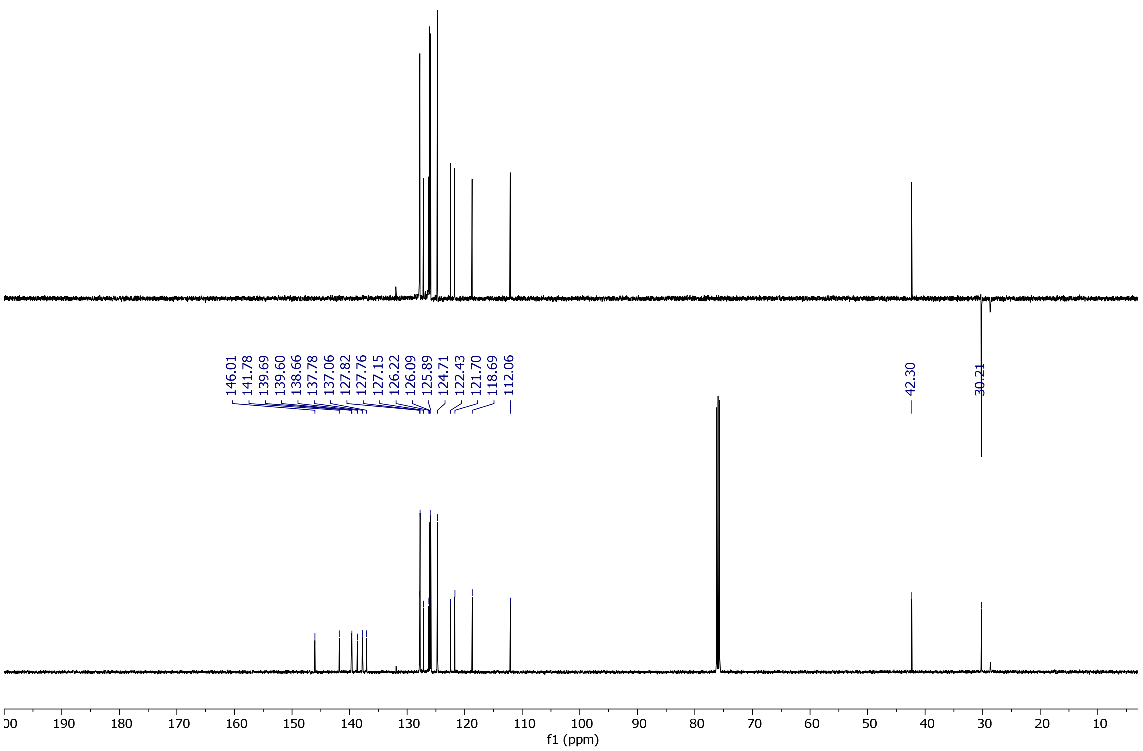

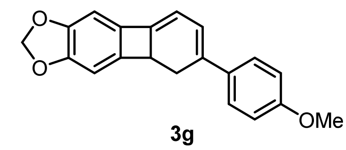

3g

^{1}H-NMR (500 Hz) and ^{13}C-NMR, DEPT (126 Hz) in $CDCl_3$

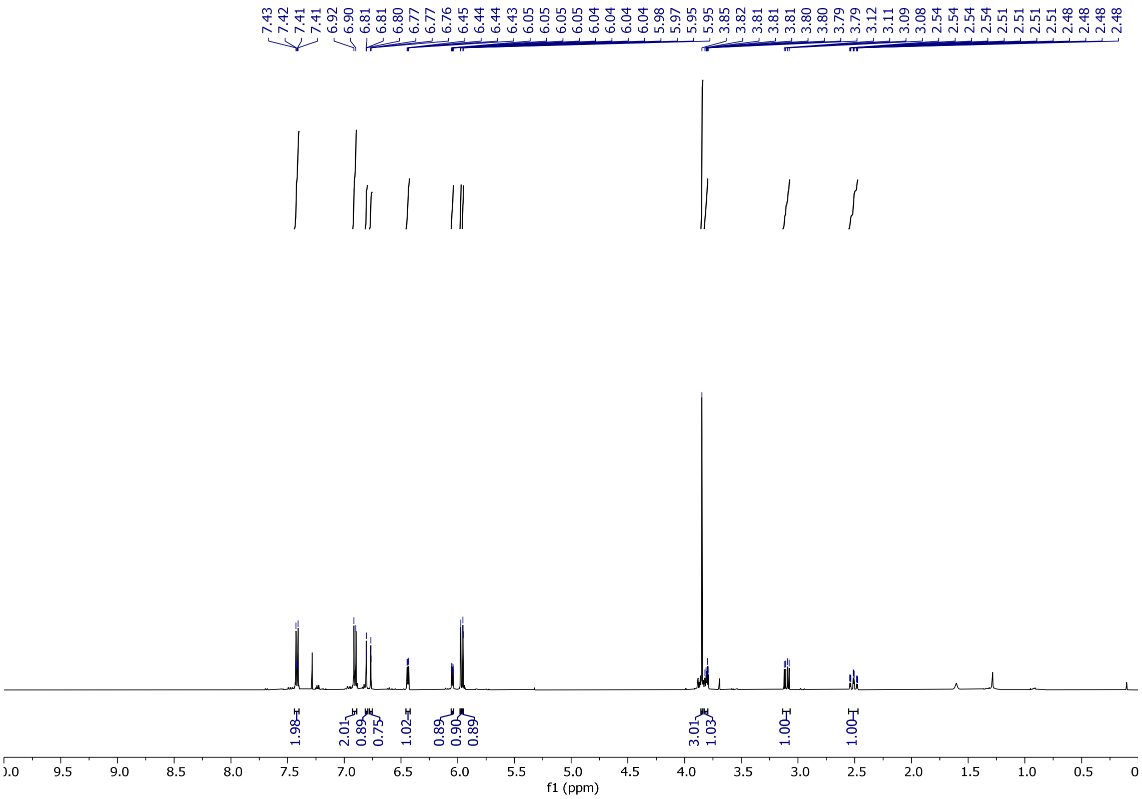

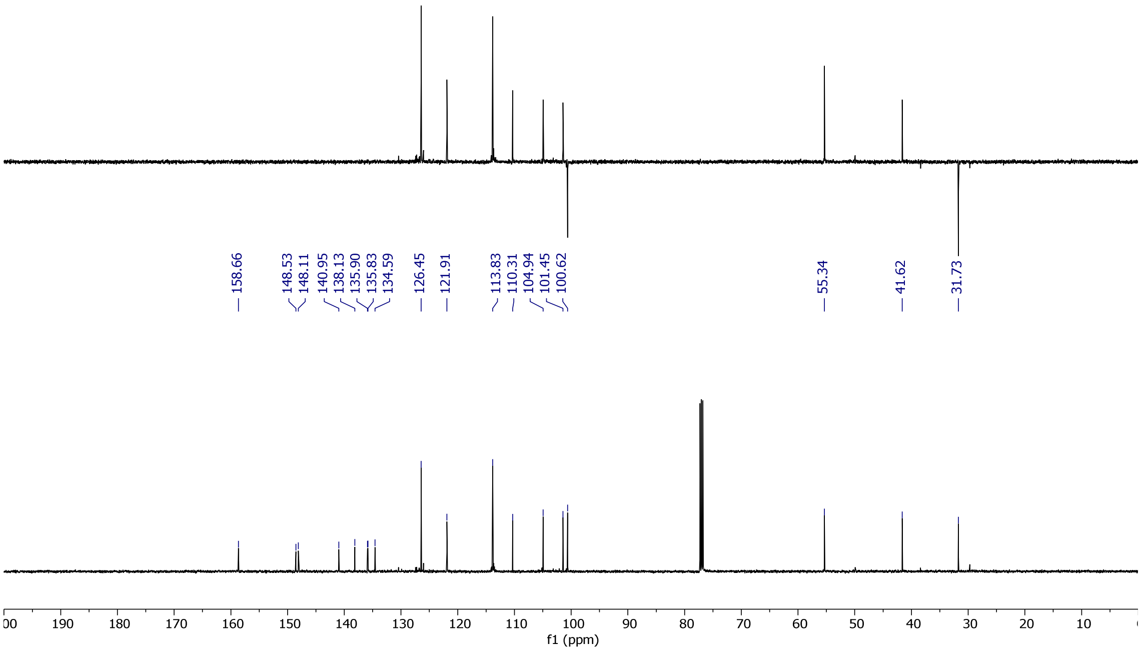

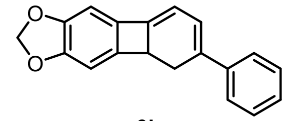

3h

^{1}H-NMR (300 Hz) and ^{13}C-NMR, DEPT (75 Hz) in $CDCl_3$

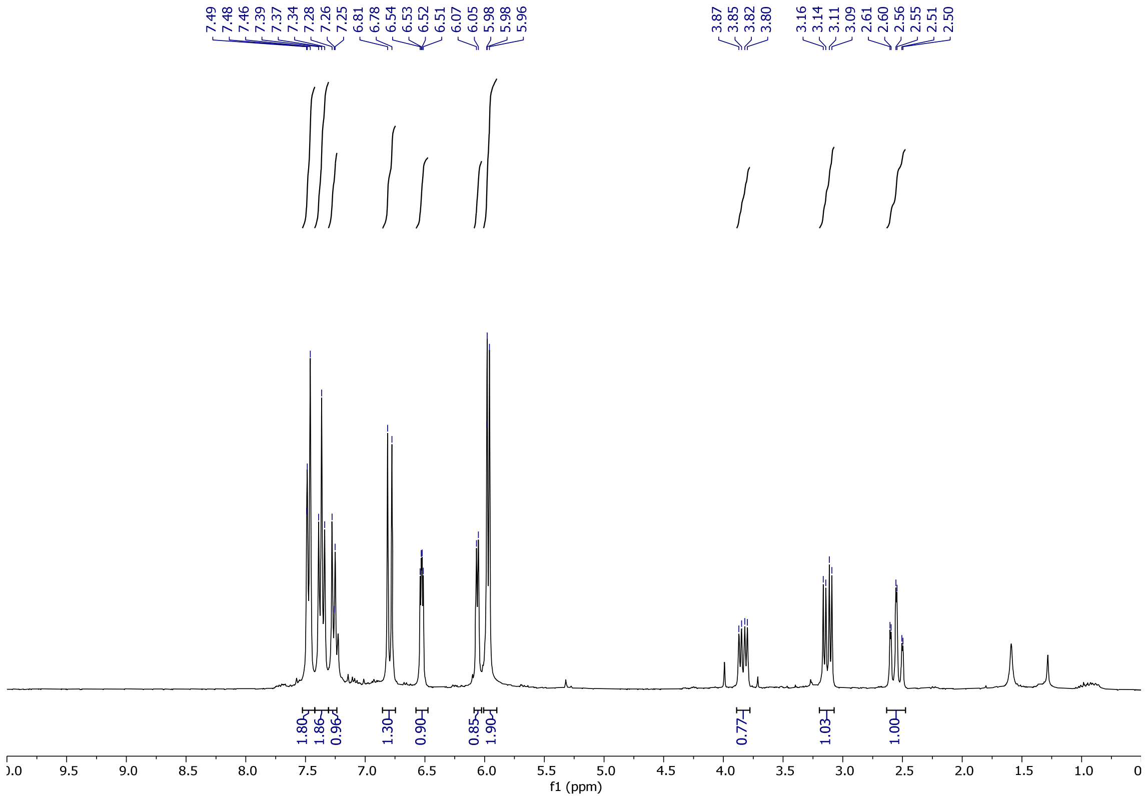

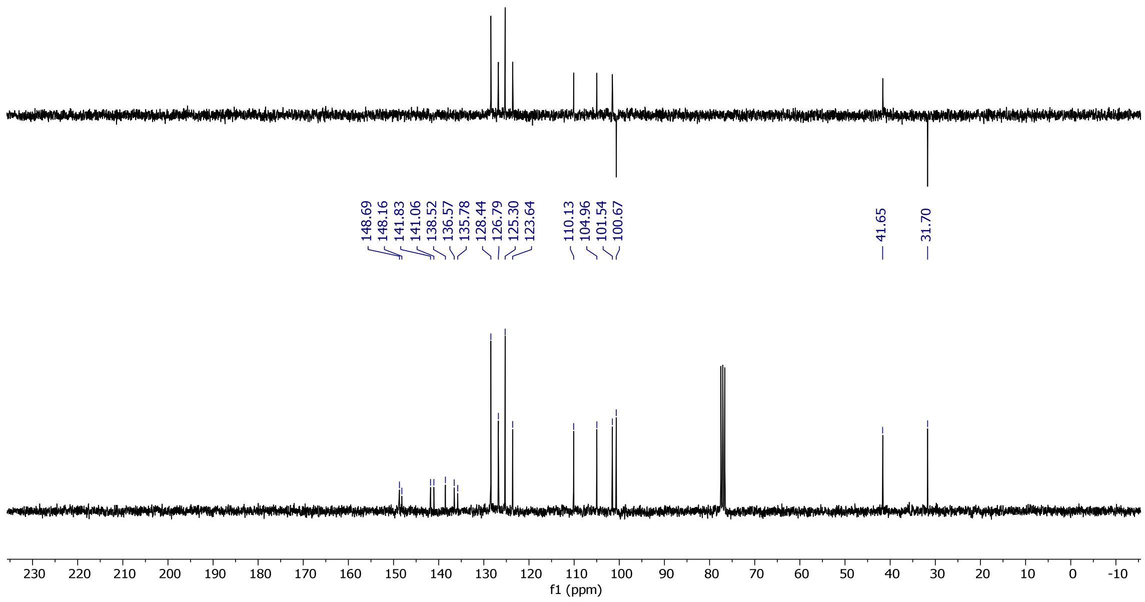

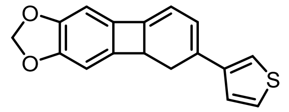

3i

^{1}H-NMR (300 Hz) and ^{13}C-NMR, DEPT (75 Hz) in $CDCl_3$

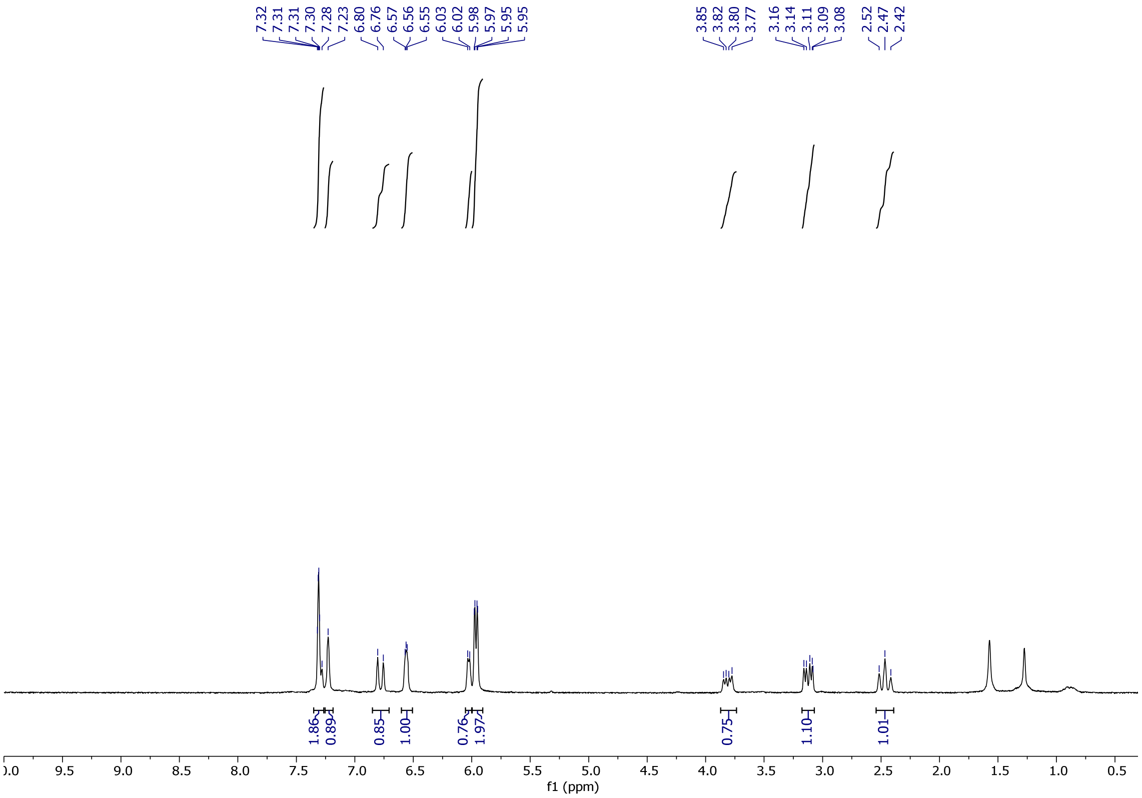

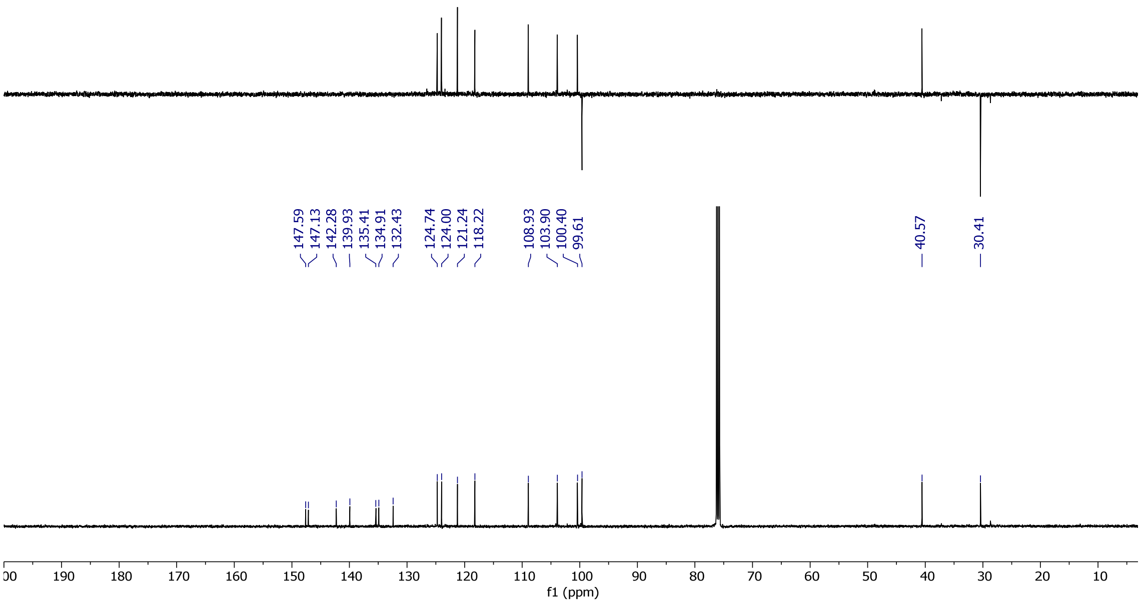

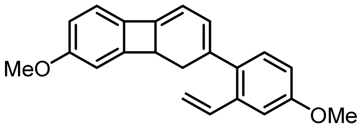

3j

^{1}H-NMR (500 Hz) and ^{13}C-NMR, DEPT (126 Hz) in $CDCl_3$

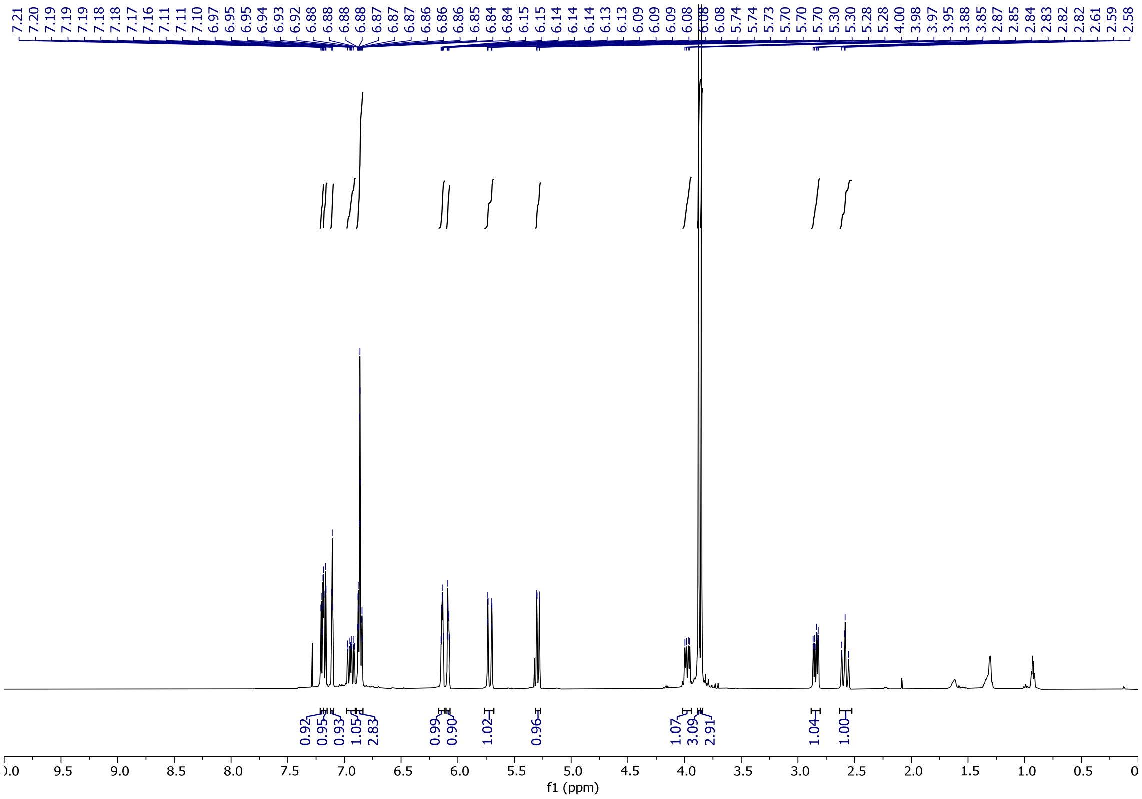

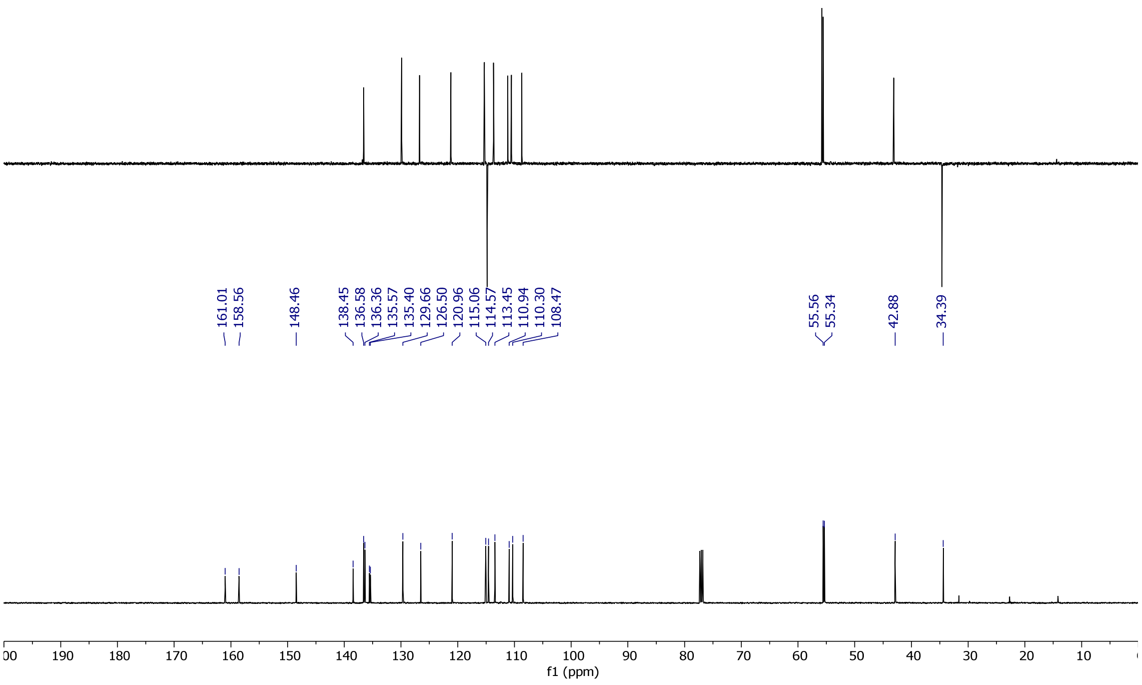

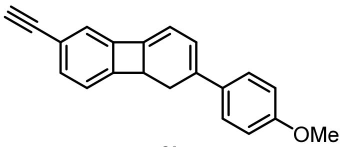

3k

^{1}H-NMR (300 Hz) and ^{13}C-NMR, DEPT (75 Hz) in $CDCl_3$

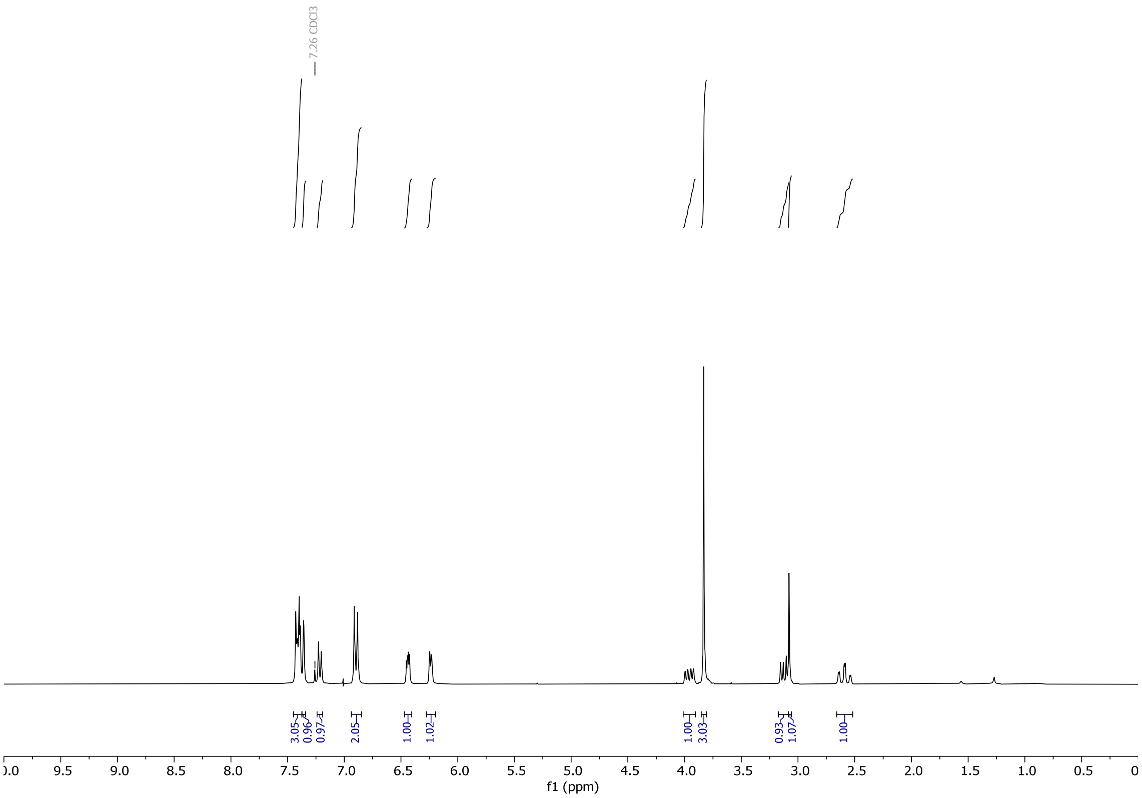

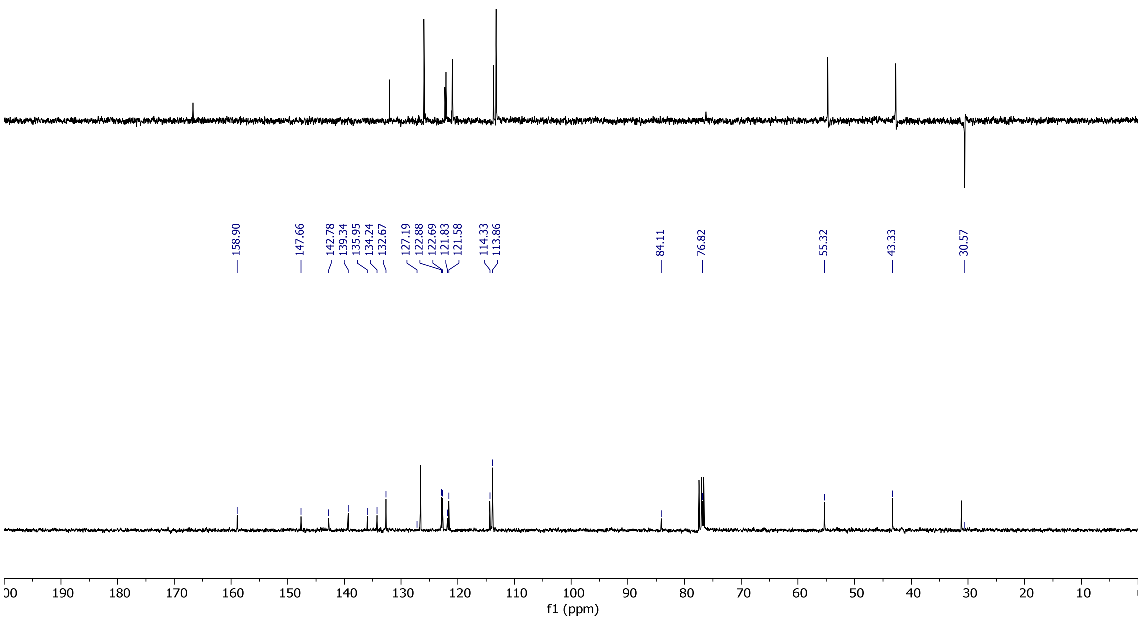

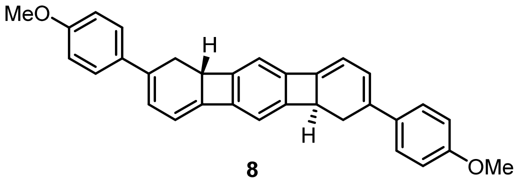

8 ^{1}H-NMR (500 Hz) and ^{13}C-NMR, DEPT (126 Hz) in 1,1,2,2-tetrachloroethane-*d*2 at 50ºC

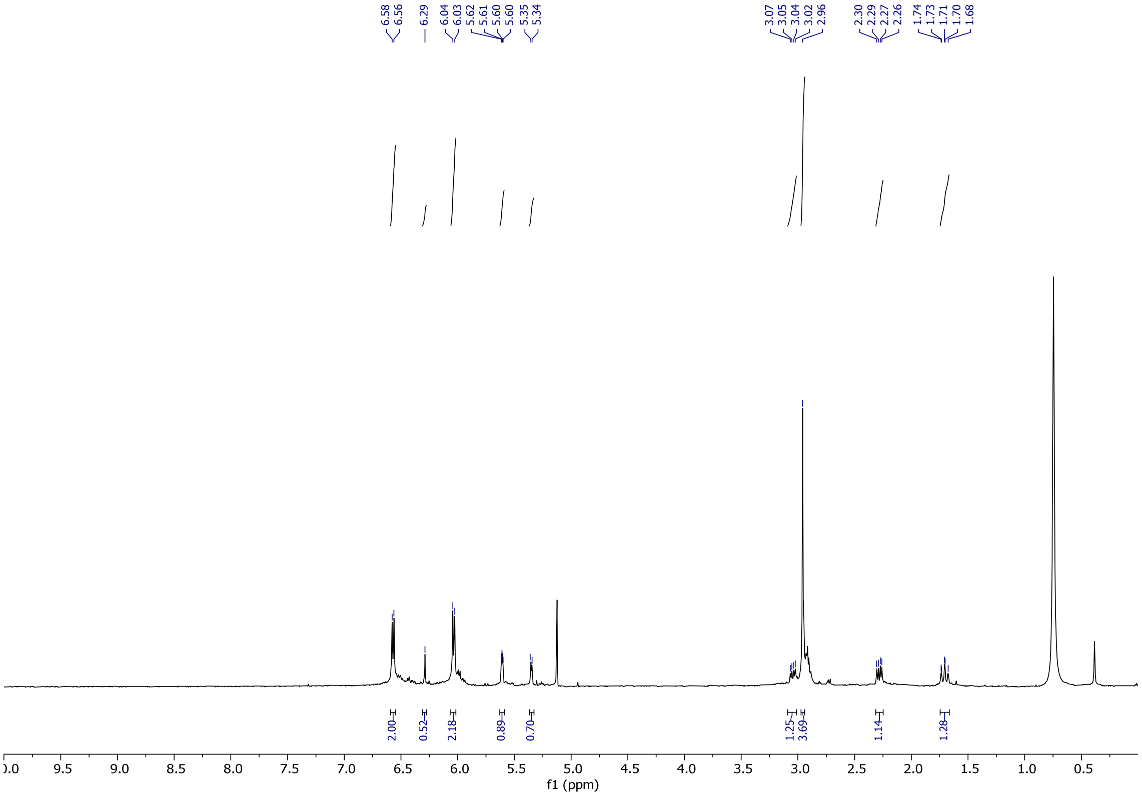

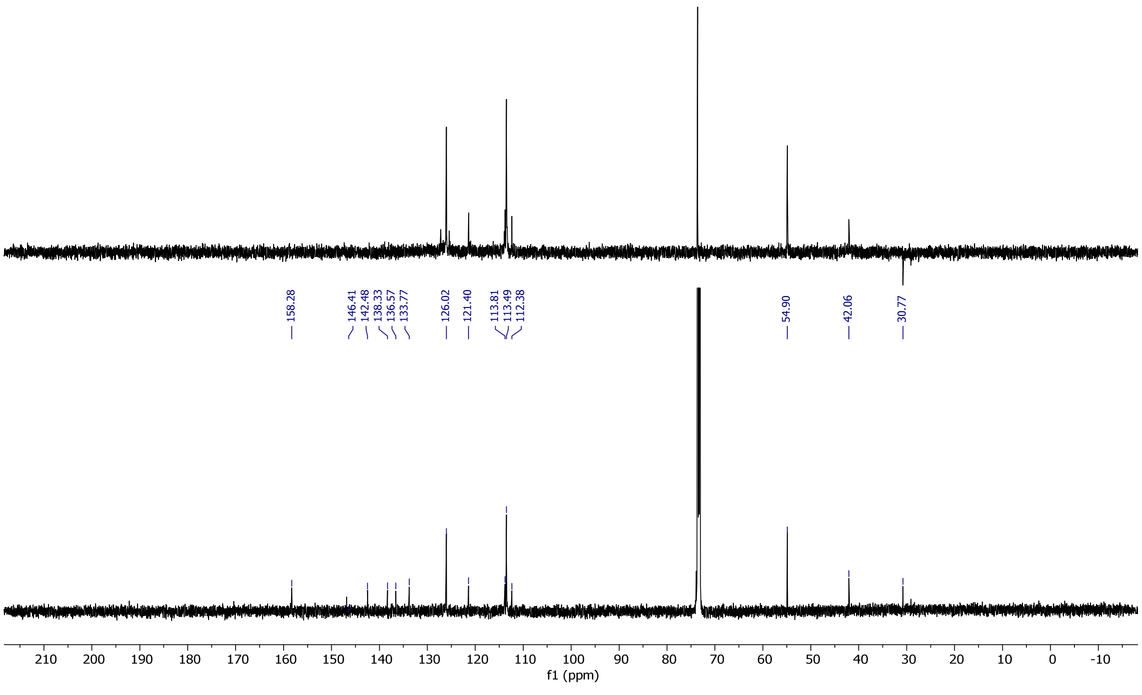

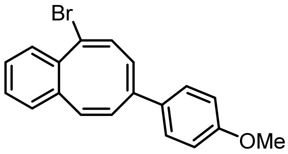

4a

^{1}H-NMR (500 Hz) and ^{13}C-NMR, DEPT (126 Hz) in $CDCl_3$

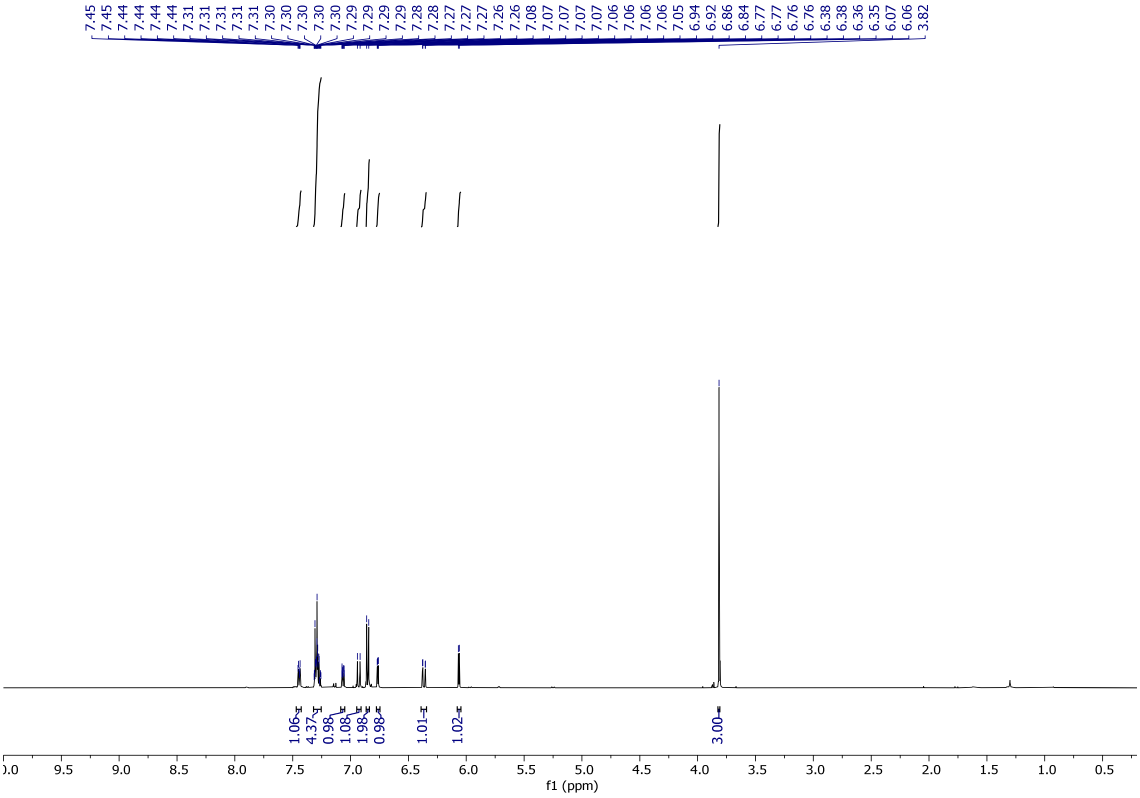

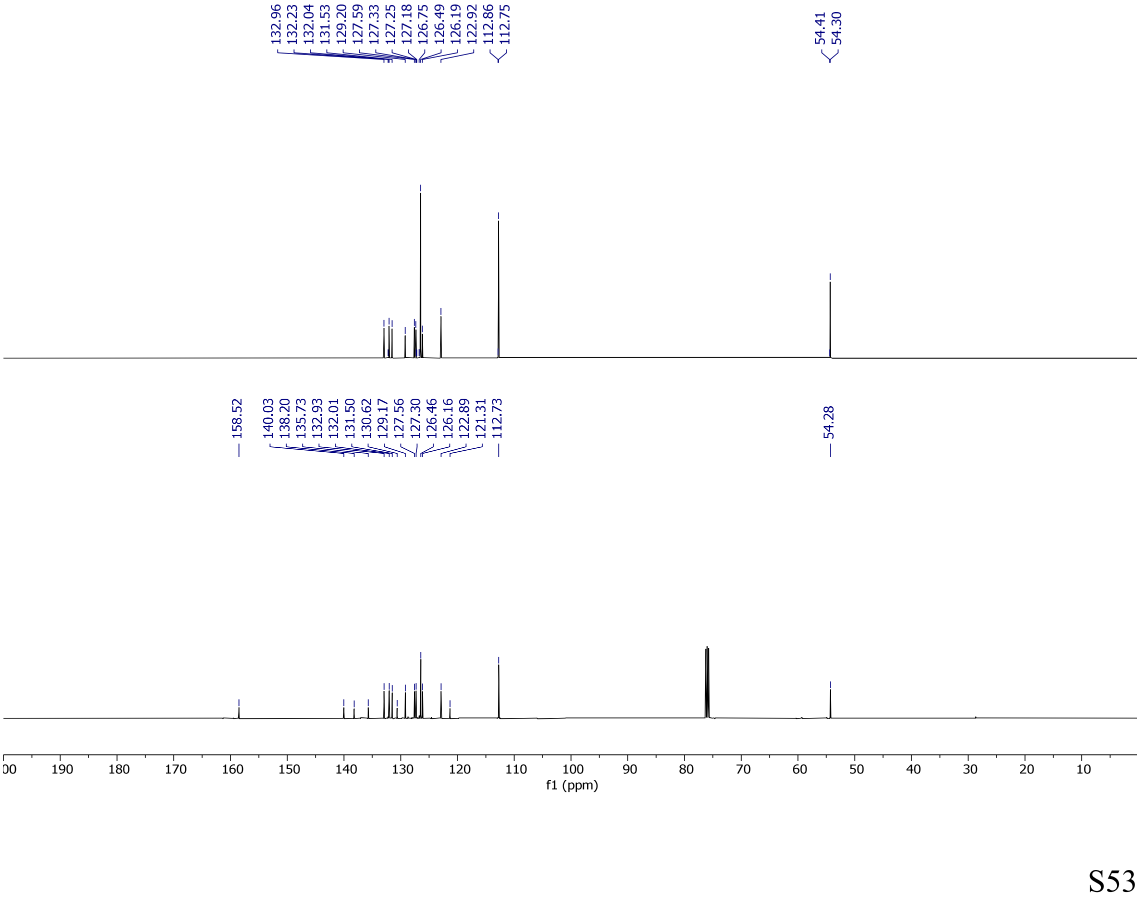

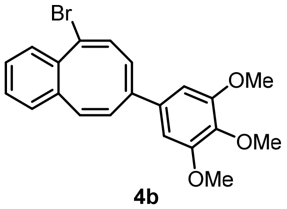

4b

^{1}H-NMR (300 Hz) and ^{13}C-NMR, DEPT (75 Hz) in $CDCl_3$

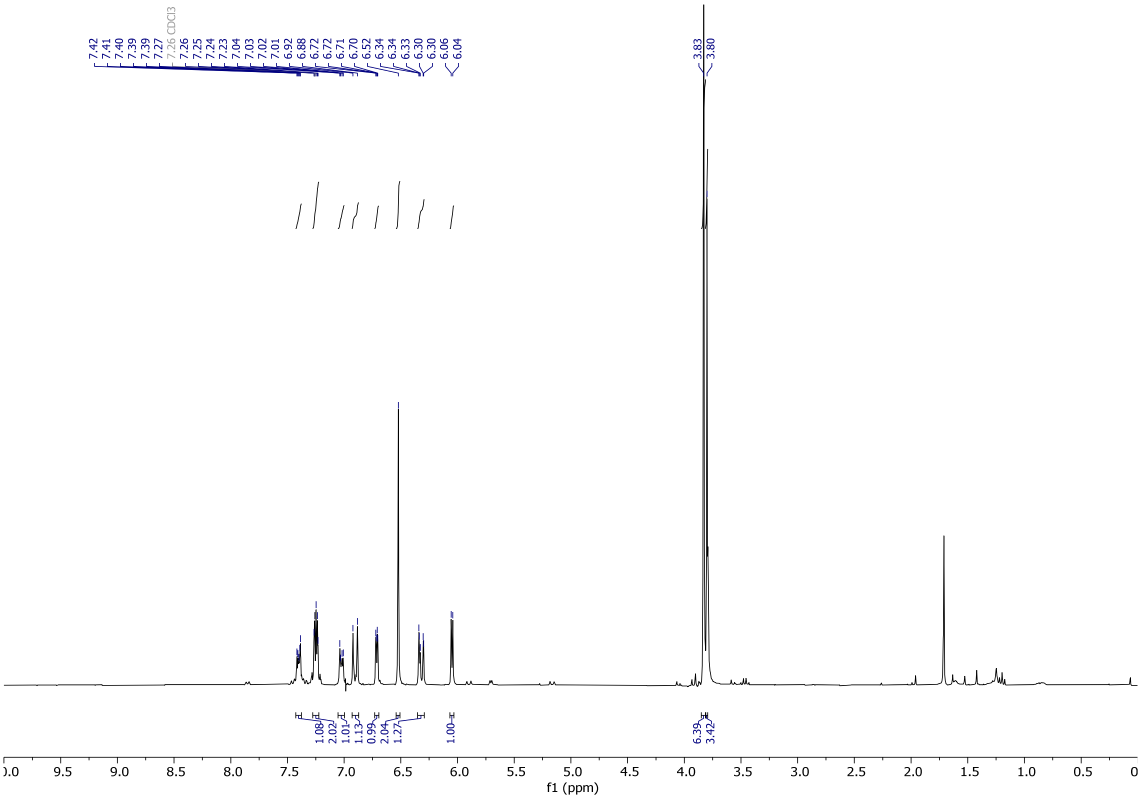

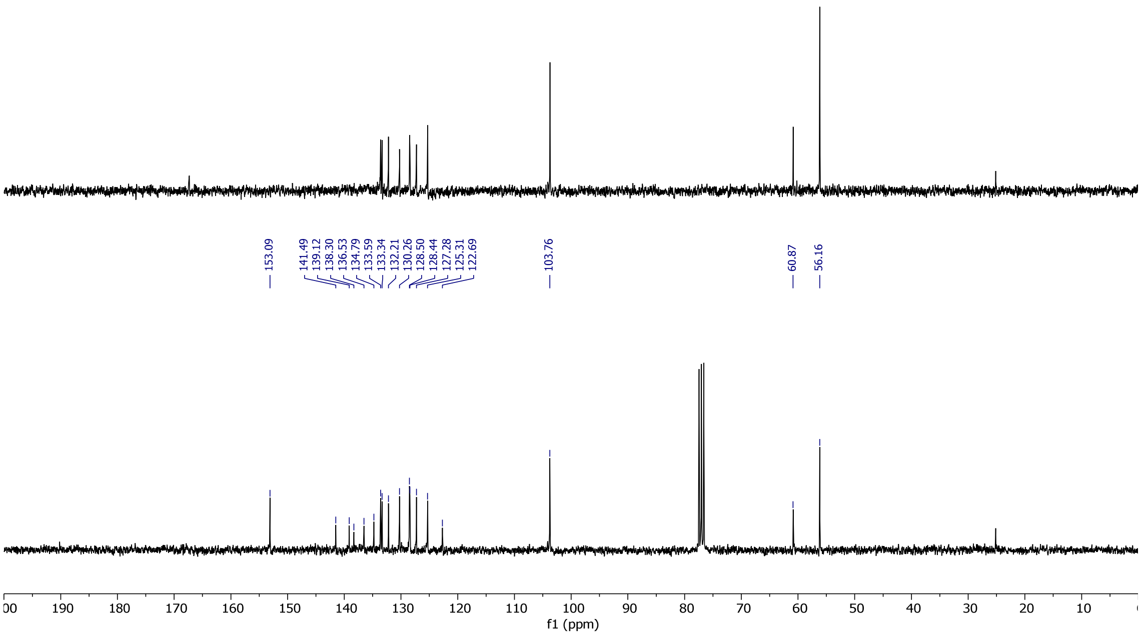

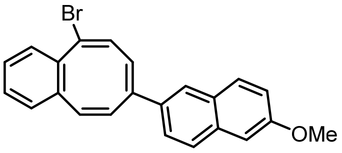

4c

^{1}H-NMR (500 Hz) and ^{13}C-NMR, DEPT (126 Hz) in $CDCl_3$

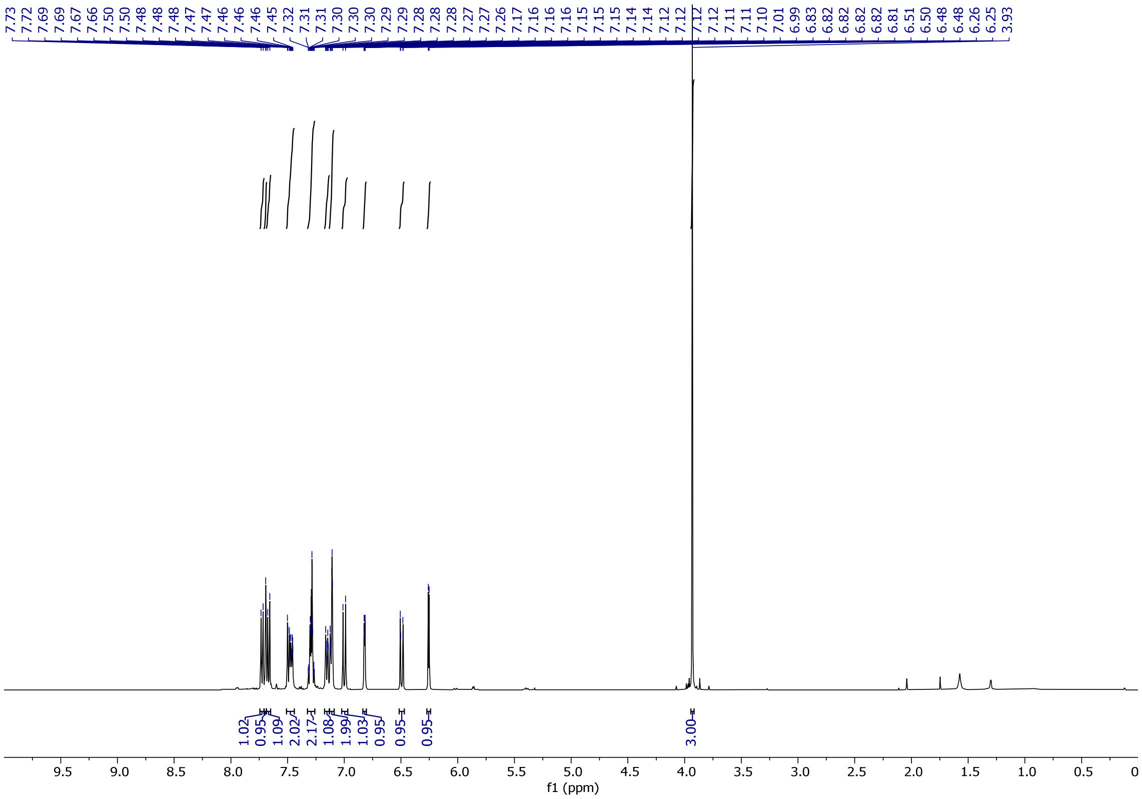

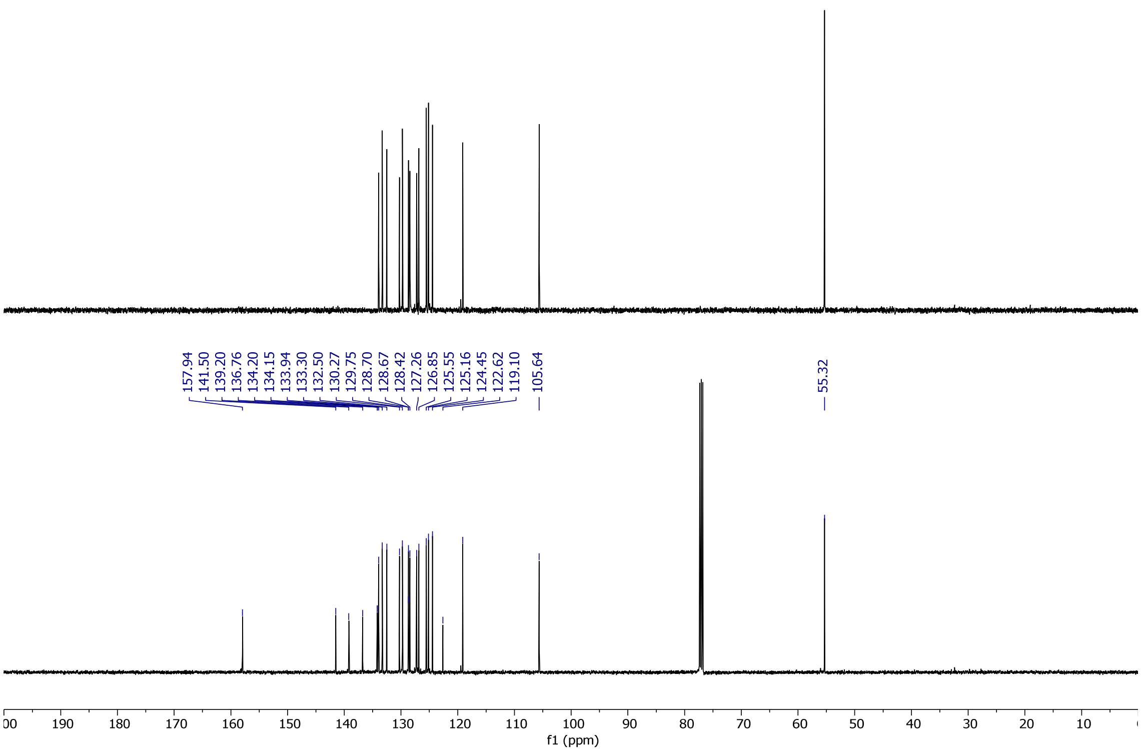

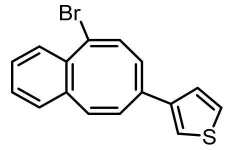

4d

^{1}H-NMR (500 Hz) and ^{13}C-NMR, DEPT (126 Hz) in $CDCl_3$

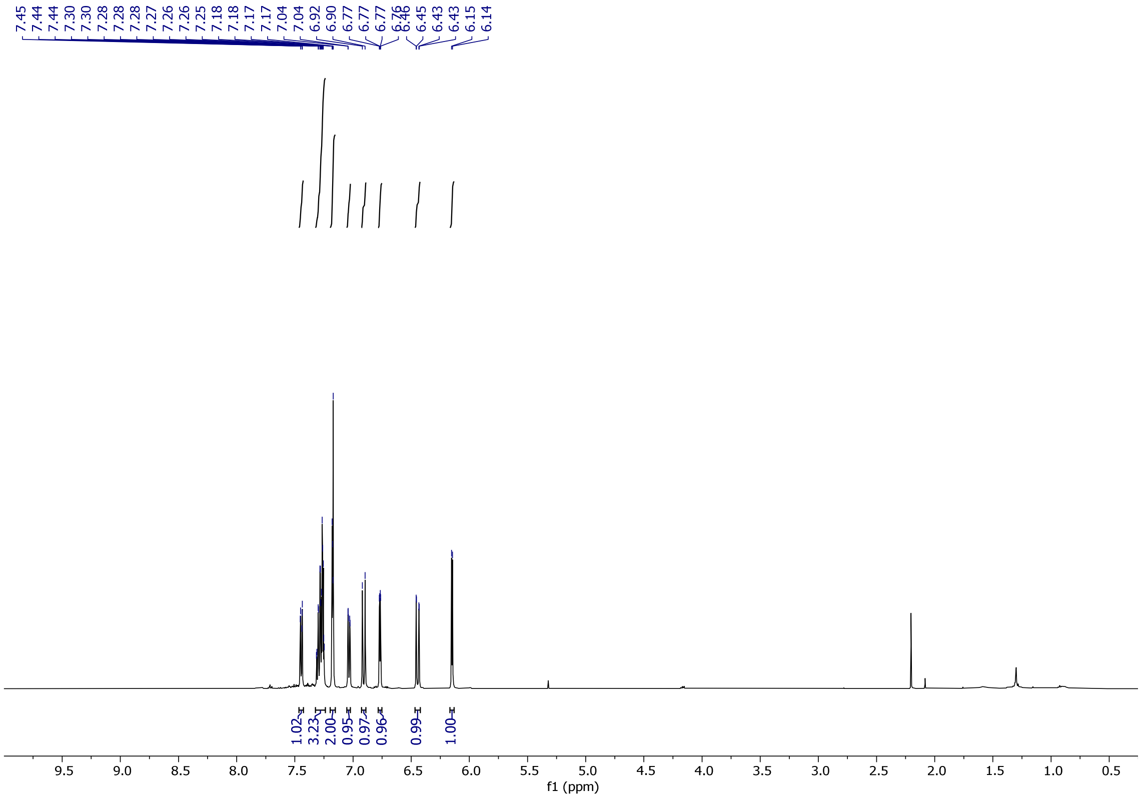

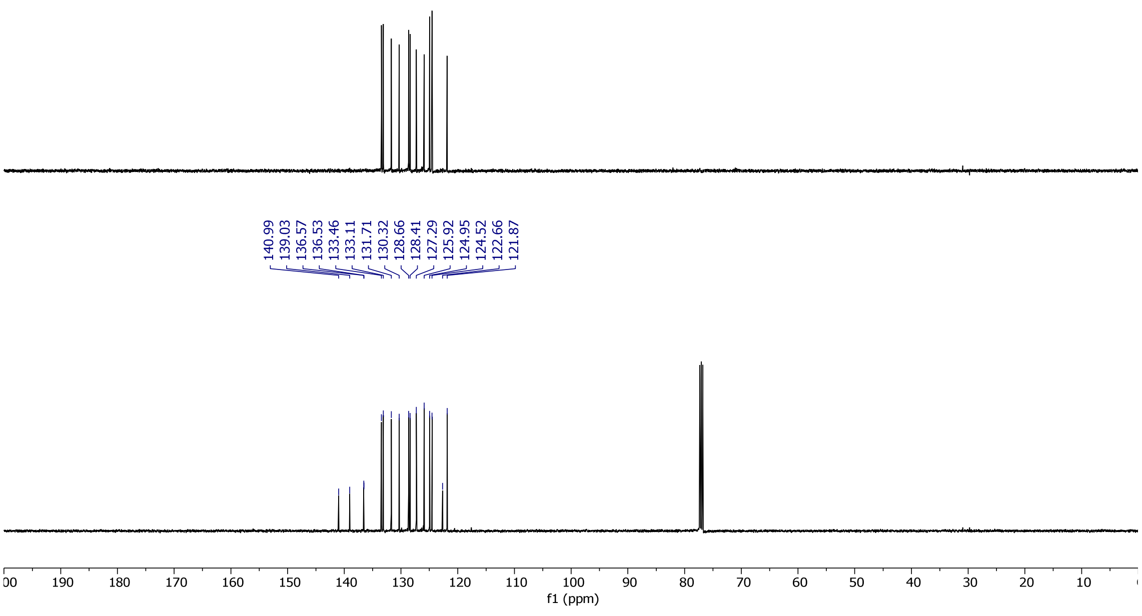

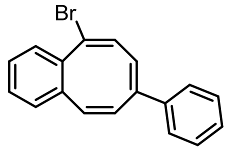

4e

^{1}H-NMR (500 Hz) and ^{13}C-NMR, DEPT (126 Hz) in $CDCl_3$

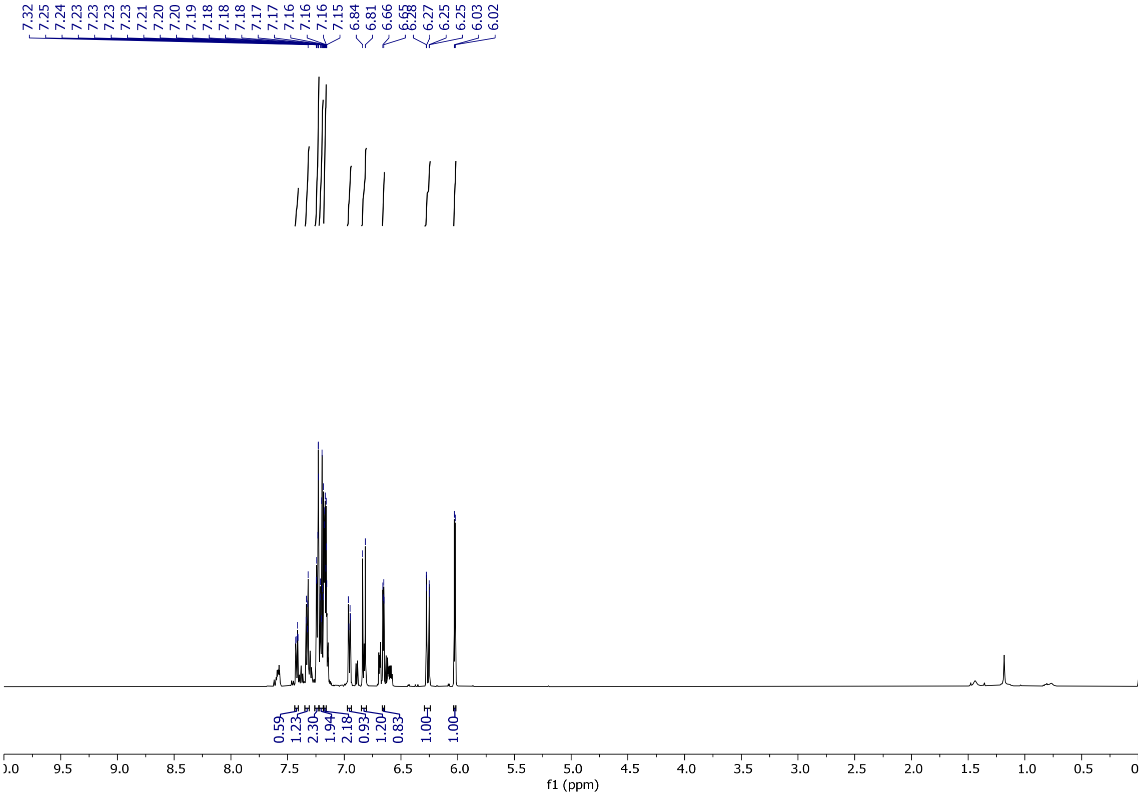

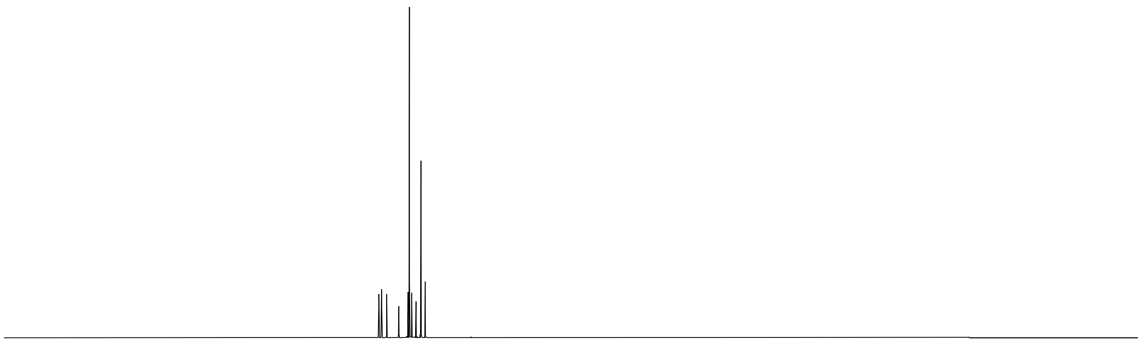

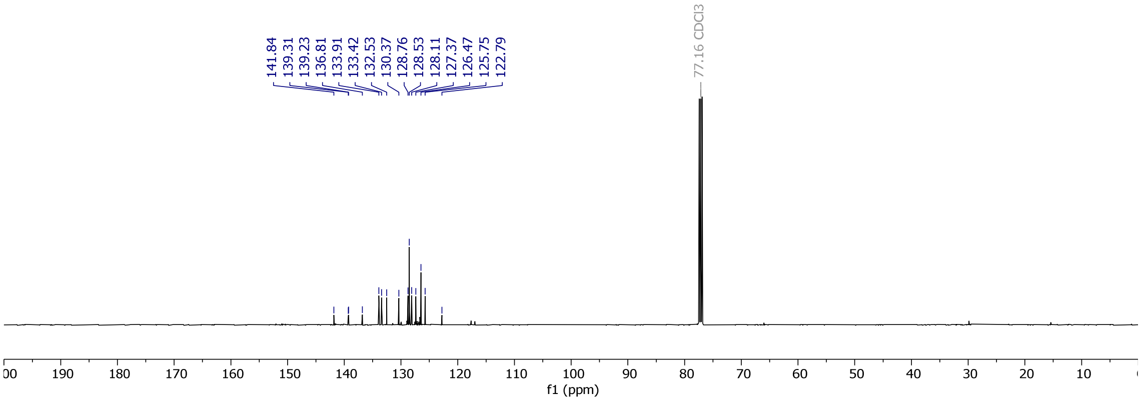

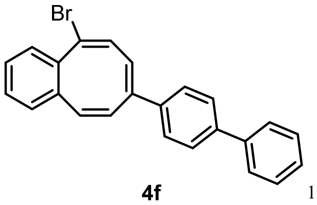

4f

^{1}H-NMR (300 Hz) and ^{13}C-NMR, DEPT (75 Hz) in $CDCl_3$

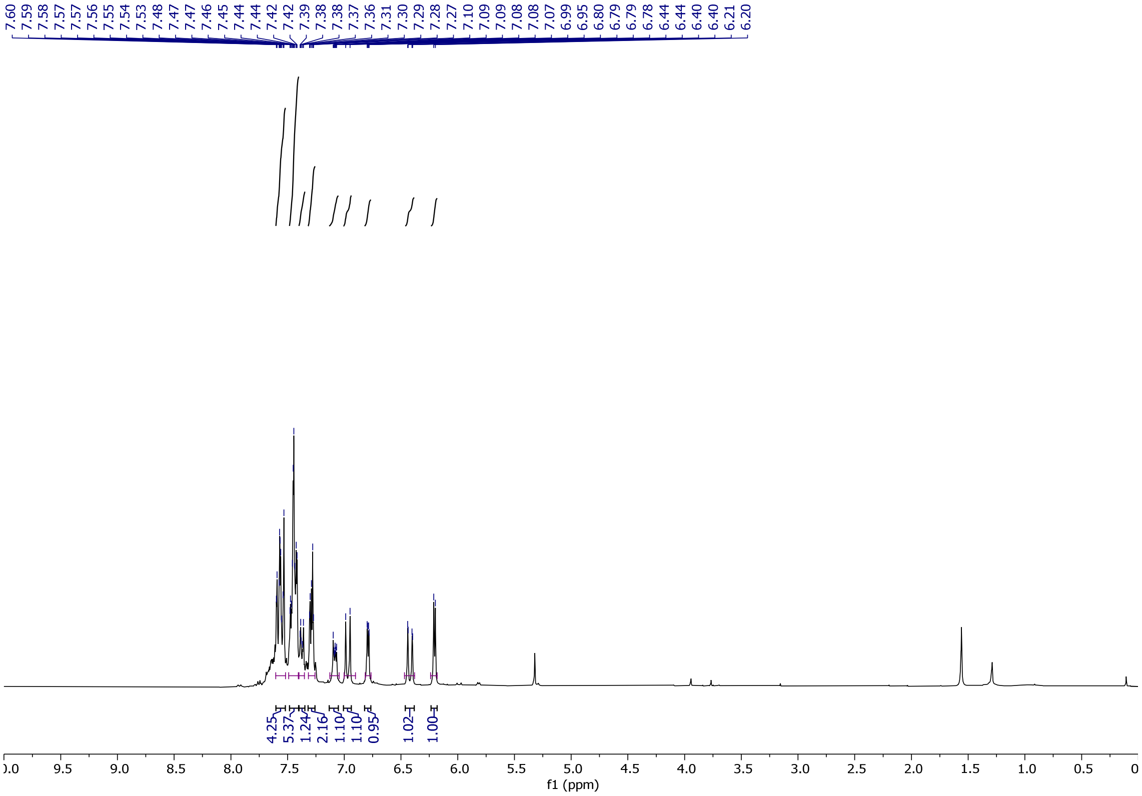

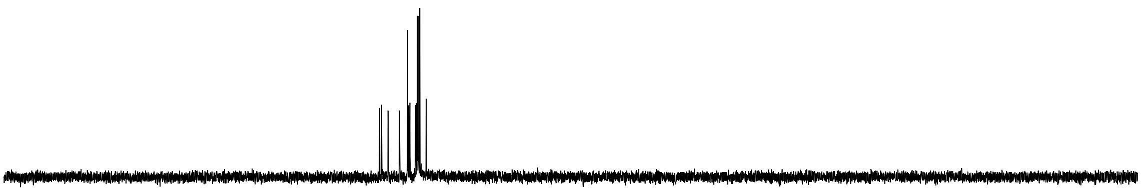

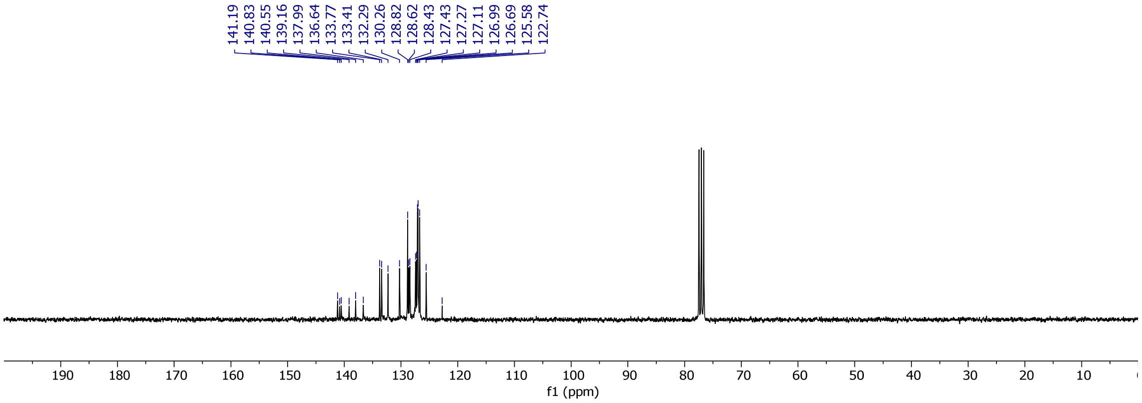

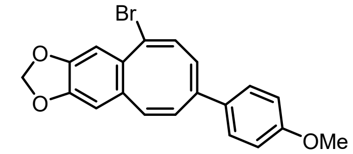

4g

^{1}H-NMR (300 Hz) and ^{13}C-NMR, DEPT (75 Hz) in $CDCl_3$

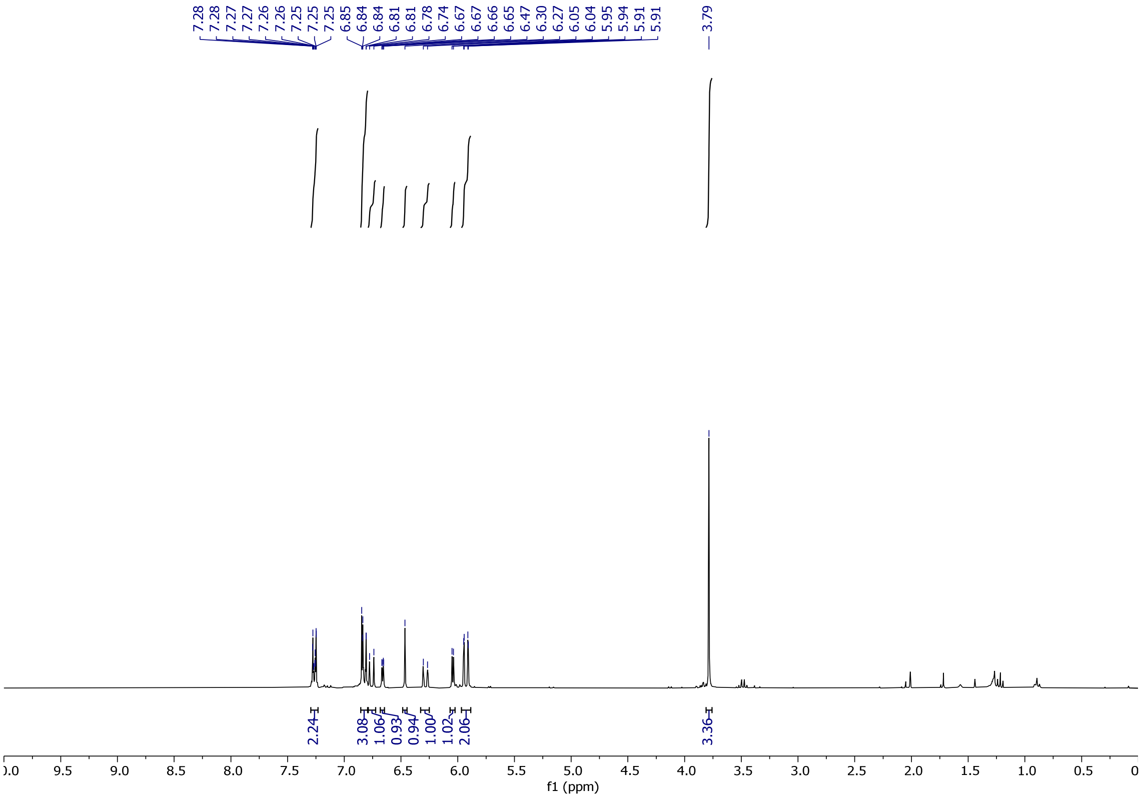

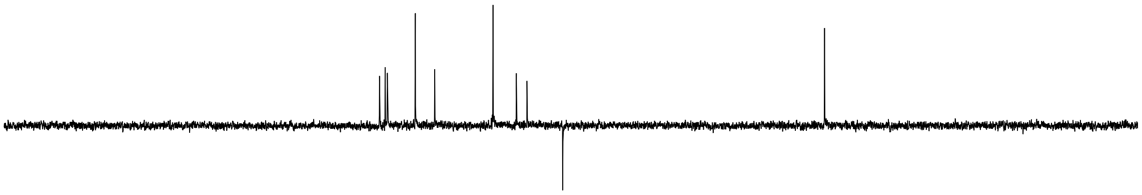

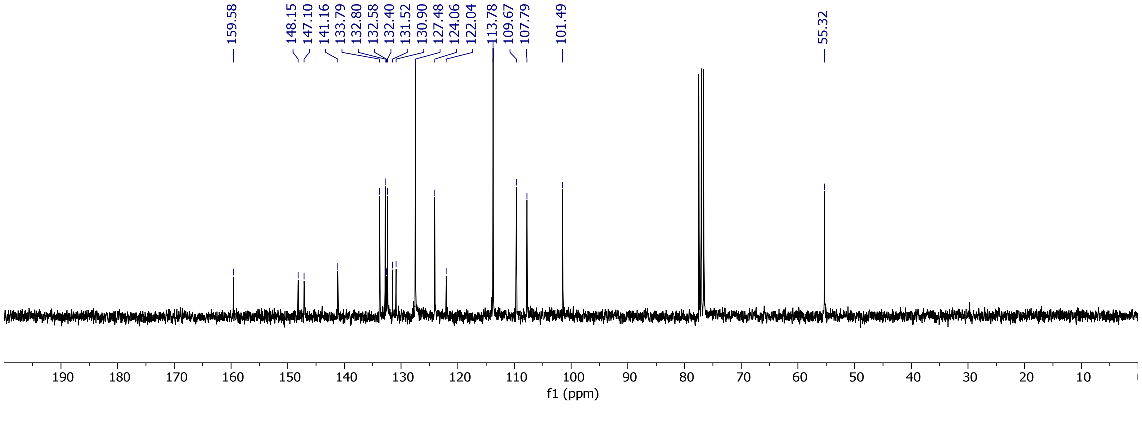

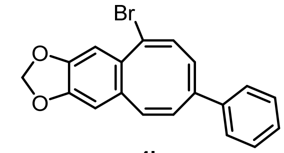

4h

^{1}H-NMR (300 Hz) and ^{13}C-NMR, DEPT (126 Hz) in $CDCl_3$

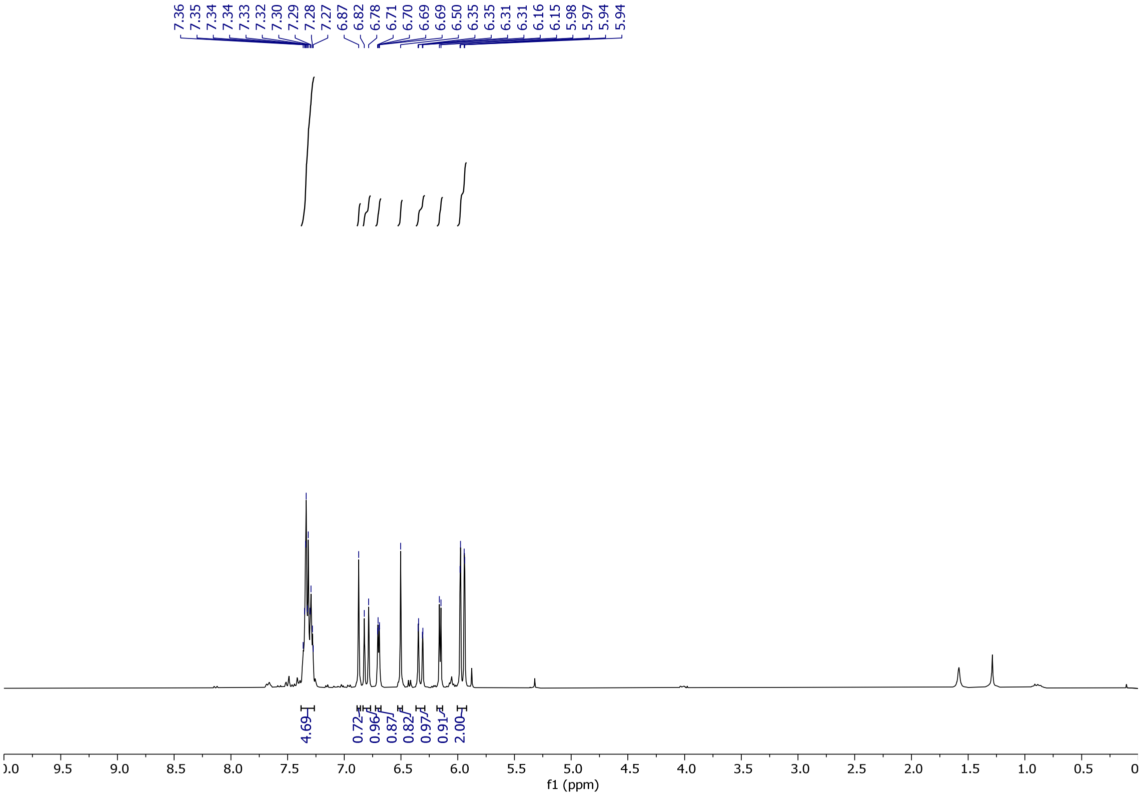

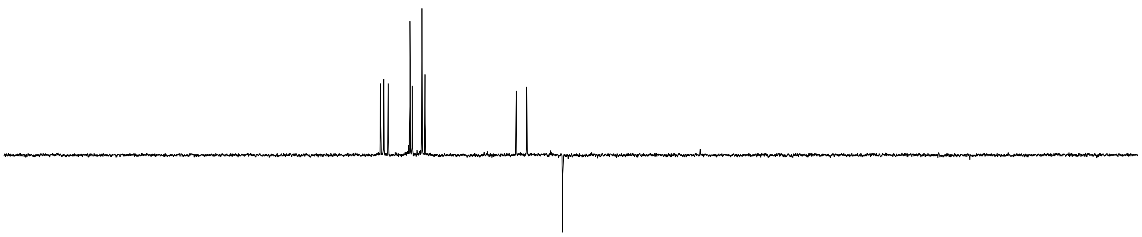

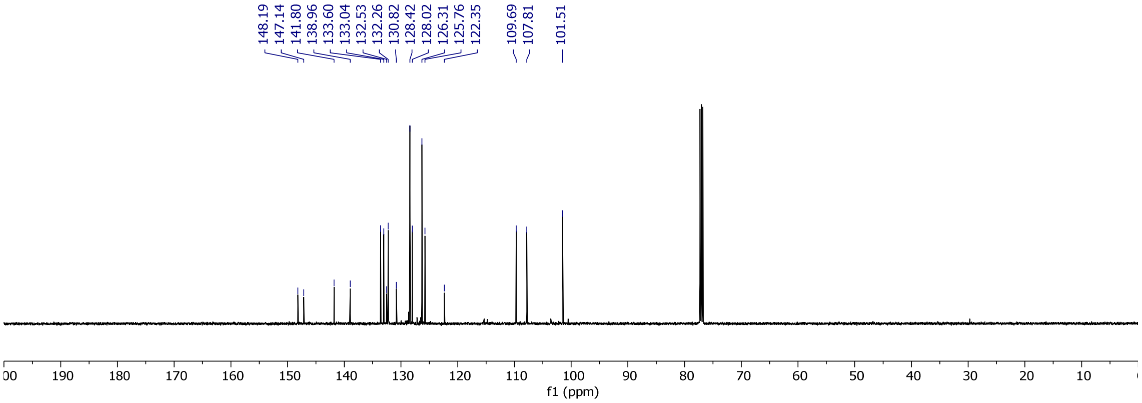

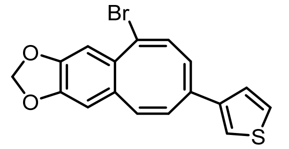

4i

^{1}H-NMR (500 Hz) and ^{13}C-NMR, DEPT (126 Hz) in $CDCl_3$

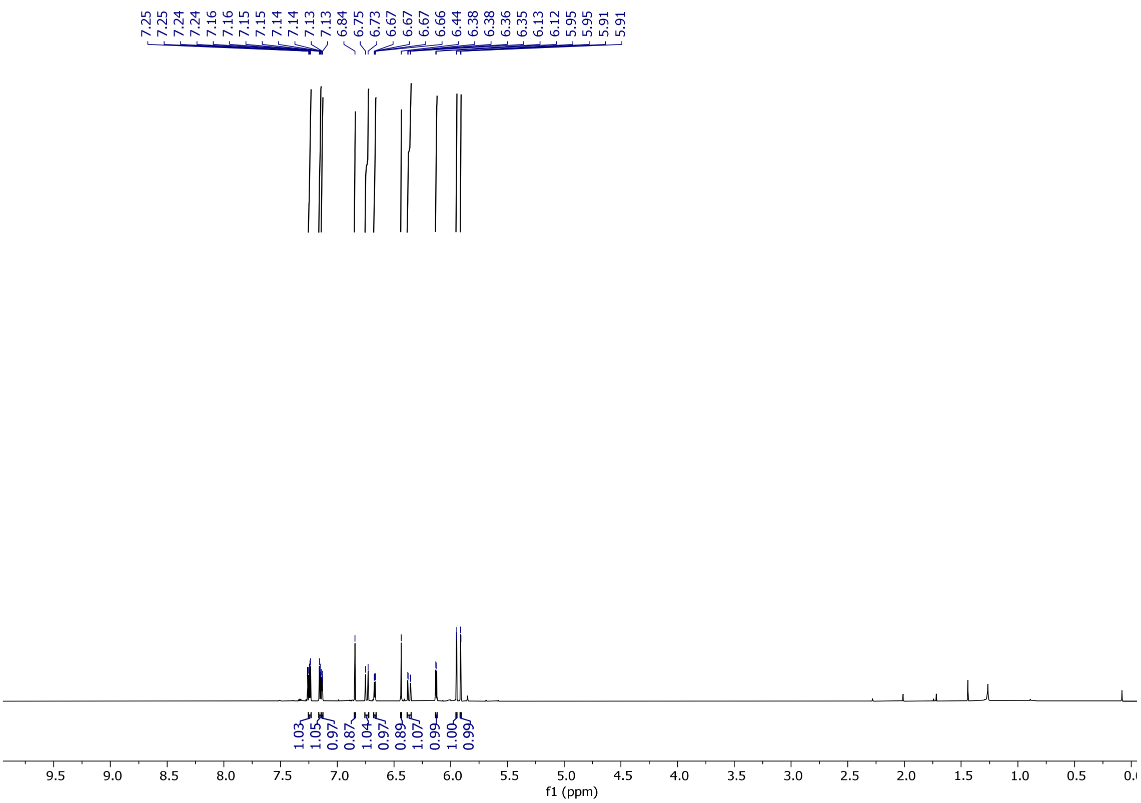

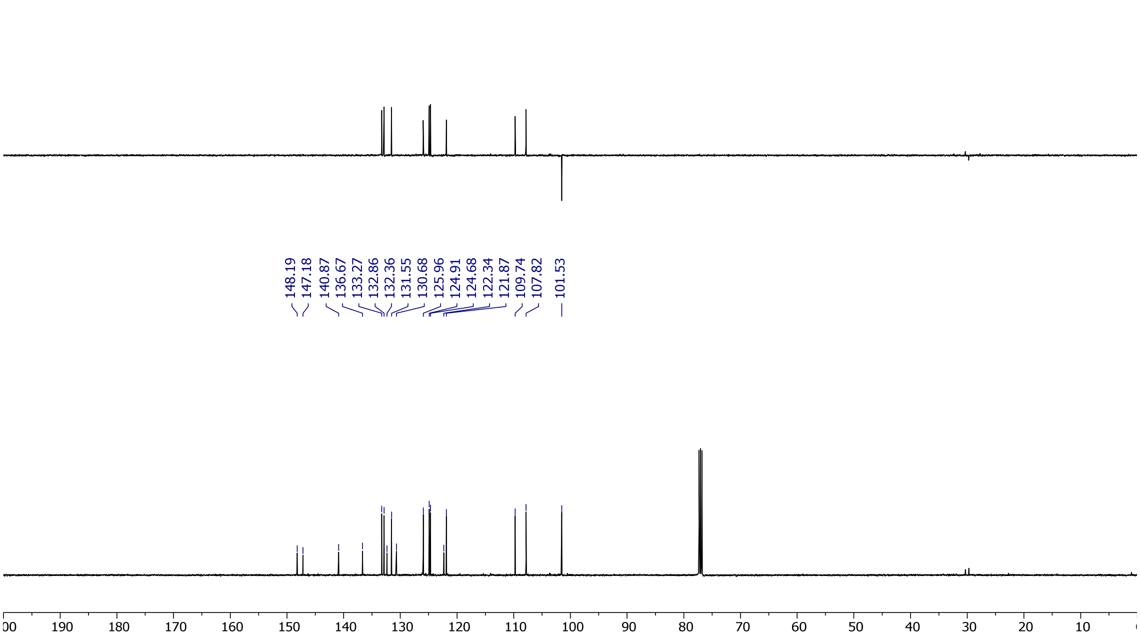

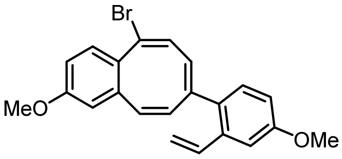

4j

^{1}H-NMR (500 Hz) and ^{13}C-NMR, DEPT (126 Hz) in $CDCl_3$

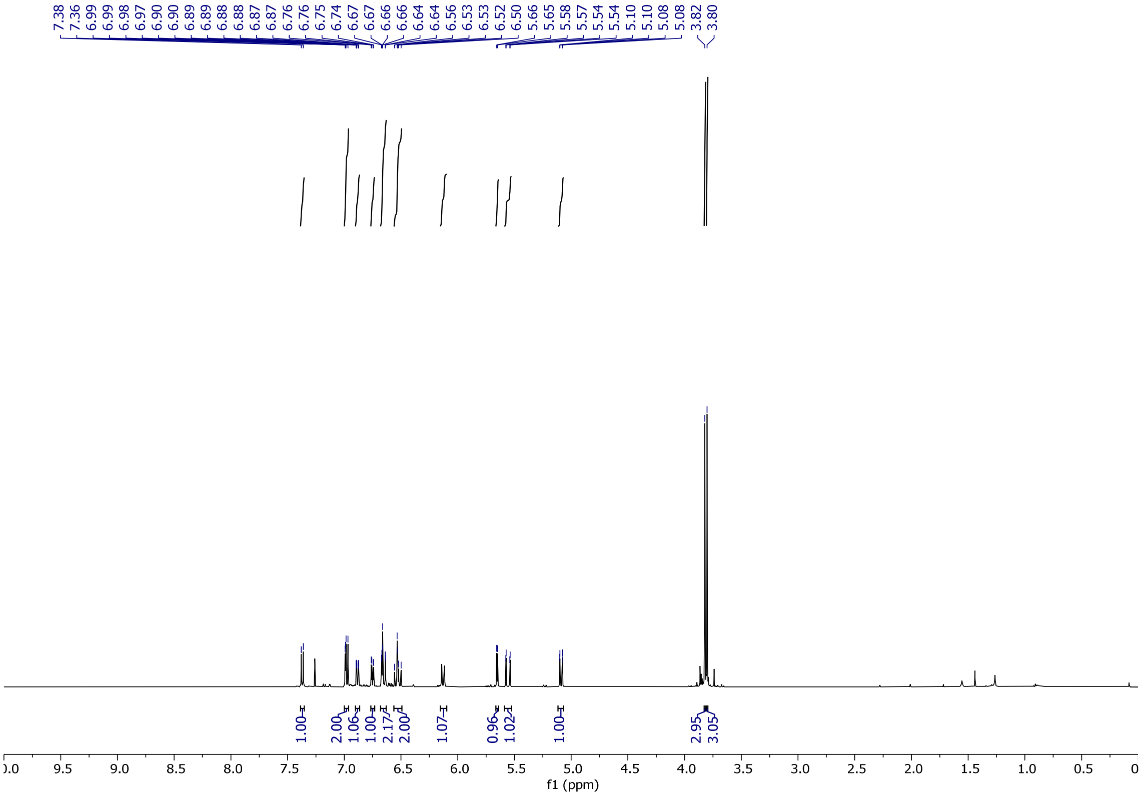

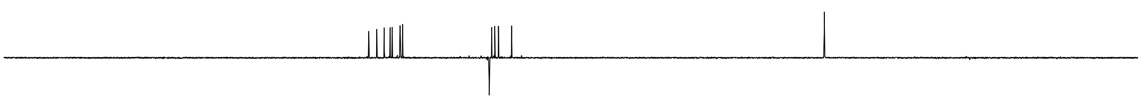

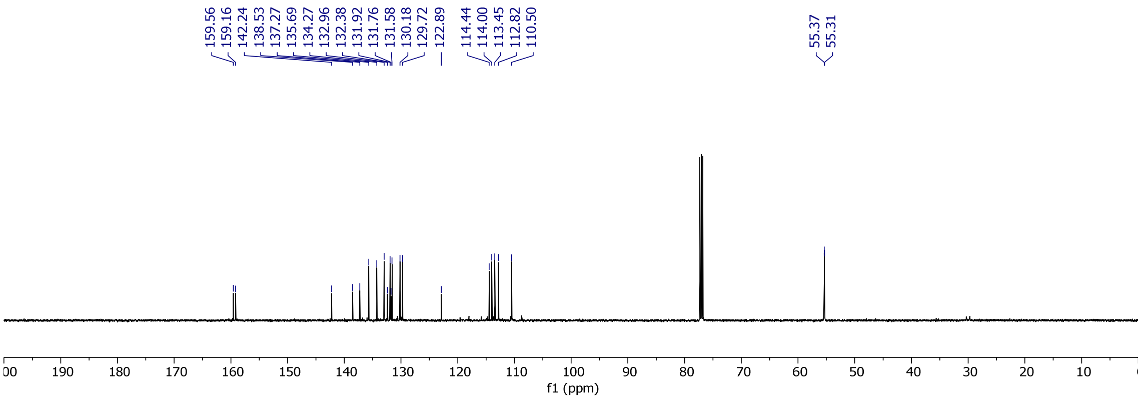

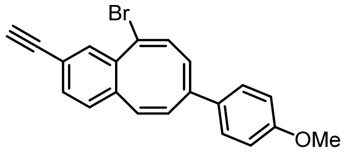

4k

^{1}H-NMR (300 Hz) and ^{13}C-NMR, DEPT (75 Hz) in $CDCl_3$

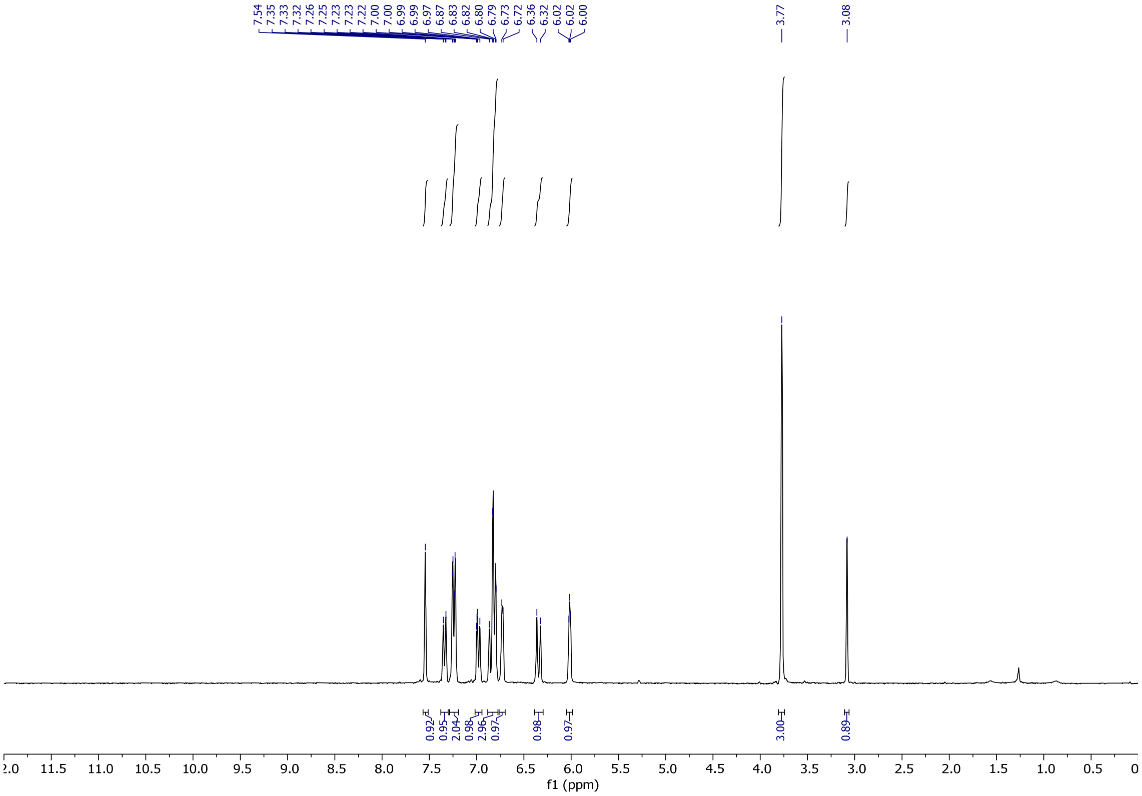

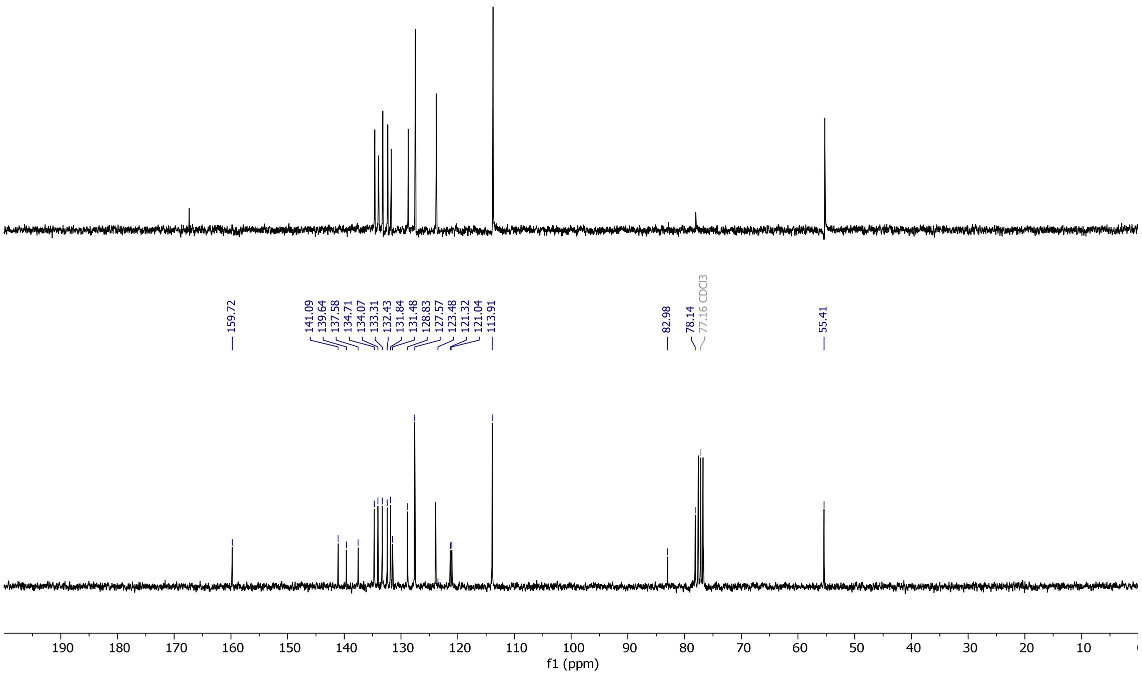

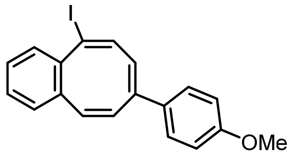

4a'

^{1}H-NMR (500 Hz) and ^{13}C-NMR, DEPT (126 Hz) in $CDCl_3$

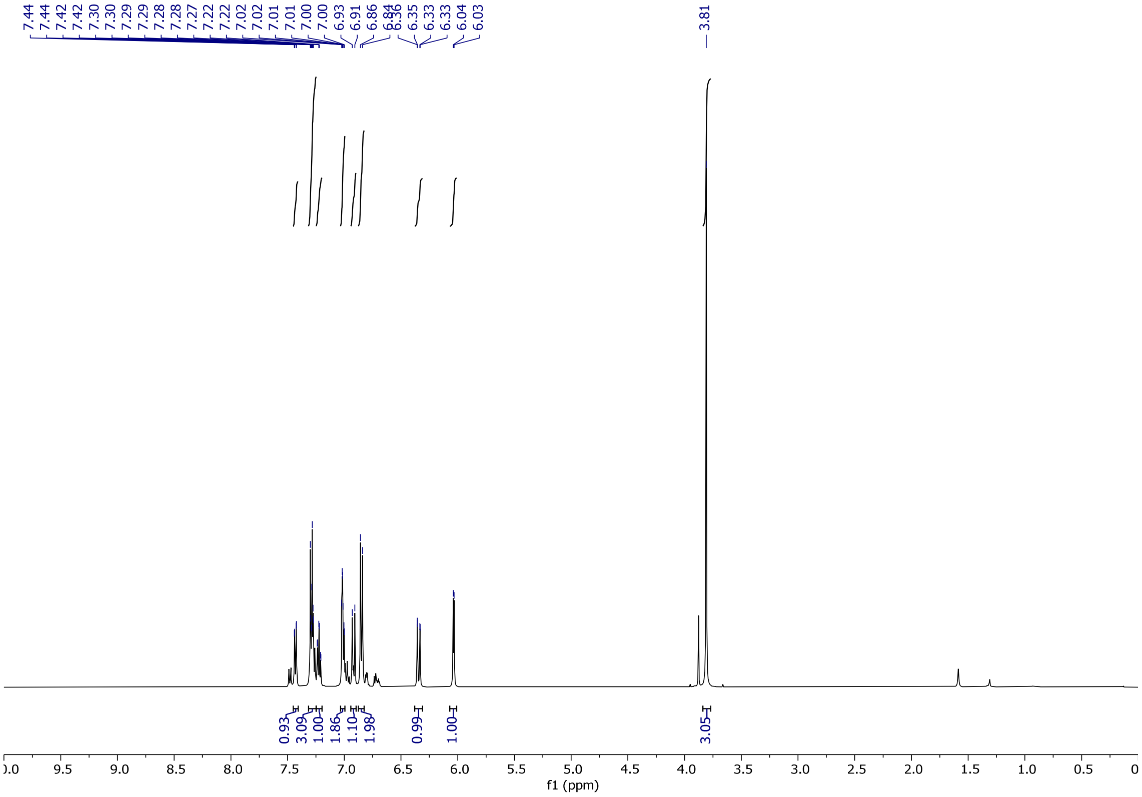

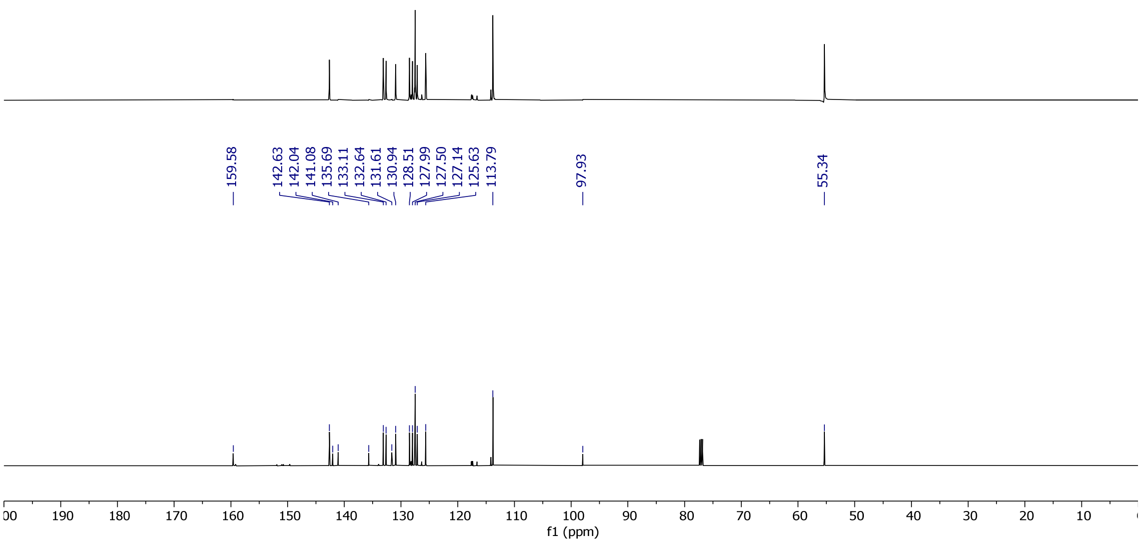

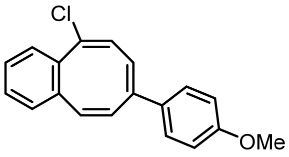

4a"

^{1}H-NMR (500 Hz) and ^{13}C-NMR, DEPT (126 Hz) in $CDCl_3$

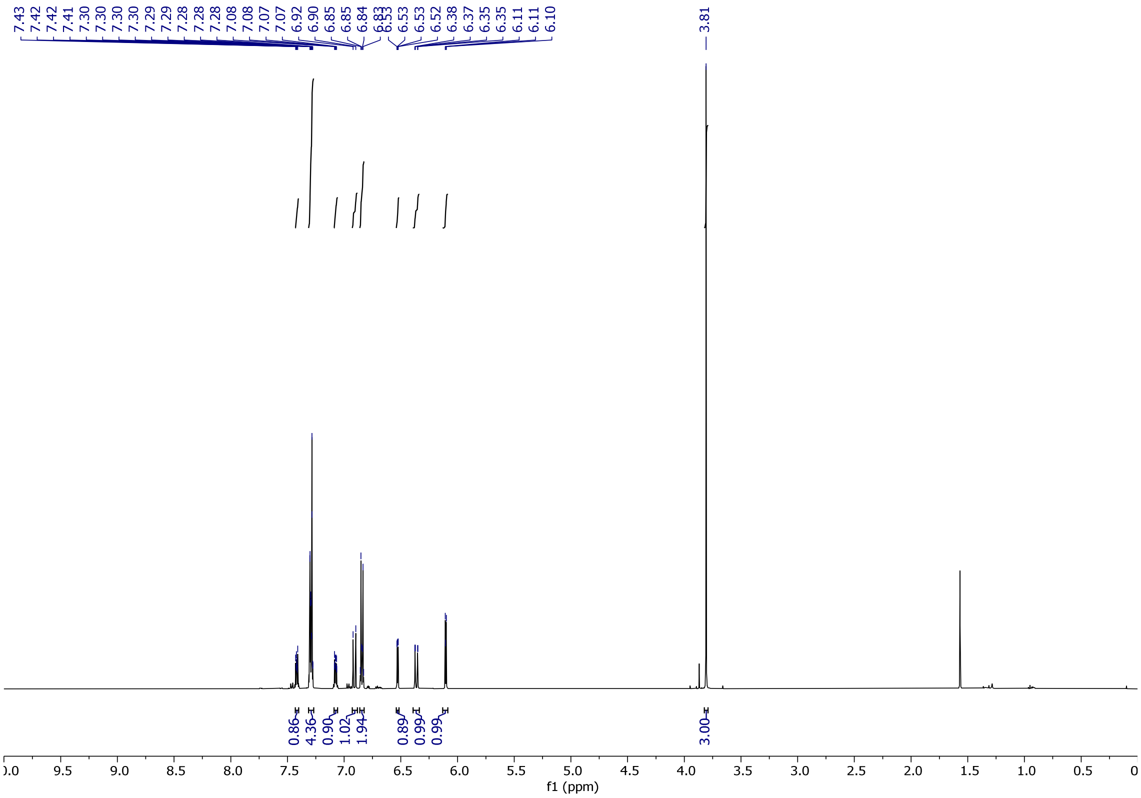

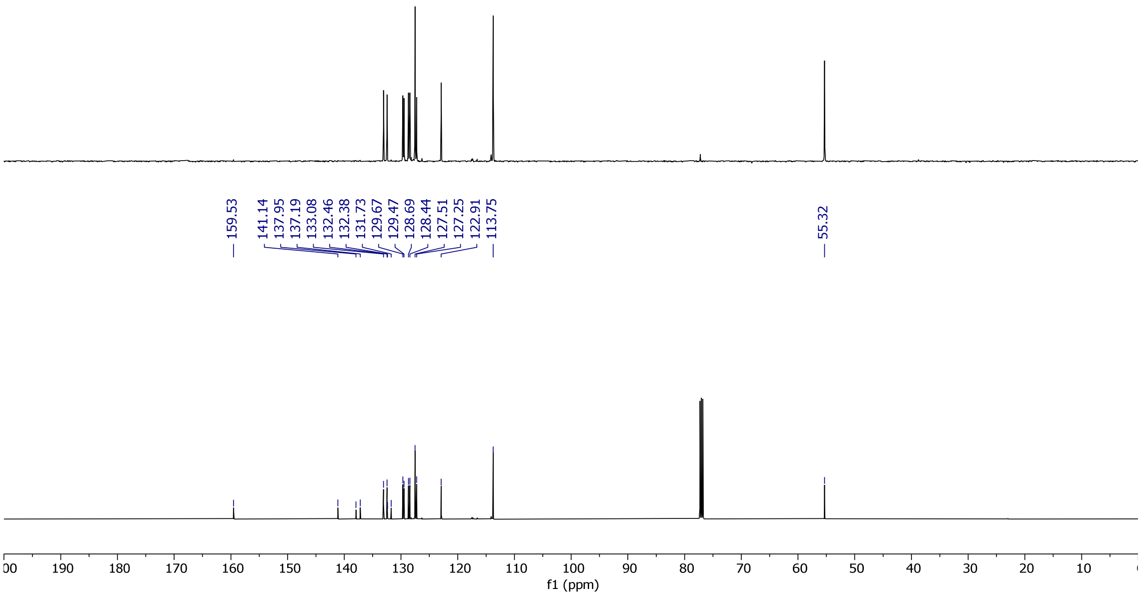

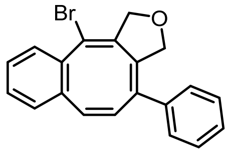

6a

^{1}H-NMR (500 Hz) and ^{13}C-NMR, DEPT (126 Hz) in $CDCl_3$

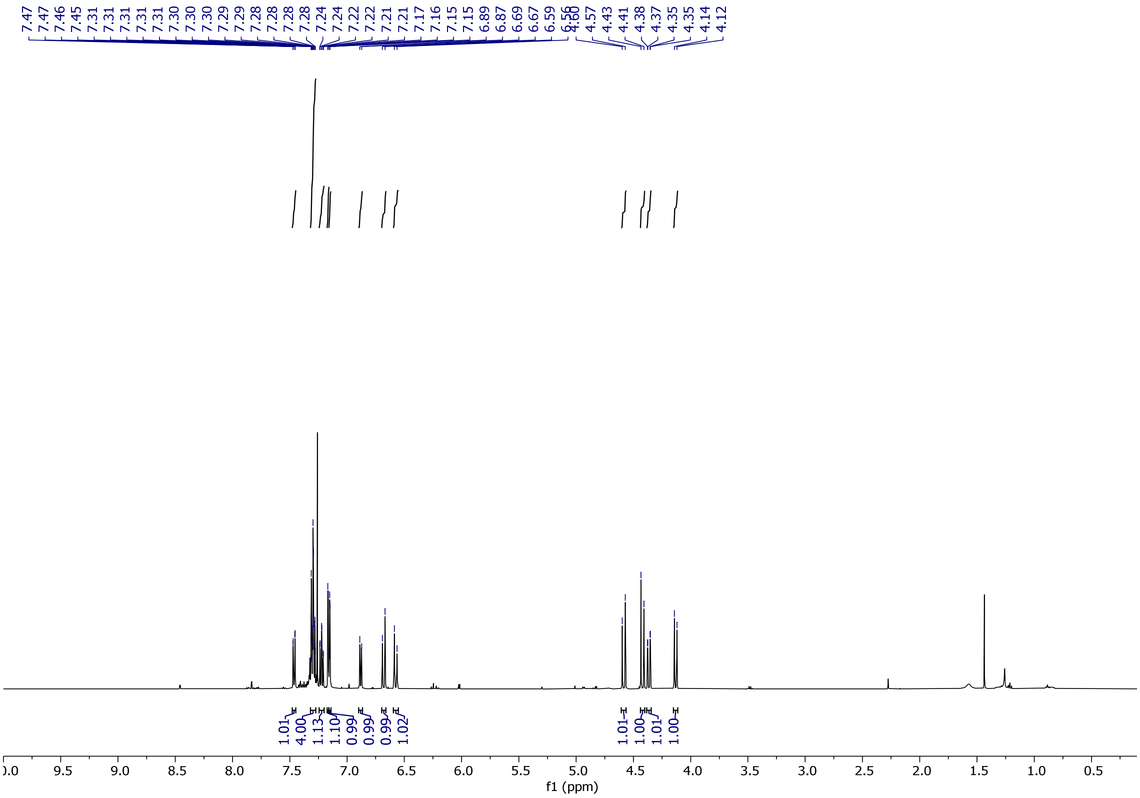

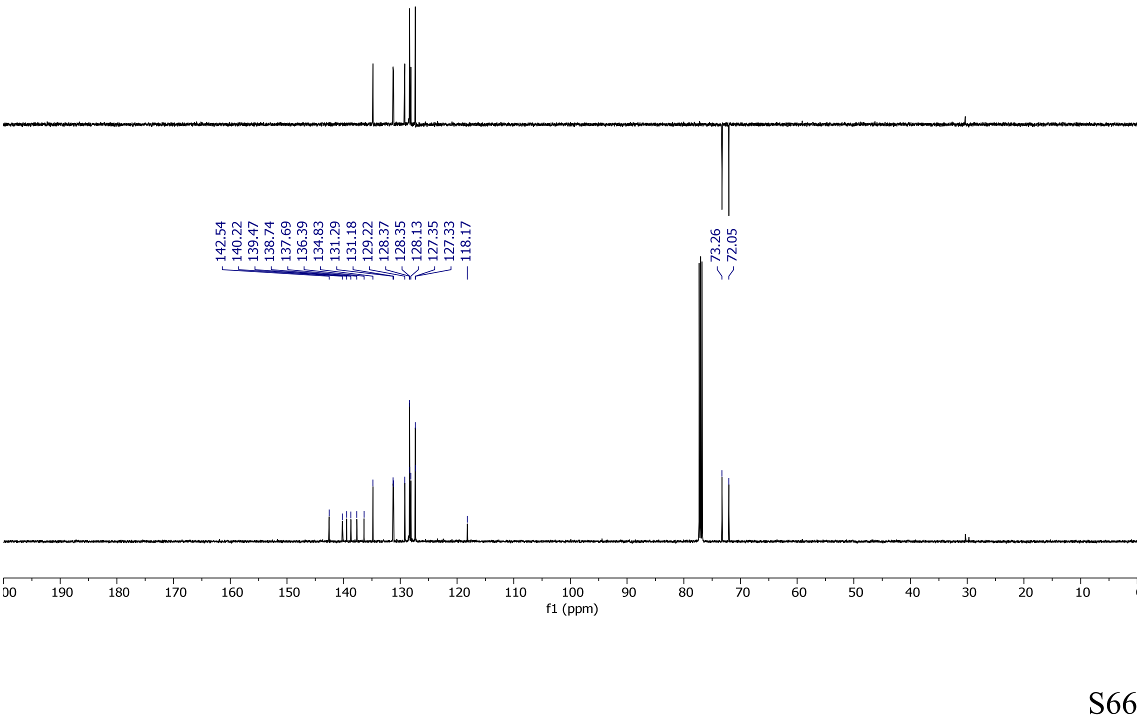

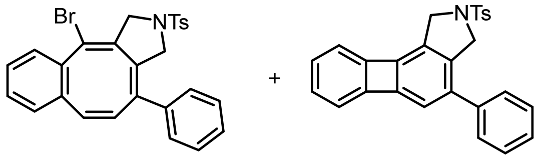

6b 6b*

^{1}H-NMR (300 Hz) and ^{13}C-NMR, DEPT (126 Hz) in $CDCl_3$

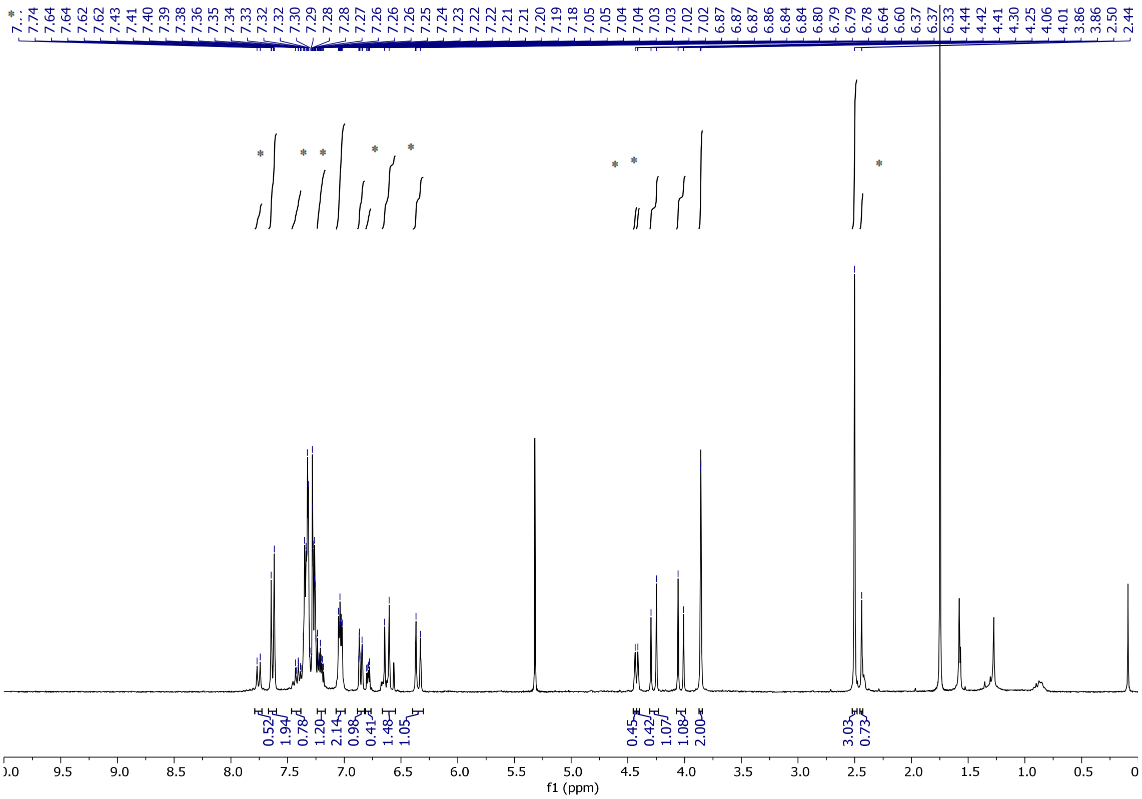

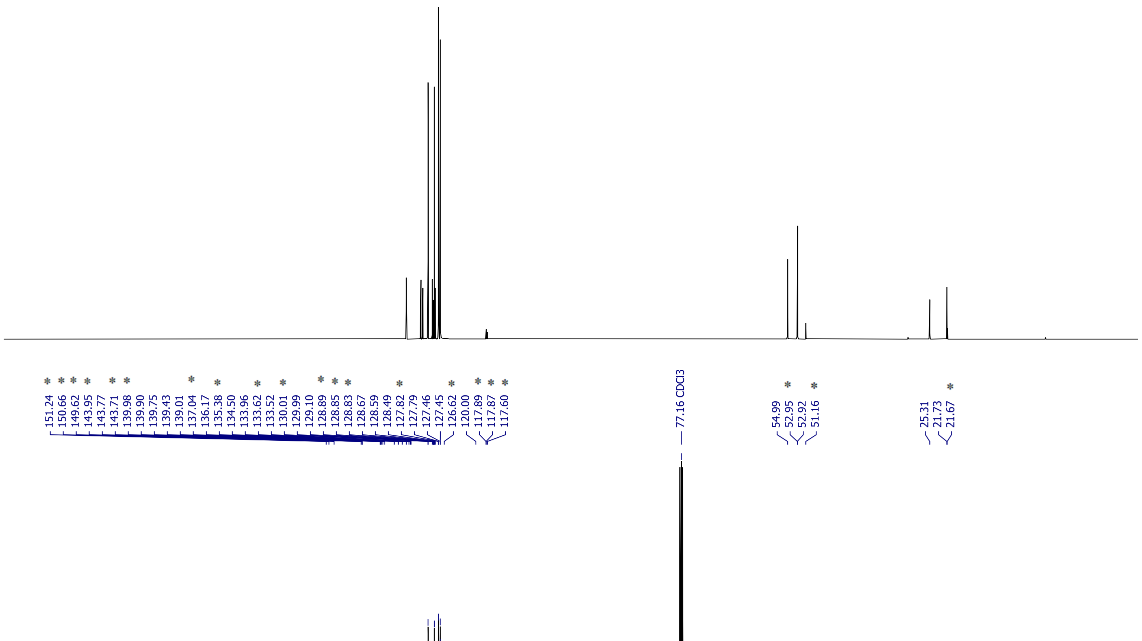

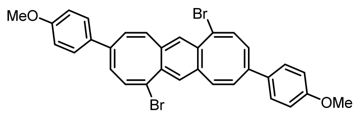

9

^{1}H-NMR (500 Hz) and ^{13}C-NMR, DEPT (126 Hz) in 1,1,2,2-tetrachloroethane-*d*2 at 25ºC

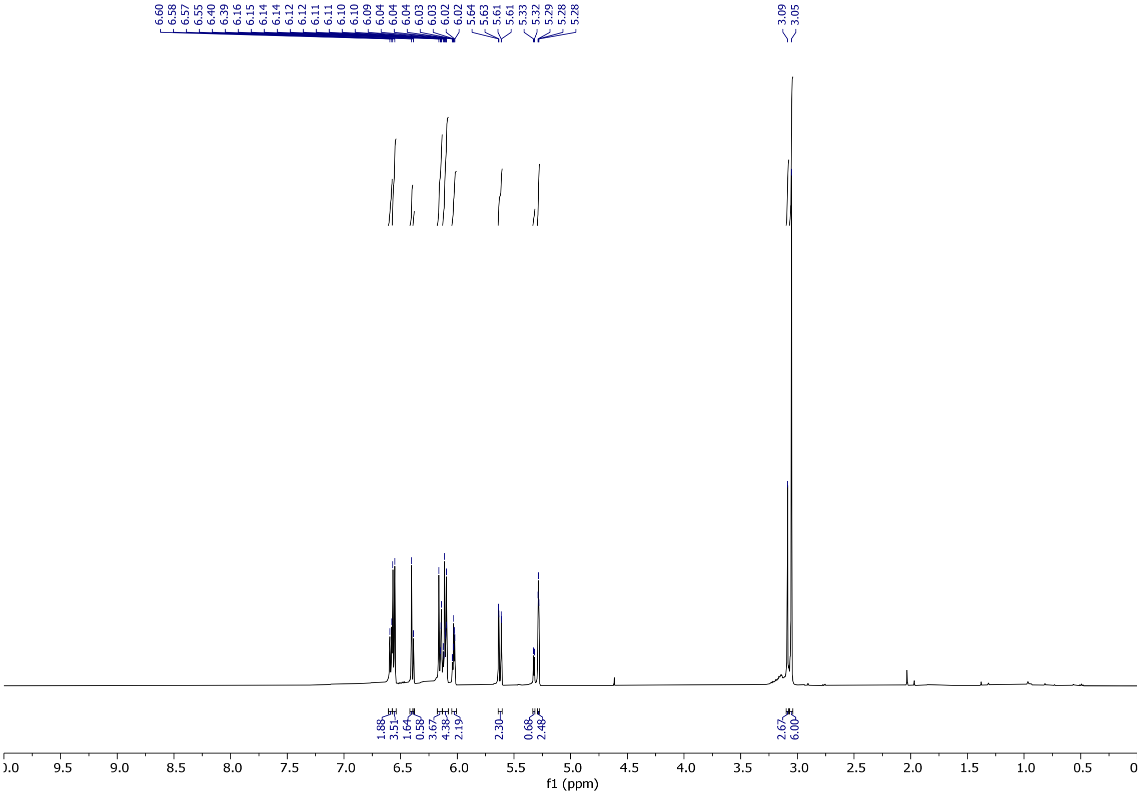

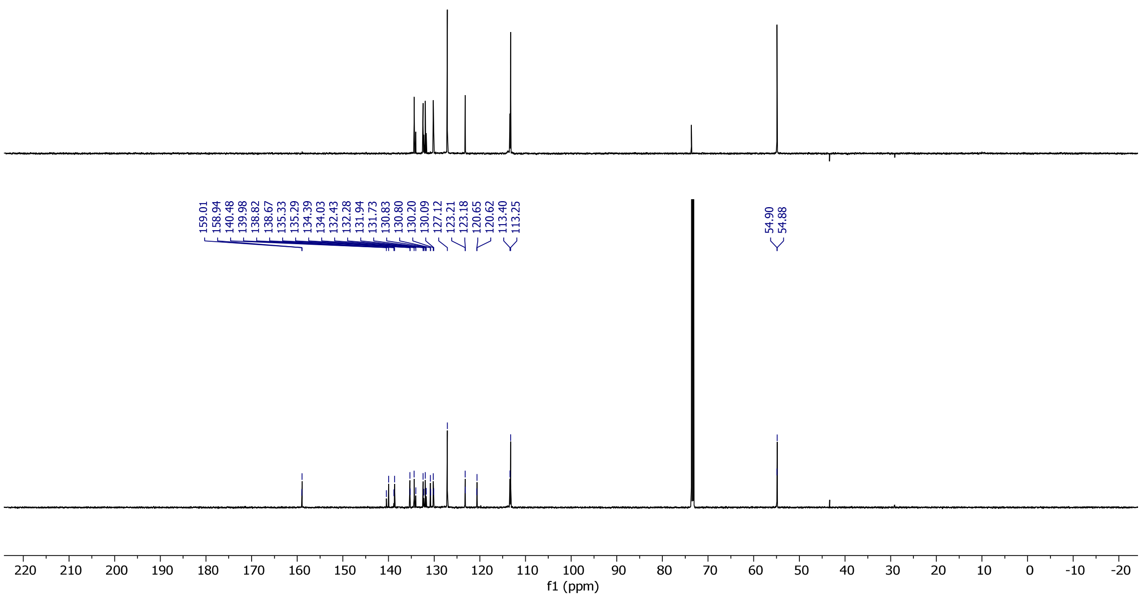

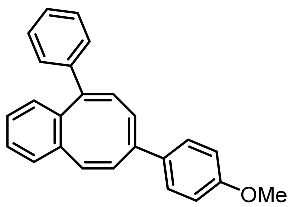

10

^{1}H-NMR (300 Hz) and ^{13}C-NMR, DEPT (75 Hz) in $CDCl_3$

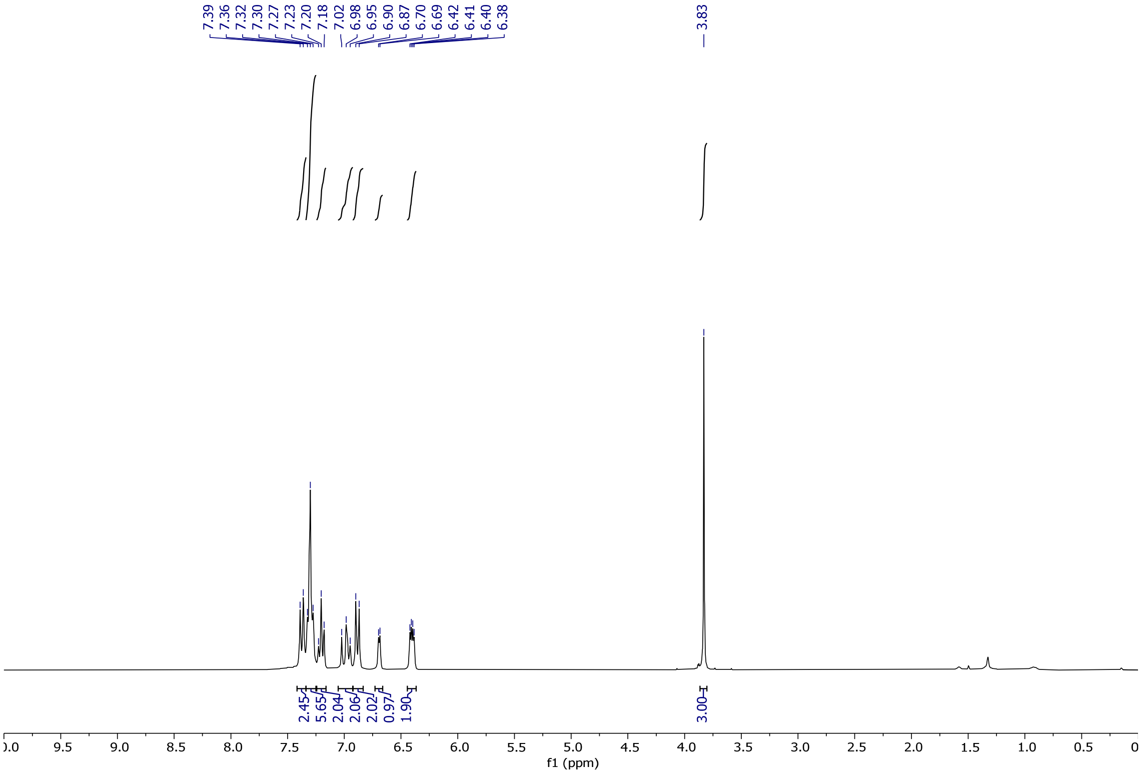

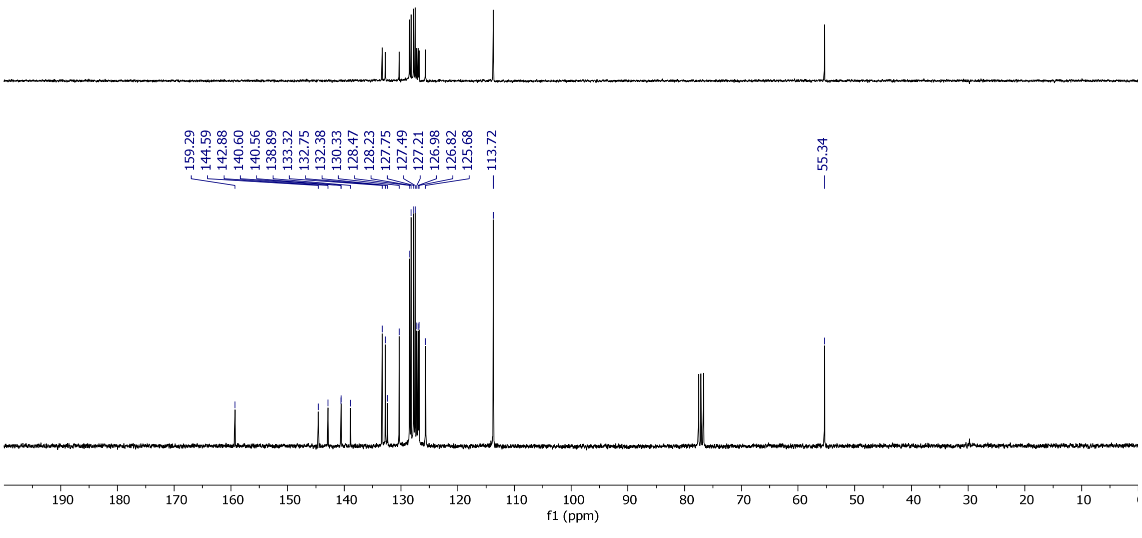

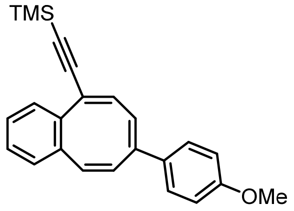

11a

^{1}H-NMR (500 Hz) and ^{13}C-NMR, DEPT (126 Hz) in $CDCl_3$

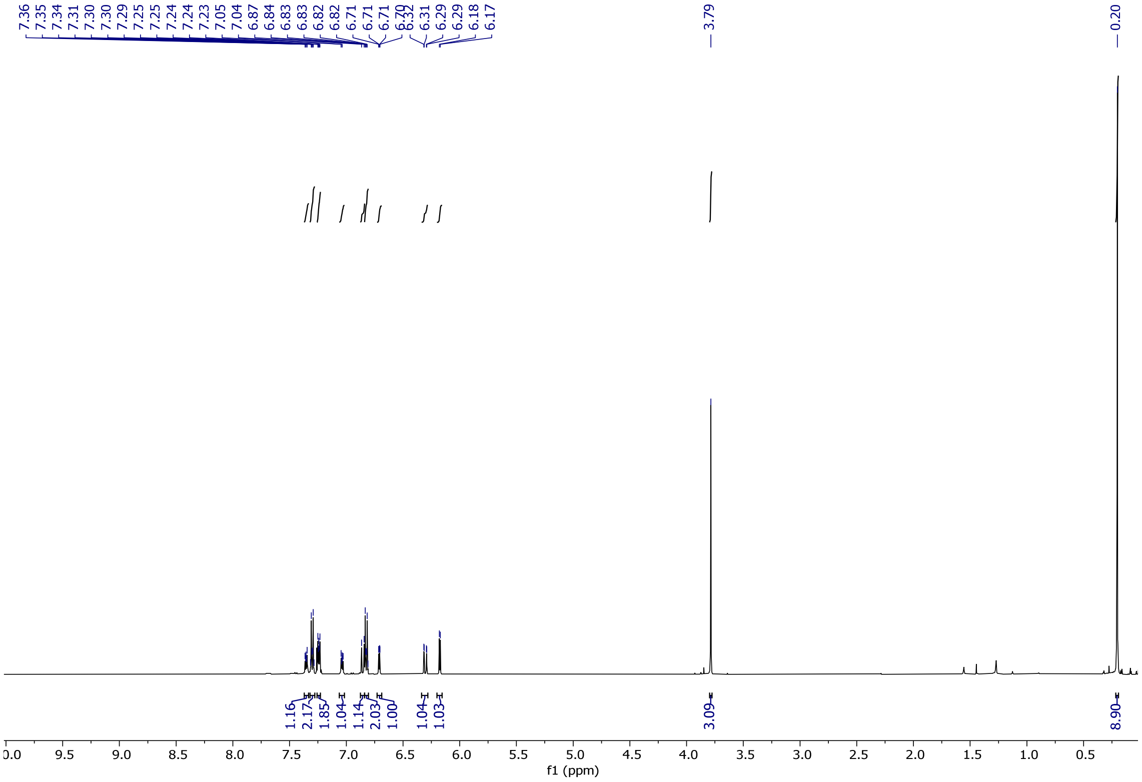

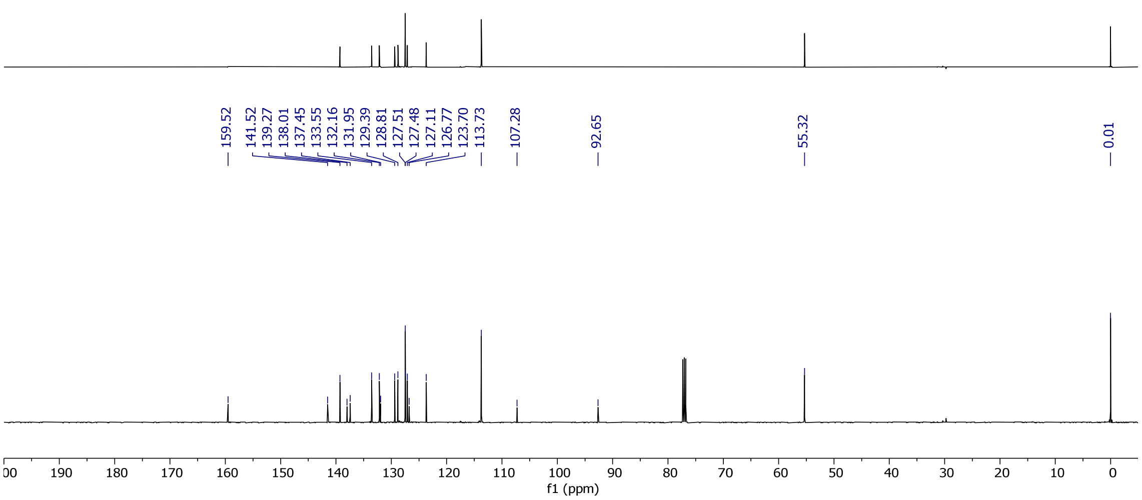

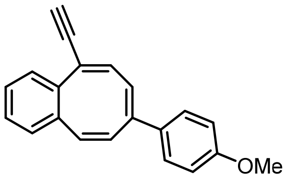

11a'

^{1}H-NMR (500 Hz) and ^{13}C-NMR, DEPT (126 Hz) in $CDCl_3$

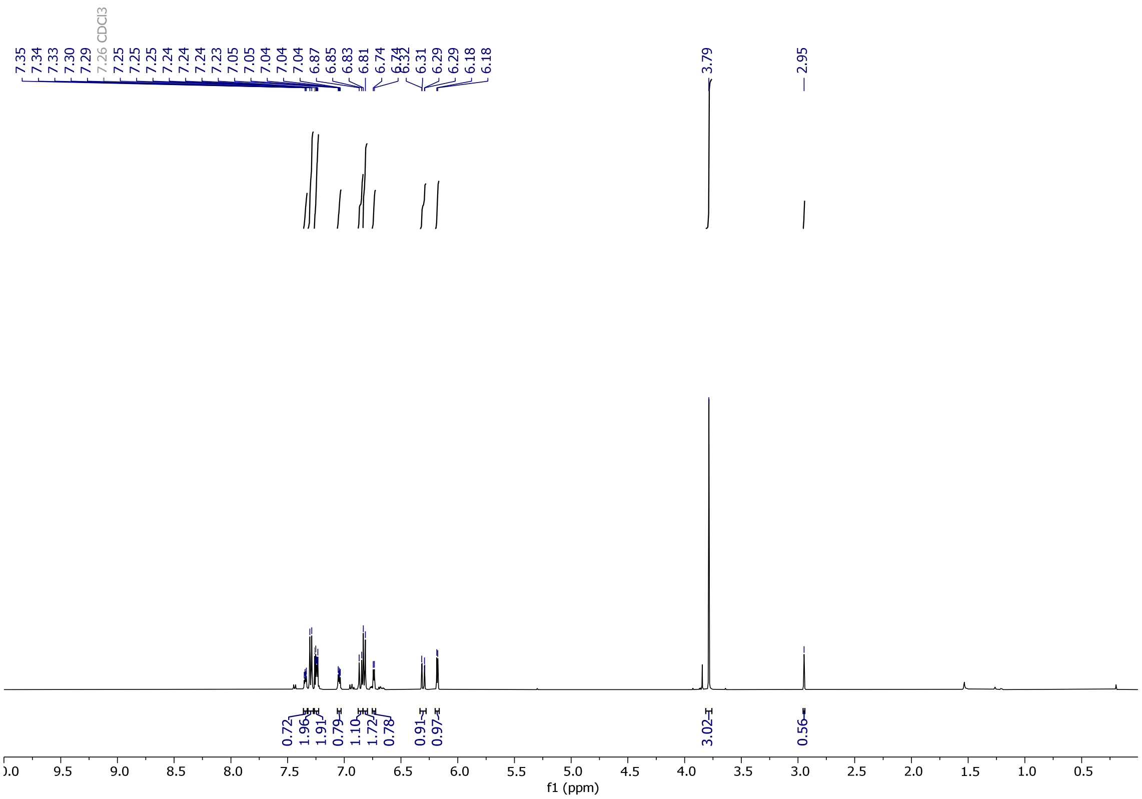

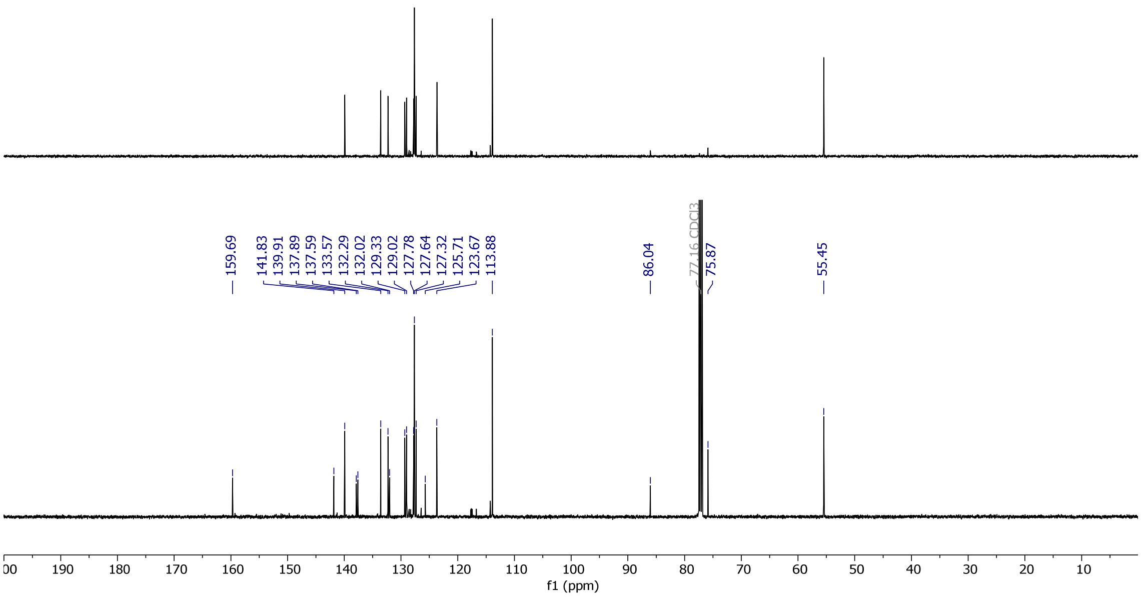

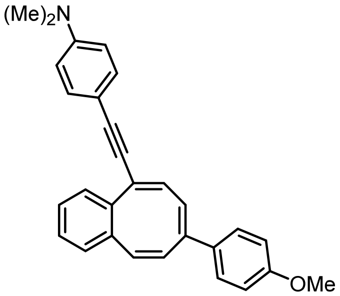

11b

^{1}H-NMR (500 Hz) and ^{13}C-NMR, DEPT (126 Hz) in $CDCl_3$

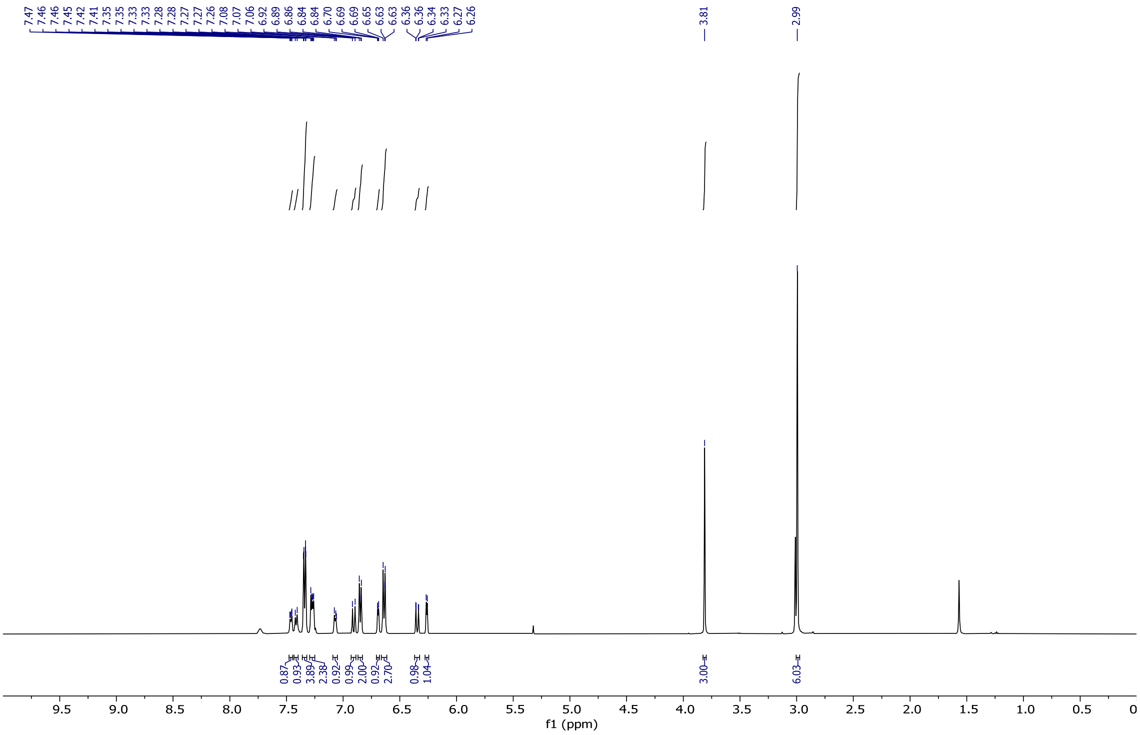

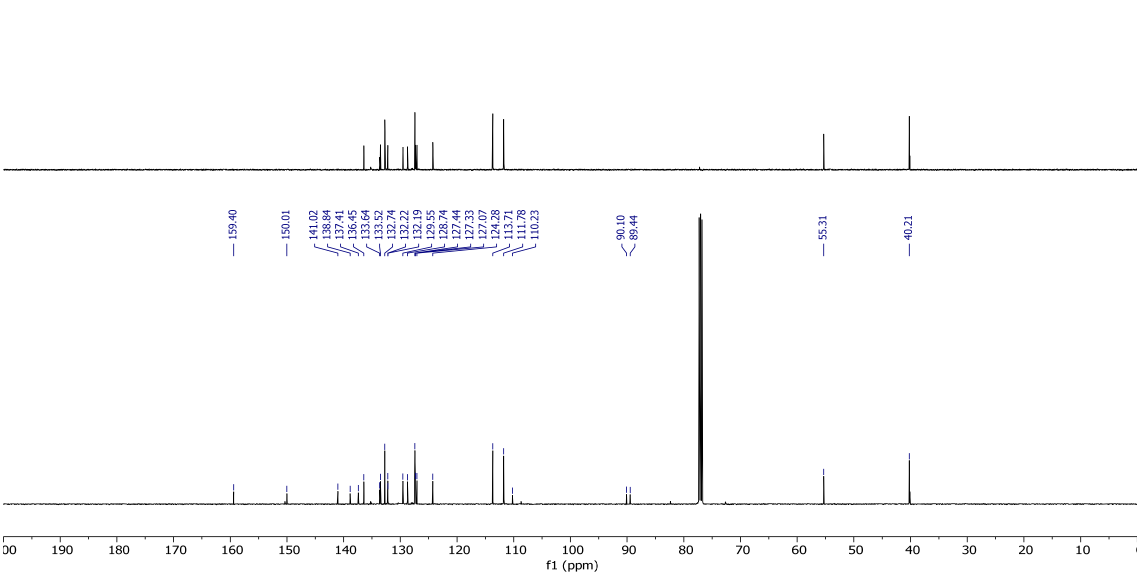

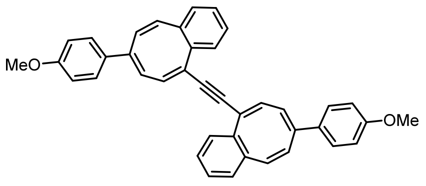

11c

^{1}H-NMR (500 Hz), ^{13}C-NMR, DEPT (126 Hz) in $CDCl_3$

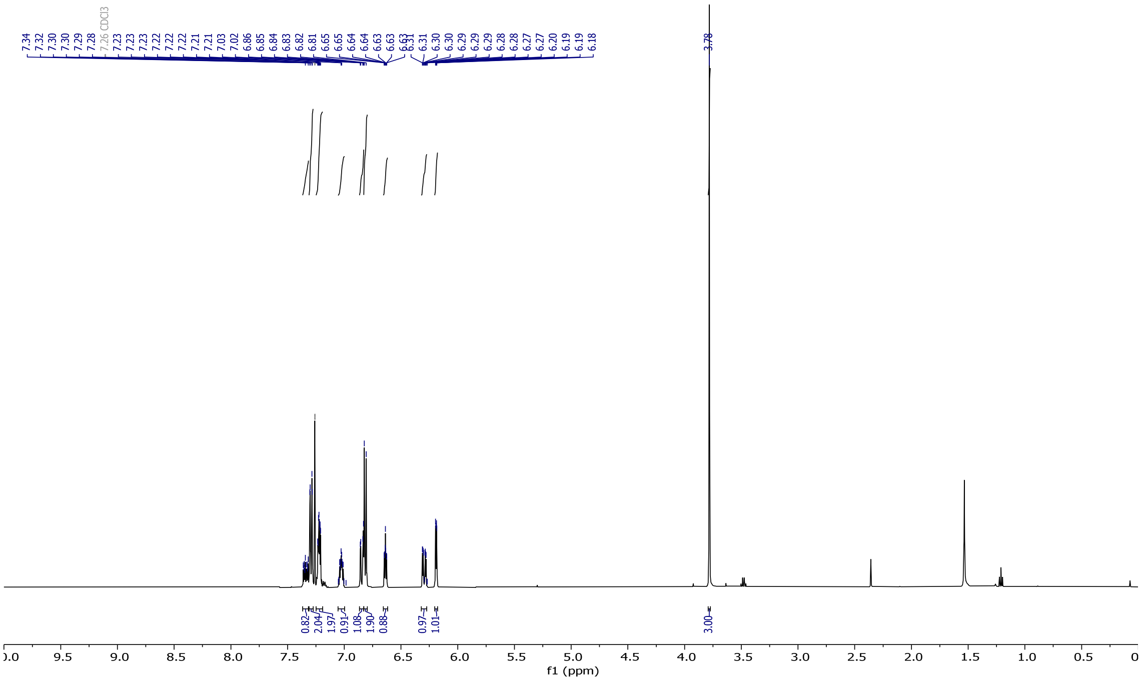

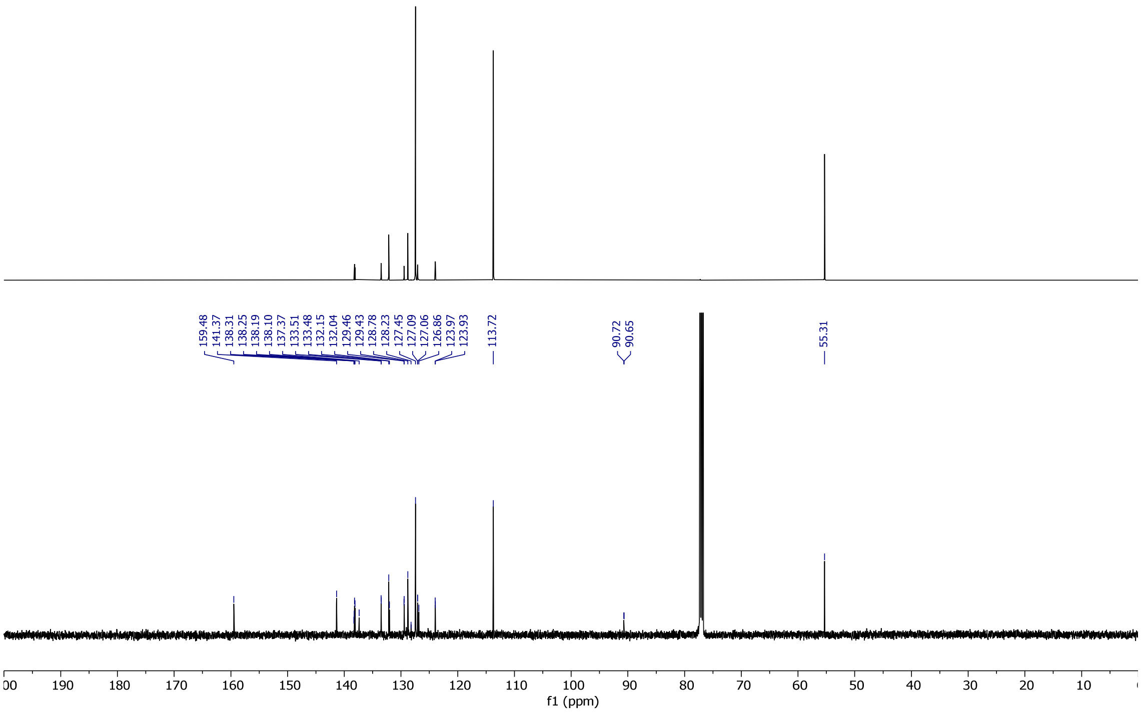

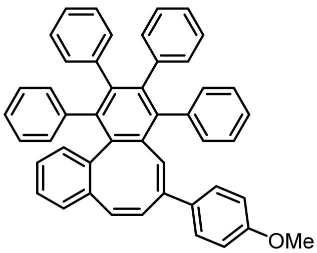

12

^{1}H-NMR (500 Hz) and ^{13}C-NMR, DEPT (126 Hz) in $CDCl_3$

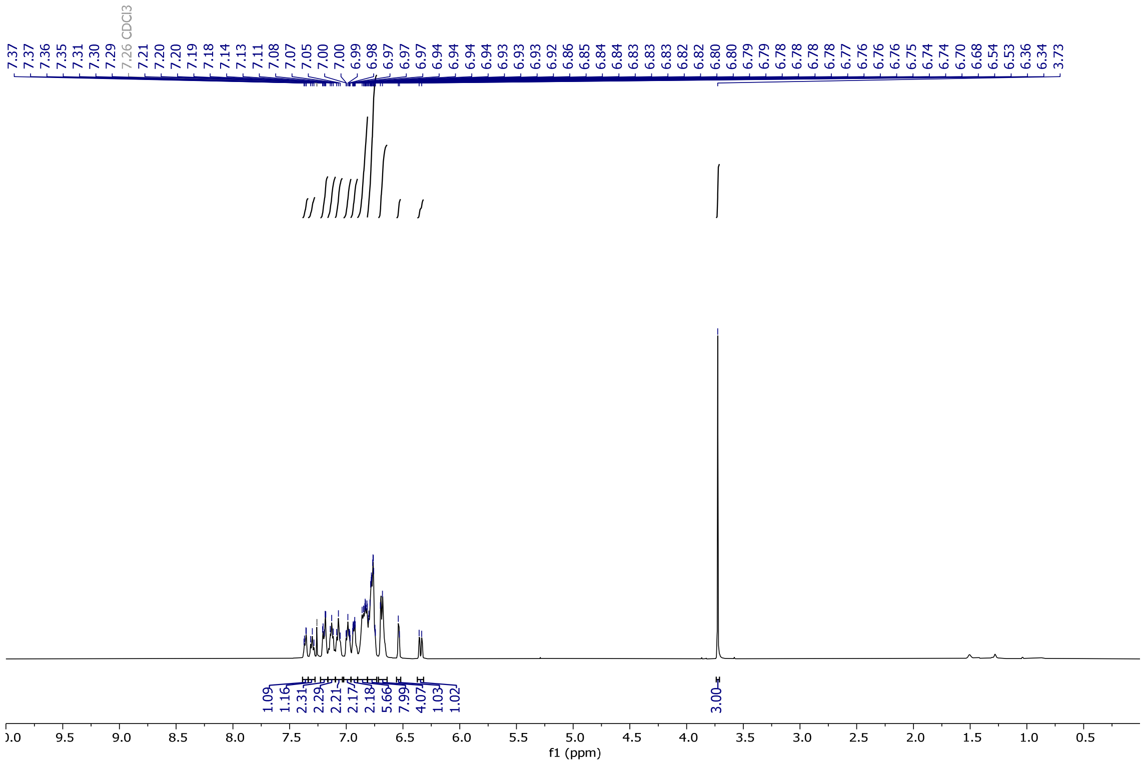

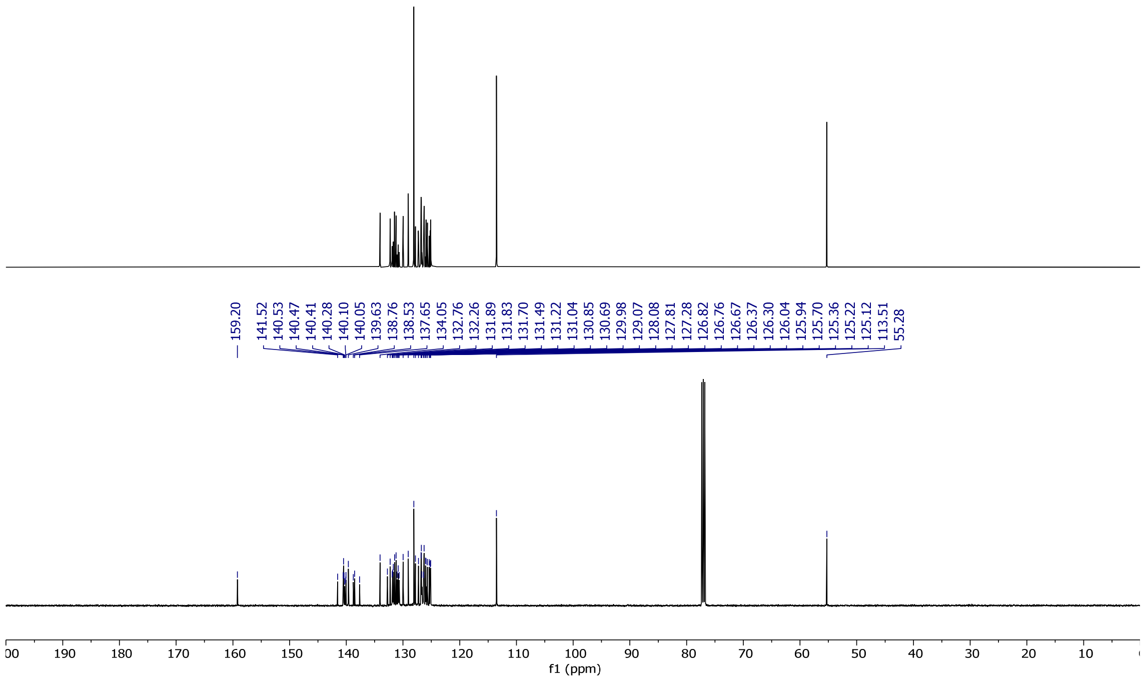

Supplement: Supplementary file 1 — ol1c01881_si_001.pdf [file ol1c01881_si_001.pdf]
