# Supplementary material for: Nonplanar Tub-Shaped Benzocyclooctatetraenes via Halogen-Radical Ring Opening of Dihydrobiphenylenes
Source: Org Lett. 2021 Jul 6;23(14):5539–44. doi: 10.1021/acs.orglett.1c01881 (PMC8499027; doi:10.1021/acs.orglett.1c01881)
Supplement: Supplementary file 2 — ol1c01881_si_002.pdf [file ol1c01881_si_002.pdf]

Nonplanar Tub-shaped Benzocyclooctatetraenes via Halogen-Radical Ring Opening of Dihydrobiphenylenes

Jesús Bello-García, Damián Padín, Jesús A. Varela and Carlos Saá*

Centro Singular de Investigación en Química Biolóxica e Materiais Moleculares (CiQUS), Departamento de Química Orgánica, Universidade de Santiago de Compostela, 15782 Santiago de Compostela (Spain)

Supporting Information

Table of Contents

1. Computational details 2

2. Free energy profile for the isomerization of benzodiCOT **9** from the U- to S-shaped conformers 2

3. Complete free energy profile for the radical bromination of dihydrobiphenylene **3e** and 6 π e^- electrocyclic ring opening of brominated dihydrobiphenylene **III** 3

4. Natural Bond Orbital (NBO) analysis 4

5. References 7

6. Cartesian coordinates in Å, energy values in Hartrees and imaginary frequencies in cm^{-1} for all the stationary points involved throughout DFT study 9

1. Computational details

All electronic structure calculations were performed using the Gaussian 09 software package[1] at the CESGA facilities. The geometries of all minima and transition states involved were optimized using the ωB97XD functional[2] (which includes a version of Grimme's D2 dispersion corrections)[3] within the self-consistent reaction field (SCRF) using the SMD model (CCl_4)[4] and using the basis set aug-cc-pVTZ.[5] Frequency calculations were performed at the same level to evaluate the zero-point vibrational energy and thermal corrections at 298 K and to confirm the nature of the stationary points, yielding one imaginary frequency for the transition states and none for the minima. Each transition state was further confirmed by following the steepest descent to both sides and identifying the minima present in the reaction energy profile. The reaction profiles were built up in terms of ΔG_{sol}.

Intermediates **9_S_shaped**, **9_U_Shaped** and **TS**$_{\mathbf{9\_S\_shaped\text{-}9\_U\_Shaped}}$ were also optimized using the ωB97XD functional[2] (which includes a version of Grimme's D2 dispersion corrections)[3] within the self-consistent reaction field (SCRF) using the SMD model (CCl_4)[4] but using the basis set 6-31G(d,p).[6] Frequency calculations were performed at the same level to evaluate the zero-point vibrational energy and thermal corrections at 298 K and to confirm the nature of the stationary points, yielding one imaginary frequency for the transition states and none for the minima. The transition state was further confirmed by following the steepest descent to both sides and identifying the minima present in the reaction energy profile. Single-point energies were calculated using ωB97XD functional[2] (which includes a version of Grimme's D2 dispersion corrections)[3] within the self-consistent reaction field (SCRF) using the SMD model (CCl_4)[4] and using the basis set aug-cc-pVTZ.[5] The resulting energies were used to correct the energies obtained from optimization calculations. The reaction profiles were built up in terms of ΔG_{sol}.

2. Free energy profile for the isomerization of benzodiCOT 9 from the U- to S-shaped conformers

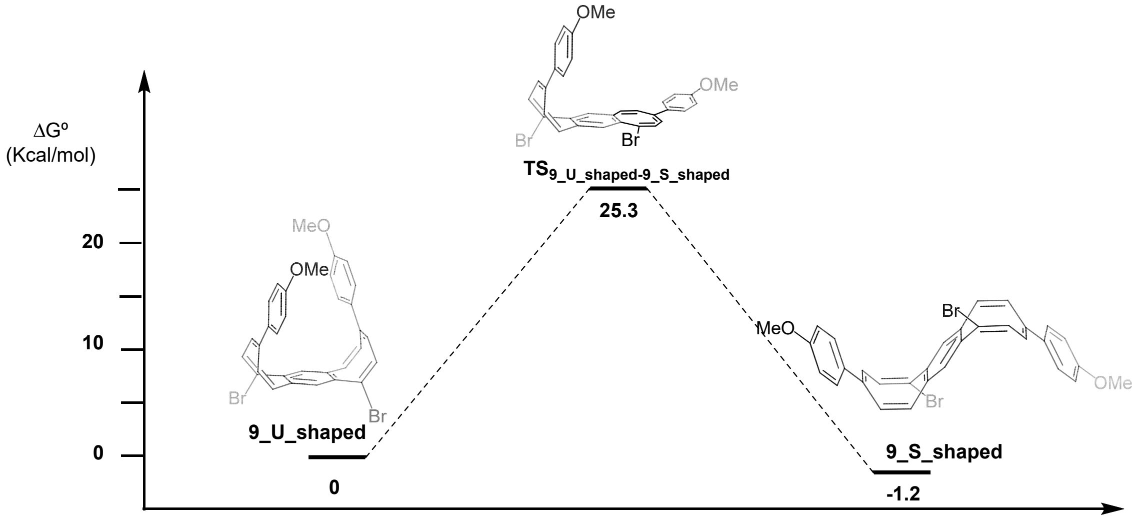

Figure 1S: Free energy profile for the isomerization of benzodiCOT **9** from the U- to S-shaped conformers

3. Complete free energy profile for the radical bromination of dihydrobiphenylene **3e** and 6 π e⁻ electrocyclic ring opening of brominated dihydrobiphenylene **III**

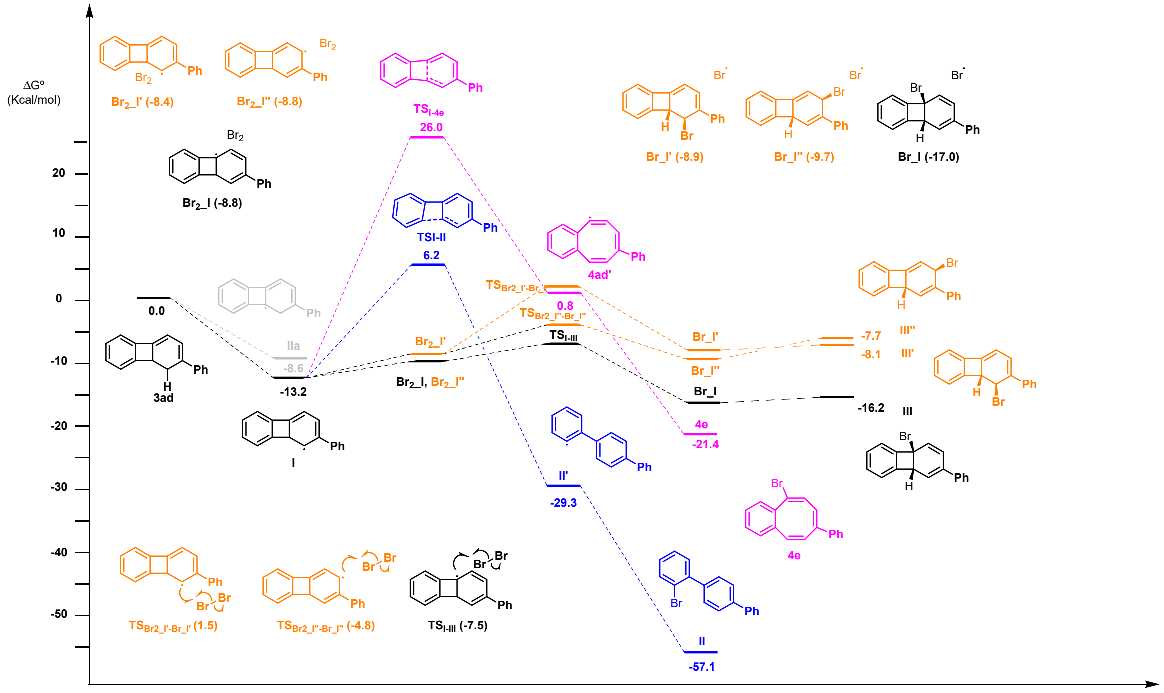

Figure 2S: Free energy profile for the radical bromination of dihydrobiphenylene **3e**. Energies are relative to **3e** and are mass balanced.

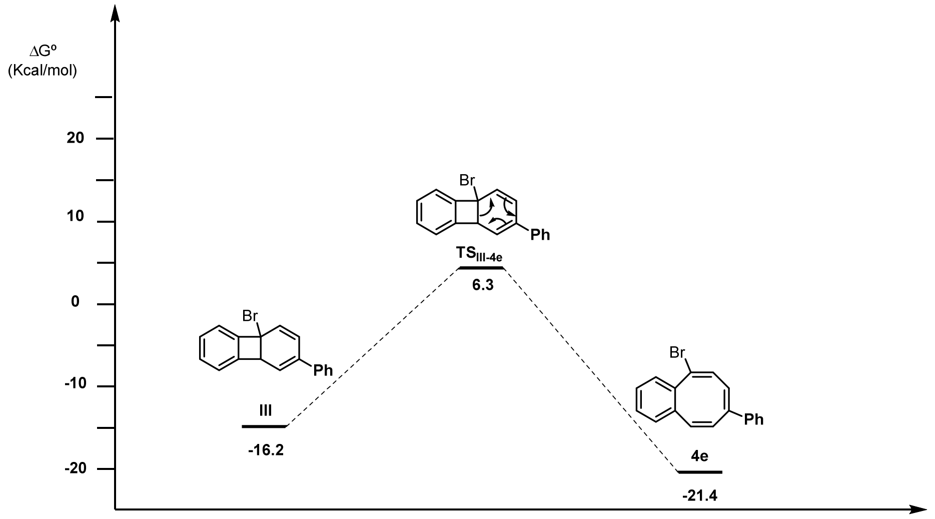

Figure 3S: Free energy profile for the 6 π e⁻ electrocyclic ring opening of brominated dihydrobiphenylene **III**. Energies are relative to **3e** and are mass balanced.

4. Natural Bond Orbital (NBO) analysis

Natural Bond Orbital analysis was carried out using NBO 3.1[7] as implemented in Gaussian package.[1] The Natural Localized Molecular Orbitals (NMLO) associated to the unpair electron and adjacent double bonds interactions have been determined for radical **I**.[8] The spin density (contour value 0.008, Figure 3S) and the NBO (contour value 0.04, Figure 4S), the NMLO (contour value 0.04, Figure 5S) and canonical orbital (contour value 0.04, Figure 6S) isosurfaces associated to the unpair electron for radical **I** were visualized using ChemCraft.[9]

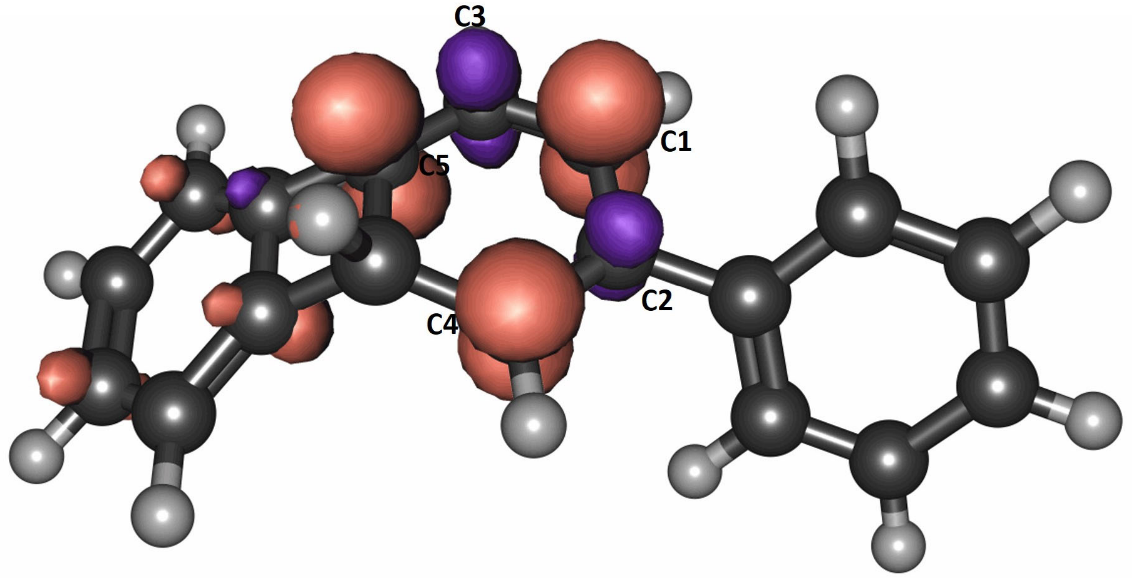

Figure 4S: Spin density map (contour value 0.008) associated to the unpair electron for radical **I**.

Carbon	Spin density
C1	0.50504
C4	0.33639
C5	0.40494

Table 1S: Mulliken spin densities for radical **I**.

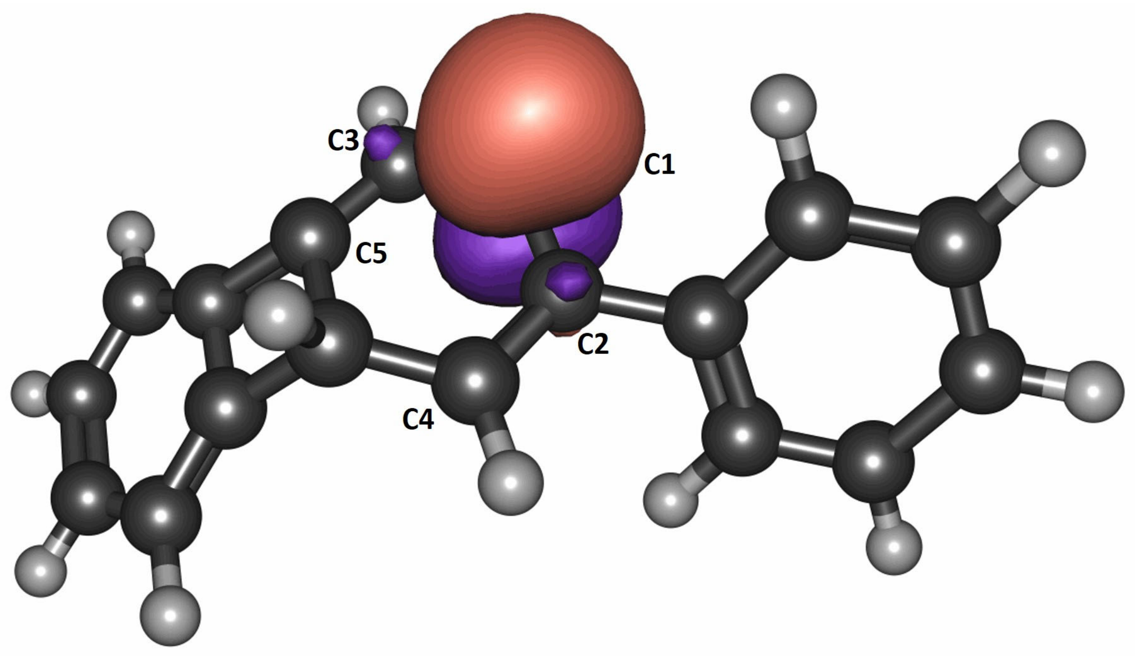

Figure 5S: Natural Bond Orbital plot (contour value 0.04) associated to the unpair electron for radical **I**.

Occupancy	Bond orbital	Coefficients/hybrids
0.72937	Lone Pair C1	s(0.20%)p99.99(99.75%)d 0.05(0.01%) f 0.20(0.04%)

Table 2S: Natural Bond Orbital analysis of occupancy, type and composition associated to the unpair electron for radical **I**.

Donor NBO	Acceptor NBO	Donor-Acceptor stabilization E (Kcal mol^{-1})
Lone Pair C1	Π* C3-C5<br>Π* C2-C4	50.34<br>37.96

Table 3S: Second order perturbation theory analysis for the stabilization due to the delocalization associated to the unpair electron for radical **I**.

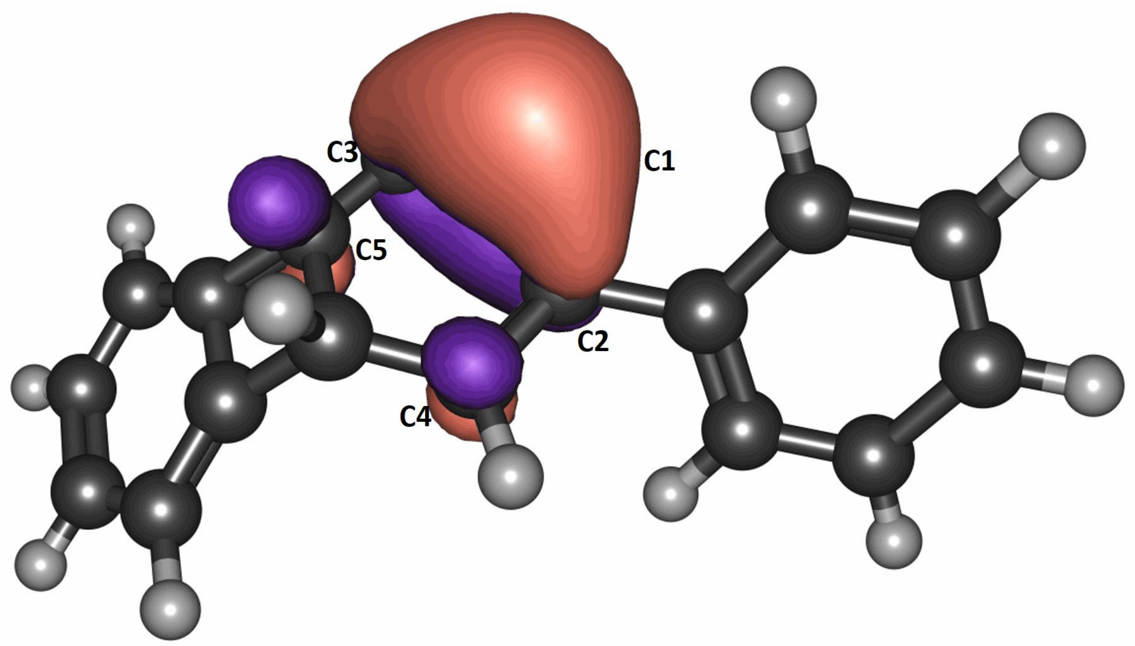

Figure 6S: Natural Localized Molecular Orbital plot (contour value 0.04) associated to the unpair electron for radical **I**.

Occupancy	Percent from Parent NBO	Atomic Hybrid contributions
1	72.2571% LP C1	72.268% C1 s(0.11%)p99.99(99.84%)d0.10(0.01%)f 0.36(0.04%)<br><br>6.773% C2 s(0.02%) p99.99(99.27%) d24.08(0.44%) f14.96(0.27%)<br><br>11.009% C3 s(0.09%) p99.99(99.55%) d3.55(0.32%) f0.35(0.03%)<br><br>3.969% C4 s(0.02%) p99.99(99.63%) d6.83(0.13%) f10.82(0.21%)<br>3.834% C5 s(2.36%) p41.09(96.84%) d0.10(0.23%) f0.24(0.57%)

Table 4S: Natural Localized Molecular Orbital analysis associated to the unpair electron for radical **I**.

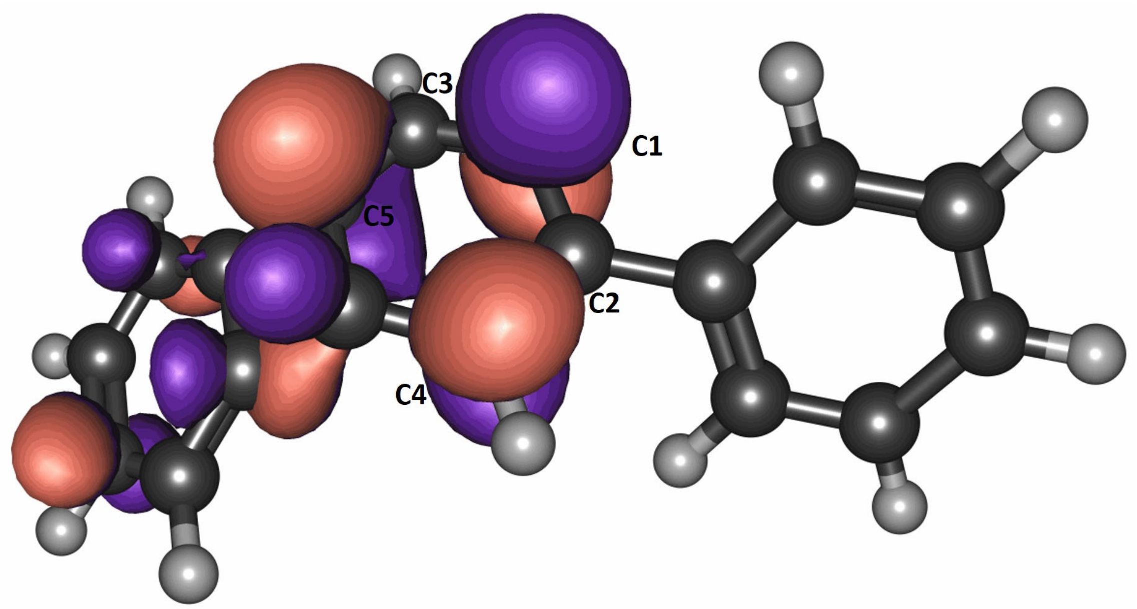

Figure 7S: Canonical orbital plot (contour value 0.04) associated to the unpair electron for radical **I**.

5. References

(1) Frisch, M. J.; Trucks, G. W.; Schlegel, H. B.; Scuseria, G. E.; Robb, M. A.; Cheeseman, J. R.; Scalmani, G.; Barone, V.; Petersson, G. A.; Nakatsuji, H.; Li, X.; Caricato, M.; Marenich, A. V.; Bloino, J.; Janesko, B. G.; Gomperts, R.; Mennucci, B.; Hratchian, H. P.; Ortiz, J. V.; Izmaylov, A. F.; Sonnenberg, J. L.; Williams; Ding, F.; Lipparini, F.; Egidi, F.; Goings, J.; Peng, B.; Petrone, A.; Henderson, T.; Ranasinghe, D.; Zakrzewski, V. G.; Gao, J.; Rega, N.; Zheng, G.; Liang, W.; Hada, M.; Ehara, M.; Toyota, K.; Fukuda, R.; Hasegawa, J.; Ishida, M.; Nakajima, T.; Honda, Y.; Kitao, O.; Nakai, H.; Vreven, T.; Throssell, K.; Montgomery Jr., J. A.; Peralta, J. E.; Ogliaro, F.; Bearpark, M. J.; Heyd, J. J.; Brothers, E. N.; Kudin, K. N.; Staroverov, V. N.; Keith, T. A.; Kobayashi, R.; Normand, J.; Raghavachari, K.; Rendell, A. P.; Burant, J. C.; Iyengar, S. S.; Tomasi, J.; Cossi, M.; Millam, J. M.; Klene, M.; Adamo, C.; Cammi, R.; Ochterski, J. W.; Martin, R. L.; Morokuma, K.; Farkas, O.; Foresman, J. B.; Fox, D. J. *Gaussian 16 Rev. C.01*: Wallingford, CT, 2019.

(2) Chai, J.-D.; Head-Gordon, M. Long-range corrected hybrid density functionals with damped atom-atom dispersion corrections *Phys. Chem. Chem. Phys.* **2008**, *10*, 6615-6620.

(3) Grimme, S. Semiempirical GGA-type density functional constructed with a long-range dispersion correction *J. Comput. Chem.* **2006**, *27*, 1787-1799.

(4) Marenich, A. V.; Cramer, C. J.; Truhlar, D. G. Universal Solvation Model Based on Solute Electron Density and on a Continuum Model of the Solvent Defined by the Bulk Dielectric Constant and Atomic Surface Tensions *J. Phys. Chem. B* **2009**, *113*, 6378-6396.

(5) (a) Dunning, T. H., Jr. Gaussian basis sets for use in correlated molecular calculations. I. The atoms boron through neon and hydrogen *J. Chem. Phys.* **1989**, *90*, 1007-1023. (b) Kendall, R. A.; Dunning, T. H., Jr.; Harrison, R. J. Electron affinities of the first-row atoms revisited. Systematic basis sets and wave functions *J. Chem. Phys.* **1992**, *96*, 6796-6806. (c) Wilson, A. K.; Woon, D. E.; Peterson, K. A.; Dunning, T. H., Jr. Gaussian basis sets for use in correlated molecular calculations. IX. The atoms gallium through krypton *J. Chem. Phys.* **1999**, *110*, 7667-7676.

(6) (a) Ditchfield, R.; Hehre, W. J.; Pople, J. A. Self-Consistent Molecular-Orbital Methods. IX. An Extended Gaussian-Type Basis for Molecular-Orbital Studies of Organic Molecules *J. Chem. Phys.* **1971**, *54*, 724-728. (b) Hehre, W. J.; Ditchfield, R.; Pople, J. A. Self-Consistent Molecular Orbital Methods. XII. Further Extensions of Gaussian-Type Basis Sets for Use in Molecular Orbital Studies of Organic Molecules *J. Chem. Phys.* **1972**, *56*, 2257-2261. (c) Hariharan, P. C.; Pople, J. A. The influence of polarization functions on molecular orbital hydrogenation energies *Theor. Chim. Acta* **1973**, *28*, 213-222. (d) Rassolov,

V. A.; Ratner, M. A.; Pople, J. A.; Redfern, P. C.; Curtiss, L. A. 6-31G* basis set for third-row atoms *J. Comput. Chem.* **2001**, *22*, 976-984.

(7) NBO Version 3.1, E. D. Glendening, A. E. Reed, J. E. Carpenter, and F. Weinhold.

(8) Reed, A. E.; Weinhold, F. Natural localized molecular orbitals *J. Chem. Phys.* **1985**, *83*, 1736-1740.

(9) Chemcraft - graphical software for visualization of quantum chemistry computations. https://www.chemcraftprog.com.

6. Cartesian coordinates in Å, energy values in Hartrees and imaginary frequencies in cm^{-1} for all the stationary points involved throughout DFT study

9_U_shaped

Electronic Energy 6-31G(d,p) = -6527.27269965 Hartree

Electronic Energy AUG-CC-PVTZ = -6532.75148381 Hartree

Zero-point Energy Correction = 0.472137 Hatree

Thermal Correction to Enthalpy = 0.504463 Hartree

Thermal Correction to Free Energy = 0.404937 Hartree

Chemical symbol X, Y, Z

C	1.330569	-2.031413	-0.196285
C	0.806489	-2.011290	1.104795
C	-2.923127	0.206645	-1.949749
C	-2.657158	-1.034680	-2.687907
C	-2.912431	-1.858932	0.499419
C	-1.812635	-1.987965	-2.293610
C	-1.461778	-1.925959	0.205259
C	-0.938911	-1.978721	-1.095069
H	-2.754753	1.124184	-2.511660
H	-3.154332	-1.144476	-3.649407
H	-3.294918	-2.636340	1.158453
C	-3.417221	0.297817	-0.702650
C	-3.747078	-0.887290	0.121184
H	-4.767677	-0.914816	0.500265
C	0.442466	-2.048605	-1.270429
C	-0.576379	-1.977637	1.280298
H	-0.979274	-1.988670	2.289124
H	0.842669	-2.112265	-2.278250
C	-3.737182	1.614599	-0.091037
C	-3.574465	1.808134	1.281700
C	-4.203706	2.697856	-0.851637
C	-3.838269	3.034696	1.886374
H	-3.215822	0.986899	1.895327
C	-4.471546	3.922312	-0.267324
H	-4.380355	2.572053	-1.915492
C	-4.287160	4.102861	1.108752
H	-3.687721	3.141297	2.953958
H	-4.839234	4.755570	-0.856559
C	2.590794	-1.149472	2.675218
C	2.953131	0.045494	1.905857
C	3.419856	0.071069	0.644032
C	3.682627	-1.162768	-0.133524
C	2.782817	-2.082370	-0.486406
C	1.676521	-2.047965	2.305029
H	3.073647	-1.268377	3.642702
H	4.703501	-1.296372	-0.486743
H	3.110263	-2.910997	-1.111475
H	2.907035	0.982503	2.458644
C	3.753295	1.365703	-0.009619

C	2.944368	2.494149	0.198209
C	4.858862	1.510612	-0.849891
C	3.242094	3.712612	-0.384834
H	2.050186	2.404287	0.807214
C	5.174703	2.730746	-1.442404
H	5.510761	0.665448	-1.045585
C	4.364982	3.843135	-1.209184
H	2.606508	4.577616	-0.228435
H	6.049196	2.797027	-2.078594
Br	-1.583037	-3.520608	-3.423675
Br	1.329051	-3.521424	3.483269
O	-4.577799	5.336207	1.586301
O	4.573377	5.073077	-1.736278
C	-4.417018	5.568384	2.970905
H	-4.704233	6.607069	3.138653
H	-5.066433	4.917152	3.568822
H	-3.375542	5.430415	3.286786
C	5.683307	5.249983	-2.592603
H	5.615438	4.610096	-3.481052
H	5.660944	6.294652	-2.904909
H	6.630137	5.052461	-2.074965

$TS_{9\_S\_shaped-\_U\_shaped}$

Imaginary Freq = -52.4778 (cm^{-1})

Electronic Energy 6-31G(d,p) = -6527.23772606 Hartree

Electronic Energy AUG-CC-PVTZ = -6532.71449836 Hartree

Zero-point Energy Correction = 0.473505 Hatree

Thermal Correction to Enthalpy = 0.504975 Hartree

Thermal Correction to Free Energy = 0.408283 Hartree

Chemical symbol X, Y, Z

C	-0.896477	0.905589	0.233417
C	-0.449340	-0.088244	1.159189
C	4.002005	1.784914	-1.203754
C	3.332827	3.033638	-0.816287
C	3.140490	0.976583	1.778230
C	2.185500	3.111040	-0.141103
C	1.779744	1.015074	1.197854
C	1.336168	1.989230	0.319523
H	4.205616	1.685559	-2.269031
H	3.791838	3.955941	-1.166459
H	3.185315	0.940763	2.865293
C	4.444185	0.823534	-0.374626
C	4.284969	0.885689	1.096152
H	5.207525	0.770367	1.663092
C	0.014257	1.902039	-0.124664
C	0.870121	0.027629	1.596335
H	1.253148	-0.695806	2.299353
H	-0.323561	2.679553	-0.801569
C	5.202448	-0.349424	-0.884605
C	5.062662	-1.595711	-0.271467
C	6.074051	-0.255184	-1.980441
C	5.739807	-2.720990	-0.734377
H	4.394940	-1.698951	0.578875

C	6.755607	-1.362266	-2.452012
H	6.237714	0.706697	-2.456777
C	6.591601	-2.607808	-1.833962
H	5.591843	-3.670308	-0.233826
H	7.435033	-1.284959	-3.294123
C	-2.492699	-1.727387	1.554471
C	-3.725553	-1.460364	0.829254
C	-4.146814	-0.538112	-0.056794
C	-3.412214	0.599645	-0.608470
C	-2.183508	1.119895	-0.477451
C	-1.249738	-1.231031	1.707591
H	-2.653839	-2.604309	2.171461
H	-4.013266	1.152344	-1.325667
H	-2.077839	1.992317	-1.116234
H	-4.482459	-2.188336	1.108061
C	-5.547095	-0.669235	-0.558616
C	-6.057916	-1.909561	-0.969630
C	-6.399886	0.434884	-0.621057
C	-7.362715	-2.040064	-1.411732
H	-5.410925	-2.781551	-0.962748
C	-7.717631	0.320058	-1.055871
H	-6.044194	1.409528	-0.299318
C	-8.206492	-0.924942	-1.455725
H	-7.749015	-2.998896	-1.740417
H	-8.346785	1.201862	-1.071975
Br	1.454320	4.846829	0.217320
Br	-0.293567	-2.337173	3.007934
O	7.302811	-3.629227	-2.366522
C	7.182693	-4.905093	-1.770320
H	7.830833	-5.568751	-2.343729
H	7.515164	-4.896132	-0.725028
H	6.153267	-5.280215	-1.820371
O	-9.464604	-1.151736	-1.901843
C	-10.354181	-0.055647	-1.970196
H	-11.296322	-0.454121	-2.348196
H	-10.525493	0.389133	-0.982232
H	-9.991811	0.717724	-2.658563

9_S_shaped

Electronic Energy 6-31G(d,p) = -6527.27482375 Hartree

Electronic Energy AUG-CC-PVTZ = -6532.75318357 Hartree

Zero-point Energy Correction = 0.471882 Hatree

Thermal Correction to Enthalpy = 0.504282 Hartree

Thermal Correction to Free Energy = 0.404718 Hartree

Chemical symbol X, Y, Z

C	-0.923632	-0.511335	-0.937006
C	-0.369656	-1.354403	0.036965
C	3.152388	2.584568	-0.499028
C	1.903310	3.340242	-0.340318
C	1.886183	0.974797	1.964371
C	0.706930	2.796738	-0.113795
C	0.923631	0.511336	0.937005
C	0.369656	1.354403	-0.036966
H	3.693015	2.774365	-1.425190

H	1.968522	4.418386	-0.471521	C	-5.029185	-1.128534	-0.178082
H	1.599511	0.784258	2.997032	C	-6.052712	-1.772388	0.533954
C	3.706622	1.765819	0.412152	C	-5.286433	0.152198	-0.670052
C	3.092937	1.497180	1.732287	C	-7.267891	-1.152230	0.760909
H	3.736671	1.695919	2.587941	H	-5.901216	-2.784312	0.897361
C	-0.537883	0.827952	-0.955293	C	-6.503321	0.792087	-0.448936
C	0.537883	-0.827951	0.955292	H	-4.511826	0.674418	-1.223994
H	0.970836	-1.486048	1.703326	C	-7.502554	0.139284	0.274066
H	-0.970837	1.486049	-1.703327	H	-8.060621	-1.654825	1.304734
C	5.029185	1.128533	0.178082	H	-6.653917	1.791223	-0.839839
C	5.286433	-0.152198	0.670053	Br	-0.807255	3.966091	0.030868
C	6.052712	1.772386	-0.533954	Br	0.807255	-3.966090	-0.030869
C	6.503321	-0.792088	0.448939	O	8.722150	-0.660377	-0.547167
H	4.511826	-0.674417	1.223996	O	-8.722150	0.660375	0.547169
C	7.267891	1.152228	-0.760909	C	9.008933	-1.962161	-0.077676
H	5.901216	2.784310	-0.897362	H	10.024618	-2.185315	-0.406407
C	7.502553	-0.139285	-0.274065	H	8.968186	-2.015894	1.017181
H	6.653917	-1.791223	0.839842	H	8.323940	-2.705829	-0.502971
H	8.060620	1.654823	-1.304735	C	-9.008934	1.962159	0.077679
C	-1.903310	-3.340242	0.340318	H	-8.968187	2.015893	-1.017178
C	-3.152388	-2.584569	0.499028	H	-10.024619	2.185313	0.406410
C	-3.706622	-1.765819	-0.412152	H	-8.323941	2.705827	0.502975
C	-3.092938	-1.497179	-1.732287				
C	-1.886184	-0.974796	-1.964371				
C	-0.706930	-2.796738	0.113794				
H	-1.968521	-4.418385	0.471520				
H	-3.736672	-1.695918	-2.587941				
H	-1.599512	-0.784257	-2.997032				
H	-3.693014	-2.774365	1.425190				

Br

Electronic Energy aug-cc-pVTZ = -2574.22224046 Hartree

Zero-point Energy Correction = 0.000000 Hartree

Thermal Correction to Enthalpy = 0.002360 Hartree

Thermal Correction to Free Energy = -0.016830 Hartree

Chemical symbol X, Y, Z

Br	0.000000	0.000000	0.000000

HBr

Electronic Energy aug-cc-pVTZ = -2574.87108533 Hartree

Zero-point Energy Correction = 0.006089 Hartree

Thermal Correction to Enthalpy = 0.009393 Hartree

Thermal Correction to Free Energy = -0.013130 Hartree

Chemical symbol X, Y, Z

H	0.000000	0.000000	-1.379909
Br	0.000000	0.000000	0.039426

Br_2

Electronic Energy aug-cc-pVTZ = -5148.52091554 Hartree

Zero-point Energy Correction = 0.000775 Hartree

Thermal Correction to Enthalpy = 0.004452 Hartree

Thermal Correction to Free Energy = -0.023330 Hartree

Chemical symbol X, Y, Z

Br	0.000000	0.000000	1.141942
Br	0.000000	0.000000	-1.141942

3ad

Electronic Energy aug-cc-pVTZ = -694.290743401 Hartree

Zero-point Energy Correction = 0.264341 Hartree

Thermal Correction to Enthalpy = 0.278400 Hartree

Thermal Correction to Free Energy = 0.223824 Hartree

Chemical symbol X, Y, Z

C	-5.122102	-0.088731	-0.316405
C	-4.738355	-1.374533	0.063946
C	-3.426355	-1.674774	0.434077
C	-2.538650	-0.622618	0.410523
C	-2.919215	0.660555	0.010185
C	-4.215427	0.967479	-0.359864
C	-1.096449	-0.207294	0.688279
C	-1.534855	1.137657	0.109137
C	0.121315	-0.724573	-0.055806
C	1.156358	0.395142	-0.102979
C	0.751137	1.670385	-0.248012
C	-0.663385	2.028360	-0.354449
C	2.588740	0.039396	-0.050133
C	3.043666	-1.156800	-0.609184
C	4.389705	-1.483818	-0.598169
C	5.312405	-0.628477	-0.015322
C	4.874270	0.555851	0.558631
C	3.528976	0.883860	0.544117
H	-6.153998	0.086466	-0.590639
H	-5.484592	-2.158108	0.074483

H	-3.147365	-2.678525	0.725332
H	-4.523584	1.956001	-0.671307
H	-0.886537	-0.144026	1.759099
H	2.340150	-1.834075	-1.074959
H	4.718853	-2.411116	-1.047989
H	6.362703	-0.886273	-0.000828
H	5.582530	1.223661	1.030856
H	3.198030	1.797318	1.019949
H	-0.951403	2.943845	-0.854078
H	1.487336	2.455532	-0.364198
H	-0.149790	-1.003085	-1.079759
H	0.537004	-1.610025	0.422605

I

Electronic Energy aug-cc-pVTZ = -693.651986200 Hartree

Zero-point Energy Correction = 0.250372 Hartree

Thermal Correction to Enthalpy = 0.264366 Hartree

Thermal Correction to Free Energy = 0.209250 Hartree

Chemical symbol X, Y, Z

C	-4.959973	-0.235295	-0.669506
C	-4.577059	-1.476192	-0.163328
C	-3.330037	-1.676369	0.438049
C	-2.510213	-0.576608	0.496068
C	-2.882694	0.666155	-0.033985
C	-4.118529	0.874122	-0.621278
C	-1.166225	-0.032561	1.006986
C	-1.561971	1.217709	0.241802
C	0.208018	-0.498896	0.684549
C	1.106520	0.394063	0.186787
C	0.704813	1.718232	-0.206221
C	-0.645602	2.081285	-0.284394
C	2.530081	0.007730	0.007484
C	2.871964	-1.233050	-0.527490
C	4.199538	-1.598475	-0.681546
C	5.211714	-0.728060	-0.305798
C	4.884361	0.511264	0.222969
C	3.556182	0.876294	0.375522
H	-5.939283	-0.136983	-1.118939
H	-5.269172	-2.304652	-0.235721
H	-3.050258	-2.645248	0.828983
H	-4.426206	1.826756	-1.029543
H	-1.240873	0.116649	2.092949
H	2.088475	-1.909786	-0.842495
H	4.443764	-2.563537	-1.105231
H	6.248211	-1.012237	-0.427357
H	5.666045	1.196368	0.523074
H	3.313076	1.840528	0.802875
H	-0.938488	2.953377	-0.855016
H	1.455583	2.382453	-0.611150
H	0.523800	-1.491786	0.977552

I'

Electronic Energy aug-cc-pVTZ = -693.643961071 Hartree

Zero-point Energy Correction = 0.250099 Hartree

Thermal Correction to Enthalpy = 0.264347 Hartree

Thermal Correction to Free Energy = 0.208519 Hartree

Chemical symbol X, Y, Z

C	-5.237642	0.005891	0.062978
C	-4.856391	-1.313956	0.229916
C	-3.506141	-1.709430	0.254794
C	-2.586057	-0.697911	0.104671
C	-2.984394	0.657752	-0.066779
C	-4.288462	1.043341	-0.093692
C	-1.180725	-0.310388	0.024613
C	-1.538275	1.051075	-0.150575
C	0.206751	-0.808452	0.016353
C	1.179942	0.355267	-0.101719
C	0.756218	1.629344	-0.273111
C	-0.631910	2.039604	-0.313940
C	2.620675	0.033894	-0.024850
C	3.110791	-1.198566	-0.463745
C	4.463393	-1.494865	-0.405999
C	5.361520	-0.571303	0.103718
C	4.890281	0.650452	0.562354
C	3.539796	0.945740	0.504026
H	-6.291266	0.250019	0.051700
H	-5.624895	-2.066934	0.345153
H	-3.233031	-2.747253	0.385214
H	-4.603712	2.069063	-0.225583
H	2.434811	-1.935735	-0.874111
H	4.815263	-2.453464	-0.763444
H	6.416630	-0.803711	0.152812
H	5.577384	1.373654	0.981183
H	3.189117	1.889503	0.898861
H	-0.888404	3.078350	-0.461491
H	1.499646	2.405822	-0.397814
H	0.352786	-1.507093	-0.818868
H	0.439456	-1.397154	0.912302

$TS_{I\text{-}4ad}$

Imaginary Freq = -650.0085 (cm^{-1})

Electronic Energy aug-cc-pVTZ = -693.587396855 Hartree

Zero-point Energy Correction = 0.248651 Hartree

Thermal Correction to Enthalpy = 0.262694 Hartree

Thermal Correction to Free Energy = 0.207058 Hartree

Chemical symbol X, Y, Z

C	-4.243001	-0.678357	-1.199633
H	-5.030194	-0.787462	-1.933709
C	-3.716766	0.579446	-0.944588
H	-4.072189	1.454647	-1.470578
C	-2.718113	0.657769	0.021433
C	-2.256805	-0.460793	0.682666
C	-2.757732	-1.719310	0.412987
H	-2.382759	-2.601005	0.915984
C	-3.772559	-1.810985	-0.535505

H	-4.204229	-2.775793	-0.765299
C	-1.860490	1.707034	0.497829
C	-1.185459	0.004837	1.616193
C	-0.798148	2.356929	-0.076872
H	-0.823051	3.433695	-0.226907
C	0.178980	-0.041760	1.341487
H	0.832348	-0.390810	2.137050
C	0.383923	1.690746	-0.378724
H	1.097649	2.219659	-0.999221
C	0.844541	0.524328	0.246765
C	2.235986	0.069763	-0.012751
C	3.289826	0.957836	-0.230725
C	2.511268	-1.297212	-0.027052
C	4.573284	0.492189	-0.461616
H	3.113701	2.024446	-0.192847
C	3.795566	-1.764623	-0.258187
H	1.702099	-1.999304	0.126410
C	4.831440	-0.870969	-0.478531
H	5.378058	1.197867	-0.618803
H	3.984652	-2.829652	-0.271931
H	5.834549	-1.232731	-0.659597
H	-1.445084	-0.094767	2.668380

4ad'

Electronic Energy aug-cc-pVTZ = -693.628948708 Hartree

Zero-point Energy Correction = 0.250177 Hartree

Thermal Correction to Enthalpy = 0.264691 Hartree

Thermal Correction to Free Energy = 0.208436 Hartree

Chemical symbol X, Y, Z

C	3.289851	0.906006	-0.283302
C	2.175335	0.143800	0.065749
C	2.299391	-1.244957	0.074427
C	3.492608	-1.854464	-0.279075
C	4.589247	-1.085491	-0.636705
C	4.483558	0.297578	-0.634385
H	3.227558	1.985846	-0.261054
H	1.446228	-1.852790	0.346112
H	3.564527	-2.933875	-0.276171
H	5.522471	-1.559995	-0.908412
H	5.338069	0.906871	-0.897127
C	0.875505	0.773927	0.423948
C	0.182953	0.148497	1.573983
C	0.458266	1.879905	-0.202484
C	-1.049863	-0.345162	1.692607
H	0.832691	0.015428	2.434456
C	-0.804337	2.580037	0.097686
H	1.082332	2.311108	-0.977995
C	-2.113026	-0.440826	0.675649
H	-1.287213	-0.824755	2.636859
C	-1.942207	1.936916	0.071584
H	-0.759631	3.643324	0.316122
C	-2.494415	0.647315	-0.137135
C	-2.762740	-1.660485	0.509413
C	-3.478272	0.456543	-1.121250
C	-3.731589	-1.833844	-0.464260

H	-4.213020	-2.795170	-0.580682
C	-4.081564	-0.771746	-1.290451
H	-3.765224	1.297114	-1.738466
H	-2.485583	-2.492140	1.145102
H	-4.835400	-0.902266	-2.054723

4ad

Electronic Energy aug-cc-pVTZ = -3267.97017451 Hartree

Zero-point Energy Correction = 0.253277 Hartree

Thermal Correction to Enthalpy = 0.269314 Hartree

Thermal Correction to Free Energy = 0.209101 Hartree

Chemical symbol X, Y, Z			
C	-3.684913	-1.482387	-0.524685
C	-2.917503	-0.474094	0.057850
C	-3.543120	0.725917	0.393527
C	-4.889572	0.922062	0.131763
C	-5.638461	-0.083531	-0.459837
C	-5.031334	-1.287891	-0.784081
H	-3.228144	-2.434980	-0.757891
H	-2.963784	1.518253	0.849249
H	-5.354344	1.864222	0.389739
H	-6.690605	0.067001	-0.660318
H	-5.611010	-2.083997	-1.231829
C	-1.466462	-0.652216	0.324563
C	-0.974121	-0.080829	1.593165
C	-0.690422	-1.347006	-0.514185
C	0.016991	0.791299	1.748521
H	-1.537934	-0.370413	2.474282
C	0.722222	-1.673732	-0.305159
H	-1.142509	-1.782565	-1.399311
C	0.804813	1.420170	0.667179
H	0.217729	1.158896	2.749262
C	1.691289	-0.780759	-0.158253
H	0.975516	-2.728004	-0.311410
C	1.574007	0.693337	-0.243108
C	0.784051	2.812475	0.569884
C	2.275504	1.368717	-1.242232
C	1.473830	3.472288	-0.428307
H	1.434448	4.551278	-0.490548
C	2.218709	2.744259	-1.346043
H	2.875728	0.801349	-1.940912
H	0.205162	3.377117	1.289689
H	2.762223	3.248975	-2.132808
Br	3.477271	-1.420446	0.122555

TS_{I-II}

Imaginary Freq = -616.5417 (cm^{-1})

Electronic Energy aug-cc-pVTZ = -693.620243769 Hartree

Zero-point Energy Correction = 0.249281 Hartree

Thermal Correction to Enthalpy = 0.263071 Hartree

Thermal Correction to Free Energy = 0.208323 Hartree

Chemical symbol X, Y, Z			
C	5.150952	-0.195948	-0.480308
H	6.125712	-0.580003	-0.749887
C	4.114981	-1.085165	-0.214853
H	4.270847	-2.154042	-0.275718
C	2.889346	-0.536509	0.128314
C	2.722639	0.838312	0.193902
C	3.729387	1.737299	-0.062400
H	3.595990	2.809161	-0.008814
C	4.965122	1.182904	-0.408245
H	5.797715	1.839765	-0.625834
C	1.512352	-0.931398	0.397310
C	0.992659	0.266904	1.030357
C	0.676321	-1.818006	-0.233591
H	1.080428	-2.670465	-0.763600
C	-0.382316	0.564451	0.851093
H	-0.804816	1.442243	1.321076
C	-0.686826	-1.531035	-0.313036
H	-1.340354	-2.211102	-0.842010
C	-1.209291	-0.320587	0.176518
C	-2.651188	-0.016184	0.009986
C	-3.618207	-1.007454	0.170143
C	-3.070676	1.273875	-0.312074
C	-4.964032	-0.717757	0.013327
H	-3.316121	-2.010814	0.440387
C	-4.415945	1.564147	-0.469971
H	-2.333292	2.052143	-0.459497
C	-5.368386	0.569203	-0.307770
H	-5.699694	-1.499158	0.149539
H	-4.720389	2.569692	-0.727927
H	-6.418887	0.795305	-0.431032
H	1.441510	0.594954	1.961099

II'

Electronic Energy aug-cc-pVTZ = -693.677837194 Hartree

Zero-point Energy Correction = 0.251615 Hartree

Thermal Correction to Enthalpy = 0.265984 Hartree

Thermal Correction to Free Energy = 0.209360 Hartree

Chemical symbol X, Y, Z			
C	5.061132	1.114628	-0.114327
H	5.626875	2.030935	-0.213512
C	3.675538	1.167870	-0.120710
H	3.179614	2.122975	-0.238444
C	2.906032	0.003404	-0.000020
C	3.636984	-1.158183	0.106902
C	4.996766	-1.277536	0.123563
H	5.491583	-2.234589	0.222332
C	5.728484	-0.095731	0.008472
H	6.810004	-0.127781	0.012013
C	1.426947	0.012842	0.005259
C	0.714910	-1.089051	-0.464335
C	0.704911	1.104388	0.481359
H	1.225118	1.961509	0.887562
C	-0.667414	-1.095862	-0.465777
H	-1.192034	-1.955825	-0.860780

C	-0.678448	1.097520	0.477876
H	-1.212050	1.949434	0.878274
C	-1.391836	-0.001141	0.003052
C	-2.873265	-0.006801	0.000491
C	-3.591192	1.138507	-0.341455
C	-3.583812	-1.157429	0.339982
C	-4.976588	1.133845	-0.344082
H	-3.059149	2.036104	-0.628299
C	-4.969188	-1.162981	0.337728
H	-3.046196	-2.051061	0.628705
C	-5.671383	-0.017171	-0.004423
H	-5.514996	2.030734	-0.619859
H	-5.501875	-2.063846	0.611614
H	-6.752980	-0.021217	-0.006369
H	1.254614	-1.944070	-0.850557

II

Electronic Energy aug-cc-pVTZ = -3268.02746078 Hartree

Zero-point Energy Correction = 0.254416 Hartree

Thermal Correction to Enthalpy = 0.270303 Hartree

Thermal Correction to Free Energy = 0.209594 Hartree

Chemical symbol X, Y, Z

C	-3.994044	2.459518	0.341232
H	-4.335801	3.476503	0.475218
C	-2.638437	2.187904	0.297318
H	-1.923011	2.992434	0.404381
C	-2.153729	0.889599	0.132738
C	-3.095678	-0.129608	-0.000477
C	-4.457441	0.127479	0.043456
H	-5.162195	-0.683782	-0.067418
C	-4.907068	1.424788	0.218438
H	-5.969661	1.621711	0.252787
C	-0.685408	0.671533	0.102009
C	-0.045230	-0.079906	1.082405
C	0.087371	1.258225	-0.894053
H	-0.393928	1.838796	-1.670233
C	1.327358	-0.245510	1.061092
H	1.804367	-0.814534	1.848035
C	1.461217	1.087719	-0.917362
H	2.036735	1.530401	-1.719543
C	2.105452	0.331491	0.058486
C	3.575233	0.144060	0.032978
C	4.425108	1.197390	-0.301401
C	4.141213	-1.091948	0.342342
C	5.799083	1.020872	-0.325029
H	4.007528	2.170254	-0.525428
C	5.515003	-1.269494	0.318377
H	3.498095	-1.928071	0.583766
C	6.349670	-0.213572	-0.015254
H	6.441869	1.852986	-0.579529
H	5.934158	-2.238628	0.553775
H	7.422171	-0.352014	-0.033909
H	-0.625051	-0.528790	1.877660
Br	-2.559809	-1.930300	-0.310381

Br_2_I

Electronic Energy aug-cc-pVTZ = -5842.17944520 Hartree

Zero-point Energy Correction = 0.251872 Hartree

Thermal Correction to Enthalpy = 0.270543 Hartree

Thermal Correction to Free Energy = 0.199468 Hartree

Chemical symbol X, Y, Z

C	-5.678292	-1.811094	0.376578
C	-5.813333	-1.071160	-0.797466
C	-4.733490	-0.398211	-1.377513
C	-3.534706	-0.514831	-0.718374
C	-3.401419	-1.237522	0.474339
C	-4.463663	-1.912172	1.050225
C	-2.037966	-0.166202	-0.754943
C	-2.021170	-0.797221	0.624015
C	-1.400729	1.175643	-0.787558
C	-0.486442	1.494615	0.164840
C	-0.253472	0.622499	1.292747
C	-1.116299	-0.447936	1.581512
C	0.276353	2.767163	0.088285
C	-0.354467	3.952654	-0.285637
C	0.356160	5.138767	-0.370580
C	1.711912	5.162951	-0.079660
C	2.349264	3.991651	0.299510
C	1.637345	2.805939	0.385735
H	-6.548423	-2.314801	0.776046
H	-6.783732	-1.022852	-1.273347
H	-4.855951	0.168006	-2.290662
H	-4.377337	-2.478964	1.966656
H	-1.543552	-0.806761	-1.497911
H	-1.416478	3.948042	-0.493238
H	-0.153830	6.049105	-0.656175
H	2.266835	6.089046	-0.144283
H	3.407237	3.997711	0.525047
H	2.152018	1.897086	0.668149
H	-1.123872	-0.881900	2.572800
H	0.444176	0.949147	2.051675
H	-1.564055	1.828905	-1.634617
Br	1.875709	-1.245060	0.301524
Br	3.663818	-2.467939	-0.517710

$TS_{I\text{-}III}$

Imaginary Freq = -19.0816 (cm^{-1})

Electronic Energy aug-cc-pVTZ = -5842.17818731 Hartree

Zero-point Energy Correction = 0.251443 Hartree

Thermal Correction to Enthalpy = 0.269320 Hartree

Thermal Correction to Free Energy = 0.200145 Hartree

Chemical symbol X, Y, Z

C	1.089787	5.415786	0.405816
C	1.806831	5.223824	-0.774381
C	1.948310	3.960857	-1.357730
C	1.337848	2.924924	-0.694676

C	0.637802	3.111231	0.504771
C	0.485355	4.359362	1.082776
C	1.010371	1.423443	-0.732796
C	0.444832	1.675158	0.653880
C	1.940875	0.265271	-0.777013
C	1.839046	-0.699782	0.178410
C	0.994947	-0.526656	1.327615
C	0.390379	0.707502	1.616070
C	2.623781	-1.956259	0.065804
C	3.965997	-1.930866	-0.307878
C	4.691312	-3.105447	-0.426558
C	4.087723	-4.327693	-0.171534
C	2.753625	-4.365162	0.204806
C	2.029331	-3.189892	0.324754
H	1.008543	6.417102	0.807406
H	2.261787	6.081184	-1.252382
H	2.504242	3.831203	-2.276247
H	-0.057858	4.522309	2.003141
H	0.213728	1.260461	-1.471764
H	4.449602	-0.979914	-0.489416
H	5.734265	-3.064657	-0.711167
H	4.653905	-5.244716	-0.262731
H	2.271791	-5.313679	0.401146
H	0.984365	-3.231770	0.604015
H	0.021805	0.908388	2.613918
H	0.959588	-1.316539	2.064605
H	2.582230	0.121083	-1.636649
Br	-2.325343	-0.407066	0.302635
Br	-4.304855	-1.243907	-0.506254

Br_I

Electronic Energy aug-cc-pVTZ = -5842.19720692 Hartree

Zero-point Energy Correction = 0.254464 Hartree

Thermal Correction to Enthalpy = 0.272596 Hartree

Thermal Correction to Free Energy = 0.204167 Hartree

Chemical symbol X, Y, Z

C	-2.206937	3.219950	1.310801
C	-1.632750	3.719276	0.140341
C	-0.837683	2.932200	-0.688823
C	-0.666493	1.625425	-0.283511
C	-1.219141	1.143052	0.888561
C	-2.007101	1.906693	1.723155
C	-0.022325	0.295582	-0.657709
C	-0.533159	-0.185164	0.749196
C	1.448870	0.238922	-0.875249
C	2.309683	-0.086959	0.095410
C	1.811255	-0.437700	1.429775
C	0.518264	-0.504754	1.743852
C	3.771906	-0.130741	-0.143074
C	4.400590	0.846182	-0.913214
C	5.763482	0.789849	-1.154930
C	6.523856	-0.242362	-0.626374
C	5.911253	-1.215953	0.148060
C	4.548586	-1.158018	0.391061

H	-2.821494	3.876950	1.911185
H	-1.821758	4.749341	-0.131137
H	-0.405770	3.333357	-1.595033
H	-2.450344	1.526852	2.633064
H	-0.554419	-0.189675	-1.475587
H	3.817854	1.667332	-1.309478
H	6.234614	1.560761	-1.749849
H	7.588394	-0.284868	-0.812549
H	6.495385	-2.026677	0.562316
H	4.080777	-1.932513	0.985042
H	0.201434	-0.741116	2.750977
H	2.543488	-0.625981	2.203930
H	1.805860	0.442605	-1.876904
Br	-1.777097	-1.764109	0.679973
Br	-3.708816	-0.747012	-1.301686

III

Electronic Energy aug-cc-pVTZ = -3267.96301952 Hartree

Zero-point Energy Correction = 0.254055 Hartree

Thermal Correction to Enthalpy = 0.269674 Hartree

Thermal Correction to Free Energy = 0.210297 Hartree

Chemical symbol X, Y, Z

C	-3.390704	2.588248	1.007570
C	-2.708646	3.321696	0.035770
C	-1.744700	2.741590	-0.785859
C	-1.519105	1.396086	-0.583907
C	-2.184204	0.677871	0.392668
C	-3.138420	1.235511	1.217268
C	-0.703521	0.200812	-1.061481
C	-1.383859	-0.573179	0.134515
C	0.781765	0.301929	-1.021137
C	1.506426	-0.119487	0.020856
C	0.845156	-0.754436	1.166817
C	-0.464825	-0.984331	1.226556
C	2.982975	0.009503	0.041256
C	3.606306	1.164604	-0.428036
C	4.987385	1.274659	-0.430647
C	5.771290	0.232814	0.041451
C	5.162942	-0.918396	0.518733
C	3.781814	-1.027014	0.522641
H	-4.131705	3.090416	1.614996
H	-2.942500	4.371470	-0.082289
H	-1.225313	3.323832	-1.534569
H	-3.664344	0.676421	1.978734
H	-1.039386	-0.164753	-2.031278
H	3.000534	1.991337	-0.775146
H	5.452168	2.181770	-0.793218
H	6.849357	0.319528	0.042667
H	5.765875	-1.737771	0.886582
H	3.320583	-1.936695	0.884742
H	-0.908597	-1.445220	2.099181
H	1.462510	-1.033186	2.010692
H	1.271084	0.714167	-1.894847
Br	-2.466820	-2.141777	-0.414184

Br_2_I'

Electronic Energy aug-cc-pVTZ = -5842.17844624 Hartree

Zero-point Energy Correction = 0.251733 Hartree

Thermal Correction to Enthalpy = 0.270425 Hartree

Thermal Correction to Free Energy = 0.199056 Hartree

Chemical symbol X, Y, Z

C	-6.025829	-0.835946	-0.133500
C	-5.522567	-0.897849	-1.432083
C	-4.158608	-0.752303	-1.704877
C	-3.350742	-0.544445	-0.614487
C	-3.856607	-0.455795	0.689161
C	-5.203500	-0.608891	0.967603
C	-1.898824	-0.383324	-0.134741
C	-2.543806	-0.095092	1.208553
C	-0.900759	0.646215	-0.534253
C	-0.346901	1.444795	0.423810
C	-0.808469	1.404945	1.786364
C	-1.973199	0.718974	2.145089
C	0.719794	2.418250	0.072251
C	0.633586	3.179511	-1.091516
C	1.634564	4.076825	-1.426941
C	2.739340	4.229938	-0.603214
C	2.833657	3.481084	0.560047
C	1.831297	2.585897	0.896084
H	-7.089648	-0.961900	0.017996
H	-6.210463	-1.068647	-2.249473
H	-3.784540	-0.807660	-2.718074
H	-5.610639	-0.550953	1.967380
H	-1.422521	-1.372144	-0.149168
H	-0.234601	3.081133	-1.729846
H	1.546868	4.663825	-2.331363
H	3.521014	4.930196	-0.864703
H	3.694093	3.588834	1.206734
H	1.924327	1.994412	1.797577
H	-2.460813	0.939368	3.086019
H	-0.338820	2.062287	2.504315
H	-0.566562	0.705986	-1.562213
Br	1.671119	-1.118499	-0.119106
Br	3.306531	-2.735257	-0.035616

$TS_{Br2\_I'-Br\_I'}$

Imaginary Freq = -104.8444 (cm^{-1})

Electronic Energy aug-cc-pVTZ = -5842.16739281 Hartree

Zero-point Energy Correction = 0.253449 Hartree

Thermal Correction to Enthalpy = 0.271090 Hartree

Thermal Correction to Free Energy = 0.203840 Hartree

Chemical symbol X, Y, Z

C	5.782618	-0.366293	0.456023
C	5.089591	-0.774573	1.596346
C	3.695233	-0.814566	1.644826
C	3.052779	-0.432313	0.491189

C	3.744460	0.012010	-0.638886
C	5.124536	0.047411	-0.698091
C	1.692432	-0.315717	-0.205427
C	2.488165	0.427820	-1.261146
C	0.450095	0.384362	0.290442
C	0.031890	1.557012	-0.448126
C	0.708751	1.958355	-1.577032
C	1.997728	1.463191	-1.959403
C	-1.130813	2.343366	0.028376
C	-1.276553	2.649098	1.380027
C	-2.353093	3.399632	1.822163
C	-3.305547	3.851134	0.921611
C	-3.172964	3.547959	-0.424915
C	-2.094716	2.800895	-0.867872
H	6.863961	-0.363673	0.478602
H	5.655934	-1.077813	2.466674
H	3.174878	-1.141174	2.534194
H	5.671568	0.380155	-1.568664
H	1.425405	-1.303560	-0.578146
H	-0.535983	2.315516	2.094944
H	-2.446732	3.633034	2.874060
H	-4.149647	4.431561	1.268042
H	-3.918419	3.883860	-1.132826
H	-2.014935	2.544588	-1.916083
H	2.606972	2.053293	-2.629691
H	0.339943	2.833665	-2.095420
H	0.282086	0.390207	1.358584
Br	-1.219777	-0.981730	-0.168115
Br	-2.668868	-3.275766	-0.016311

Br_I'

Electronic Energy aug-cc-pVTZ = -5842.18488708 Hartree

Zero-point Energy Correction = 0.254683 Hartree

Thermal Correction to Enthalpy = 0.272716 Hartree

Thermal Correction to Free Energy = 0.204612 Hartree

Chemical symbol X, Y, Z

C	5.209262	-1.088066	-0.380823
C	4.837259	0.127778	0.194356
C	3.530472	0.380813	0.611024
C	2.636762	-0.648557	0.422512
C	3.002441	-1.857888	-0.170983
C	4.295731	-2.117866	-0.585027
C	1.196530	-1.094266	0.642885
C	1.615877	-2.334110	-0.148575
C	-0.015820	-0.462164	0.002094
C	-1.083973	-1.529362	-0.223739
C	-0.678940	-2.769221	-0.553331
C	0.727029	-3.141953	-0.713714
C	-2.523267	-1.188923	-0.219833
C	-3.000562	-0.095190	-0.942344
C	-4.354160	0.190218	-0.977987
C	-5.255080	-0.607772	-0.287110
C	-4.791719	-1.694077	0.437727
C	-3.435880	-1.981473	0.472133

H	6.239052	-1.226303	-0.682094
H	5.590088	0.894231	0.321420
H	3.257795	1.329157	1.052331
H	4.594355	-3.048927	-1.045860
H	0.982392	-1.336200	1.686229
H	-2.311516	0.532073	-1.492756
H	-4.706635	1.038682	-1.548650
H	-6.312194	-0.380531	-0.311425
H	-5.485541	-2.317207	0.985890
H	-3.075751	-2.820485	1.052770
H	0.989304	-3.987125	-1.334983
H	-1.434102	-3.501082	-0.809815
H	0.227696	0.043345	-0.932121
Br	-0.626281	1.029268	1.154552
Br	0.339804	3.149384	-0.652166

III'

Electronic Energy aug-cc-pVTZ = -3267.95057846 Hartree

Zero-point Energy Correction = 0.254247 Hartree

Thermal Correction to Enthalpy = 0.269786 Hartree

Thermal Correction to Free Energy = 0.210660 Hartree

Chemical symbol X, Y, Z

C	5.293093	0.366300	0.434527
C	4.879959	-0.960167	0.305885
C	3.564421	-1.297884	-0.013570
C	2.704832	-0.239020	-0.198789
C	3.112528	1.088240	-0.050442
C	4.414147	1.432176	0.263815
C	1.280370	0.150300	-0.575542
C	1.743103	1.571667	-0.255915
C	0.047324	-0.190077	0.230172
C	-0.980284	0.928646	0.048548
C	-0.535071	2.193574	-0.063870
C	0.882541	2.553318	-0.014597
C	-2.433090	0.660729	0.120514
C	-2.972318	-0.089946	1.165271
C	-4.338445	-0.288851	1.260823
C	-5.191823	0.250902	0.308759
C	-4.667309	0.992776	-0.737382
C	-3.298861	1.195208	-0.830578
H	6.327540	0.566801	0.679855
H	5.607236	-1.747309	0.454470
H	3.260225	-2.330759	-0.111581
H	4.745026	2.454791	0.379707
H	1.073843	0.017789	-1.639683
H	-2.319321	-0.513290	1.916886
H	-4.738974	-0.867595	2.082257
H	-6.258875	0.089512	0.381014
H	-5.323364	1.410285	-1.489437
H	-2.891611	1.761004	-1.658040
H	1.172865	3.551643	0.283110
H	-1.264221	2.992929	-0.098847
H	0.283517	-0.305233	1.288143
Br	-0.591559	-1.971083	-0.310104

Br_2_I''

Electronic Energy aug-cc-pVTZ = -5842.17944520 Hartree

Zero-point Energy Correction = 0.251872 Hartree

Thermal Correction to Enthalpy = 0.270542 Hartree

Thermal Correction to Free Energy = 0.199465 Hartree

Chemical symbol X, Y, Z

C	-5.678209	-1.811384	0.376606
C	-5.813293	-1.071463	-0.797442
C	-4.733484	-0.398479	-1.377508
C	-3.534688	-0.515046	-0.718383
C	-3.401358	-1.237722	0.474334
C	-4.463568	-1.912409	1.050238
C	-2.037968	-0.166342	-0.754971
C	-2.021123	-0.797364	0.623988
C	-1.400832	1.175553	-0.787567
C	-0.486563	1.494585	0.164824
C	-0.253524	0.622479	1.292727
C	-1.116279	-0.448013	1.581485
C	0.276118	2.767200	0.088282
C	-0.354800	3.952636	-0.285644
C	0.355724	5.138811	-0.370578
C	1.711469	5.163115	-0.079640
C	2.348918	3.991869	0.299537
C	1.637102	2.806096	0.385750
H	-6.548315	-2.315123	0.776087
H	-6.783701	-1.023196	-1.273309
H	-4.855981	0.167726	-2.290660
H	-4.377209	-2.479193	1.966671
H	-1.543521	-0.806852	-1.497955
H	-1.416807	3.947932	-0.493265
H	-0.154343	6.049104	-0.656178
H	2.266311	6.089258	-0.144255
H	3.406888	3.998023	0.525089
H	2.151849	1.897290	0.668175
H	-1.123840	-0.881970	2.572777
H	0.444124	0.949153	2.051642
H	-1.564228	1.828821	-1.634610
Br	1.875851	-1.245000	0.301511
Br	3.664061	-2.467734	-0.517695

$TSBr_{2\_I''\text{-}Br\_I''}$

Imaginary Freq = -72.3215 (cm^{-1})

Electronic Energy aug-cc-pVTZ = -5842.17681759 Hartree

Zero-point Energy Correction = 0.253137 Hartree

Thermal Correction to Enthalpy = 0.270908 Hartree

Thermal Correction to Free Energy = 0.203218 Hartree

Chemical symbol X, Y, Z

C	-5.864611	-1.311091	0.373683
C	-5.913335	-0.522040	-0.776828
C	-4.765066	0.013492	-1.362815
C	-3.581903	-0.301730	-0.738523

C	-3.533872	-1.067982	0.428506
C	-4.666119	-1.600367	1.017387
C	-2.053528	-0.160759	-0.797564
C	-2.106314	-0.801372	0.571238
C	-1.263414	1.092661	-0.827387
C	-0.301095	1.288048	0.094188
C	-0.102112	0.301265	1.166850
C	-1.160605	-0.602307	1.507208
C	0.552632	2.502311	0.081852
C	0.004755	3.735717	-0.270362
C	0.786944	4.877726	-0.298698
C	2.132216	4.809690	0.031418
C	2.685304	3.591761	0.393769
C	1.902890	2.448446	0.423350
H	-6.790161	-1.698878	0.776769
H	-6.876077	-0.326822	-1.230058
H	-4.823813	0.621748	-2.254435
H	-4.641011	-2.195882	1.918818
H	-1.642690	-0.853351	-1.542390
H	-1.049877	3.805662	-0.502473
H	0.341211	5.825569	-0.568863
H	2.743686	5.701521	0.011598
H	3.733868	3.525860	0.650438
H	2.357691	1.504364	0.689138
H	-1.212945	-1.008790	2.507245
H	0.532894	0.620242	1.981379
H	-1.395633	1.788221	-1.645402
Br	1.533788	-1.233667	0.356852
Br	3.672105	-2.396912	-0.553863

Br_I''

Electronic Energy aug-cc-pVTZ = -5842.18488790 Hartree

Zero-point Energy Correction = 0.254550 Hartree

Thermal Correction to Enthalpy = 0.272675 Hartree

Thermal Correction to Free Energy = 0.203465 Hartree

Chemical symbol X, Y, Z

C	5.784448	-0.982774	-0.938573
C	5.987882	0.127239	-0.119669
C	4.946280	0.724600	0.592292
C	3.709681	0.142456	0.437805
C	3.502012	-0.954074	-0.398700
C	4.524986	-1.553274	-1.105992
C	2.246096	0.176519	0.890415
C	2.049874	-0.882941	-0.182471
C	1.308312	1.317995	0.725956
C	0.116272	1.120514	0.149859
C	-0.252158	-0.258059	-0.340429
C	0.891802	-1.091924	-0.789349
C	-0.856726	2.220984	-0.063163
C	-0.405295	3.527160	-0.263844
C	-1.295894	4.568907	-0.453945
C	-2.662039	4.328415	-0.460180
C	-3.124532	3.035229	-0.281412
C	-2.232421	1.992106	-0.088671

H	6.631460	-1.405234	-1.462159
H	6.987458	0.531911	-0.032252
H	5.124243	1.583612	1.224450
H	4.376472	-2.404926	-1.754797
H	2.148785	-0.241559	1.897928
H	0.656811	3.728512	-0.297390
H	-0.920073	5.570859	-0.612136
H	-3.358797	5.141224	-0.613406
H	-4.186528	2.830669	-0.289709
H	-2.626674	0.996445	0.058478
H	0.752668	-1.748891	-1.635921
H	-1.038976	-0.222870	-1.085081
H	1.579763	2.287625	1.121937
Br	-1.252464	-1.242616	1.162382
Br	-3.363481	-2.243151	-0.601797

III''

Electronic Energy aug-cc-pVTZ = -3267.94969835 Hartree

Zero-point Energy Correction = 0.254213 Hartree

Thermal Correction to Enthalpy = 0.269773 Hartree

Thermal Correction to Free Energy = 0.210491 Hartree

Chemical symbol X, Y, Z

C	5.241522	-0.786213	0.679073
C	4.962771	-1.660281	-0.370269
C	3.742160	-1.637905	-1.047952
C	2.837080	-0.696258	-0.616660
C	3.107812	0.164119	0.447639
C	4.311959	0.152367	1.122401
C	1.458310	-0.079379	-0.871016
C	1.736376	0.696051	0.408535
C	0.159881	-0.801702	-0.804691
C	-0.834252	-0.305805	-0.060247
C	-0.625587	0.974792	0.720049
C	0.778726	1.174634	1.184853
C	-2.141952	-0.995816	0.065552
C	-2.204774	-2.390336	0.033103
C	-3.414913	-3.053748	0.139282
C	-4.593003	-2.337973	0.292580
C	-4.544967	-0.954244	0.343984
C	-3.333253	-0.290135	0.235479
H	6.206484	-0.846643	1.164312
H	5.720185	-2.373007	-0.668542
H	3.544358	-2.322046	-1.861820
H	4.535584	0.816022	1.945930
H	1.481760	0.568146	-1.753489
H	-1.291107	-2.963135	-0.051359
H	-3.435686	-4.135145	0.116286
H	-5.538726	-2.855305	0.380225
H	-5.455935	-0.383678	0.465532
H	-3.324666	0.790556	0.261580
H	0.945712	1.611378	2.159460
H	-1.319330	1.033046	1.551116
H	0.016105	-1.694619	-1.399112
Br	-1.198518	2.563469	-0.392008

$TS_{III-4ad}$

Imaginary Freq = -582.6821 (cm^{-1})

Electronic Energy aug-cc-pVTZ = -3267.92498723 Hartree

Zero-point Energy Correction = 0.251746 Hartree

Thermal Correction to Enthalpy = 0.267259 Hartree

Thermal Correction to Free Energy = 0.208117 Hartree

Chemical symbol X, Y, Z			
C	2.711502	2.830225	-1.134544
H	3.341058	3.401004	-1.803843
C	2.755594	1.443788	-1.169024
H	3.403680	0.919537	-1.858036
C	1.927878	0.776672	-0.281425
C	1.118208	1.433344	0.602399
C	1.040962	2.816292	0.624219
H	0.388983	3.341795	1.309105
C	1.859934	3.505862	-0.259715
H	1.847049	4.587571	-0.267529
C	1.575918	-0.656105	-0.099787
C	0.466734	0.372206	1.421368
C	0.585608	-1.241322	-0.912918
H	0.853744	-2.123866	-1.483786
C	-0.886840	0.063830	1.275426
H	-1.486081	-0.102588	2.164929
C	-0.731956	-0.844108	-0.976688
H	-1.308089	-1.261135	-1.793388
C	-1.477016	-0.237500	0.055327
C	-2.958940	-0.222395	-0.036607
C	-3.686446	-1.253931	-0.630774
C	-3.659921	0.856211	0.502092
C	-5.069114	-1.203743	-0.687916
H	-3.172392	-2.118819	-1.027823
C	-5.043623	0.907353	0.445273
H	-3.108408	1.671063	0.952550
C	-5.753320	-0.122313	-0.152065
H	-5.615498	-2.018037	-1.144632
H	-5.566818	1.756969	0.863073
H	-6.833260	-0.084630	-0.198552
H	0.898137	0.232738	2.409155
Br	3.077341	-1.815651	0.296312
